# Behavioral-psychological motivations encoded in the vocal repertoire of captive Amur tiger (*Panthera tigris altaica*) cubs

**DOI:** 10.1186/s40850-021-00102-9

**Published:** 2022-01-04

**Authors:** Xuanmin Kong, Dan Liu, Atul Kathait, Yonglu Cui, Qi Wang, Shenfan Yang, Xin Li, Ming Gong, Nathan Roberts, Xiaoying Xing, Guangshun Jiang

**Affiliations:** 1 Feline Research Center of National Forestry and Grassland Administration, College of Wildlife and Protected Area, Northeast Forestry University, Harbin, Heilongjiang China; 2 Siberia Tiger Park, Harbin, Heilongjiang China; 3 School of Biosciences, Apeejay Stya University, Gurugram, Haryana India

## Abstract

**Background:**

The Amur tiger (*Panthera tigris altaica*) is the largest and one of the most endangered cats in the world. In wild and captive cats, communication is mainly dependent on olfaction. However, vocal communication also plays a key role between mother and cubs during the breeding period. How cubs express their physiological and psychological needs to their mother and companions by using acoustic signals is little known and mainly hindered by the difficult process of data collection. Here, we quantitatively summarized the vocal repertoire and behavioral contexts of captive Amur tiger cubs. The aim of the present work was to investigate the behavioral motivations of cub calls by considering influential factors of age, sex, and rearing experiences.

**Results:**

The 5335 high-quality calls from 65 tiger cubs were classified into nine call types (*Ar-1, Ar-2*, *Er*, *eee*, *Chuff*, *Growl*, *Hiss*, *Haer,* and *Roar*) produced in seven behavioral contexts. Except for *Er*, eight of the nine call types were context-specific, related to Play (*Ar-2*, *eee*, and *Roar*), Isolation (*Ar-1*), Offensive Context (*Haer*, *Growl,* and *Hiss*), and a friendly context (*Chuff*).

**Conclusions:**

The results suggest that cubs are not quiet, but instead they express rich information by emitting various call types, which are probably crucial for survival in the wild. We herein provide the first detailed spectrogram classification to indicate vocal repertoires of calls and their coding with respect to behavioral contexts in Amur tiger cubs, and we pave the steps for revealing their social communication system, which can be applied for conservation of populations. These insights can help tiger managers or keepers to improve the rearing conditions by understanding the feline cubs’ inner status and needs by monitoring their vocal information expressions and exchanges.

**Supplementary Information:**

The online version contains supplementary material available at 10.1186/s40850-021-00102-9.

## Background

Vocal signals are the essential form of communication in animals [1–3]. Animals use vocal communication to maintain social relations, including mate selection and intercourse, competition, and signal food and security. Understanding the critical role of vocalization in animal communication systems can be an essential tool for the conservation of endangered species [4]. In altricial species, infant calls can trigger adaptive care-giving behaviors from parents, which can even determine infant survival [5–7]. The acoustic parameters of kitten calls depict their emotional state and maintain their individuality [8, 9]. Some studies have indicated that non-human adult animals respond to infant calls of various mammalian species [5, 10]. In some species of, for instance, birds [11, 12], felids [13], and rodents, [14], infants use acoustic signals to compete in situations of limited resources.

The Amur tiger (*Panthera tigris altaica*), also known as the Siberian tiger, is the largest felid in the world [15]. This attractive species inhabits threatened environments in the wild, and it has been listed in the International Union for Conservation of Nature’s (IUCN) Red List as Endangered [16]. All tiger subspecies are solitary [17], which makes it is nearly impossible to locate and directly observe their behavior in the wild. In China, Amur tigers have been successfully bred in captivity for more than 30 years [18], with a large captive population of about 1000 individuals in Amur Tiger Parks.

Few studies have focused on basic postures, habits, and behaviors of captive Amur tigers [19–23]. Regarding Amur tiger vocalization, very limited information is available [24], mainly characterizing these animals’ prusten, growl, snarl, grunt, moan, meow, spit, hiss, and roar [24]. Information about vocal repertorie [25], individual signatures [26], habitat influence on call frequency [27], and physiology base of vocalization production [28–31] have been investigated in other *Felidae* species. Acoustic research on tiger cubs is also very limited. These researches focused on factors correlated with vocal development of felid calls, including body size and vocal tract length [32]. The most studied felid species regarding infant vocalization is the domestic cat (*Felis catus*) [33, 34] and the call parameters of kittens showed developmental changes, and motivational valuation of the rearing situations [35]. Regarding large cats, the vocal repertoire of calls of adult and cub cheetahs (*Acinonyx jubatus*) has been systematically described [36, 37]. Calls of cubs of many other species have also been shown to have developmental dynamics during ontogeny [38–44].

Exploring the acoustic structure and developmental dynamics of tiger cub vocalizations can provide a foundation for uncovering the communication system of tigers, especially regarding mother-offspring interaction. We hypothesized that there would be a quantitative relationship between the vocal repertoire and behavioral context of calls of captive Amur tiger cubs.

## Results

A total of 5335 samples from 65 tiger cubs were extracted for analysis. The distribution of calls differed by rearing condition and sex (Table 1).

**Table 1:** Sample size distribution in different rearing conditions and sexes

	Hand-reared			Maternal-reared		
	Female	Male	unknown	Female	Male	unknown
**Number of cubs**	20	20	1	11	1	12
**Call samples**	2055	2054	9	823	29	365

We identified eight distinct call types: *Ar* (*n* = 3679), *Er* (*n* = 628), *Eee* (*n* = 105), *Chuff* (*n* = 428), *Growl* (*n* = 67), *Hiss* (*n* = 211), *Haer* (*n* = 126), and *Roar* (*n* = 91). In this study, *Growl* was not recorded for hand-reared cubs. Not all 11 acoustic parameters were measured for each call type. For non-laryngeal sounds (i.e., *Hiss*), we did not measure the fundamental-frequency-related parameters.

### Vocal repertoire of Amur Tiger cubs

The PCA extracted four principal components (PC1–4), accumulatively explaining 87% of the sample variance, with PC5–7 capturing another 10% of the sample variance. The highest loading factors of the first four PCs were explicit. PC1 was driven by MaxFreq, Freq25, Freq50, and Freq75. PC2 showed a high correlation of temporal parameters (duration and risk time). PC3 was heavily influenced by three fundamental frequency parameters (F0, MaxF0, and MinF0). PC4 was dominated by pulse parameters (PulseNum and PulseRate, Table 2).

**Table 2:** Proportion of variance of the first four principal components, with acoustic parameters ranked by factor loadings

	PC1		PC2		PC3		PC4	
Proportion of variance (cumulative)	0.477 (0.477)		0.155 (0.632)		0.138 (0.77)		0.101 (0.871)	
**Loading variables**	Freq50	−0.376	Duration	0.578	MinF0	0.446	PulseRate	0.495
	Freq25	−0.372	Risetime	0.506	F0	0.385	PulseNum	0.358
	Freq75	−0.365			MaxF0	0.366		
	MaxFreq	−0.342						

**Fig. 1:**
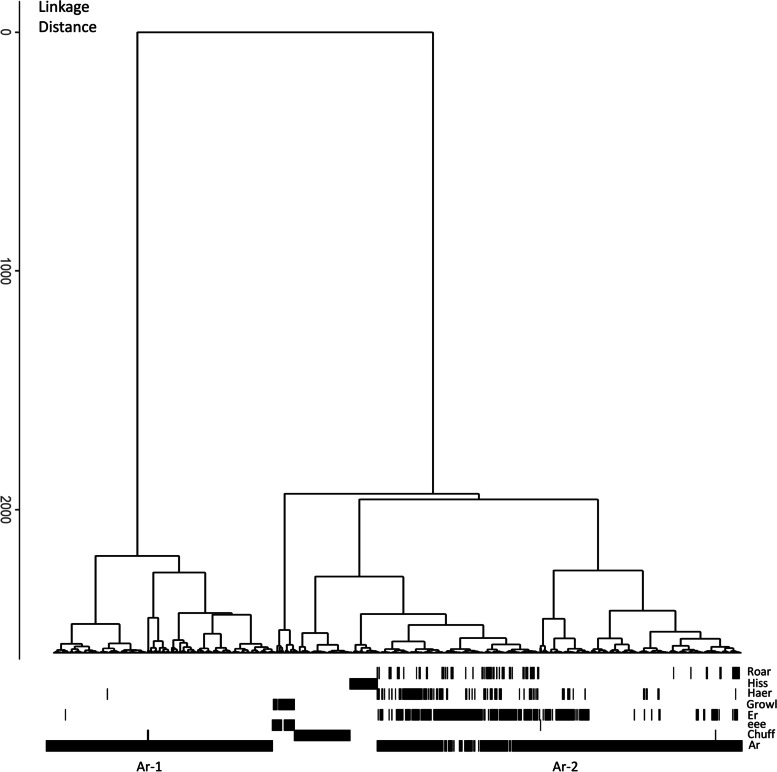
Dendrogram from hierarchical cluster analysis using PC scores. Priori call types (*Ar, Chuff, eee, Er, Growl, Haer, Hiss, Roar*) of each sample were shown below. The samples of *Ar* were labeled by two classifications (*Ar-1* and *Ar-2*)

The results of the hierarchical cluster analysis showed that the samples were distinctly clustered by three calls with rhythmic pulsation (*eee*, *Growl*, and *Chuff*) and *Hiss* (Fig. 1). However, the calls of *eee* and *Growl* were clustered together. The samples of *Ar* were separated into two groups. One was clearly clustered separately from another sample, and the other was assigned with nearly all samples of three call types (*Er*, *Haer,* and *Roar*) (Fig. 1). Based on this classification, we labeled the groups as *Ar-1* and *Ar-2*. To identify if *Ar-1* and *Ar-2* were different call types, we compared each of the 11 parameters for these two call types. Non-parametric tests showed that all acoustic parameters significantly differed between these groups (Mann-Whitney U-test: U = 85,513–1,556,825, *p* < 0.001). Therefore, we adopted *Ar-1* and *Ar-2* as a replacement for *Ar*, and the complete samples were reclassified into nine call types.

Four discriminant functions from PC1–4 revealed significant differences between the nine call types. The first two discriminant functions collectively explained 98.3% of the variance. Among them, the first discriminant function (DF1) explained 77.7% of the data and had a higher discriminating ability among the nine call types of the Amur tiger cubs. Of the 5335 experimental calls, 3834 calls were correctly classified, indicating that the percentage of correct classification was 71.9% (cross-validation = 71.8%), which is better than the percent correct discrimination attributed to chance (11.1%, binomial test, *p* < 0.001). Among the correctly classified calls, the correct classification rates of *Chuff*, *Hiss*, and *Ar-1* were high (100, 98.6, and 94.6%, respectively). The correct classification rate of *Er* was low (34.1%), with most of the samples having been assigned to *Roar* and *Ar-2*. The lowest correct classification rate was 23% (pertaining to *Haer*), with most samples having been assigned to *Er* and *Roar* (see Additional file 2).

**Fig. 2:**
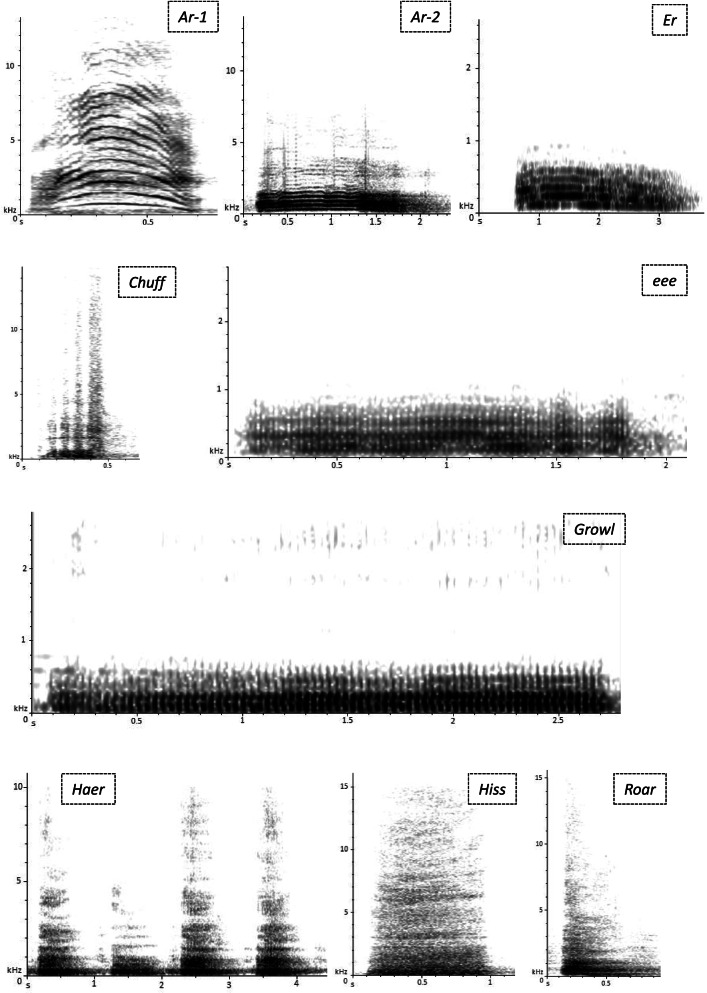
Spectrograms of nine call types of captive Amur tiger cubs. Spectrograms of *Ar-1*, *Ar-2*, *Chuff*, *Growl*, *Er*, *eee*, *Haer*, *Hiss*, and *Roar* are shown in (**A**)–(**I**), separately. The spectrograms were created at a FFT size of 2048, Hann window of 1024 samples, and overlap of 50%

The call types that were classified together in the cluster analysis also had overlaps in the result of DFA (*eee* and *Growl*; *Ar-2*, *Er*, *Haer,* and *Roar*). To identify the acoustic parameters that differed between them, we used a non-parametric test to compare 11 or 13 of their parameters. All 11 parameters between *Ar-2*, *Er*, *Haer,* and *Roar* were significantly different (Kruskal-Wallis test: *χ*^2^ = 138.857–855.801, df = 3, *p* < 0.001). Ten of the 13 parameters for *eee* and *Growl* were significantly different (Mann-Whitney U-test: U = 598.5–3479, *p* < 0.001–0.895): MaxFreq, F0, MaxF0, MinF0, Freq25, Freq50, Freq75, IQRBW, PulseNum, and PulseRate. From this analysis, nine call types produced by the Amur tiger cubs were identified (Fig. 2A–I; Table 3).

**Table 3:** Values (mean ± SD) of acoustic parameters for the call types of Amur tiger cubs. The total number of each call type is given in the parentheses

Acoustic parameter	Call types								
	***Ar-1*** (1725)	***Ar-2*** (1954)	***Chuff*** (428)	***eee*** (105)	***Er*** (628)	***Growl*** (67)	***Haer*** (126)	***Hiss*** (211)	Roar (91)
**Duration**	0.80 ± 0.36	0.95 ± 0.65	0.32 ± 0.07	1.60 ± 0.93	0.60 ± 0.88	1.78 ± 1.16	0.69 ± 0.32	0.71 ± 0.27	0.38 ± 0.19
**MaxFreq**	0.41 ± 0.24	0.42 ± 0.40	0.14 ± 0.07	0.64 ± 0.65	0.27 ± 0.54	0.52 ± 0.56	0.19 ± 0.11	0.37 ± 0.25	0.14 ± 0.06
**RiseTime**	2.17 ± 0.72	0.89 ± 0.57	0.23 ± 0.13	0.26 ± 0.24	0.37 ± 0.24	0.17 ± 0.11	0.34 ± 0.42	0.43 ± 0.36	0.62 ± 0.30
**F0**	0.46 ± 0.28	0.20 ± 0.06	0.19 ± 0.04	0.17 ± 0.08	0.16 ± 0.05	0.12 ± 0.03	0.16 ± 0.03	–	0.17 ± 0.04
**SDF0**	0.09 ± 0.05	0.04 ± 0.02	0.03 ± 0.02	0.04 ± 0.05	0.02 ± 0.01	0.03 ± 0.01	0.04 ± 0.01	–	0.04 ± 0.02
**MaxF0**	0.59 ± 0.32	0.26 ± 0.08	0.25 ± 0.10	0.24 ± 0.13	0.20 ± 0.06	0.18 ± 0.04	0.22 ± 0.05	–	0.24 ± 0.06
**MinF0**	0.26 ± 0.24	0.13 ± 0.05	0.12 ± 0.04	0.08 ± 0.03	0.12 ± 0.05	0.05 ± 0.03	0.08 ± 0.03	–	0.10 ± 0.03
**Freq25**	1.70 ± 0.47	0.59 ± 0.35	0.28 ± 0.15	0.20 ± 0.12	0.26 ± 0.13	0.16 ± 0.14	0.21 ± 0.13	0.38 ± 0.18	0.38 ± 0.17
**Freq50**	2.11 ± 0.61	0.82 ± 0.41	0.51 ± 0.35	0.30 ± 0.19	0.38 ± 0.17	0.25 ± 0.25	0.35 ± 0.28	0.59 ± 0.31	0.55 ± 0.19
**Freq75**	2.73 ± 0.91	1.08 ± 0.46	0.99 ± 0.71	0.42 ± 0.31	0.52 ± 0.22	0.38 ± 0.37	0.58 ± 0.45	1.00 ± 0.49	0.72 ± 0.18
**IQRBW**	1.03 ± 0.61	0.49 ± 0.24	0.71 ± 0.60	0.22 ± 0.21	0.26 ± 0.15	0.22 ± 0.25	0.37 ± 0.35	0.62 ± 0.36	0.34 ± 0.14
**PulseNum**	–	–	4.30 ± 1.03	82.00 ± 50.23	–	61.16 ± 42.31	–	–	–
**PulseRate**	–	–	19.03 ± 2.09	53.21 ± 9.83	–	35.70 ± 9.02	–	–	–

### Behavioral context

We initially preset six behavioral contexts in which calls were produced; however, we later added a new behavioral context named “Suckled”, resulting in a total of seven behavioral contexts. Suckled context refers to the sucking behavior given by one cub to the other, usually at the ear. We found that all calls in the Suckled and Play context were uttered from cubs who were sucked, bitten or pressed by the other, to express uncomfortable feelings.

Eight of nine call types were context-specific (i.e., given mostly in one specific context), including *Ar-1* (Isolation), *Ar-2* (Play), *Chuff* (Human-Contact), *eee* (Play), *Growl* (Offensive-to-Human), *Haer* (Offensive-to-Conspecific), *Hiss* (Offensive-to-Human), and *Roar* (Play; Table 4). Except for *Ar-2* and *Roar*, the other six context-specific call types were given in only one context in more than 80% of cases.

**Table 4:** Context-specificity calls of Amur tiger cubs

Call type	Context							N_**VOC**_
	BS	CC	HC	IS	PL	OH	OC	
***Ar-1***	0.01	0	0	**0.96**^a^	0.02	0	0	1725
***Ar-2***	0.01	0	0.01	0.31	**0.67**^a^	0	0	1954
***Chuff***	0	0.06	**0.88**^a^	0.02	0.04	0	0	428
***eee***	0.01	0	0.03	0.05	**0.88**^a^	0	0.04	105
***Er***	0.01	0.01	0.05	0.44	0.49	0	0	628
***Growl***	0	0	0	0	0.01	**0.87**^a^	0.12	67
***Haer***	0	0	0	0	0.15	0.01	**0.84**^a^	126
***Hiss***	0	0	0	0	0.06	**0.84**^a^	0.09	211
***Roar***	0	0	0	0	**0.79**^a^	0.02	0.19	91
**N**_**BC**_	42	32	426	2556	1881	239	159	5335

^a^ The percentage of calls used in each context (values greater than 65% are indicated in bold). N_VOC_ = total number of calls of each type recorded in all contexts; N_BC_ = total number of calls given in each behavioral context. Contexts include BS (Suckled), CC (Conspecific-Contact), HC (Human-Contact), IS (Isolation), PL (Play), OH (Offensive-to-Human), and OC (Offensive-to-Conspecific)

Six of the seven contexts have a unique signal-specific call type (i.e., the most common call type used in a specific context), including Isolation (*Ar-1*), Play (*Ar-2*), Human-Contact and Conspecific-Contact (*Chuff*), Offensive-to-Conspecific (*Haer*), and Offensive-to-Human (*Hiss*; Table 5). A total of five signal-specific call types were also context-specific call types. *Chuff* was a signal-specific call type for two contexts. The remaining three context-specific call types (*eee*, *Growl*, and *Roar*) were not the only call types given during a specific behavioral context.

**Table 5:** Signal-specificity call of Amur tiger cubs

Context	Call type									N_**BC**_
	***Ar-1***	***Ar-2***	***Chuff***	***eee***	***Er***	***Growl***	***Haer***	***Hiss***	***Roar***	
**BS**	0.50	0.31	0	0.02	0.17	0	0	0	0	42
**CC**	0	0.06	**0.81**^a^	0	0.13	0	0	0	0	32
**HC**	0	0.03	**0.88** ^a^	0.01	0.07	0	0	0	0	426
**IS**	**0.65** ^a^	0.24	0	0	0.11	0	0	0	0	2556
**PL**	0.02	**0.70** ^a^	0.01	0.05	0.16	0	0.01	0.01	0.04	1881
**OH**	0	0	0	0	0	0.24	0	**0.74** ^a^	0.01	239
**OC**	0.01	0.02	0	0.03	0	0.05	**0.67** ^a^	0.13	0.11	159
**N**_**VOC**_	1725	1954	428	105	628	67	126	211	91	5335

^a^The percentage of nine call types given in each context (values greater than 65% are indicated in bold). N_VOC_ = total number of calls of each type recorded in all contexts; N_BC_ = total number of calls given in each behavioral context. Contexts include BS (Suckled), CC (Conspecific-Contact), HC (Human-Contact), IS (Isolation), PL (Play), OH (Offensive-to-Human), and OC (Offensive-to-Conspecific)

*Ar-1* is the context-specific call type and signal-specific call type of the isolation context, and it is similar to the isolation call of domestic kittens [8, 33]. Based on previous data from the literature [33, 34], we regarded *Ar-1* during the isolation context as the isolation call of Amur tiger cubs.

## Discussion

### Vocal repertoire of Amur Tiger cubs

In this study, nine call types produced by captive Amur tiger cubs were identified. The number of calls of each of the nine call types was not equal. The numbers of *Ar-1* and *Ar-2* were higher than those of other call types, comprising 32.3 and 36.6% of the whole sample dataset, respectively. This is the result of random sampling and the behavioral rhythm of Amur tiger cubs. Because of the experimental environment (breeding beds) and the motor and sensory capabilities of tiger cubs under two months old, calls mostly occurred in an isolation context. According to Yu’s study [45], except for sleep and rest, play is the main routine behavior of Amur tigers after two months of age (play accounted for 12.12%, exercise for 8.11%, feeding for 6.33%, and others for 7.09%). Therefore, *Ar-1* and *Ar-2*, respectively, as the signal-specific calls of these two contexts (Isolation and Play), had a large number of calls.

Three of nine Amur tiger call types are also uttered by captive adult Sumatran Tigers, including *Chuff*, *Hiss*, and *Roar* [25]. Although *Growl* was also identified in adult Sumatran Tigers, the *Growl* in our study was different from that in Rose et al.’s study [25] which had no rhythmic pulsation. The definition herein used for this call type was based on Volodina [36] and Stanton [46]: *Growl* was a low-pitched, rumbling sound consisting of numerous short pulses with a long duration. Comparing the spectrograms and audios of *Growl* from Rose et al.’s study [25] and the calls of our study, we found that the calls of *Haer* were similar to those of *Growl*. A short harsh and repeated call named ‘coughing snarl’, which was used when attacking [24], was similar to *Haer* in our study, which was similar to coughing and was also used to show offensive behavior toward a conspecific.

In the hierarchical cluster analysis and DFA, the samples of *eee* and *Growl* were always classified together, consistent with aural and visual sense. Both are calls with rhythmic pulsation. However, the calls of *Growl* were relatively rare with lower fundamental frequency and lower three quartiles frequency, and it was mostly used in the offensive context (87% in Offensive-to-Human, 12% in Offensive-to-Conspecific), while the calls of *eee* were mostly used in the Play context (88%), which occurred when a cub felt uncomfortable in a playing situation. The samples of *Ar-2*, *Er*, *Haer,* and *Roar* were also assessed together in the quantitative analysis; however, unlike *eee* and *Growl*, they are not consistent with aural and visual sense. Spectrograms showed that their power was mostly concentrated at low frequencies, but the spectrograms of *Ar-2* and *Er* had clear harmonics (Fig. 2B–C). For aural sense, the calls of *Haer* and *Roar* exhibited higher power and shorter duration than the calls of *Ar-2* and *Er*. *Ar-2* vocalizations were a tonal sound similar to the “a” vowel, while *Er* vocalizations were also a tonal sound, but sounded like the “e” vowel. *Roar* and *Haer* sounded alike, but *Haer* usually occurred repeatedly in quick succession, while *Roar* was usually given alone or with a long interval. Regarding the behavioral context, the use of *Haer* was different from that of *Ar-2, Er,* and *Roar* (Table 4). Although the *Ar-2*, *Er,* and *Roar* vocalizations were often given in the Play context, *Roar* vocalizations were used to show stronger unwillingness to join in playing, sometimes even occurring with some threatening actions. *Roar* vocalizations of tiger cubs are powerful, harsh, and alarming, like the roar of an adult tiger [25]. *Haer* vocalizations were short and harsh, but less powerful than *Roar* and usually uttered continuously. Regarding the behavioral context, *Haer* was usually used to express offensive to conspecifics. It is possible that the acoustic parameters could not reflect the characteristics of the calls very well so that they could not be classified precisely in the quantitative analyses.

*Meow* (*Mew*/*Meow*) is the most well-known felid call [32, 47]. In our study, *Meow* was not recorded in Amur tigers. However, according to several other studies [24, 25], *Meow* has been recorded in adult tigers. In Peters [32], *Mew* calls were found in tiger cubs, but the author did not indicate from which tiger subspecies the calls were. During this study period, we found that *Meow* does exist in adult Amur tigers. Therefore, there may be vocal repertoire changes in the ontogeny of Amur tigers. More studies on the vocalization of adult Amur tigers and the comparison of vocalizations between adults and cubs need to be carried out to confirm this assumption.

### Contextual use of call types

Based on previous observations, we found that eyesight can influence the behavior of Amur tiger cubs. When a newborn tiger cub without good eyesight is separated from its mother, it behaves anxiously, constantly crawling and uttering a loud sound, even with recognizes its littermates. However, when the eyesight of a tiger cub improves during the ontogeny, it realizes its littermates seemingly, and it behaves anxiously only when it is separated from both its mother and littermates. Therefore, the definition of the isolation context considered the eyesight of tiger cubs. The cubs uttered six call types (*Ar-1*, *Ar-2*, *Chuff*, *eee*, *Er*, *Hiss*), with *Ar-1* (1658/2556) as the major call in this situation. *Ar-1* is the context-specific call and signal-specific call of the isolation context. We, therefore, deemed *Ar-1* given in the isolation context as the isolation call of Amur tiger cubs. Comparing the spectrograms of isolation calls between domestic cat kittens [8, 33] and Amur tiger cubs, we found that they were similar and both very intense and tonal in quality with a number of clear resonant frequency components. However, according to Moelk [34] and Brown et al. [33], the isolated call of domestic kittens was a complex vowel call (“mi-a:ou”), that is similar to *Meow*, while the isolated call of Amur tiger cubs (*Ar-1*) sounds like a monosyllabic “a”, different from kittens.

Haskins [48] found that kitten vocalization (from the isolation context) could prompt maternal behavior. However, we have no explicit evidence to show that the isolation call of Amur tiger cubs can influence maternal behavior. During the study, we noted that the tiger cubs would be able to emit isolation calls at a few hours after birth. From the observation of a female tiger and her babies, we found that the maternal-reared tiger cubs would not utter isolation calls until their mother left for a period of time. When keepers hold the tiger cub and make a daily check, they would be more likely to utter intense isolation calls. This situation is similar to that of kittens that were confronted with a high-arousal condition (experimenters grasped the kitten and lifted it off the ground) [8]. It was found that the kittens’ isolation calls varied their acoustic parameters between low and high-arousal conditions [8]. In our study, we did not consider the effect of arousal conditions in data collection because of the difficulty in identifying the different arousal conditions.

Three call types, *Growl*, *Haer,* and *Hiss*, were usually used in the offensive context by Amur tiger cubs. However, *Haer* was commonly used in the Offensive-to-Conspecific context, whereas *Growl* and *Hiss* were commonly used in the Offensive-to-Human context. In our study, the Offensive-to-Conspecific context always occurred during feeding. Thus, we are not sure if *Haer* is a specific call type for Offensive-to-Conspecific or for food conflicts. During the study, clash during non-feeding time was extremely rare. We suggest that this is caused by the captivity circumstance and the lack of territory consciousness in tiger cubs. *Chuff* was the signal-specific call of both Conspecific-Contact and Human-Contact contexts. It seems that Amur tiger cubs used the same vocal signal to show their amity to the other tigers and humans.

In the Play and Suckled context, the Amur tiger cubs who bit or sucked others would rarely utter calls. The calls (*Ar-2*, *eee*, and *Roar*) from these two contexts were all given by the cubs who were sucked or bitten to express uncomfortable feelings. It has been hypothesized that the playing of kittens is a way to practice predation [49]. According to the feeders, a cub was killed by another cub or cubs during play. Therefore, we suggest that the calls may be a vital indicator to ask for help from the mother.

## Conclusion

Our study identified nine call types of Amur tiger cubs (*Ar-1, Ar-2*, *Er*, *eee*, *Chuff*, *Growl*, *Hiss*, *Haer,* and *Roar*) and their contextual use. We assigned *Ar-1* from the isolation context as isolation calls of Amur tiger cubs. Our study provides advanced knowledge on the vocalization of Amur tigers. This contributes to further research on the vocal communication system of Amur tigers.

## Materials and methods

### Site and subjects

This study was conducted at the Amur Tiger Park, Harbin, Heilongjiang, China. The park was established in 1996 to protect the Amur tiger population. All the tiger cubs in our study were born in captivity, including hand-reared cubs and maternal-reared cubs. In total, 65 tiger cubs (21 males, 31 females, and 13 unknown) aged from 0 month to 10 months old were our subjects, including 41 hand-reared cubs and 24 maternal-reared cubs. All tigers at the Amur Tiger Park are managed in accordance with the Technical Code of Feeding and Management for Wild Animals-Amur Tiger (LY/T 2199-2013, State Forestry Administration 2013).

The tiger cubs at the Amur Tiger Park had two living environments; one was the breeding bed (about 1 m^2^) for the younger hand-reared cubs (under 2 months old; each litter lived in one bed); the other was the indoor enclosure (minimum of 16 m^2^) paired with an adjoining outdoor enclosure (minimum of 16 m^2^) for the older cubs (> 2 months old; hand-reared and maternal-reared cubs lived together). The breeding beds were wooden and covered with absorbent paper. The maternal-reared cubs younger than 2 months of age were usually housed in indoor enclosures with their mothers. When they were temporarily separated from their mothers, they were also kept in the breeding bed.

### Call collection

All audio recordings were obtained from May 2017 to February 2018. The call collector was isolated from the tiger cubs and had zero contact with animals. Individuals were recognized and distinguished based on their stripe patterns. Calls were recorded by random sampling during routine procedures (9:00–16:00 h) [50]. The recording equipment used was a Zoom-H5 handy recorder combined with a Zoom SSH-6 stereo shotgun microphone (Zoom Corporation, Tokyo, Japan). All recordings were made at a 44.1 kHz sampling rate and 24-bit depth as uncompressed.wav files. Distance to the cubs varied from 0.5 to 5 m. Each recording lasted 0.1 to 10 min.

According to the preliminary experiment and reference researches [37, 51], six behavioral contexts in which calls were produced were preset: (1) Isolation (when a newborn cub without good eyesight was separated from its mother, or when a cub with good eyesight was separated from its mother and littermates; e.g., the cub was crawling or walking around, frequently emitting long distance calls and looking around until it was tired or met its mother, littermates or breeders, or received food from breeders. The cubs opened their eyes at 21 ~ 25 days old.); (2) Offensive-to-Human (when a cub threatened or attacked a human, e.g., by crouching, showing bare teeth, pushing ears back, and rushing at human with raised paw and slapping); (3) Offensive-to-Conspecific (when a cub threatened or attacked a conspecific, e.g., by crouching, showing bare teeth, pushing ears back, rushing at a conspecific with raising paw and slapping); (4) Conspecific-Contact (friendly greeting with conspecific, e.g., by using head or nose to touch conspecific’s face); (5) Human-Contact (friendly greeting using head or nose through the net in front of a human, while walking to and fro); and (6) Play (friendly playing with conspecifics, e.g., chasing, pouncing, gently slapping and biting other cubs or being slapped and bit by other cubs). Whenever a call was recorded, we simultaneously labeled the context of production. All recordings of maternal-reared cubs were obtained when they were separated from their mothers for additional feeding or routine health checks. Therefore, no maternal-offspring interaction behavior was recorded in our study. Moreover, any weight data available for the tiger cubs were collected at the same time.

### Acoustic analyses

Calls with a high sound to noise ratio that did not overlap with other sounds were identified and analyzed using Raven Pro 1.5 (Cornell Laboratory of Ornithology, Ithaca, NY). To create the spectrograms, a discrete Fourier transform with an FFT size of 2048, Hann window of 1024 samples, and overlap of 50% was performed. Prior to quantitative analyses, calls were initially classified into different types based on visual and auditory differences (*Ar*, *Er*, *eee*, *Chuff*, *Growl*, *Hiss*, *Haer,* and *Roar*).

Eleven acoustic parameters were measured for each call. Duration was measured manually by selecting the entire visible portion of the call in the spectrogram window. The frequency at maximum energy (also named as peak frequency, MaxFreq) and the three quartiles (Freq25, Freq50, and Freq75), covering 25, 50, and 75% of the entire call energy, were measured automatically. The lag from the call’s start time to when maximum power first occurred (RiseTime) and the interval from Freq75 to Freq25 (interquartile range bandwidth, IQRBW) were also measured. The peak frequency contour of the fundamental frequency was used to calculate the mean (F0), standard deviation (SDF0), maximum (MaxF0), and minimum (MinF0) of the fundamental frequency band. For the calls with rhythmic pulsation, the pulse number (PulseNum) by the Standard Marker cursor was additionally counted in the spectrogram window, and the pulse rate (PulseRate) was measured.

### Statistical analyses

To determine if the acoustic parameters supported the prior classification, an agglomerative hierarchical cluster analysis was performed using a Euclidean distance matrix and Ward’s linkage, combined with a principal component analysis (PCA) for all samples. Considering that large value differences among variables can influence the variability and compromise the explanatory value of PCA, scaled unit variance was used to stabilize the variance. Then, a discriminant function analysis (DFA) was used to calculate the probability of the assignment of calls to the correct types. In DFA, the call type was used as a grouping variable, and the PC score was used as an independent variable. In order to test the result of the discriminant analysis, the “leave-one-out” classification (cross-validation) was used to distinguish one call from another. A binomial test was performed to investigate whether the values of correct assignment of calls were significantly higher than those expected by chance.

Following previous research [37, 51], for the analysis of the relationships between call type and behavioral context, a call type was classified as context-specific if it was produced in the same behavioral context in more than 65% of the cases. Because multiple calls can co-occur in the same behavioral context, a standard of 65% of occurrence was used to determine whether this was the primary call type for this context (signal-specific call). The main call type in the isolation context was determined as the isolation call of Amur tiger cubs (the context-specific call and signal-specific call in the isolation context).

Statistical analyses were performed using the SPSS software (SPSS, Inc., USA) and R version 3.5 [52]; tests were two-tailed, and significance levels were set at 0.05.

## Supplementary Information

**Additional file 1: Data S1**. Acoustic measurements of nine call types for describing the acoustics of call types.

**Additional file 2: Table S2**. Discriminant function analysis with Principal Component scores for the classification of nine call types.

## Data Availability

The dataset supporting the conclusions of this article is included within the supplementary information file (Additional files: Data S1).

## Abbreviations

MaxFreq: The frequency at maximum energy
Freq25: The frequency at 25% energy
Freq50: The frequency at 50% energy
Freq75: The frequency at 75% energy
IQRBW: Interquartile range bandwidth
F0: The mean of fundamental frequency contour
SDF0: The standard deviation of fundamental frequency contour
MaxF0: The maximum of fundamental frequency contour
MinF0: The minimum of fundamental frequency contour
PulseNum: The pulse number
PulseRate: The pulse rate
PCA: Principal component analysis
PC: Principle component
DFA: Discriminant function analysis
BS: Suckled
CC: Conspecific-Contact
HC: Human-Contact
IS: Isolation
PL: Play
OH: Offensive-to-Human
OC: Offensive-to-Conspecific
