# Supplementary material for: Behavioral-psychological motivations encoded in the vocal repertoire of captive Amur tiger (Panthera tigris altaica) cubs
Source: BMC Zool. 2022 Jan 4;7:2. doi: 10.1186/s40850-021-00102-9 (PMC10127000; doi:10.1186/s40850-021-00102-9)
Supplement: Supplementary file 1 — Additional file 1: Data S1. Acoustic measurements of nine call types for describing the acoustics of call types. [file 40850_2021_102_MOESM1_ESM.pdf]

Num	clusterT	Duration	MaxFreq	Risetime	F0	SDF0	MaxF0	MinF0	Freq25
1	Ar1	0.84	2.26	0.43	0.3	0.09	0.52	0.13	1.84
2	Ar1	1.15	2.35	0.3	0.32	0.07	0.47	0.17	1.83
3	Ar1	1.46	2.33	0.21	0.38	0.06	0.56	0.09	1.61
4	Ar1	0.87	2.09	0.52	0.33	0.1	0.43	0.13	1.62
5	Ar1	1.11	2.17	0.61	0.35	0.04	0.43	0.26	1.93
6	Ar1	1.11	2.11	0.48	0.35	0.07	0.45	0.15	1.85
7	Ar1	0.9	2.26	0.57	0.31	0.1	0.45	0.11	1.71
8	Ar1	1.01	2.13	0.59	0.37	0.09	0.5	0.17	1.5
9	Ar1	1.08	2.13	0.52	0.39	0.06	0.45	0.22	1.65
10	Ar1	1.17	2.11	0.39	0.39	0.07	0.45	0.17	1.89
11	Ar1	1.09	2.2	0.41	0.35	0.1	0.5	0.15	1.81
12	Ar1	1.1	2.24	0.54	0.39	0.08	0.5	0.19	1.48
13	Ar1	1.25	2.15	0.65	0.37	0.07	0.5	0.17	1.61
14	Ar1	0.94	2.05	0.8	0.35	0.1	0.54	0.15	1.21
15	Ar1	0.83	2.2	0.63	0.32	0.09	0.45	0.11	1.8
16	Ar1	1.12	2.2	0.57	0.32	0.07	0.43	0.17	1.95
17	Ar1	1.11	2.2	0.56	0.32	0.07	0.39	0.19	1.96
18	Ar1	0.86	2.09	0.7	0.41	0.08	0.5	0.19	2
19	Ar1	0.96	2.41	0.63	0.39	0.1	0.5	0.19	1.38
20	Ar1	1.06	2.39	0.54	0.35	0.09	0.5	0.17	1.83
21	Ar1	1.03	2.17	0.8	0.37	0.12	0.5	0.06	1.75
22	Ar1	1.1	2.2	0.71	0.36	0.08	0.5	0.19	1.85
23	Ar1	0.96	2.07	0.72	0.38	0.08	0.5	0.17	1.75
24	Ar1	0.93	2.15	0.76	0.36	0.08	0.54	0.17	1.62
25	Ar1	0.75	2.17	0.47	0.32	0.08	0.41	0.15	1.91
26	Ar1	0.82	2.35	0.41	0.32	0.07	0.41	0.17	1.76
27	Ar1	0.73	1.94	0.53	0.32	0.06	0.41	0.19	1.67
28	Ar1	0.74	2.09	0.44	0.36	0.07	0.43	0.15	1.77
29	Ar1	0.67	1.92	0.53	0.34	0.09	0.45	0.15	1.69
30	Ar1	1.12	2.09	0.26	0.3	0.08	0.45	0.13	1.65
31	Ar1	0.87	2	0.43	0.32	0.08	0.41	0.13	1.44
32	Ar1	0.79	2.26	0.48	0.32	0.08	0.43	0.11	1.41
33	Ar1	0.85	2.28	0.42	0.31	0.07	0.41	0.11	1.74
34	Ar1	0.97	2.11	0.3	0.33	0.09	0.43	0.15	1.48
35	Ar1	0.85	2.11	0.38	0.33	0.1	0.45	0.13	1.3
36	Ar1	0.9	2.24	0.33	0.32	0.09	0.45	0.15	1.59
37	Ar1	0.65	2.22	0.49	0.35	0.08	0.43	0.15	1.7
38	Ar1	0.87	2.02	0.67	0.39	0.14	0.58	0.02	1.49
39	Ar1	1.11	2.43	0.43	0.34	0.08	0.47	0.15	2.12
40	Ar1	1.08	2.63	0.41	0.34	0.09	0.5	0.15	1.79
41	Ar1	0.82	2.24	0.55	0.31	0.08	0.43	0.17	1.89
42	Ar1	0.89	2.45	0.56	0.32	0.09	0.5	0.15	1.83
43	Ar1	0.79	2.13	0.55	0.35	0.07	0.47	0.22	1.91
44	Ar1	1.08	2.22	0.51	0.4	0.05	0.47	0.28	1.74
45	Ar1	0.89	2.37	0.48	0.37	0.11	0.5	0.15	1.69
46	Ar1	0.92	2.02	0.48	0.41	0.06	0.54	0.28	1.75
47	Ar1	0.84	2.13	0.5	0.41	0.03	0.47	0.3	1.78
49	Ar1	0.81	2.26	0.48	0.39	0.08	0.52	0.19	1.78
50	Ar1	0.81	2.39	0.24	0.33	0.07	0.52	0.22	1.76
51	Ar1	0.6	2.26	0.34	0.37	0.1	0.45	0.11	1.79
52	Ar1	1.1	2.11	0.17	0.35	0.08	0.47	0.19	1.73
53	Ar1	0.7	1.36	0.58	0.39	0.05	0.5	0.26	1.68
54	Ar1	0.86	2.05	0.37	0.37	0.07	0.52	0.22	1.43

55	Ar1	0.92	2.39	0.29	0.45	0.05	0.54	0.28	2.12
56	Ar1	0.64	1.96	0.4458	0.4	0.1	0.54	0.19	1.69
57	Ar1	0.79	2.22	0.44	0.38	0.1	0.56	0.17	1.59
58	Ar1	0.79	2.28	0.5	0.41	0.05	0.5	0.26	1.72
59	Ar1	0.88	2.33	0.47	0.36	0.08	0.52	0.19	1.67
60	Ar1	0.87	2.24	0.4	0.34	0.08	0.47	0.19	1.95
61	Ar1	0.98	2.09	0.3	0.32	0.11	0.52	0.11	1.74
62	Ar1	0.74	1.36	0.5	0.4	0.14	0.58	0.17	1.4
63	Ar1	0.9	2.02	0.42	0.44	0.1	0.6	0.15	1.6
64	Ar1	0.91	2.15	0.36	0.42	0.11	0.54	0.19	1.53
65	Ar1	0.89	2.11	0.48	0.42	0.11	0.56	0.19	1.73
66	Ar1	0.89	2.05	0.48	0.43	0.1	0.54	0.19	1.91
67	Ar1	0.78	2.26	0.52	0.44	0.07	0.56	0.24	1.95
68	Ar1	0.82	2.26	0.43	0.34	0.1	0.58	0.22	1.29
69	Ar1	0.74	2.11	0.54	0.45	0.09	0.58	0.24	1.41
71	Ar1	0.81	1.79	0.46	0.47	0.12	0.6	0.17	1.34
70	Ar1	0.93	2.02	0.45	0.47	0.07	0.54	0.26	1.69
72	Ar1	0.78	2.09	0.43	0.43	0.11	0.58	0.15	1.53
73	Ar1	1.04	2.15	0.34	0.4	0.11	0.58	0.17	1.59
74	Ar1	1.05	1.79	0.48	0.44	0.1	0.58	0.22	1.29
75	Ar1	1.02	1.79	0.36	0.4	0.09	0.54	0.17	1.49
76	Ar1	0.96	1.7	0.44	0.38	0.09	0.56	0.22	1.43
78	Ar1	0.61	0.6	0.58	0.42	0.14	0.6	0.17	1.24
77	Ar1	1.1	1.4	0.35	0.49	0.1	0.58	0.15	1.34
79	Ar1	0.75	2.11	0.51	0.38	0.08	0.62	0.19	1.51
80	Ar1	0.81	1.96	0.54	0.39	0.12	0.62	0.17	1.33
81	Ar1	0.78	1.83	0.5	0.45	0.09	0.58	0.19	1.52
82	Ar1	0.82	2.09	0.41	0.41	0.13	0.6	0.15	1.34
83	Ar1	0.94	2.11	0.54	0.47	0.12	0.65	0.15	1.57
84	Ar1	1	1.83	0.6	0.46	0.15	0.62	0.15	1.23
85	Ar1	0.75	2.02	0.55	0.46	0.11	0.6	0.26	1.37
86	Ar1	0.7	1.7	0.57	0.55	0.12	0.67	0.19	1.53
87	Ar1	0.73	2	0.44	0.43	0.12	0.58	0.24	1.45
88	Ar1	0.58	1.77	0.51	0.49	0.14	0.65	0.19	1.48
89	Ar1	0.69	0.6	0.67	0.47	0.14	0.62	0.17	1.28
90	Ar1	0.84	2.13	0.48	0.41	0.14	0.62	0.19	1.59
91	Ar1	0.76	1.81	0.51	0.45	0.16	0.65	0.15	1.39
92	Ar1	1.01	0.39	0.98	0.48	0.05	0.56	0.37	1.18
93	Ar1	1.13	2.35	0.86	0.48	0.09	0.6	0.19	1.15
94	Ar1	1.25	2.09	0.63	0.38	0.15	0.6	0.11	1.41
95	Ar1	1.14	1.83	0.79	0.37	0.09	0.58	0.19	1.67
96	Ar1	1.26	2.22	0.69	0.42	0.09	0.52	0.17	1.84
97	Ar1	1.16	2.24	0.79	0.44	0.05	0.52	0.34	1.68
98	Ar1	1.12	2.05	0.62	0.42	0.07	0.52	0.26	1.69
99	Ar1	0.89	1.92	0.74	0.41	0.05	0.47	0.26	1.57
100	Ar1	0.93	2.05	0.63	0.36	0.1	0.62	0.11	1.55
101	Ar1	1.06	2.05	0.59	0.38	0.1	0.54	0.15	1.64
102	Ar1	1.23	2.07	0.48	0.31	0.14	0.52	0.11	1.31
103	Ar1	0.87	1.81	0.61	0.46	0.07	0.52	0.22	1.72
104	Ar1	0.96	2.07	0.58	0.39	0.05	0.5	0.22	1.59
105	Ar1	0.99	2.05	0.54	0.3	0.09	0.47	0.13	1.49
106	Ar1	0.88	2.15	0.65	0.35	0.09	0.45	0.13	1.54
107	Ar1	0.84	1.83	0.73	0.36	0.06	0.43	0.19	1.59
108	Ar1	0.9	1.94	0.59	0.37	0.03	0.41	0.3	1.98

109	Ar1	0.82	1.81	0.57	0.36	0.08	0.47	0.17	1.69
110	Ar1	0.84	2.09	0.53	0.35	0.12	0.5	0.11	1.29
111	Ar1	0.99	1.4	0.45	0.31	0.1	0.47	0.13	1.13
112	Ar1	1.09	2.17	0.5	0.38	0.06	0.43	0.22	0.98
113	Ar1	0.84	2.2	0.54	0.35	0.08	0.45	0.17	1.34
114	Ar1	0.96	1.25	0.83	0.36	0.06	0.45	0.22	1.34
116	Ar1	0.95	1.4	0.63	0.37	0.1	0.47	0.09	1.14
115	Ar1	0.98	3.08	0.68	0.34	0.1	0.54	0.15	0.65
117	Ar1	1.01	2.37	0.58	0.3	0.1	0.41	0.11	1.17
118	Ar1	1.18	2.05	0.48	0.32	0.11	0.5	0.11	0.98
119	Ar1	1.01	1.89	0.71	0.35	0.07	0.41	0.22	0.94
120	Ar1	1.09	1.89	0.68	0.33	0.08	0.41	0.17	1.14
121	Ar1	0.98	2.02	0.71	0.34	0.1	0.54	0.09	1.55
122	Ar1	1.11	1.92	0.71	0.31	0.11	0.45	0.11	1.38
123	Ar1	1.43	1.98	0.52	0.34	0.06	0.41	0.17	1.57
125	Ar1	0.91	1.49	0.28	0.31	0.09	0.5	0.17	0.95
124	Ar1	1.32	2.09	0.55	0.34	0.09	0.43	0.13	1.4
126	Ar1	0.8	1.72	0.25	0.32	0.08	0.45	0.19	1.11
127	Ar1	0.78	1.31	0.32	0.36	0.08	0.45	0.13	1.14
128	Ar1	0.61	1.51	0.5	0.34	0.03	0.39	0.3	1.31
129	Ar1	0.81	1.96	0.24	0.31	0.08	0.41	0.15	1.25
132	Ar1	0.63	1.59	0.41	0.32	0.08	0.41	0.11	1.02
130	Ar1	0.6	1.62	0.39	0.25	0.09	0.5	0.11	1.27
131	Ar1	1	1.77	0.16	0.29	0.09	0.47	0.15	0.49
135	Ar1	0.59	1.85	0.25	0.33	0.07	0.43	0.19	1.27
133	Ar1	0.64	1.55	0.32	0.28	0.04	0.39	0.22	1.46
134	Ar1	0.73	1.59	0.29	0.28	0.05	0.39	0.19	1.33
137	Ar1	0.66	1.72	0.29	0.32	0.06	0.43	0.19	1.02
136	Ar1	0.6	1.7	0.21	0.36	0.06	0.43	0.19	1.35
138	Ar1	0.63	1.83	0.25	0.33	0.05	0.41	0.22	1.2
139	Ar1	0.48	1.81	0.33	0.32	0.06	0.43	0.19	1.1
141	Ar1	0.36	1.72	0.21	0.4	0.02	0.45	0.34	0.92
140	Ar1	0.57	1.34	0.33	0.31	0.05	0.41	0.22	1.32
142	Ar1	0.38	0.82	0.23	0.42	0.06	0.52	0.32	0.87
143	Ar1	0.49	0.9	0.07	0.41	0.11	0.56	0.13	0.92
144	Ar1	0.52	1.59	0.19	0.41	0.02	0.52	0.37	0.79
146	Ar1	0.33	0.39	0.29	0.44	0.03	0.47	0.39	0.59
145	Er	0.14	1.1	0.06	0.36	0.09	0.45	0.22	0.74
153	Ar1	0.24	1.7	0.19	0.32	0.09	0.43	0.22	0.69
147	Ar1	0.43	1.25	0.35	0.34	0.07	0.45	0.24	0.83
148	Ar1	0.43	1.59	0.24	0.33	0.07	0.41	0.19	1.18
149	Ar1	0.53	1.77	0.25	0.36	0.04	0.43	0.28	1.03
150	Ar1	0.35	1.85	0.2	0.31	0.06	0.41	0.22	1.1
151	Ar1	0.52	1.53	0.15	0.38	0.04	0.43	0.28	0.78
152	Ar1	0.52	1.53	0.16	0.38	0.05	0.45	0.26	0.79
155	Ar1	0.29	1.85	0.18	0.35	0.1	0.47	0.19	0.82
154	Ar1	0.31	1.98	0.16	0.33	0.07	0.41	0.22	1.05
157	Ar1	0.34	1.64	0.23	0.35	0.11	0.47	0.15	1.35
156	Ar1	0.39	1.68	0.22	0.37	0.05	0.43	0.28	1.09
158	Ar1	0.51	1.53	0.17	0.37	0.1	0.5	0.17	1.04
159	Ar1	0.75	2.54	0.35	0.34	0.08	0.43	0.11	1.02
160	Ar1	0.64	2.26	0.36	0.31	0.11	0.45	0.06	0.98
162	Ar1	0.85	1.92	0.34	0.28	0.06	0.39	0.15	1.7
161	Ar1	0.77	2.3	0.32	0.26	0.07	0.41	0.17	1.38

163	Ar1	0.64	1.92	0.45	0.35	0.08	0.45	0.15	1.08
164	Ar1	0.86	2.3	0.42	0.33	0.07	0.41	0.17	1.15
165	Ar1	0.9	2.17	0.39	0.33	0.06	0.45	0.22	1.34
166	Ar1	1.15	1.74	0.19	0.32	0.07	0.54	0.13	1.47
168	Ar1	0.76	2	0.44	0.33	0.08	0.5	0.19	0.91
167	Ar1	0.89	2.15	0.31	0.31	0.11	0.5	0.11	1.11
169	Ar1	0.78	2.07	0.46	0.33	0.06	0.43	0.22	1.53
170	Ar1	0.74	1.79	0.49	0.32	0.07	0.43	0.17	1.57
171	Ar1	0.66	1.89	0.53	0.3	0.08	0.39	0.15	1.22
172	Ar1	0.87	2	0.4	0.28	0.06	0.37	0.17	1.38
173	Ar1	0.72	1.87	0.49	0.34	0.06	0.41	0.09	1.88
174	Ar1	0.8	2.09	0.48	0.32	0.06	0.41	0.15	1.65
175	Ar1	0.67	1.66	0.46	0.32	0.06	0.41	0.15	1.18
176	Ar1	0.66	1.85	0.3	0.32	0.11	0.32	0.11	1.18
177	Ar1	0.62	2.17	0.37	0.28	0.06	0.37	0.17	1.45
178	Ar1	0.77	1.83	0.37	0.32	0.06	0.39	0.19	1.58
181	Ar1	0.56	2.07	0.49	0.31	0.08	0.39	0.15	1.23
182	Ar1	0.9	1.55	0.33	0.31	0.07	0.41	0.17	1.29
180	Ar1	1.1	2.22	0.21	0.36	0.06	0.45	0.19	0.92
186	Ar1	0.66	1.62	0.29	0.3	0.07	0.43	0.19	1.1
187	Ar1	0.8	1.64	0.07	0.29	0.07	0.39	0.15	1.61
183	Ar1	0.51	2.11	0.34	0.3	0.06	0.34	0.17	1.48
184	Ar1	0.47	2.22	0.31	0.29	0.07	0.37	0.15	1.44
185	Ar1	0.51	1.92	0.29	0.28	0.05	0.39	0.17	1.56
189	Ar1	0.73	2.02	0.22	0.32	0.07	0.41	0.17	1.51
190	Ar1	0.59	2.11	0.26	0.31	0.06	0.45	0.19	1.43
188	Ar1	0.48	1.27	0.37	0.31	0.05	0.39	0.22	1.58
191	Ar1	0.67	1.94	0.23	0.35	0.06	0.43	0.19	1.05
192	Ar1	0.67	2.05	0.27	0.3	0.04	0.37	0.22	1.69
193	Ar1	0.54	2.09	0.27	0.28	0.05	0.37	0.19	1.52
195	Ar1	0.7	2.05	0.32	0.34	0.1	0.45	0.11	1.13
196	Ar1	0.7	2.05	0.35	0.33	0.09	0.5	0.15	1.15
194	Ar1	0.5	1.68	0.3	0.31	0.06	0.39	0.17	1.58
197	Ar1	0.71	1.77	0.38	0.37	0.06	0.43	0.22	1.27
198	Ar1	0.87	2.26	0.22	0.31	0.08	0.47	0.19	1.21
199	Ar1	0.68	1.92	0.23	0.38	0.1	0.52	0.06	1.65
201	Ar1	0.38	2.24	0.21	0.32	0.07	0.41	0.17	1.73
204	Ar1	0.59	2.09	0.24	0.35	0.07	0.41	0.15	1.59
200	Ar1	0.96	1.79	0.2	0.38	0.04	0.43	0.28	1.26
205	Ar1	0.58	1.94	0.4	0.42	0.11	0.52	0.15	1.47
206	Ar1	0.69	1.89	0.33	0.42	0.12	0.58	0.13	1.46
203	Ar1	0.45	2.35	0.31	0.33	0.07	0.43	0.17	1.46
207	Ar1	0.62	2.15	0.38	0.38	0.13	0.54	0.15	1.3
208	Ar1	0.61	2.3	0.29	0.37	0.12	0.54	0.15	1.43
210	Ar1	0.77	2.09	0.26	0.37	0.12	0.52	0.15	1.46
211	Ar1	0.71	2.11	0.35	0.39	0.08	0.45	0.19	1.71
212	Ar1	0.74	1.92	0.31	0.37	0.08	0.5	0.19	1.52
209	Ar1	0.55	1.72	0.41	0.44	0.1	0.58	0.13	1.37
213	Ar1	0.63	2.26	0.56	0.37	0.03	0.41	0.3	1.58
214	Ar1	0.64	2	0.51	0.37	0.07	0.58	0.17	1.44
215	Ar1	1.08	1.89	0.18	0.35	0.09	0.56	0.15	1.48
217	Ar1	0.6	2.05	0.19	0.38	0.08	0.47	0.19	1.33
218	Ar1	0.73	2.13	0.19	0.4	0.08	0.47	0.22	1.67
221	Ar1	0.81	1.83	0.59	0.35	0.11	0.56	0.13	1.03

216	Ar1	0.64	2.09	0.5	0.4	0.06	0.54	0.26	1.03
222	Ar1	1.29	2	0.45	0.41	0.08	0.5	0.17	0.98
223	Ar1	0.89	1.87	0.53	0.33	0.09	0.47	0.19	1.25
219	Ar1	0.6	1.94	0.3	0.38	0.09	0.5	0.17	1.44
220	Ar1	0.58	1.87	0.33	0.38	0.09	0.5	0.17	1.44
224	Ar1	0.79	1.85	0.63	0.37	0.1	0.52	0.19	1.06
225	Ar1	0.76	1.68	0.63	0.34	0.12	0.47	0.15	1.27
226	Ar1	0.66	1.36	0.52	0.43	0.11	0.52	0.17	1.14
227	Ar1	0.78	0.3	0.62	0.35	0.1	0.47	0.17	1.29
228	Ar1	1.04	0.97	0.38	0.37	0.11	0.52	0.15	1.02
229	Ar1	0.83	1.51	0.41	0.37	0.1	0.5	0.15	1.05
230	Ar1	0.49	1.87	0.36	0.39	0.09	0.47	0.19	0.93
232	Ar1	0.75	1.98	0.26	0.32	0.11	0.5	0.15	1.24
234	Ar1	0.63	2.02	0.28	0.33	0.13	0.54	0.15	1.26
236	Ar1	0.57	1.7	0.3	0.38	0.08	0.52	0.17	1.44
231	Ar1	0.68	1.79	0.42	0.35	0.07	0.5	0.22	1.28
237	Ar1	0.42	2.17	0.27	0.29	0.14	0.5	0.09	1.14
233	Ar1	0.9	1.81	0.28	0.37	0.04	0.41	0.3	1.28
238	Ar1	0.74	2	0.37	0.34	0.06	0.45	0.17	1.55
235	Ar1	0.66	1.83	0.34	0.45	0.08	0.52	0.19	1.18
239	Ar1	0.72	1.62	0.41	0.34	0.09	0.41	0.17	1.44
240	Ar1	0.9	2.15	0.29	0.31	0.06	0.5	0.19	1.28
243	Ar1	0.83	1.7	0.44	0.31	0.05	0.39	0.22	1.4
244	Ar1	0.82	2.02	0.44	0.32	0.05	0.39	0.19	1.38
245	Ar1	0.82	1.94	0.52	0.39	0.09	0.54	0.19	1.31
241	Ar1	0.72	1.49	0.49	0.34	0.05	0.41	0.22	1.38
242	Ar1	0.72	1.85	0.46	0.32	0.09	0.45	0.13	1.36
246	Ar1	0.71	1.96	0.54	0.39	0.06	0.47	0.24	1.65
247	Ar1	0.91	1.81	0.44	0.4	0.05	0.45	0.28	1.63
248	Ar1	0.74	1.44	0.51	0.35	0.08	0.45	0.19	1.46
249	Ar1	0.91	1.83	0.39	0.37	0.07	0.47	0.19	1.6
250	Ar1	0.91	2.07	0.52	0.38	0.04	0.45	0.26	1.31
263	Ar1	0.37	1.74	0.21	0.57	0.05	0.67	0.43	1.01
264	Ar1	0.42	1.83	0.19	0.52	0.09	0.62	0.3	1.19
267	Ar1	0.57	0.09	0.16	0.42	0.13	0.58	0.19	0.76
251	Ar1	0.82	0.97	0.36	0.52	0.07	0.62	0.37	0.72
252	Ar1	0.72	0.67	0.63	0.53	0.12	0.69	0.26	0.51
253	Ar1	0.54	0.99	0.37	0.57	0.11	0.69	0.28	0.83
254	Ar1	0.42	1.01	0.38	0.5	0.07	0.6	0.39	1.16
255	Ar1	0.61	1.49	0.24	0.46	0.08	0.54	0.28	0.79
256	Ar1	0.53	0.82	0.39	0.49	0.06	0.6	0.37	0.96
257	Ar1	0.6	1.12	0.35	0.5	0.05	0.56	0.39	0.69
258	Ar1	0.69	0.88	0.53	0.56	0.08	0.65	0.41	1.01
259	Ar1	0.67	0.84	0.52	0.55	0.18	0.8	0.28	0.58
260	Ar1	0.77	0.84	0.53	0.53	0.14	0.8	0.3	0.78
261	Ar1	0.66	1.34	0.29	0.55	0.06	0.69	0.43	1.21
262	Ar1	0.68	1.06	0.4	0.61	0.08	0.73	0.39	1.23
269	Ar1	0.54	1.85	0.3	0.42	0.04	0.52	0.34	1.07
270	Ar1	0.57	1.81	0.15	0.41	0.07	0.54	0.26	0.82
265	Ar1	0.72	1.34	0.25	0.37	0.13	0.56	0.13	0.62
266	Ar1	0.68	0.54	0.44	0.45	0.1	0.6	0.17	0.5
277	Ar1	1.41	1.62	0.22	0.33	0.08	0.58	0.19	0.64
268	Ar1	0.61	1.68	0.24	0.39	0.06	0.5	0.3	1.36
284	Ar1	0.64	1.89	0.33	0.44	0.11	0.6	0.26	1.3

285	Ar1	0.5	1.57	0.35	0.45	0.14	0.62	0.19	1.36
271	Ar1	0.55	1.25	0.37	0.46	0.08	0.56	0.24	0.53
272	Ar1	0.49	1.85	0.23	0.41	0.1	0.52	0.19	0.67
273	Ar1	1.24	1.64	0.44	0.44	0.09	0.54	0.24	0.69
274	Ar1	1.23	1.29	0.21	0.49	0.13	0.62	0.15	0.84
275	Ar1	1.23	1.16	0.13	0.39	0.14	0.6	0.13	0.72
276	Ar1	1.32	0.93	0.13	0.44	0.13	0.56	0.11	0.67
287	Ar1	0.62	1.96	0.46	0.45	0.08	0.56	0.28	1.48
278	Ar1	1.43	1.4	0.15	0.42	0.11	0.54	0.15	0.63
279	Ar1	0.55	1.1	0.43	0.48	0.1	0.58	0.26	0.81
280	Ar1	1.03	1.18	0.28	0.51	0.1	0.65	0.24	0.69
281	Ar1	0.77	0.95	0.55	0.45	0.06	0.52	0.28	0.81
282	Ar1	0.62	0.9	0.35	0.45	0.13	0.58	0.11	0.71
283	Ar1	0.61	0.93	0.5	0.5	0.07	0.56	0.3	0.84
290	Ar1	0.58	1.98	0.21	0.51	0.13	0.69	0.15	1.14
291	Ar1	0.55	2.3	0.3	0.47	0.09	0.65	0.3	1.32
286	Ar1	0.62	1.79	0.4	0.44	0.09	0.58	0.28	1.59
292	Ar1	0.7	2.15	0.14	0.42	0.15	0.6	0.15	1.54
288	Ar1	0.59	2.2	0.27	0.48	0.09	0.65	0.28	1.85
289	Ar1	0.47	2.13	0.27	0.45	0.09	0.58	0.28	1.95
295	Ar1	0.59	2.09	0.33	0.47	0.08	0.54	0.24	1.49
298	Ar1	0.55	2.13	0.24	0.42	0.09	0.54	0.26	1.67
299	Ar1	0.71	1.79	0.36	0.41	0.09	0.58	0.26	1.52
293	Ar1	0.48	1.94	0.37	0.46	0.1	0.58	0.26	1.78
294	Ar1	0.64	1.96	0.31	0.44	0.11	0.6	0.24	1.76
300	Ar1	0.72	2.07	0.23	0.41	0.17	0.62	0.09	1.46
296	Ar1	0.48	1.98	0.28	0.43	0.11	0.58	0.15	1.58
297	Ar1	0.57	1.94	0.26	0.43	0.08	0.56	0.28	1.52
301	Ar1	0.6	2.13	0.39	0.47	0.1	0.56	0.19	1.7
302	Ar1	0.54	2.17	0.34	0.44	0.12	0.56	0.19	1.74
304	Ar1	0.67	1.55	0.14	0.49	0.1	0.67	0.26	1.33
306	Ar1	0.6	1.87	0.34	0.5	0.1	0.65	0.32	1.57
307	Ar1	0.71	2.02	0.26	0.47	0.1	0.65	0.32	1.43
303	Ar1	0.5	1.77	0.14	0.49	0.11	0.62	0.3	1.46
310	Ar1	0.64	1.64	0.35	0.52	0.08	0.67	0.32	1.24
305	Ar1	0.5	1.79	0.13	0.49	0.1	0.62	0.28	1.49
316	Ar1	0.96	1.64	0.31	0.51	0.12	0.75	0.24	1.07
323	Ar1	0.28	1.89	0.2	0.49	0.04	0.58	0.43	1.28
308	Ar1	0.7	1.49	0.16	0.46	0.17	0.69	0.17	0.97
309	Ar1	0.67	1.83	0.33	0.55	0.04	0.62	0.41	1.55
326	Ar1	0.48	1.98	0.16	0.45	0.09	0.6	0.32	1.14
311	Ar1	0.68	1.74	0.35	0.51	0.06	0.62	0.39	1.06
312	Ar1	0.76	1.27	0.45	0.55	0.08	0.65	0.32	1.13
313	Ar1	0.74	1.57	0.32	0.5	0.05	0.6	0.41	1.17
314	Ar1	0.56	1.7	0.33	0.5	0.1	0.67	0.26	1.06
315	Ar1	0.88	1.36	0.49	0.59	0.08	0.69	0.34	1.18
327	Ar1	0.4	2.05	0.22	0.37	0.11	0.52	0.22	1.32
317	Ar1	1	1.59	0.11	0.53	0.17	0.75	0.15	1.1
318	Ar1	0.07	2.11	0.03	0.47	0.07	0.58	0.41	1.46
319	Ar1	0.07	1.92	0.04	0.52	0.06	0.58	0.47	1.56
320	Ar1	0.13	2.05	0.05	0.45	0.02	0.47	0.43	1.27
321	Ar1	0.1	1.98	0.08	0.46	0.02	0.5	0.43	1.59
322	Ar1	0.17	1.81	0.09	0.46	0.02	0.5	0.43	1.77
328	Ar1	0.5	1.96	0.18	0.41	0.11	0.58	0.22	1.27

324	Ar1	0.26	1.68	0.07	0.47	0.03	0.58	0.43	1.62
325	Ar1	0.44	1.66	0.23	0.47	0.03	0.54	0.43	1.2
329	Ar1	0.44	1.98	0.2	0.44	0.08	0.54	0.28	1.44
330	Ar1	0.28	2.05	0.19	0.45	0.07	0.58	0.32	1.53
332	Ar1	0.38	1.98	0.24	0.43	0.08	0.54	0.3	1.5
334	Ar1	0.52	2.17	0.18	0.42	0.08	0.58	0.22	1.22
335	Ar1	0.43	2.05	0.21	0.41	0.09	0.54	0.22	1.34
331	Ar1	0.56	1.94	0.14	0.42	0.1	0.58	0.24	1.69
337	Ar1	0.52	2.07	0.22	0.46	0.09	0.65	0.26	0.67
333	Ar1	0.55	1.85	0.16	0.36	0.09	0.54	0.24	1.37
339	Ar1	0.29	0.73	0.27	0.4	0.07	0.52	0.26	1.32
341	Ar1	0.31	1.94	0.25	0.39	0.13	0.52	0.19	0.57
336	Ar1	0.43	0.5	0.3	0.5	0.09	0.6	0.28	0.71
342	Ar1	0.48	2.22	0.24	0.39	0.06	0.54	0.24	0.91
338	Ar1	0.54	0.5	0.35	0.5	0.1	0.6	0.28	0.91
343	Ar1	0.44	1.34	0.24	0.43	0.07	0.5	0.19	1.3
340	Ar1	0.49	1.81	0.18	0.43	0.08	0.54	0.26	1.07
344	Ar1	0.44	1.92	0.2	0.41	0.1	0.5	0.19	1.19
345	Ar1	0.47	2.05	0.27	0.4	0.07	0.45	0.26	1.39
349	Ar1	0.49	1.87	0.34	0.43	0.09	0.56	0.24	1.35
350	Ar1	0.5	1.89	0.41	0.42	0.12	0.6	0.22	1.02
352	Ar1	0.52	2.24	0.41	0.47	0.1	0.62	0.22	0.68
346	Ar1	0.55	0.54	0.27	0.42	0.11	0.56	0.24	0.67
347	Ar1	0.61	1.66	0.23	0.39	0.06	0.52	0.3	1.37
348	Ar1	0.62	1.74	0.27	0.45	0.05	0.54	0.26	1.28
356	Ar1	0.87	1.96	0.72	0.22	0.02	0.28	0.15	1.15
357	Ar1	0.78	2.35	0.61	0.19	0.05	0.28	0.11	1
351	Ar1	0.47	2.11	0.28	0.4	0.09	0.52	0.24	1.39
358	Ar1	0.94	1.89	0.51	0.19	0.05	0.28	0.06	1.32
353	Ar1	0.46	1.66	0.27	0.47	0.09	0.58	0.26	1
354	Ar1	0.51	1.98	0.21	0.45	0.09	0.58	0.26	1.16
355	Ar1	0.59	1.62	0.18	0.42	0.1	0.58	0.22	1.48
360	Ar1	0.79	2.05	0.57	0.2	0.02	0.22	0.15	1.46
362	Ar1	0.68	1.92	0.51	0.2	0.04	0.26	0.09	1.54
363	Ar1	0.86	1.85	0.43	0.22	0.04	0.3	0.11	1.12
359	Ar1	0.92	1.68	0.59	0.22	0.04	0.3	0.13	1.04
365	Ar1	0.72	2.11	0.59	0.24	0.05	0.32	0.13	0.64
361	Ar1	0.7	0.17	0.69	0.2	0.04	0.26	0.13	1.4
366	Ar1	0.77	1.49	0.48	0.25	0.06	0.32	0.11	1.1
367	Ar1	0.76	1.68	0.49	0.21	0.05	0.32	0.13	1.28
364	Ar1	0.79	1.83	0.43	0.25	0.05	0.34	0.09	1.31
368	Ar1	0.68	0.3	0.56	0.22	0.07	0.34	0.11	0.79
369	Ar1	0.65	0.34	0.54	0.25	0.05	0.37	0.15	0.73
372	Ar1	1.27	1.59	0.72	0.21	0.05	0.28	0.11	0.89
377	Ar1	1	2.43	0.57	0.23	0.04	0.3	0.13	1.17
378	Ar1	1.01	2.2	0.46	0.21	0.03	0.28	0.13	1.48
370	Ar1	0.97	1.89	0.31	0.23	0.05	0.32	0.11	1.01
371	Ar1	1.81	1.77	0.21	0.2	0.04	0.26	0.06	1.61
379	Ar1	1.09	2.33	0.49	0.24	0.02	0.28	0.17	0.92
373	Ar1	0.8	1.77	0.66	0.16	0.04	0.22	0.06	1.24
374	Ar1	1.36	2.54	0.38	0.18	0.03	0.22	0.13	0.84
375	Ar1	1.33	2.3	0.32	0.19	0.03	0.22	0.11	1.05
376	Ar1	0.88	0.78	0.7	0.23	0.04	0.3	0.13	1.1
380	Ar1	0.64	2.41	0.48	0.23	0.04	0.26	0.13	1.58

381	Ar1	0.78	2.24	0.44233	0.19	0.02	0.24	0.15	1.91
384	Ar1	1.05	1.7	0.364471	0.21	0.06	0.32	0.06	1.35
385	Ar1	0.76	2.2	0.51	0.21	0.04	0.32	0.13	1.03
386	Ar1	0.85	1.85	0.48	0.22	0.04	0.32	0.11	1.17
382	Ar1	0.61	2.28	0.48	0.2	0.05	0.26	0.06	1.39
383	Ar1	0.61	2.28	0.48	0.2	0.05	0.26	0.06	1.39
387	Ar1	0.73	1.92	0.33	0.23	0.05	0.28	0.06	1.55
388	Ar1	0.82	1.89	0.24	0.23	0.05	0.32	0.06	1.47
391	Ar1	0.75	1.92	0.4	0.23	0.04	0.28	0.15	1.32
396	Ar1	0.52	2.28	0.39	0.26	0.05	0.32	0.11	1.24
398	Ar1	0.57	2.5	0.34	0.22	0.06	0.28	0.11	1.33
389	Ar1	0.86	2.17	0.2	0.22	0.03	0.26	0.15	1.78
390	Ar1	0.86	2.17	0.2	0.22	0.03	0.26	0.15	1.78
400	Ar1	0.58	2.17	0.28	0.23	0.04	0.28	0.13	1.73
392	Ar1	0.56	2.22	0.42	0.22	0.03	0.26	0.15	1.45
393	Ar1	0.56	1.68	0.42	0.22	0.04	0.28	0.15	1.49
394	Ar1	0.56	2.76	0.37	0.22	0.04	0.28	0.15	1.27
395	Ar1	0.6	1.77	0.39	0.22	0.03	0.26	0.15	1.88
404	Ar1	0.67	1.74	0.37	0.26	0.05	0.37	0.15	1.25
397	Ar1	0.52	2.28	0.39	0.26	0.05	0.32	0.11	1.24
405	Ar1	0.67	2.22	0.28	0.25	0.04	0.32	0.17	1.61
399	Ar1	0.54	2.41	0.35	0.21	0.05	0.3	0.13	1.48
407	Ar1	0.95	2.13	0.051738	0.22	0.05	0.3	0.13	1.33
401	Ar1	0.5	2.84	0.3	0.22	0.04	0.28	0.15	1.38
402	Ar1	0.63	2.82	0.25	0.24	0.06	0.3	0.13	1.27
403	Ar1	0.5	2.54	0.32	0.24	0.05	0.32	0.15	1.64
409	Ar1	0.37	2.33	0.27	0.25	0.05	0.3	0.17	1.2
410	Ar1	0.62	1.94	0.23	0.25	0.06	0.32	0.11	1.32
406	Ar1	0.51	2.45	0.32	0.24	0.04	0.28	0.17	1.74
412	Ar1	0.96	2.13	0.22	0.24	0.03	0.3	0.17	0.83
408	Ar1	0.44	1.79	0.39	0.23	0.03	0.26	0.15	1.57
415	Ar1	0.58	1.7	0.49	0.16	0.02	0.19	0.11	1.56
416	Ar1	1.13	2.11	0.17	0.24	0.03	0.3	0.15	1.14
411	Ar1	0.31	2.71	0.2	0.15	0.03	0.24	0.06	1.62
417	Ar1	0.84	2.17	0.36	0.23	0.03	0.28	0.15	1.51
413	Ar1	0.72	2.26	0.181491	0.19	0.04	0.24	0.09	1.26
414	Ar1	0.87	2.43	0.37	0.19	0.03	0.24	0.06	1.52
418	Ar1	0.66	2.54	0.32	0.24	0.05	0.3	0.11	1.73
419	Ar1	0.83	1.87	0.32	0.25	0.04	0.32	0.15	1.58
435	Ar1	0.6	0.09	0.41	0.25	0.05	0.32	0.17	1.23
436	Ar1	0.91	1.85	0.13	0.28	0.03	0.32	0.19	0.96
438	Ar1	0.78	0.41	0.38	0.32	0.09	0.41	0.13	0.52
420	Ar1	0.66	2.3	0.28	0.27	0.04	0.32	0.15	1.39
421	Ar1	0.52	3.12	0.3	0.22	0.04	0.28	0.15	1.62
422	Ar1	0.6	1.85	0.36	0.22	0.04	0.28	0.13	1.25
423	Ar1	0.69	2.45	0.2	0.2	0.03	0.26	0.15	1.32
424	Ar1	0.51	1.62	0.35	0.24	0.04	0.28	0.15	1.21
425	Ar1	0.54	1.96	0.31	0.22	0.04	0.28	0.15	1.29
426	Ar1	0.59	2.15	0.27	0.26	0.04	0.32	0.13	1.2
427	Ar1	0.59	1.77	0.28	0.21	0.04	0.3	0.13	1.53
428	Ar1	0.48	1.79	0.4	0.22	0.03	0.3	0.17	1.33
429	Ar1	0.58	1.96	0.28	0.2	0.04	0.26	0.15	1.72
430	Ar1	0.47	1.27	0.43	0.23	0.03	0.3	0.17	1.58
431	Ar1	0.43	1.53	0.34	0.24	0.04	0.3	0.17	1.24

432	Ar1	0.5	1.68	0.3	0.23	0.05	0.32	0.13	1.18
433	Ar1	0.54	1.92	0.26	0.23	0.05	0.28	0.11	1.43
434	Ar1	0.42	1.29	0.34	0.24	0.03	0.3	0.17	1.55
443	Ar1	0.53	1.85	0.32	0.22	0.05	0.3	0.13	0.86
446	Ar1	0.81	1.92	0.13	0.2	0.02	0.24	0.15	1.38
437	Ar1	0.48	1.23	0.36	0.24	0.05	0.3	0.15	1.33
451	Ar1	0.63	1.83	0.35	0.2	0.04	0.3	0.13	0.78
439	Ar1	0.4	1.83	0.3	0.23	0.03	0.3	0.19	1.65
440	Ar1	0.43	1.4	0.35	0.24	0.04	0.3	0.17	1.71
441	Ar1	0.59	1.81	0.31	0.28	0.05	0.32	0.15	1.53
442	Ar1	0.53	1.49	0.39	0.23	0.05	0.34	0.15	1.41
457	Ar1	0.37	1.94	0.21	0.22	0.07	0.32	0.06	1.01
444	Ar1	0.49	1.87	0.29	0.19	0.03	0.24	0.15	1.36
445	Ar1	0.46	1.96	0.27	0.21	0.03	0.24	0.13	1.5
465	Ar1	0.54	1.81	0.123064	0.19	0.02	0.24	0.15	1.3
447	Ar1	0.43	1.87	0.33	0.19	0.03	0.22	0.13	1.38
448	Ar1	0.46	1.4	0.36	0.2	0.03	0.24	0.13	1.43
449	Ar1	0.53	1.59	0.34	0.2	0.03	0.26	0.13	1.48
450	Ar1	0.8	1.94	0.18	0.22	0.03	0.26	0.09	0.98
474	Ar1	0.59	2.24	0.22	0.21	0.05	0.32	0.11	0.62
452	Ar1	0.5	1.81	0.23	0.21	0.04	0.26	0.15	1.26
453	Ar1	0.56	1.87	0.22	0.19	0.04	0.28	0.13	1.26
454	Ar1	0.4	1.68	0.31	0.22	0.03	0.26	0.15	1.31
455	Ar1	0.46	1.96	0.23	0.21	0.03	0.26	0.15	1.57
456	Ar1	0.52	2	0.19	0.21	0.05	0.3	0.13	1.27
481	Ar1	0.57	2.28	0.32	0.18	0.03	0.24	0.13	1.55
458	Ar1	0.42	2.41	0.24	0.22	0.03	0.26	0.15	1.11
459	Ar1	0.31	1.98	0.13	0.21	0.03	0.26	0.15	1.21
460	Ar1	0.36	1.83	0.24	0.23	0.03	0.28	0.17	1.17
461	Ar1	0.42	1.79	0.27	0.23	0.04	0.28	0.17	1.26
462	Ar1	0.45	1.92	0.22	0.23	0.03	0.28	0.17	1.51
463	Haer	0.24	0.24	0.1	0.26	0.03	0.32	0.22	0.58
464	Ar1	0.23	2.45	0.15	0.18	0.02	0.22	0.15	1.27
487	Ar1	0.7	2.58	0.089735	0.21	0.03	0.28	0.11	1.79
466	Haer	0.35	2.33	0.13	0.17	0.05	0.26	0.09	1.05
467	Ar1	0.49	2	0.23	0.19	0.02	0.24	0.15	1.46
468	Ar1	0.4	2.07	0.21	0.19	0.03	0.24	0.13	1.33
469	Ar1	0.51	2.02	0.26	0.2	0.03	0.26	0.15	1.48
470	Ar1	0.49	2.2	0.17	0.22	0.04	0.26	0.13	1.48
471	Ar1	0.48	1.68	0.25	0.21	0.02	0.28	0.17	1.69
472	Ar1	0.44	0.32	0.31	0.26	0.05	0.34	0.17	1.14
473	Ar1	0.44	1.92	0.19	0.22	0.03	0.26	0.15	1.49
490	Ar1	0.66	2.3	0.22	0.32	0.04	0.41	0.22	1.73
475	Ar1	0.87	0.09	0.28	0.22	0.04	0.32	0.17	0.92
493	Ar1	0.51	1.92	0.38	0.35	0.06	0.39	0.19	1.6
477	Ar1	0.58	1.94	0.17	0.24	0.05	0.3	0.13	1.47
478	Ar1	0.47	1.81	0.24	0.23	0.03	0.28	0.15	1.76
479	Ar1	0.5	1.96	0.26	0.25	0.04	0.3	0.15	1.55
480	Ar1	0.41	1.79	0.31	0.24	0.03	0.28	0.17	1.66
496	Ar1	0.48	2.39	0.31	0.28	0.05	0.37	0.22	1.42
482	Ar1	0.6	1.64	0.26	0.21	0.05	0.26	0.09	1.55
483	Ar1	0.47	2.71	0.28	0.2	0.04	0.28	0.13	1.15
484	Ar1	0.59	1.85	0.29	0.22	0.03	0.26	0.15	1.65
485	Ar1	0.49	1.89	0.31	0.23	0.04	0.28	0.15	1.54

486	Ar1	0.5	1.89	0.31	0.23	0.04	0.28	0.15	1.41
502	Ar1	0.58	2.22	0.43	0.33	0.08	0.41	0.13	1.47
488	Ar1	0.5	1.89	0.3	0.2	0.04	0.26	0.13	1.57
489	Ar1	0.5	1.94	0.34	0.22	0.04	0.26	0.15	1.48
503	Ar1	0.62	2.56	0.4	0.32	0.07	0.41	0.17	1.08
491	Ar1	0.46	1.7	0.31	0.32	0.06	0.39	0.19	1.85
492	Ar1	0.53	1.92	0.19	0.32	0.09	0.45	0.11	1.62
505	Ar1	0.9	1.94	0.2	0.35	0.05	0.41	0.17	1.63
494	Ar1	0.71	1.89	0.28	0.34	0.06	0.47	0.22	1.38
495	Ar1	0.49	2.05	0.3	0.26	0.06	0.37	0.17	1.71
508	Ar1	0.44	1.81	0.39	0.3	0.02	0.34	0.26	1.66
513	Ar1	0.66	2.09	0.27	0.33	0.09	0.43	0.11	1.46
514	Ar1	0.65	2.35	0.26	0.31	0.09	0.41	0.13	1.06
499	Ar1	0.48	2.13	0.23	0.33	0.04	0.39	0.22	1.59
515	Ar1	0.58	2.28	0.25	0.33	0.07	0.39	0.13	1.54
501	Ar1	0.7	2.13	0.41	0.32	0.04	0.41	0.19	1.68
516	Ar1	0.87	2.2	0.15	0.31	0.06	0.39	0.13	1.7
517	Ar1	0.64	2.07	0.32	0.31	0.04	0.34	0.17	1.96
504	Ar1	0.56	1.62	0.42	0.27	0.07	0.39	0.17	1.64
523	Ar1	0.5	2.35	0.29	0.26	0.03	0.32	0.17	1.75
506	Ar1	0.54	1.59	0.38	0.29	0.07	0.37	0.17	1.67
507	Ar1	0.58	2	0.26	0.29	0.04	0.37	0.17	1.66
524	Ar1	0.46	2.39	0.26	0.25	0.03	0.34	0.19	1.39
509	Ar1	0.51	2.39	0.25	0.28	0.04	0.39	0.19	1.57
510	Ar1	0.56	2.28	0.31	0.26	0.05	0.34	0.17	1.8
511	Ar1	0.48	1.64	0.38	0.31	0.05	0.37	0.19	1.62
512	Ar1	0.51	1.89	0.37	0.28	0.06	0.37	0.17	1.62
527	Ar1	0.43	2.17	0.23	0.27	0.07	0.39	0.09	1.52
536	Ar1	0.4	2.26	0.13	0.28	0.05	0.34	0.17	1.68
538	Ar1	0.53	2.52	0.2	0.34	0.03	0.39	0.22	0.88
542	Ar1	0.62	1.98	0.47	0.28	0.03	0.32	0.19	1.54
543	Ar1	0.47	1.94	0.542184	0.25	0.05	0.32	0.15	1.52
518	Ar1	0.57	1.85	0.49	0.29	0.05	0.34	0.17	1.6
519	Ar1	0.54	1.92	0.39	0.28	0.04	0.34	0.17	1.81
520	Ar1	0.75	1.55	0.1	0.3	0.05	0.39	0.15	1.45
521	Ar1	0.36	1.81	0.26	0.26	0.05	0.32	0.15	1.65
522	Ar1	0.5	1.87	0.2	0.27	0.05	0.34	0.17	1.5
544	Ar1	1.21	1.89	0.069775	0.25	0.04	0.3	0.17	1.23
545	Ar1	0.63	1.98	0.42	0.23	0.06	0.32	0.13	1.31
525	Ar1	0.57	1.85	0.2	0.25	0.05	0.32	0.15	1.58
526	Ar1	0.51	2.37	0.16	0.25	0.05	0.32	0.17	1.64
546	Ar1	0.79	1.7	0.39	0.27	0.05	0.34	0.19	1.01
528	Ar1	0.43	2.17	0.23	0.27	0.07	0.39	0.09	1.52
529	Ar1	0.68	2.28	0.22	0.26	0.04	0.34	0.17	1.17
530	Ar1	0.57	2.05	0.19	0.29	0.05	0.34	0.11	1.68
531	Ar1	0.57	1.96	0.22	0.26	0.05	0.32	0.15	1.59
547	Ar1	0.58	2.07	0.45	0.22	0.05	0.34	0.11	1.24
533	Ar1	0.48	1.31	0.11	0.28	0.05	0.34	0.15	1.38
534	Ar1	0.41	1.89	0.13	0.25	0.05	0.3	0.15	1.73
535	Ar1	0.49	1.68	0.18	0.26	0.06	0.37	0.19	1.65
548	Ar1	0.79	2.22	0.29	0.23	0.04	0.34	0.15	1.35
537	Ar1	0.5	1.89	0.15	0.25	0.06	0.39	0.17	1.7
549	Ar1	0.63	2.11	0.38	0.24	0.05	0.37	0.13	1.11
539	Ar1	0.51	2.13	0.22	0.28	0.05	0.37	0.19	1.05

540	Ar1	0.47	2.02	0.19	0.26	0.03	0.32	0.17	1.73
541	Ar1	0.46	1.83	0.09	0.25	0.04	0.3	0.15	1.75
550	Ar1	0.82	1.92	0.21	0.25	0.05	0.3	0.13	1.66
552	Ar1	0.57	2.22	0.32	0.24	0.04	0.32	0.11	1.62
554	Ar1	0.56	1.98	0.43	0.25	0.03	0.3	0.17	1.67
555	Ar1	0.58	2.26	0.31	0.23	0.06	0.3	0.11	1.52
557	Ar1	0.66	1.98	0.23	0.28	0.03	0.34	0.22	1.73
558	Ar1	0.57	2	0.26	0.27	0.07	0.37	0.15	1.33
566	Ar1	0.75	2.17	0.27	0.29	0.07	0.37	0.11	1.06
569	Ar1	0.87	1.96	0.56	0.24	0.06	0.3	0.11	1.6
570	Ar1	0.69	2.39	0.55	0.25	0.04	0.3	0.13	1.75
551	Ar1	0.7	1.83	0.42	0.28	0.04	0.34	0.19	1.14
572	Ar1	0.75	2.48	0.53	0.26	0.03	0.32	0.17	1.22
553	Ar1	0.5	2.33	0.35	0.24	0.05	0.28	0.15	1.45
573	Ar1	0.96	2.17	0.36	0.28	0.1	0.39	0.09	1.04
574	Ar1	0.82	2.3	0.46	0.26	0.05	0.32	0.17	1.55
556	Ar1	0.53	1.77	0.33	0.24	0.05	0.3	0.15	1.56
575	Ar1	0.95	1.96	0.42	0.28	0.05	0.37	0.17	1.45
576	Ar1	0.9	1.94	0.49	0.28	0.06	0.34	0.13	1.51
559	Ar1	0.47	1.27	0.41	0.27	0.06	0.34	0.19	1.54
579	Ar1	1.15	1.87	0.38	0.29	0.09	0.41	0.06	1.38
561	Ar1	0.69	1.68	0.31	0.3	0.04	0.37	0.19	1.48
562	Ar1	0.51	1.66	0.35	0.27	0.06	0.32	0.17	1.49
563	Ar1	0.72	2.15	0.35	0.28	0.05	0.34	0.15	1.43
564	Ar1	0.64	1.94	0.39	0.26	0.05	0.34	0.17	1.68
565	Ar1	0.71	1.92	0.26	0.29	0.04	0.34	0.15	1.58
581	Ar1	0.94	1.85	0.47	0.31	0.07	0.41	0.13	1.55
567	Ar1	0.59	1.4	0.35	0.27	0.08	0.39	0.09	1.46
568	Ar1	0.88	2.22	0.47	0.21	0.04	0.26	0.13	1.98
582	Ar1	0.76	2.45	0.32	0.24	0.06	0.3	0.13	1.93
584	Ar1	0.64	2.41	0.48	0.26	0.04	0.32	0.19	1.11
571	Ar1	1.1	2.5	0.34	0.23	0.05	0.34	0.13	1.44
588	Ar1	1.06	2.37	0.49	0.24	0.03	0.3	0.13	1.38
589	Ar1	0.9	2.35	0.46	0.24	0.05	0.3	0.13	1.76
590	Ar1	0.79	2.48	0.47	0.22	0.04	0.26	0.11	1.8
591	Ar1	0.85	2	0.54	0.24	0.03	0.3	0.15	1.64
592	Ar1	0.84	0.39	0.64	0.36	0.07	0.43	0.13	0.95
577	Ar1	0.88	1.98	0.52	0.24	0.05	0.37	0.09	1.8
578	Ar1	1.06	2.2	0.43	0.27	0.03	0.32	0.19	1.75
593	Ar1	0.79	2.05	0.49	0.27	0.07	0.37	0.13	0.86
594	Ar1	0.8	2.26	0.45	0.24	0.05	0.3	0.13	1.74
596	Ar1	1.46	2.2	0.52	0.22	0.05	0.3	0.11	1.95
597	Ar1	1.86	2.26	0.42	0.2	0.03	0.28	0.13	1.95
583	Ar1	0.6	2.61	0.39	0.25	0.04	0.3	0.17	1.64
598	Ar1	1.15	2.35	0.82	0.24	0.03	0.3	0.15	1.33
585	Ar1	0.56	1.89	0.39	0.25	0.06	0.32	0.13	1.75
586	Ar1	0.68	2.07	0.31	0.28	0.06	0.37	0.11	1.85
587	Ar1	0.65	1.98	0.44	0.27	0.06	0.32	0.15	1.71
599	Ar1	1.02	2.5	0.81	0.23	0.04	0.28	0.13	1.35
600	Ar1	1.1	1.96	0.8	0.22	0.04	0.32	0.15	1.51
603	Ar1	1.01	1.94	0.67	0.24	0.05	0.32	0.15	1.52
604	Ar1	1.52	1.92	0.369073	0.27	0.08	0.37	0.02	1.59
605	Ar1	0.94	2.09	0.67	0.23	0.03	0.26	0.15	1.64
607	Ar1	0.86	2.24	0.59	0.22	0.04	0.3	0.13	1.94

608	Ar1	0.81	2.26	0.69	0.25	0.05	0.34	0.09	1.5
595	Ar1	0.82	1.83	0.49	0.26	0.06	0.34	0.11	1.59
609	Ar1	1.11	2	0.52	0.24	0.08	0.34	0.09	1.3
612	Ar1	0.97	1.98	0.53	0.3	0.07	0.41	0.13	1.81
613	Ar1	1.18	2.3	0.39	0.3	0.07	0.41	0.15	1.7
614	Ar1	0.92	3.79	0.63	0.33	0.06	0.43	0.15	0.89
615	Ar1	0.98	1.98	0.64	0.31	0.08	0.41	0.11	1.33
601	Ar1	0.82	2.3	0.66	0.23	0.04	0.3	0.15	1.63
602	Ar1	0.82	2.3	0.66	0.23	0.04	0.3	0.15	1.63
618	Ar1	1	2.39	0.77	0.3	0.03	0.34	0.22	1.38
619	Ar1	1.16	1.94	0.73	0.27	0.06	0.39	0.15	1.51
620	Ar1	1.07	2.17	0.72	0.33	0.06	0.39	0.15	1.12
606	Ar1	1.39	2.13	0.44	0.22	0.04	0.28	0.11	1.65
621	Ar1	1.4	2.02	0.48	0.31	0.07	0.39	0.15	1.34
622	Ar1	1.23	1.96	0.53	0.3	0.07	0.43	0.13	1.71
623	Ar1	0.98	2.3	0.7	0.29	0.08	0.41	0.13	1.57
610	Ar1	0.81	2.22	0.66	0.25	0.07	0.43	0.06	1.45
611	Ar1	1.1	2.17	0.55	0.26	0.03	0.32	0.19	1.73
624	Ar1	1.25	2.24	0.39	0.28	0.07	0.39	0.17	1.89
625	Ar1	0.85	2.2	0.58	0.3	0.07	0.41	0.15	1.74
626	Ar1	0.91	2.43	0.51	0.3	0.06	0.37	0.13	1.98
627	Ar1	0.86	2	0.54	0.32	0.08	0.43	0.15	1.54
616	Ar1	1.01	2.07	0.72	0.27	0.07	0.37	0.15	1.71
617	Ar1	1.01	2.07	0.72	0.27	0.07	0.37	0.15	1.71
628	Ar1	0.79	2.78	0.58	0.29	0.05	0.5	0.19	1.32
629	Ar1	0.99	2.2	0.51	0.32	0.04	0.43	0.19	1.95
630	Ar1	1.02	2.37	0.45	0.3	0.06	0.39	0.15	1.76
631	Ar1	1.1	1.79	0.54	0.26	0.06	0.37	0.11	1.71
632	Ar1	1.24	2.05	0.42	0.25	0.06	0.34	0.13	1.6
633	Ar1	1.49	2.02	0.23	0.28	0.03	0.34	0.19	1.93
634	Ar1	1.04	2.3	0.52	0.27	0.06	0.37	0.13	1.49
635	Ar1	1.09	2.17	0.62	0.34	0.08	0.41	0.13	1
637	Ar1	0.97	2.22	0.58	0.29	0.07	0.39	0.15	1.39
639	Ar1	0.87	1.96	0.36	0.3	0.07	0.39	0.17	1.64
640	Ar1	0.78	2.35	0.41	0.35	0.05	0.39	0.22	1.59
641	Ar1	0.65	1.96	0.56	0.35	0.05	0.47	0.19	1.75
642	Ar1	1.18	2.22	0.41	0.38	0.07	0.47	0.17	1.54
643	Ar1	1.33	2.22	0.34	0.35	0.07	0.45	0.13	1.71
644	Ar1	1.07	2.39	0.39	0.34	0.06	0.41	0.19	1.96
645	Ar1	0.95	2.22	0.48	0.33	0.07	0.43	0.15	1.87
647	Ar1	0.89	2.07	0.42	0.3	0.09	0.52	0.09	1.62
648	Ar1	1	1.89	0.36	0.32	0.08	0.45	0.17	1.72
636	Ar1	1.06	2.24	0.57	0.31	0.05	0.37	0.15	1.84
649	Ar1	0.69	2.33	0.49	0.32	0.11	0.41	0.06	1.28
638	Ar1	1.18	2.56	0.45	0.29	0.04	0.34	0.19	1.62
650	Ar1	1.01	2.22	0.35	0.35	0.08	0.43	0.09	1.3
651	Ar1	0.98	1.98	0.42	0.35	0.07	0.43	0.15	1.26
652	Ar1	0.79	2.24	0.4	0.24	0.04	0.32	0.19	1.98
654	Ar1	0.63	2.8	0.51	0.25	0.05	0.3	0.17	1.83
656	Ar1	0.93	2.05	0.56	0.28	0.04	0.37	0.22	1.81
657	Ar1	1.01	2.26	0.38	0.27	0.07	0.37	0.17	1.95
658	Ar1	0.78	2.26	0.41	0.3	0.05	0.39	0.15	1.9
646	Ar1	1.55	1.57	0.12	0.34	0.09	0.45	0.15	1.55
660	Ar1	0.99	2.09	0.38	0.33	0.04	0.39	0.22	1.7

661	Ar1	1.02	1.85	0.42	0.32	0.05	0.41	0.19	1.67
665	Ar1	1.04	2.24	0.63	0.33	0.07	0.39	0.17	0.91
666	Ar1	0.88	1.62	0.75	0.27	0.07	0.34	0.11	1.6
667	Ar1	0.89	2.11	0.4	0.34	0.08	0.43	0.09	1.31
668	Ar1	0.86	1.87	0.47	0.3	0.08	0.39	0.11	1.59
653	Ar1	0.69	2.15	0.51	0.25	0.05	0.39	0.13	1.68
669	Ar1	1.14	2.2	0.33	0.29	0.09	0.47	0.09	1.4
655	Ar1	0.84	2.65	0.47	0.27	0.04	0.32	0.17	2.06
673	Ar1	1.31	1.85	0.51	0.25	0.05	0.32	0.09	1.75
674	Ar1	1.65	2.17	0.3	0.19	0.05	0.3	0.13	1.79
675	Ar1	1.07	1.55	0.43	0.25	0.05	0.32	0.17	1.48
659	Ar1	0.95	1.98	0.39	0.29	0.05	0.37	0.19	1.88
676	Ar1	1.3	2.22	0.34	0.23	0.05	0.3	0.13	1.45
677	Ar1	0.88	1.74	0.62	0.23	0.07	0.34	0.09	1.34
662	Ar1	0.77	2.56	0.59	0.25	0.06	0.32	0.13	1.92
663	Ar1	1.01	2.43	0.52	0.26	0.04	0.32	0.15	1.99
664	Ar1	1.01	2.43	0.52	0.26	0.04	0.32	0.15	1.99
678	Ar1	0.83	2.28	0.52	0.23	0.05	0.32	0.11	1.96
679	Ar1	0.81	2.58	0.42	0.25	0.04	0.28	0.13	2.03
680	Ar1	0.88	1.85	0.39	0.24	0.05	0.32	0.13	1.32
681	Ar1	0.65	2.3	0.48	0.21	0.04	0.26	0.13	1.99
682	Ar1	0.67	2.3	0.49	0.29	0.07	0.34	0.06	1.15
670	Ar1	1.48	2.33	0.24	0.26	0.06	0.37	0.13	1.9
671	Ar1	0.97	2.69	0.52	0.2	0.05	0.26	0.06	1.87
672	Ar1	0.97	2.69	0.52	0.2	0.05	0.26	0.06	1.87
683	Ar1	0.53	2.2	0.26	0.27	0.07	0.37	0.15	1.83
685	Ar1	0.9	2.15	0.18	0.25	0.04	0.34	0.19	1.78
687	Ar1	0.77	2.22	0.27	0.32	0.05	0.37	0.19	1.5
689	Ar1	0.68	2.05	0.28	0.29	0.05	0.34	0.19	1.77
690	Ar1	0.67	2.45	0.3	0.32	0.09	0.43	0.09	0.99
691	Ar1	0.63	2.26	0.35	0.32	0.08	0.39	0.15	1.28
692	Ar1	0.69	2.26	0.4	0.28	0.04	0.41	0.19	1.92
693	Ar1	0.8	2.15	0.37	0.3	0.06	0.41	0.19	1.38
696	Ar1	0.67	2.07	0.47	0.37	0.04	0.43	0.28	1.13
697	Ar1	0.59	2.28	0.35	0.26	0.06	0.39	0.15	2.02
698	Ar1	0.66	2.45	0.2	0.31	0.05	0.39	0.15	2.14
684	Ar1	0.47	2.71	0.29	0.26	0.05	0.32	0.15	1.83
700	Ar1	0.63	2.22	0.3	0.33	0.03	0.37	0.24	2.2
686	Ar1	0.62	1.94	0.4	0.29	0.03	0.34	0.19	1.78
701	Ar1	0.67	2.2	0.31	0.34	0.05	0.43	0.26	2.05
688	Ar1	0.77	1.89	0.27	0.3	0.04	0.37	0.19	1.68
702	Ar1	0.72	2.26	0.33	0.33	0.04	0.41	0.26	2
705	Ar1	0.9	2.35	0.22	0.28	0.05	0.37	0.15	1.68
706	Ar1	0.83	2.43	0.26	0.29	0.05	0.39	0.15	1.52
710	Ar1	0.75	2.33	0.33	0.23	0.05	0.3	0.13	1.43
711	Ar1	0.68	2.35	0.36	0.22	0.05	0.3	0.13	1.29
712	Ar1	0.84	1.94	0.38	0.25	0.06	0.32	0.09	1.44
695	Ar1	0.51	2.93	0.27	0.29	0.05	0.34	0.19	1.59
713	Ar1	0.74	2.3	0.38	0.24	0.03	0.3	0.15	1.56
715	Ar1	0.81	2.15	0.48	0.25	0.06	0.32	0.13	1.64
716	Ar1	0.94	2.11	0.34	0.26	0.05	0.34	0.13	1.74
718	Ar1	0.72	2.22	0.5	0.22	0.04	0.3	0.15	1.59
720	Ar1	0.93	2.24	0.28	0.24	0.03	0.32	0.19	1.94
721	Ar1	0.65	2.39	0.39	0.23	0.06	0.3	0.09	1.72

722	Ar1	0.72	2.02	0.12	0.27	0.04	0.32	0.19	1.2
703	Ar1	0.65	2.05	0.34	0.32	0.05	0.39	0.19	1.58
704	Ar1	0.72	2.26	0.26	0.27	0.07	0.39	0.13	1.4
725	Ar1	0.45	2.65	0.225186	0.21	0.03	0.28	0.15	1.67
727	Ar1	0.66	2.33	0.14	0.28	0.04	0.34	0.19	1.89
707	Ar1	0.46	1.79	0.11	0.27	0.06	0.39	0.19	1.37
728	Ar1	0.59	2.11	0.17	0.28	0.06	0.39	0.13	2.04
709	Ar1	0.83	2.02	0.07	0.26	0.04	0.3	0.15	1.63
729	Ar1	0.48	2.63	0.3	2.89	0.37	3.7	2.56	4.15
730	Ar1	0.93	4.89	0.24	2.54	0.2	2.93	2.13	3.77
731	Ar1	0.41	2.82	0.29	2.91	0.22	3.21	2.37	3.96
735	Ar1	0.36	2.17	0.24	2.32	0.2	2.69	2.02	2.23
714	Ar1	0.73	2.33	0.45	0.25	0.06	0.32	0.13	1.73
738	Ar1	0.61	2.82	0.37	2.43	0.29	2.84	1.94	2.28
829	Ar1	0.4	1.96	0.24	0.95	0.12	1.14	0.67	1.47
717	Ar1	0.78	2.26	0.45	0.23	0.05	0.28	0.15	1.69
830	Ar1	0.55	1.92	0.41	0.91	0.2	1.14	0.5	1.41
719	Ar1	0.63	0.8	0.54	0.28	0.05	0.37	0.19	1.41
846	Ar1	0.52	1.79	0.19	0.73	0.12	0.93	0.5	1.4
874	Ar1	0.52	1.81	0.33	0.53	0.12	0.73	0.34	1.29
875	Ar1	0.43	1.74	0.26	0.6	0.06	0.69	0.47	1.5
723	Ar1	0.71	1.96	0.1	0.25	0.04	0.28	0.15	1.72
724	Ar1	0.55	2.2	0.22	0.24	0.04	0.28	0.15	1.68
876	Ar1	0.44	1.74	0.28	0.59	0.06	0.69	0.39	1.44
726	Ar1	0.43	3.57	0.27	0.25	0.04	0.28	0.15	1.69
878	Ar1	0.42	1.87	0.24	0.54	0.12	0.73	0.39	1.32
880	Ar1	0.59	2.07	0.31	0.56	0.1	0.69	0.43	1.15
881	Ar1	0.54	2.05	0.22	0.5	0.13	0.75	0.32	1.43
882	Ar1	0.91	2.13	0.11	0.58	0.1	0.75	0.43	1.08
883	Ar1	0.23	2.26	0.08	0.58	0.05	0.69	0.52	1.38
732	Ar1	0.2	2.13	0.08	2.08	0.18	2.41	1.85	2.07
733	Ar1	0.23	3.32	0.14	2.34	0.08	2.45	2.17	2.89
734	Ar1	0.21	2.24	0.16	2.2	0.05	2.33	2.11	2.91
884	Ar1	0.52	2.3	0.17	0.6	0.06	0.67	0.47	1.92
736	Ar1	0.18	2.41	0.04	2.23	0.24	2.69	2	2.21
737	Ar1	0.18	2.48	0.06	2.35	0.26	2.82	2.09	2.33
885	Ar1	0.34	2.02	0.14	0.56	0.13	0.71	0.3	1.56
739	Ar1	0.42	2.37	0.26	2.42	0.29	2.82	1.94	2.4
740	Ar1	0.42	2.28	0.21	2.03	0.45	2.48	0.97	3.07
741	Ar1	0.26	4.29	0.1	2.11	0.14	2.33	1.79	2.55
742	Ar1	0.45	2.15	0.2	2	0.2	2.2	1.53	2.33
743	Ar1	0.13	3.01	0.1	1.78	0.27	2.2	1.51	2.56
744	Ar1	0.32	1.96	0.1	1.84	0.25	2.17	1.21	1.83
745	Ar1	0.3	2	0.1	1.99	0.1	2.28	1.83	1.11
746	Ar1	0.1	1.03	0.07	1.03	0.02	1.08	1.01	0.91
747	Ar1	0.09	1.08	0.03	1.08	0.02	1.12	1.08	1.09
748	Ar1	0.3	0.97	0.21	0.98	0.03	1.03	0.93	0.97
749	Ar1	0.12	0.93	0.05	0.92	0.07	1.01	0.8	0.9
750	Ar1	0.15	0.9	0.03	0.91	0.04	1.01	0.86	0.99
751	Ar1	0.11	0.97	0.04	0.97	0.02	0.99	0.93	1.02
752	Ar1	0.2	1.06	0.12	1.03	0.07	1.08	0.88	1.01
753	Ar1	0.13	1.01	0.06	1	0.04	1.03	0.95	1.02
754	Ar1	0.18	1.06	0.1	0.99	0.08	1.06	0.8	0.98
755	Ar1	0.1	1.66	0.03	0.82	0.02	0.84	0.8	0.9

756	Ar1	0.18	1.64	0.09	0.82	0.02	0.86	0.8	1.16
757	Ar1	0.1	1.62	0.05	0.82	0.03	0.86	0.78	1.09
758	Ar1	0.2	0.97	0.17	0.89	0.11	0.99	0.65	0.96
759	Ar1	0.12	0.9	0.07	0.87	0.06	0.93	0.75	0.93
760	Ar1	0.15	0.88	0.07	0.85	0.07	0.93	0.69	0.97
761	Ar1	0.46	2.05	0.23	0.91	0.03	0.99	0.86	1.65
762	Ar1	0.31	1.96	0.2	0.9	0.03	0.97	0.86	1.53
763	Ar1	0.31	1.74	0.21	0.91	0.04	0.95	0.82	1.09
764	Ar1	0.08	1.38	0.06	0.98	0.03	1.01	0.95	1.06
765	Ar1	0.09	0.95	0.02	0.95	0	0.95	0.95	0.99
766	Ar1	0.47	1.08	0.16	0.65	0.04	0.69	0.52	0.76
767	Ar1	0.12	1.21	0.04	0.63	0.03	0.67	0.58	0.95
768	Ar1	0.15	1.49	0.04	0.74	0.03	0.78	0.69	0.92
769	Ar1	0.12	0.75	0.07	0.68	0.14	0.82	0.45	0.66
770	Ar1	0.08	0.75	0.05	0.77	0.01	0.78	0.75	0.76
771	Ar1	0.43	0.86	0.19	0.85	0.05	0.9	0.71	0.69
772	Ar1	0.53	0.78	0.14	0.78	0.06	0.86	0.6	0.81
773	Ar1	0.39	0.86	0.2	0.78	0.07	0.86	0.62	0.77
774	Ar1	0.08	0.84	0.03	0.84	0.01	0.86	0.84	0.83
775	Ar1	0.11	0.65	0.06	0.67	0.05	0.75	0.6	0.65
776	Chuff	0.39	0.26	0.08	0.33	0.3	1.51	0.13	0.24
777	Chuff	0.39	0.26	0.11	0.35	0.33	1.62	0.11	0.24
778	Ar1	0.83	1.03	0.66	1.04	0.09	1.18	0.62	1.33
779	Ar1	0.78	1.51	0.6	0.92	0.07	1.01	0.73	1.23
780	Ar1	0.65	1.4	0.6	0.88	0.08	1.01	0.65	1.4
781	Ar1	0.55	0.95	0.4	0.89	0.06	0.99	0.78	0.88
782	Ar1	0.61	0.95	0.33	0.92	0.04	0.99	0.75	0.91
783	Ar1	0.94	0.95	0.59	0.89	0.07	0.97	0.71	1
784	Ar1	1	0.95	0.27	0.91	0.05	0.99	0.75	0.92
785	Ar1	0.75	1.01	0.23	0.89	0.16	1.06	0.43	0.84
786	Ar1	0.5	1.14	0.18	1.16	0.12	1.44	0.93	1.15
787	Ar1	0.36	1.34	0.17	1.28	0.06	1.4	1.18	1.38
788	Ar1	0.27	1.62	0.02	1.22	0.06	1.29	1.08	1.55
789	Ar1	0.28	1.31	0.16	1.25	0.11	1.36	0.99	1.34
790	Ar1	0.08	1.53	0.04	1.53	0.01	1.55	1.53	1.51
791	Ar1	0.53	1.49	0.39	1.54	0.07	1.62	1.31	1.52
792	Ar1	0.6	1.62	0.18	1.41	0.16	1.59	0.93	1.39
793	Ar1	0.44	1.57	0.17	1.07	0.1	1.14	0.8	1.48
794	Ar1	0.36	1.79	0.1	1.05	0.06	1.1	0.93	1.45
795	Ar1	0.29	1.85	0.09	1.03	0.05	1.14	0.95	1.66
796	Ar1	0.29	1.83	0.11	0.96	0.03	1.06	0.9	1.63
797	Ar1	0.24	1.94	0.11	1.02	0.03	1.08	0.93	1.62
798	Ar1	0.26	1.79	0.11	1	0.04	1.08	0.9	1.59
799	Ar1	0.28	1.92	0.17	1	0.05	1.08	0.9	1.35
800	Ar1	0.15	1.98	0.06	1.01	0.1	1.12	0.82	1.31
801	Ar1	0.07	2.05	0.04	1.01	0.01	1.03	0.99	2.05
802	Ar1	0.16	2.15	0.04	1.11	0.01	1.12	1.1	1.19
803	Ar1	0.12	1.64	0.05	1.1	0.03	1.14	1.06	1.52
804	Ar1	0.11	2.13	0.04	1.05	0.05	1.1	0.97	1.68
805	Ar1	0.1	2.17	0.04	1.09	0.04	1.14	1.01	1.2
806	Ar1	0.13	2.07	0.06	1.1	0.05	1.14	1.01	1.46
807	Ar1	0.18	2.11	0.04	1.08	0.02	1.12	1.06	1.54
808	Ar1	0.06	2.13	0.03	1.07	0.01	1.08	1.06	1.09
809	Ar1	0.06	2.22	0.05	1.1	0	1.1	1.1	1.14

810	Ar1	0.08	2.28	0.03	1.15	0.01	1.16	1.14	1.61
811	Ar1	0.13	2.26	0.06	1.13	0.01	1.14	1.12	1.13
812	Ar1	0.1	2.17	0.04	1.08	0.02	1.1	1.03	1.84
813	Ar1	0.17	2.17	0.07	1.09	0.01	1.12	1.08	1.43
814	Ar1	2.04	2.3	1.74	0.81	0.07	0.93	0.6	1.84
815	Ar1	2.39	2.54	1.23	0.76	0.05	0.82	0.65	1.97
816	Ar1	2.53	1.72	2.39	1.05	0.09	1.21	0.78	1.55
817	Ar1	1.51	1.96	1.35	1.15	0.09	1.34	0.9	1.67
818	Ar1	1.8	0.82	1.46	0.91	0.11	1.12	0.75	0.95
819	Ar1	1.66	1.23	1.36	0.7	0.24	1.08	0.32	1.17
820	Ar1	1.17	1.55	0.91	0.95	0.11	1.16	0.71	1.31
821	Ar1	0.97	1.72	0.84	1.04	0.17	1.21	0.45	1.36
822	Ar1	1.11	1.53	1.03	1.03	0.13	1.16	0.56	1.38
823	Ar1	1.51	1.92	0.76	0.91	0.09	1.03	0.62	1.41
824	Ar1	1.52	1.44	0.62	0.96	0.11	1.1	0.39	1.19
825	Ar1	1.53	1.14	0.47	0.95	0.16	1.14	0.32	1.15
826	Ar1	1.04	2.56	0.57	0.87	0.18	1.01	0.37	1.61
827	Ar1	1.07	1.89	0.74	0.88	0.2	1.06	0.24	1.71
828	Ar1	0.53	1.98	0.4	1.07	0.16	1.31	0.73	2.02
886	Ar1	0.45	1.98	0.06	0.61	0.12	0.73	0.43	1.59
890	Ar1	0.49	2.5	0.3	0.65	0.14	0.86	0.45	1.01
831	Ar1	0.71	2.45	0.41	1.03	0.11	1.18	0.71	1.25
832	Ar1	0.52	0.71	0.47	1.04	0.2	1.27	0.69	1.07
833	Ar1	0.61	2.11	0.47	1.12	0.11	1.23	0.82	1.5
834	Ar1	0.42	1.79	0.3	0.55	0.21	0.9	0.22	0.94
835	Ar1	0.75	1.77	0.27	0.68	0.13	0.8	0.32	1.14
836	Ar1	0.75	0.82	0.46	0.71	0.17	0.93	0.28	0.75
837	Ar1	0.51	1.1	0.33	0.65	0.13	0.82	0.43	1.07
838	Ar1	0.64	1.77	0.39	0.69	0.14	0.84	0.34	1.32
839	Ar1	0.57	1.55	0.43	0.76	0.11	0.86	0.37	1.04
840	Ar1	0.65	1.68	0.38	0.69	0.18	0.86	0.32	0.71
841	Ar1	1.5	0.86	0.47	0.6	0.19	0.9	0.3	0.68
842	Ar1	0.8	1.23	0.65	0.77	0.16	0.97	0.34	1.34
843	Ar1	0.93	1.25	0.79	0.79	0.13	0.95	0.41	1.09
844	Ar1	0.42	1.68	0.18	0.77	0.16	0.88	0.34	1.08
845	Ar1	0.37	1.72	0.2	0.81	0.12	0.88	0.39	1.52
891	Ar1	0.42	2.17	0.24	0.62	0.12	0.73	0.43	1.64
847	Ar1	0.69	1.81	0.3	0.81	0.05	0.88	0.65	1.52
848	Ar1	0.5	1.81	0.31	0.86	0.15	0.95	0.34	1.48
849	Ar1	0.41	1.77	0.27	0.77	0.19	0.95	0.34	1.02
850	Ar1	0.45	1.83	0.23	0.82	0.14	0.95	0.34	1.56
851	Ar1	0.63	1.21	0.47	0.83	0.12	0.95	0.39	1.19
852	Ar1	0.71	1.74	0.35	0.82	0.09	0.93	0.47	1.45
853	Ar1	0.56	1.38	0.42	0.76	0.15	0.9	0.37	1.18
854	Ar1	1.15	1.79	0.25	0.88	0.06	0.95	0.65	1.17
855	Ar1	0.56	1.81	0.33	0.88	0.04	0.93	0.75	1.4
856	Ar1	0.45	0.84	0.31	0.86	0.15	0.99	0.34	0.86
857	Ar1	0.36	1.57	0.21	0.81	0.04	0.88	0.69	1.49
858	Ar1	0.36	1.94	0.18	0.85	0.15	0.99	0.41	1.61
859	Ar1	0.49	2.09	0.2	0.9	0.12	1.01	0.52	1.61
860	Ar1	0.46	2.02	0.2	0.85	0.19	1.06	0.39	1.58
861	Ar1	0.4	1.77	0.16	0.77	0.16	0.99	0.39	1.49
862	Ar1	0.35	2.05	0.1	0.76	0.17	0.9	0.34	1.56
863	Ar1	0.93	2.07	0.44	0.95	0.17	1.08	0.37	1.17

864	Ar1	0.87	1.72	0.33	0.86	0.17	1.03	0.39	1.34
865	Ar1	0.77	1.44	0.56	0.97	0.12	1.08	0.37	1.18
866	Ar1	0.76	1.57	0.56	0.94	0.13	1.1	0.39	1.34
867	Ar1	0.63	2	0.31	0.83	0.15	0.99	0.39	1.51
868	Ar1	0.66	1.98	0.27	0.83	0.15	0.95	0.34	1.57
869	Ar1	0.7	1.87	0.23	0.74	0.17	0.9	0.37	1.43
870	Ar1	0.63	1.51	0.44	0.71	0.27	0.99	0.17	1.43
871	Ar1	0.62	1.74	0.46	0.72	0.23	0.93	0.19	1.34
872	Ar1	0.55	1.96	0.35	0.6	0.25	0.99	0.26	1.3
873	Ar1	0.69	1.55	0.42	0.8	0.17	0.95	0.37	1.44
892	Ar1	0.37	2.3	0.24	0.67	0.09	0.8	0.45	1.39
895	Ar1	1.22	2.02	0.43	0.66	0.07	0.82	0.47	1.94
897	Ar1	1.03	2	0.33	0.58	0.08	0.78	0.43	1.39
877	Ar1	0.39	1.68	0.2	0.54	0.12	0.69	0.32	1.43
898	Ar1	0.75	2.07	0.4	0.58	0.17	0.73	0.24	1.89
879	Ar1	0.44	2.09	0.17	0.58	0.07	0.67	0.45	1.73
899	Ar1	0.87	2.13	0.21	0.57	0.18	0.75	0.24	1.89
900	Ar1	0.64	2.2	0.46	0.57	0.12	0.8	0.26	1.91
901	Ar1	0.82	2.15	0.57	0.59	0.1	0.73	0.39	1.96
902	Ar1	0.59	2.39	0.3	0.63	0.11	0.78	0.43	1.75
903	Ar1	0.51	2.28	0.29	0.68	0.1	0.78	0.37	1.87
904	Ar1	0.74	2.17	0.16	0.67	0.11	0.8	0.43	1.95
905	Ar1	0.52	2.41	0.23	0.63	0.12	0.78	0.43	1.87
887	Ar1	0.3	1.62	0.14	0.62	0.15	0.8	0.3	1.22
888	Ar1	0.38	1.96	0.08	0.63	0.07	0.73	0.47	1.76
889	Ar1	0.32	1.72	0.1	0.65	0.14	0.78	0.37	1.11
906	Ar1	0.45	2.33	0.21	0.62	0.16	0.84	0.3	2.01
907	Ar1	0.48	1.64	0.11	0.45	0.2	0.78	0.22	1.74
908	Ar1	0.75	2.78	0.14	0.55	0.11	0.82	0.34	2.19
893	Ar1	1.19	1.85	0.28	0.81	0.13	0.93	0.24	1.32
894	Ar1	0.89	2.26	0.42	0.7	0.12	0.86	0.37	1.68
909	Ar1	0.48	2.33	0.26	0.59	0.13	0.9	0.32	1.93
896	Ar1	0.68	2.07	0.39	0.59	0.14	0.78	0.26	1.68
910	Ar1	0.42	2.09	0.24	0.62	0.08	0.71	0.43	2.08
911	Ar1	0.51	2.52	0.31	0.59	0.09	0.71	0.34	2.12
912	Ar1	0.59	2.11	0.3	0.65	0.09	0.75	0.37	1.98
925	Ar1	0.37	0.45	0.34	0.65	0.12	0.8	0.43	2.72
926	Ar1	0.45	3.55	0.28	0.62	0.08	0.75	0.47	2.37
928	Ar1	0.62	2.39	0.36	0.63	0.12	0.8	0.39	2.48
929	Ar1	0.5	2.37	0.44	0.64	0.15	0.8	0.32	2.35
930	Ar1	0.58	2.5	0.28	0.68	0.21	0.93	0.28	2.55
931	Ar1	0.64	2.5	0.5	0.7	0.12	0.93	0.43	2.24
932	Ar1	0.64	2.5	0.4	0.69	0.17	0.86	0.28	2.38
933	Ar1	0.89	2.45	0.54	0.78	0.19	1.03	0.37	2.55
934	Ar1	0.89	2.45	0.5	0.85	0.1	0.99	0.52	2.39
935	Ar1	0.64	2.86	0.47	0.75	0.24	0.99	0.19	2.09
936	Ar1	0.78	2.45	0.34	0.81	0.11	0.95	0.58	2.49
937	Ar1	0.6	2.99	0.39	0.68	0.33	1.08	0.15	1.7
938	Ar1	0.62	2.67	0.42	0.78	0.21	1.06	0.28	2.27
913	Ar1	0.49	2.41	0.27	1.61	0.14	2.11	1.36	2.26
914	Ar1	0.66	1.89	0.63	1.73	0.14	1.89	1.16	1.76
915	Ar1	0.93	2.37	0.35	1.53	0.14	1.77	0.9	2.62
916	Ar1	0.62	2.07	0.11	1.54	0.21	1.74	0.95	2.14
917	Ar1	0.67	0.93	0.65	1.48	0.16	1.7	0.88	2.33

918	Ar1	1.12	2.78	1.07	1.68	0.22	1.87	0.52	3.37
919	Ar1	0.35	5.23	0.12	1.31	0.29	1.55	0.34	3.34
920	Ar1	0.71	3.68	0.48	0.66	0.06	0.82	0.54	2.34
921	Ar1	0.57	3.66	0.4	0.66	0.06	0.78	0.58	2.4
922	Ar1	0.67	3.64	0.41	0.64	0.09	0.8	0.45	2.36
923	Ar1	0.47	0.45	0.42	0.77	0.2	0.95	0.3	1.94
924	Ar1	0.55	2.45	0.42	0.65	0.12	0.88	0.47	1.88
939	Ar1	0.59	2.54	0.37	0.61	0.2	0.93	0.3	2.05
940	Ar1	0.56	1.94	0.25	0.65	0.28	0.97	0.19	1.74
927	Ar1	0.57	3.83	0.3	0.64	0.09	0.78	0.39	2.35
941	Ar1	0.38	2.02	0.35	0.75	0.21	1.01	0.37	1.81
942	Ar1	0.44	2.09	0.22	0.86	0.17	1.06	0.37	1.79
944	Ar1	0.97	2.41	0.91	0.84	0.13	1.01	0.52	2.71
945	Ar1	0.89	2.22	0.79	0.68	0.2	1.36	0.43	2.21
947	Ar1	0.54	2.48	0.22	0.81	0.19	1.31	0.34	2.56
948	Ar1	0.71	2.24	0.41	0.81	0.29	1.12	0.22	2.02
949	Ar1	0.7	2.58	0.38	0.75	0.21	1.06	0.37	2.45
950	Ar1	0.57	2.22	0.5	0.85	0.18	1.06	0.32	2.01
951	Ar1	0.9	2.69	0.38	0.73	0.22	0.99	0.22	2.7
953	Ar1	0.73	3.14	0.22	0.92	0.24	1.16	0.37	2.57
954	Ar1	1.07	2.05	0.16	0.95	0.25	1.25	0.22	2.45
955	Ar1	0.78	2.39	0.43	0.97	0.27	1.29	0.22	2.46
956	Ar1	0.7	3.57	0.46	0.77	0.28	1.03	0.19	2.09
957	Ar1	0.56	2.35	0.49	0.72	0.26	1.03	0.19	2.61
958	Ar1	0.58	2.35	0.46	0.76	0.14	1.06	0.47	3.2
943	Ar1	1.07	2.3	0.46	0.67	0.35	1.12	0.13	1.82
959	Ar1	0.67	2.5	0.6	0.84	0.14	1.06	0.45	2.95
960	Ar1	0.75	2.69	0.27	0.93	0.17	1.12	0.34	2.45
946	Ar1	0.45	2.91	0.23	0.81	0.26	1.1	0.26	1.81
961	Ar1	0.51	2.71	0.34	0.83	0.21	1.1	0.43	2.42
963	Ar1	0.4	4.2	0.25	0.79	0.07	0.88	0.65	3.1
964	Ar1	0.68	5.86	0.47	0.95	0.26	1.21	0.24	3.46
965	Ar1	0.62	5.25	0.16	0.92	0.19	1.08	0.26	3.89
966	Ar1	0.71	5.56	0.45	0.88	0.22	1.12	0.3	3.05
952	Ar1	0.49	2.41	0.39	1.04	0.17	1.23	0.56	2.71
967	Ar1	1.72	2.99	1.63	0.93	0.12	1.12	0.6	3.28
968	Ar1	1.38	4.93	0.45	0.78	0.28	1.1	0.17	3.25
969	Ar1	1.2	2.3	0.74	0.67	0.26	1.1	0.17	3.07
970	Ar1	0.82	3.06	0.65	0.86	0.29	1.16	0.22	2.79
971	Ar1	0.85	5.02	0.58	0.71	0.3	1.12	0.17	2.18
973	Ar1	0.55	8.01	0.24	0.51	0.18	0.71	0.22	2.26
974	Ar1	0.66	6.03	0.25	0.5	0.17	0.67	0.15	2.22
975	Ar1	1.38	6.72	0.44	0.57	0.1	0.67	0.24	3.04
976	Ar1	1.49	2.65	0.44	0.55	0.1	0.69	0.22	3.33
962	Ar1	0.42	2.45	0.36	0.78	0.1	1.06	0.62	2.52
977	Ar1	0.89	6.7	0.18	0.55	0.09	0.67	0.26	2.88
978	Ar1	1.21	6.91	0.27	0.57	0.08	0.69	0.3	2.2
979	Ar1	0.84	6.44	0.31	0.54	0.13	0.65	0.22	2.36
980	Ar1	1.22	6.65	0.31	0.5	0.11	0.58	0.06	3.18
981	Ar1	1.01	6.96	0.45	0.53	0.12	0.65	0.22	2.82
982	Ar1	1.34	7.11	0.38	0.53	0.09	0.65	0.19	3.54
983	Ar1	1.17	6.7	0.28	0.55	0.15	0.67	0.15	3.38
984	Ar1	1.19	6.22	0.33	0.49	0.07	0.56	0.24	3.62
985	Ar1	1.4	7.08	0.26	0.56	0.12	0.67	0.15	3.07

972	Ar1	1.29	2.84	0.32	0.92	0.14	1.1	0.45	2.84
986	Ar1	0.2	2.41	0.15	0.26	0.04	0.32	0.15	3.12
988	Ar1	1.38	1.81	0.07	0.57	0.09	0.67	0.26	2.45
989	Ar1	0.22	4.93	0.1	0.29	0.04	0.39	0.19	4.31
990	Ar1	0.8	2.5	0.49	0.57	0.14	0.84	0.28	2.55
991	Ar1	0.78	2.41	0.72	0.68	0.11	0.82	0.41	2.56
993	Ar1	0.63	2.58	0.48	0.66	0.17	1.01	0.24	2.48
996	Ar1	0.85	2.43	0.56	0.47	0.13	0.65	0.19	2.2
997	Ar1	1.02	2.5	0.48	0.55	0.09	0.65	0.26	2.76
999	Ar1	1.67	2.71	0.31	0.48	0.14	0.82	0.22	2.25
1000	Ar1	0.99	2.58	0.54	0.56	0.17	0.73	0.17	2.41
1002	Ar1	0.83	2.54	0.59	0.59	0.12	0.75	0.3	2.25
1003	Ar1	0.82	2.48	0.48	0.53	0.15	0.75	0.22	2.41
1004	Ar1	1.23	5.1	0.71	0.54	0.07	0.67	0.34	2.59
1005	Ar1	1.32	2.15	0.87	0.52	0.09	0.58	0.22	2.05
987	Ar1	0.52	4.57	0.33	0.44	0.08	0.54	0.3	2.76
1006	Ar1	1.33	2.52	0.6	0.51	0.11	0.6	0.19	2.36
1007	Ar1	0.98	2.15	0.8	0.49	0.17	0.62	0.17	1.79
1008	Ar1	1.32	2.37	0.71	0.42	0.06	0.52	0.32	2.35
1009	Ar1	1.04	2.63	0.8	0.5	0.11	0.62	0.19	2.29
992	Ar1	0.97	2.09	0.09	0.75	0.12	0.9	0.3	2.56
1010	Ar1	1.41	2.3	0.56	0.53	0.09	0.65	0.22	2.28
994	Ar1	0.63	2.39	0.47	0.77	0.09	0.86	0.34	2.69
995	Ar1	0.61	3.01	0.37	0.7	0.11	0.86	0.28	3.05
1011	Ar1	1.62	2.58	0.43	0.59	0.08	0.71	0.22	2.67
1012	Ar1	1.24	2.37	0.31	0.42	0.12	0.58	0.15	2.27
1013	Ar1	1.1	2.69	0.39	0.55	0.1	0.62	0.24	2.4
1014	Ar1	1.1	2.37	0.55	0.41	0.12	0.6	0.22	2.34
1015	Ar1	0.88	2.41	0.35	0.54	0.1	0.69	0.28	2.27
1016	Ar1	1.12	2.54	0.26	0.52	0.13	0.65	0.22	2.06
1017	Ar1	0.64	2	0.45	0.4	0.1	0.58	0.17	2.16
1018	Ar1	0.64	2.67	0.32	0.45	0.17	0.6	0.06	2.14
1019	Ar1	0.82	2.2	0.56	0.51	0.05	0.56	0.34	2.27
1020	Ar1	1.4	2.39	0.34	0.5	0.09	0.62	0.22	2.1
1021	Ar1	0.45	2.37	0.08	0.44	0.16	0.6	0.09	2.24
1022	Ar1	0.93	1.98	0.06	0.51	0.1	0.6	0.22	1.81
1023	Ar1	1.28	2.2	0.17	0.57	0.07	0.65	0.26	2.18
1024	Ar1	1.01	2.5	0.26	0.51	0.12	0.62	0.22	2.12
1026	Ar1	0.56	4.8	0.31	0.45	0.08	0.56	0.26	2.03
1027	Ar1	0.47	2.58	0.4	0.48	0.12	0.6	0.22	2.44
1028	Ar1	1.56	1.92	1.08	0.34	0.06	0.45	0.22	2.81
1029	Ar1	1.1	2.28	0.74	0.27	0.05	0.39	0.17	2.5
1031	Ar1	1.35	2	0.05	0.49	0.07	0.56	0.24	2.44
1032	Ar1	1.5	2.95	0.27	0.53	0.1	0.62	0.22	2.41
1033	Ar1	1.23	2.56	0.2	0.49	0.1	0.56	0.17	2.15
1034	Ar1	1.12	1.83	0.23	0.43	0.09	0.52	0.24	1.94
1036	Ar1	0.23	2.3	0.1	0.35	0.07	0.45	0.24	2.75
1037	Ar1	0.93	2.67	0.59	0.67	0.21	0.95	0.28	2.41
1038	Ar1	0.94	2.37	0.74	0.69	0.19	0.9	0.26	2.41
1039	Ar1	1.69	2.58	1.17	0.77	0.13	0.99	0.3	2.55
1040	Ar1	1.16	2.48	0.96	0.78	0.1	0.93	0.52	2.46
1041	Ar1	2.02	2.52	0.39	0.7	0.14	0.93	0.22	2.55
1042	Ar1	1.17	2.37	0.94	0.77	0.11	0.9	0.37	2.38
1025	Ar1	0.56	4.31	0.18	0.41	0.07	0.52	0.26	2.57

1043	Ar1	1.08	2.35	0.65	0.55	0.17	0.71	0.17	1.98
1044	Ar1	1.01	2.35	0.6	0.53	0.16	0.75	0.19	1.9
1045	Ar1	1.62	2.39	0.51	0.61	0.13	0.78	0.22	2.26
1047	Ar1	0.98	2.71	0.76	0.6	0.17	0.73	0.15	2.38
1030	Ar1	1.2	5.06	0.47	0.23	0.03	0.3	0.15	2.57
1048	Ar1	1.01	2.52	0.82	0.53	0.18	0.71	0.15	1.78
1049	Ar1	0.9	2.52	0.7	0.53	0.18	0.69	0.15	2.21
1051	Ar1	2.21	2.58	0.56	0.7	0.14	0.93	0.22	2.56
1052	Ar1	1.51	2.52	0.99	0.65	0.13	0.82	0.28	2.13
1035	Ar1	0.5	2.5	0.22	0.42	0.09	0.58	0.26	2.3
1053	Ar1	1.24	2.24	1	0.57	0.12	0.73	0.24	2.06
1054	Ar1	1.54	2.39	0.86	0.54	0.1	0.69	0.22	2.2
1055	Ar1	1.28	2.41	1.14	0.56	0.1	0.65	0.13	2.15
1056	Ar1	1.32	2.35	1.13	0.58	0.11	0.73	0.22	2.24
1057	Ar1	1.22	2.56	0.29	0.5	0.08	0.65	0.3	2.46
1058	Ar1	0.93	2.45	0.41	0.5	0.13	0.73	0.17	2.08
1065	Ar1	1.03	2.3	0.5	0.49	0.11	0.6	0.24	2.15
1066	Ar1	1.07	2.89	0.64	0.54	0.1	0.65	0.19	2.33
1067	Ar1	1.06	2.56	0.54	0.55	0.15	0.69	0.15	2.06
1068	Ar1	0.91	2.69	0.62	0.47	0.12	0.71	0.19	2.35
1069	Ar1	1.5	2.69	0.33	0.58	0.09	0.69	0.24	2.19
1070	Ar1	0.84	2.22	0.52	0.52	0.18	0.88	0.22	2.1
1071	Ar1	1.4	2.39	0.2	0.56	0.1	0.8	0.43	2.3
1072	Ar1	0.89	2.35	0.64	0.64	0.11	0.84	0.37	2.25
1050	Ar1	1.18	1.77	1.16	0.66	0.16	0.84	0.22	2.46
1076	Ar1	0.85	2.67	0.47	0.52	0.19	0.73	0.15	1.88
1077	Ar1	1.21	2.56	0.2	0.58	0.12	0.69	0.22	2.4
1079	Ar1	0.96	2.41	0.25	0.5	0.1	0.67	0.34	2
1080	Ar1	0.72	2.54	0.27	0.58	0.11	0.73	0.26	2.43
1081	Ar1	0.76	2.43	0.51	0.57	0.08	0.69	0.39	2.16
1084	Ar1	0.73	2.63	0.36	0.53	0.12	0.73	0.26	2.58
1085	Ar1	0.83	2.35	0.27	0.56	0.16	0.78	0.19	2.25
1088	Ar1	0.51	2.3	0.41	0.53	0.09	0.6	0.28	1.55
1092	Ar1	0.54	4.07	0.37	0.54	0.07	0.6	0.22	1.52
1093	Ar1	0.65	2.26	0.25	0.4	0.13	0.67	0.19	2.08
1096	Ar1	0.73	2.26	0.53	0.43	0.11	0.6	0.26	1.48
1097	Ar1	0.88	2.28	0.37	0.45	0.12	0.58	0.19	1.95
1098	Ar1	1.03	2.2	0.19	0.52	0.13	0.62	0.15	1.88
1101	Ar1	0.83	1.94	0.25	0.57	0.09	0.73	0.39	2.12
1102	Ar1	0.82	2.54	0.23	0.55	0.14	0.82	0.22	2.38
1103	Ar1	0.5	2.26	0.37	0.54	0.16	0.73	0.24	2.27
1106	Ar1	0.67	3.64	0.17	0.4	0.17	0.67	0.19	2.24
1109	Ar1	0.69	2.41	0.15	0.53	0.08	0.71	0.3	2.24
1110	Ar1	0.59	2.41	0.17	0.55	0.11	0.71	0.24	2.22
1112	Ar1	0.54	2.22	0.21	0.56	0.16	0.75	0.26	2.08
1114	Ar1	0.48	2.61	0.28	0.53	0.16	0.71	0.24	2.36
1115	Ar1	0.76	2.07	0.33	0.55	0.14	0.73	0.22	1.71
1116	Ar1	0.83	2.45	0.26	0.55	0.11	0.8	0.28	1.92
1117	Ar1	0.57	2.58	0.33	0.55	0.12	0.69	0.26	1.76
1118	Ar1	0.64	2.05	0.3	0.48	0.17	0.69	0.22	1.8
1119	Ar1	0.98	2.37	0.18	0.54	0.15	0.69	0.19	1.75
1120	Ar1	0.65	2.22	0.44	0.47	0.13	0.6	0.19	1.6
1121	Ar1	0.64	2.15	0.39	0.38	0.14	0.67	0.19	1.51
1122	Ar1	0.82	2.35	0.4	0.51	0.08	0.62	0.32	1.8

1123	Ar1	0.82	2.17	0.34	0.43	0.12	0.62	0.26	1.65
1125	Ar1	0.48	2.33	0.29	0.53	0.07	0.65	0.37	2.03
1126	Ar1	0.77	1.74	0.34	0.51	0.13	0.67	0.22	1.81
1127	Ar1	0.79	2.2	0.34	0.53	0.1	0.71	0.3	1.77
1128	Ar1	0.8	1.83	0.38	0.54	0.08	0.62	0.39	1.76
1129	Ar1	0.45	1.77	0.32	0.43	0.15	0.78	0.24	1.37
1086	Ar1	0.55	2.5	0.31	0.39	0.09	0.54	0.24	2.1
1130	Ar1	0.69	2.24	0.33	0.55	0.1	0.71	0.24	1.65
1131	Ar1	0.56	2	0.41	0.59	0.09	0.69	0.3	1.18
1089	Ar1	0.6	2.11	0.29	0.5	0.07	0.58	0.3	1.88
1090	Ar1	0.59	3.01	0.24	0.46	0.11	0.6	0.26	2.15
1091	Ar1	0.6	2.37	0.28	0.48	0.11	0.67	0.26	1.99
1132	Ar1	0.73	1.81	0.33	0.55	0.06	0.65	0.37	1.78
1133	Ar1	0.79	2.24	0.36	0.46	0.07	0.6	0.32	2.04
1135	Ar1	0.72	2.33	0.3	0.45	0.12	0.6	0.22	1.79
1136	Ar1	0.79	1.96	0.32	0.43	0.15	0.65	0.17	1.55
1142	Ar1	0.72	2.02	0.37	0.52	0.04	0.58	0.41	1.76
1143	Ar1	0.5	2.26	0.34	0.4	0.1	0.6	0.26	1.93
1145	Ar1	0.37	2.43	0.25	0.47	0.1	0.56	0.22	1.65
1146	Ar1	0.35	2.07	0.29	0.48	0.09	0.6	0.3	1.32
1147	Ar1	0.52	0.26	0.49	0.43	0.14	0.58	0.22	1.39
1148	Ar1	0.7	1.66	0.33	0.49	0.11	0.6	0.22	1.51
1149	Ar1	0.97	1.72	0.07	0.43	0.13	0.58	0.22	1.47
1150	Ar1	0.71	2.15	0.19	0.47	0.07	0.56	0.26	2
1104	Ar1	0.56	2.45	0.27	0.51	0.11	0.69	0.3	2.38
1151	Ar1	0.65	2.43	0.16	0.46	0.14	0.67	0.19	1.28
1152	Ar1	0.4	2.35	0.19	0.51	0.1	0.62	0.28	1.9
1107	Ar1	0.49	2.11	0.18	0.48	0.17	0.71	0.19	2
1108	Ar1	0.69	2.28	0.17	0.53	0.11	0.67	0.26	2.13
1153	Ar1	0.37	2.26	0.2	0.52	0.04	0.56	0.43	1.89
1154	Ar1	0.57	2.11	0.1	0.47	0.1	0.62	0.3	1.72
1111	Ar1	0.46	2.39	0.13	0.49	0.17	0.78	0.19	2.62
1155	Ar1	0.54	2	0.06	0.61	0.08	0.62	0.24	1.76
1113	Ar1	0.72	2.22	0.08	0.62	0.15	0.73	0.22	2.02
1156	Ar1	0.33	2.39	0.19	0.55	0.05	0.6	0.45	1.72
1157	Ar1	0.39	2.35	0.18	0.51	0.1	0.73	0.32	1.11
1159	Ar1	0.77	2.22	0.116816	0.42	0.05	0.5	0.34	2.05
1164	Ar1	0.35	2.61	0.16	0.44	0.1	0.54	0.26	1.95
1168	Ar1	0.59	2.48	0.14	0.49	0.07	0.6	0.37	2.12
1170	Ar1	0.35	2.45	0.16	0.47	0.08	0.6	0.34	2.13
1171	Ar1	0.68	1.77	0.09	0.5	0.14	0.62	0.22	1.46
1176	Ar1	0.48	2.05	0.2	0.53	0.08	0.67	0.28	1.63
1177	Ar1	0.35	2.24	0.18	0.48	0.08	0.56	0.32	2.04
1178	Ar1	0.72	2.33	0.13	0.45	0.15	0.65	0.19	1.77
1124	Ar1	0.57	2.17	0.3	0.54	0.05	0.65	0.47	2.01
1180	Ar1	0.59	2.45	0.15	0.5	0.09	0.69	0.26	2.08
1181	Ar1	0.5	2.43	0.12	0.5	0.12	0.65	0.22	1.95
1182	Ar1	0.24	2.02	0.07	0.34	0.06	0.45	0.22	1.61
1183	Ar1	0.2	2.13	0.08	0.36	0.04	0.43	0.3	1.75
1185	Ar1	0.47	2	0.1	0.36	0.07	0.43	0.22	1.69
1190	Ar1	0.28	2.45	0.06	0.36	0.05	0.5	0.3	1.17
1192	Ar1	0.47	2.35	0.07	0.32	0.02	0.37	0.26	2.14
1194	Ar1	0.79	1.49	0.18	0.35	0.11	0.47	0.15	1.5
1195	Ar1	0.64	1.79	0.26	0.36	0.06	0.47	0.26	1.38

1134	Ar1	0.63	1.94	0.41	0.49	0.11	0.6	0.28	1.73
1196	Ar1	0.6	2.17	0.19	0.38	0.08	0.45	0.22	1.32
1197	Ar1	0.63	2.15	0.16	0.37	0.08	0.45	0.17	1.7
1137	Ar1	0.71	2	0.49	0.46	0.1	0.58	0.28	1.57
1138	Ar1	0.75	2.26	0.22	0.46	0.1	0.58	0.24	1.97
1139	Ar1	0.73	2.15	0.33	0.49	0.08	0.6	0.28	1.75
1140	Ar1	0.75	2.11	0.3	0.49	0.07	0.58	0.28	1.83
1141	Ar1	0.62	1.94	0.42	0.49	0.09	0.58	0.28	1.67
1198	Ar1	0.46	2.39	0.24	0.41	0.08	0.47	0.19	1.48
1200	Ar1	0.49	2.41	0.29	0.38	0.09	0.5	0.19	1.5
1144	Ar1	0.44	2.28	0.09	0.44	0.07	0.52	0.28	1.77
1201	Ar1	0.38	1.74	0.27	0.42	0.1	0.56	0.17	1.42
1202	Ar1	0.67	2.33	0.24	0.39	0.06	0.47	0.26	1.53
1203	Ar1	0.6	2.37	0.21	0.39	0.08	0.47	0.22	1.8
1204	Ar1	1.19	2.11	0.15	0.39	0.07	0.45	0.17	1.63
1205	Ar1	0.9	2.22	0.33	0.38	0.07	0.45	0.19	1.77
1206	Ar1	0.98	2.35	0.27	0.35	0.08	0.47	0.19	1.64
1207	Ar1	0.68	2.26	0.34	0.38	0.09	0.47	0.17	1.55
1208	Ar1	0.77	2.13	0.28	0.33	0.08	0.43	0.17	1.8
1210	Ar1	0.62	2.39	0.33	0.4	0.09	0.47	0.17	1.44
1211	Ar1	0.5	2.45	0.38	0.36	0.07	0.47	0.22	1.87
1212	Ar1	0.47	2.28	0.41	0.35	0.08	0.5	0.22	1.62
1213	Ar1	0.75	2.28	0.21	0.4	0.06	0.47	0.22	1.86
1214	Ar1	0.44	2.35	0.33	0.34	0.03	0.41	0.28	1.98
1158	Ar1	0.52	2.15	0.27	0.45	0.1	0.58	0.26	1.47
1216	Ar1	0.5	2.09	0.25	0.38	0.09	0.47	0.19	1.68
1217	Ar1	0.72	2.24	0.21	0.39	0.05	0.47	0.26	1.85
1161	Ar1	0.51	2.37	0.17	0.41	0.05	0.54	0.34	2.12
1162	Ar1	0.45	2.13	0.35	0.51	0.05	0.58	0.32	1.81
1163	Ar1	0.5	2.09	0.18	0.46	0.08	0.58	0.28	1.95
1220	Ar1	0.72	2.3	0.32	0.44	0.11	0.6	0.19	1.73
1165	Ar1	0.28	1.98	0.04	0.5	0.04	0.54	0.39	2.07
1166	Ar1	0.25	2.52	0.03	0.52	0.09	0.69	0.32	1.36
1167	Ar1	0.53	2.09	0.27	0.52	0.07	0.65	0.37	2.01
1221	Ar1	1	2.45	0.17	0.47	0.05	0.56	0.32	2.02
1169	Ar1	0.39	2.43	0.11	0.5	0.1	0.6	0.26	1.9
1222	Ar1	0.73	2.54	0.37	0.49	0.1	0.56	0.17	1.97
1223	Ar1	0.86	2.45	0.26	0.5	0.11	0.6	0.09	1.88
1172	Ar1	0.45	2.41	0.16	0.44	0.13	0.62	0.19	2.06
1173	Ar1	0.52	2.13	0.33	0.49	0.05	0.58	0.37	2.09
1174	Ar1	0.69	2.43	0.13	0.5	0.06	0.6	0.3	2.04
1175	Ar1	0.44	2.3	0.23	0.42	0.11	0.58	0.22	2.1
1224	Ar1	0.63	2.65	0.33	0.33	0.09	0.56	0.15	1.84
1226	Ar1	0.9	2.17	0.23	0.37	0.11	0.54	0.17	1.68
1227	Ar1	0.95	2.15	0.17	0.48	0.09	0.56	0.09	1.99
1179	Ar1	0.55	2.17	0.23	0.52	0.06	0.65	0.3	2.06
1229	Ar1	0.78	2.28	0.3	0.36	0.1	0.56	0.17	2.11
1230	Ar1	0.93	2.63	0.18	0.44	0.08	0.54	0.22	1.92
1232	Ar1	0.7	2.24	0.14	0.48	0.11	0.56	0.22	2.05
1233	Ar1	0.41	2.73	0.24	0.5	0.06	0.62	0.37	2
1184	Ar1	0.47	1.85	0.21	0.4	0.07	0.47	0.24	1.2
1234	Ar1	0.47	2	0.2	0.57	0.1	0.69	0.22	1.6
1186	Ar1	0.52	2.43	0.15	0.4	0.08	0.5	0.24	0.99
1187	Ar1	0.64	2.2	0.1	0.44	0.04	0.5	0.28	1.4

1188	Ar1	0.47	2.17	0.24	0.39	0.04	0.52	0.32	1.71
1189	Ar1	0.58	2.17	0.17	0.39	0.08	0.56	0.22	1.43
1240	Ar1	0.58	1.92	0.09	0.42	0.13	0.6	0.19	1.58
1247	Ar1	0.45	2.52	0.24	0.44	0.06	0.52	0.26	2.13
1249	Ar1	0.7	2.5	0.3	0.43	0.09	0.58	0.26	2.18
1193	Ar1	0.3	2.17	0.17	0.29	0.07	0.41	0.19	1.89
1251	Ar1	0.96	2.22	0.26	0.42	0.1	0.54	0.11	2.01
1252	Ar1	0.46	2.24	0.24	0.44	0.05	0.56	0.3	2.26
1257	Ar1	0.49	2.37	0.27	0.42	0.06	0.5	0.24	1.86
1259	Ar1	0.93	1.87	0.56	0.47	0.17	0.67	0.11	1.52
1260	Ar1	0.73	2.26	0.57	0.46	0.18	0.65	0.13	1.44
1199	Ar1	0.46	2.09	0.26	0.31	0.12	0.5	0.15	1.71
1261	Ar1	0.85	2.07	0.72	0.49	0.14	0.62	0.13	1.51
1262	Ar1	1.1	2.3	0.56	0.43	0.15	0.6	0.13	1.74
1263	Ar1	0.98	1.87	0.65	0.5	0.11	0.65	0.26	1.68
1264	Ar1	1.11	2.28	0.59	0.52	0.11	0.65	0.19	1.58
1265	Ar1	0.91	2.33	0.73	0.48	0.1	0.71	0.24	1.34
1268	Ar1	1.48	2.13	0.67	0.5	0.09	0.6	0.32	1.87
1269	Ar1	1.09	1.94	0.79	0.52	0.08	0.67	0.34	1.75
1270	Ar1	1.03	2.41	0.7	0.6	0.09	0.69	0.37	2.11
1271	Ar1	1.22	2.11	0.58	0.55	0.17	0.73	0.17	1.75
1272	Ar1	1.06	2.24	0.67	0.52	0.13	0.62	0.17	2.15
1273	Ar1	1.28	2.33	0.63	0.51	0.13	0.67	0.17	1.88
1274	Ar1	1.28	2.52	0.67	0.55	0.14	0.71	0.19	1.67
1275	Ar1	0.92	2.33	0.74	0.55	0.12	0.71	0.22	1.87
1276	Ar1	1.06	2.26	0.7	0.52	0.13	0.73	0.34	1.65
1277	Ar1	0.88	2.48	0.59	0.47	0.1	0.65	0.32	1.65
1215	Ar1	0.62	2.24	0.23	0.36	0.08	0.5	0.15	1.83
1278	Ar1	0.81	1.85	0.58	0.59	0.11	0.71	0.26	1.62
1279	Ar1	0.95	2.15	0.5	0.54	0.11	0.69	0.3	1.72
1280	Ar1	0.75	2.39	0.61	0.57	0.1	0.67	0.28	2.09
1281	Ar1	0.8	2.15	0.67	0.58	0.06	0.71	0.39	2.01
1282	Ar1	0.75	2.11	0.55	0.54	0.15	0.75	0.22	1.76
1283	Ar1	1.05	2.2	0.41	0.52	0.12	0.65	0.34	1.96
1284	Ar1	1.07	2.24	0.49	0.54	0.11	0.65	0.19	1.97
1285	Ar1	0.97	2.2	0.5	0.54	0.11	0.65	0.22	1.98
1286	Ar1	0.8	1.89	0.73	0.51	0.09	0.67	0.34	1.81
1288	Ar1	0.92	2.48	0.35	0.49	0.1	0.67	0.24	1.92
1289	Ar1	0.65	2.26	0.47	0.5	0.13	0.62	0.17	1.98
1290	Ar1	0.84	2.26	0.62	0.48	0.13	0.65	0.13	1.73
1228	Ar1	0.66	2.3	0.37	0.36	0.09	0.47	0.19	1.85
1292	Ar1	0.96	2.26	0.59	0.46	0.1	0.58	0.15	1.93
1294	Ar1	1.08	2.41	0.43	0.4	0.08	0.62	0.24	1.84
1231	Ar1	0.52	2.37	0.28	0.48	0.07	0.56	0.3	2.23
1295	Ar1	0.87	2.24	0.541597	0.43	0.06	0.6	0.3	1.91
1296	Ar1	1.03	2.3	0.31	0.51	0.1	0.62	0.17	2.03
1297	Ar1	0.81	2.3	0.52	0.48	0.11	0.58	0.22	1.76
1298	Ar1	1.12	2.2	0.37	0.49	0.09	0.56	0.22	2.1
1236	Ar1	0.51	2.17	0.31	0.4	0.09	0.54	0.26	2.06
1299	Ar1	0.83	2.17	0.29	0.5	0.13	0.62	0.11	1.86
1238	Ar1	0.44	1.74	0.37	0.43	0.1	0.6	0.26	1.62
1239	Ar1	0.48	1.79	0.34	0.5	0.09	0.6	0.28	1.52
1300	Ar1	0.98	2.43	0.26	0.46	0.12	0.56	0.13	1.83
1241	Ar1	0.49	2.45	0.13	0.41	0.09	0.56	0.24	1.85

1242	Ar1	0.43	2.39	0.18	0.43	0.13	0.56	0.22	1.6
1243	Ar1	0.49	2.17	0.2	0.38	0.08	0.52	0.24	1.85
1244	Ar1	0.47	2.76	0.25	0.39	0.04	0.47	0.34	1.76
1245	Ar1	0.57	2.22	0.28	0.42	0.08	0.52	0.26	1.97
1246	Ar1	0.5	2.09	0.32	0.42	0.04	0.52	0.32	2.1
1301	Ar1	0.91	2.24	0.4	0.5	0.08	0.56	0.26	2.07
1248	Ar1	0.56	2.58	0.4	0.41	0.14	0.56	0.17	1.97
1302	Ar1	0.91	2.33	0.36	0.48	0.11	0.58	0.17	1.91
1250	Ar1	0.64	2.24	0.41	0.42	0.07	0.54	0.26	1.96
1303	Ar1	0.78	2.41	0.41	0.44	0.11	0.6	0.13	1.91
1305	Ar1	1.06	2.24	0.23	0.41	0.1	0.56	0.22	1.98
1253	Ar1	0.59	2.26	0.18	0.42	0.07	0.52	0.26	1.8
1254	Ar1	0.51	2.37	0.2	0.46	0.08	0.56	0.19	1.98
1306	Ar1	0.87	2.09	0.56	0.43	0.13	0.62	0.13	1.43
1308	Ar1	0.83	2.3	0.62	0.5	0.11	0.58	0.22	1.94
1309	Ar1	0.78	2.13	0.53	0.45	0.12	0.6	0.22	1.88
1258	Ar1	0.55	1.98	0.15	0.43	0.05	0.47	0.3	2
1312	Ar1	1.01	1.79	0.53	0.49	0.13	0.62	0.19	1.66
1313	Ar1	1.3	2.35	0.66	0.46	0.13	0.6	0.15	1.77
1314	Ar1	1.18	2.07	0.59	0.45	0.16	0.65	0.11	1.96
1315	Ar1	1.02	2.26	0.65	0.52	0.11	0.62	0.22	1.87
1316	Ar1	1.02	2.43	0.67	0.49	0.12	0.65	0.17	1.94
1317	Ar1	0.99	2.02	0.66	0.44	0.14	0.65	0.19	1.43
1319	Ar1	0.98	2.33	0.77	0.54	0.15	0.73	0.17	1.82
1266	Ar1	1.64	1.46	0.92	0.51	0.16	0.75	0.15	1.14
1267	Ar1	1.04	1.62	0.9	0.52	0.09	0.62	0.3	1.18
1322	Ar1	0.89	2.33	0.52	0.53	0.14	0.69	0.15	1.92
1323	Ar1	0.72	2.5	0.49	0.47	0.15	0.62	0.15	2.06
1324	Ar1	1.23	2.95	0.25	0.51	0.1	0.6	0.22	2.07
1327	Ar1	0.82	2.24	0.46	0.49	0.1	0.6	0.28	2.11
1328	Ar1	1.26	2.17	0.24	0.5	0.16	0.71	0.04	2.1
1329	Ar1	0.9	2.02	0.62	0.48	0.15	0.62	0.15	1.78
1330	Ar1	1.06	2.52	0.39	0.44	0.12	0.62	0.13	2
1332	Ar1	0.94	2.8	0.62	0.47	0.11	0.58	0.15	2
1333	Ar1	0.6	2.78	0.25	0.57	0.13	0.73	0.26	1.95
1334	Ar1	0.52	2.41	0.37	0.58	0.12	0.71	0.34	2.02
1335	Ar1	0.66	2.58	0.38	0.58	0.09	0.69	0.39	2.07
1337	Ar1	0.47	2.65	0.28	0.6	0.11	0.71	0.3	1.93
1339	Ar1	0.57	3.34	0.22	0.59	0.07	0.71	0.43	1.54
1342	Ar1	0.61	2.39	0.34	0.57	0.12	0.73	0.34	2.07
1343	Ar1	0.63	2.22	0.28	0.57	0.15	0.73	0.3	1.8
1344	Ar1	0.99	2.43	0.37	0.48	0.19	0.78	0.09	2.27
1345	Ar1	0.73	2.39	0.57	0.53	0.17	0.69	0.24	1.52
1346	Ar1	0.75	2.41	0.51	0.6	0.07	0.71	0.34	2.03
1348	Ar1	1.05	2.45	0.18	0.58	0.13	0.73	0.24	2.34
1350	Ar1	0.55	2.41	0.42	0.44	0.18	0.67	0.17	1.77
1352	Ar1	0.57	2.67	0.29	0.47	0.1	0.62	0.26	2.31
1353	Ar1	0.64	2.43	0.39	0.52	0.08	0.67	0.28	2.17
1354	Ar1	0.68	2.76	0.2	0.47	0.09	0.62	0.32	2.27
1291	Ar1	1.68	2.28	0.2	0.43	0.06	0.58	0.3	2.23
1355	Ar1	0.69	2.3	0.17	0.46	0.13	0.67	0.24	2.16
1357	Ar1	0.66	2.43	0.29	0.52	0.1	0.69	0.26	2.09
1358	Ar1	0.63	2.13	0.39	0.53	0.06	0.62	0.41	2.17
1361	Ar1	0.8	2.41	0.33	0.47	0.12	0.71	0.24	1.84

1362	Ar1	0.81	2.33	0.23	0.43	0.15	0.67	0.17	1.82
1363	Ar1	0.63	2.35	0.45	0.42	0.14	0.6	0.24	1.35
1364	Ar1	0.74	1.53	0.44	0.43	0.17	0.69	0.17	1.61
1365	Ar1	0.91	2.33	0.31	0.45	0.14	0.58	0.15	1.8
1366	Ar1	1.17	1.38	0.14	0.53	0.17	0.75	0.15	1.55
1367	Ar1	0.99	2.58	0.19	0.48	0.12	0.67	0.24	1.97
1368	Ar1	0.79	2.22	0.4	0.49	0.1	0.65	0.26	2.07
1370	Ar1	0.93	2.39	0.33	0.45	0.12	0.71	0.22	2.26
1304	Ar1	1.13	2.11	0.22	0.4	0.07	0.56	0.26	2.33
1372	Ar1	0.7	2.33	0.46	0.51	0.11	0.65	0.22	2.15
1373	Ar1	0.74	2.05	0.53	0.5	0.11	0.69	0.26	1.66
1374	Ar1	0.83	2.33	0.38	0.5	0.09	0.62	0.24	2.16
1375	Ar1	0.66	2.41	0.38	0.44	0.14	0.6	0.17	2.11
1377	Ar1	0.58	2.43	0.23	0.5	0.19	0.73	0.19	1.78
1378	Ar1	0.49	2.3	0.26	0.54	0.19	0.78	0.17	1.8
1311	Ar1	1.48	2.2	0.27	0.46	0.07	0.58	0.3	2.15
1379	Ar1	0.54	2.39	0.31	0.58	0.14	0.69	0.28	2.05
1380	Ar1	0.54	2.39	0.09	0.56	0.13	0.67	0.26	1.96
1381	Ar1	0.52	2.56	0.14	0.53	0.14	0.65	0.24	1.99
1382	Ar1	0.77	2.2	0.27	0.61	0.12	0.73	0.28	1.93
1383	Ar1	0.73	2.45	0.22	0.55	0.11	0.71	0.32	2.06
1384	Ar1	0.55	2.39	0.19	0.54	0.15	0.71	0.22	1.94
1386	Ar1	0.49	2.28	0.24	0.47	0.17	0.67	0.15	2
1387	Ar1	0.64	2.63	0.31	0.56	0.13	0.67	0.19	1.99
1388	Ar1	0.62	2.45	0.35	0.48	0.14	0.69	0.28	1.77
1389	Ar1	0.66	2.69	0.25	0.49	0.11	0.62	0.26	2.18
1390	Ar1	0.61	2.33	0.46	0.54	0.12	0.69	0.26	1.6
1393	Ar1	0.76	2.3	0.18	0.34	0.09	0.93	0.19	1.95
1394	Ar1	0.68	2.05	0.26	0.49	0.07	0.65	0.3	1.9
1396	Ar1	0.52	2.39	0.25	0.48	0.14	0.62	0.22	1.84
1397	Ar1	0.51	2.35	0.32	0.5	0.11	0.67	0.32	1.75
1398	Ar1	0.63	2.24	0.23	0.51	0.12	0.62	0.22	2
1399	Ar1	0.62	2.5	0.23	0.49	0.14	0.62	0.19	1.68
1401	Ar1	0.85	2.48	0.56	0.6	0.07	0.67	0.39	2.17
1402	Ar1	0.87	2.22	0.55	0.55	0.13	0.8	0.26	1.7
1331	Ar1	0.79	2.54	0.69	0.38	0.14	0.62	0.13	1.93
1403	Ar1	0.82	2.5	0.4	0.55	0.14	0.75	0.22	2.02
1404	Ar1	0.91	2.02	0.45	0.55	0.13	0.73	0.28	1.76
1405	Ar1	1.01	2.15	0.46	0.57	0.12	0.73	0.3	1.9
1406	Ar1	0.69	2.45	0.51	0.51	0.17	0.67	0.19	1.86
1407	Ar1	0.67	2.17	0.5	0.48	0.16	0.78	0.28	1.76
1408	Ar1	0.9	2.24	0.27	0.56	0.15	0.8	0.15	2.16
1338	Ar1	0.44	2.48	0.18	0.54	0.14	0.78	0.26	2.02
1409	Ar1	0.63	2.26	0.49	0.53	0.14	0.67	0.28	2.11
1340	Ar1	0.82	2.11	0.1	0.62	0.12	0.88	0.3	1.83
1341	Ar1	0.47	2.37	0.21	0.51	0.16	0.75	0.22	2.21
1410	Ar1	0.76	2.43	0.28	0.55	0.1	0.78	0.26	2.34
1411	Ar1	0.76	2.54	0.34	0.49	0.16	0.65	0.22	2.04
1412	Ar1	0.76	2.22	0.67	0.58	0.19	0.78	0.17	1.83
1414	Ar1	0.92	2.28	0.67	0.56	0.14	0.86	0.26	1.93
1415	Ar1	0.71	2.67	0.52	0.57	0.16	0.84	0.26	1.93
1347	Ar1	1.11	2.22	0.23	0.52	0.11	0.67	0.28	2.2
1416	Ar1	0.83	2.63	0.42	0.59	0.12	0.88	0.28	2
1417	Ar1	0.83	2.41	0.52	0.6	0.15	0.82	0.26	1.92

1418	Ar1	0.8	2.39	0.44	0.62	0.16	0.9	0.32	2.26
1419	Ar1	0.81	2.3	0.59	0.6	0.2	0.93	0.22	1.81
1420	Ar1	0.86	2.67	0.3	0.58	0.17	0.86	0.22	2.35
1421	Ar1	0.87	2.41	0.39	0.58	0.18	0.88	0.22	2.29
1422	Ar1	0.87	2.22	0.46	0.51	0.32	1.03	0.06	1.2
1423	Ar1	0.99	2.37	0.49	0.64	0.21	0.88	0.13	1.88
1424	Ar1	0.97	2.43	0.57	0.57	0.18	0.9	0.24	1.82
1425	Ar1	0.76	2.11	0.63	0.58	0.16	0.84	0.28	1.73
1427	Ar1	0.72	2.3	0.5	0.6	0.14	0.82	0.28	2.01
1428	Ar1	0.76	2.3	0.5	0.57	0.07	0.8	0.41	2.06
1360	Ar1	0.6	2.45	0.33	0.48	0.15	0.69	0.17	2.06
1429	Ar1	0.52	1.87	0.41	0.6	0.15	0.78	0.28	1.78
1430	Ar1	0.74	2.24	0.3	0.6	0.18	0.8	0.19	1.79
1431	Ar1	0.48	2.3	0.34	0.63	0.21	0.86	0.17	1.91
1432	Ar1	0.81	2.43	0.18	0.6	0.17	0.9	0.19	2.22
1433	Ar1	0.87	2.43	0.24	0.59	0.16	0.84	0.24	2.22
1434	Ar1	0.63	2.67	0.35	0.67	0.08	0.8	0.52	2.3
1436	Ar1	0.52	2.48	0.34	0.63	0.11	0.86	0.45	2.01
1437	Ar1	0.79	1.92	0.4	0.65	0.19	0.99	0.32	1.81
1438	Ar1	1.05	2.41	0.35	0.69	0.17	0.95	0.32	2.05
1439	Ar1	0.64	1.81	0.48	0.66	0.13	0.99	0.45	1.94
1440	Ar1	0.88	2.54	0.21	0.69	0.19	0.86	0.22	2.21
1441	Ar1	0.46	0.86	0.43	0.52	0.32	1.08	0.17	1.31
1443	Ar1	0.55	2.35	0.31	0.69	0.18	0.93	0.37	2.18
1444	Ar1	0.49	2.56	0.24	0.72	0.16	0.9	0.41	2.12
1446	Ar1	0.47	2.63	0.27	0.68	0.08	0.84	0.54	2.44
1376	Ar1	0.48	2.52	0.28	0.5	0.18	0.73	0.19	1.94
1448	Ar1	0.45	2.28	0.18	0.68	0.09	0.78	0.47	2.14
1450	Ar1	0.61	2.56	0.21	0.62	0.09	0.75	0.5	2.21
1451	Ar1	0.51	2.78	0.26	0.65	0.09	0.78	0.39	2.32
1452	Ar1	0.94	2.33	0.17	0.59	0.13	0.82	0.37	2.08
1453	Ar1	0.98	2.61	0.18	0.55	0.15	0.82	0.32	2.23
1454	Ar1	0.64	2.5	0.34	0.63	0.14	0.86	0.22	2.05
1455	Ar1	0.74	2.37	0.26	0.63	0.15	0.86	0.26	2.1
1456	Ar1	0.61	2.26	0.25	0.61	0.17	0.75	0.26	2.07
1385	Ar1	0.58	2.63	0.37	0.51	0.06	0.67	0.43	1.75
1457	Ar1	0.62	2.45	0.42	0.71	0.17	0.88	0.24	2
1458	Ar1	0.51	2.5	0.29	0.66	0.16	0.88	0.24	2.3
1459	Ar1	1.2	2.2	0.69	0.26	0.05	0.34	0.11	1.7
1461	Ar1	1.1	1.94	0.91	0.27	0.05	0.34	0.17	1.6
1462	Ar1	1.27	2.35	0.68	0.28	0.05	0.34	0.15	2.02
1391	Ar1	0.56	2.2	0.31	0.5	0.08	0.62	0.3	2.14
1392	Ar1	0.48	1.96	0.36	0.48	0.09	0.58	0.28	1.92
1463	Ar1	1.04	1.85	0.67	0.33	0.08	0.45	0.17	1.56
1464	Ar1	1.04	2.17	0.69	0.35	0.07	0.43	0.15	1.52
1395	Ar1	0.43	2.3	0.28	0.46	0.13	0.65	0.19	1.92
1465	Ar1	1.52	2.33	0.38	0.3	0.06	0.41	0.15	2.07
1466	Ar1	1.18	0.39	0.88	0.38	0.07	0.5	0.17	0.78
1467	Ar1	1.79	2.37	0.31	0.28	0.03	0.37	0.24	1.46
1468	Ar1	1.39	2.09	0.53	0.27	0.05	0.34	0.19	1.85
1400	Ar1	1.01	2.48	0.51	0.54	0.1	0.78	0.26	2.12
1469	Ar1	1.75	2.17	0.34	0.3	0.07	0.41	0.15	1.5
1470	Ar1	1.51	2.56	0.4	0.35	0.07	0.5	0.17	2.1
1471	Ar1	1.14	2.28	0.64	0.37	0.09	0.52	0.15	1.84

1472	Ar1	1.14	2.58	0.39	0.37	0.07	0.43	0.17	2.22
1474	Ar1	1.44	2.09	0.36	0.41	0.09	0.52	0.17	1.94
1476	Ar1	1.71	2.2	0.36	0.33	0.08	0.5	0.15	1.87
1477	Ar1	1.16	2.39	0.68	0.33	0.08	0.45	0.19	1.72
1478	Ar1	1.2	2.22	0.73	0.33	0.06	0.41	0.19	1.95
1479	Ar1	1	2.15	0.82	0.35	0.06	0.45	0.22	1.94
1480	Ar1	1.02	2.15	0.84	0.35	0.06	0.45	0.19	1.92
1481	Ar1	1.13	2.05	0.553385	0.32	0.05	0.41	0.19	1.51
1483	Ar1	1.27	2.2	0.44	0.28	0.06	0.41	0.19	1.8
1413	Ar1	0.93	2.58	0.6	0.57	0.14	0.71	0.19	2.34
1484	Ar1	1.12	2.09	0.67	0.37	0.06	0.43	0.17	1.5
1485	Ar1	1.08	2.28	0.64	0.34	0.06	0.43	0.15	1.51
1486	Ar1	1.41	2.13	0.46	0.34	0.08	0.45	0.15	1.59
1487	Ar1	1.1	2.09	0.62	0.32	0.07	0.41	0.17	1.69
1488	Ar1	2.18	2.3	1.08	0.26	0.05	0.37	0.13	2.06
1489	Ar1	1.46	2.24	1.15	0.2	0.04	0.26	0.13	1.96
1490	Ar1	2.1	1.92	0.86	0.28	0.08	0.39	0.09	1.69
1491	Ar1	2.11	2.28	0.42	0.35	0.08	0.56	0.17	2.12
1492	Ar1	1.33	2.39	0.78	0.37	0.12	0.5	0.13	1.67
1493	Ar1	1.28	1.89	1.12	0.37	0.11	0.52	0.09	1.77
1495	Ar1	1.33	2.24	1.02	0.33	0.07	0.41	0.15	1.88
1496	Ar1	1.22	2	0.69	0.31	0.1	0.41	0.06	2.06
1426	Ar1	0.88	2.2	0.59	0.64	0.1	0.86	0.45	1.55
1497	Ar1	1.74	2.15	0.57	0.34	0.07	0.41	0.11	1.82
1501	Ar1	1.51	2.22	0.59	0.28	0.1	0.43	0.09	1.98
1502	Ar1	1.72	2.37	0.51	0.3	0.08	0.45	0.17	1.9
1503	Ar1	1.46	2.17	0.74	0.32	0.08	0.43	0.06	2.01
1504	Ar1	1.22	2.24	0.88	0.3	0.08	0.45	0.11	1.4
1505	Ar1	1.26	1.34	0.93	0.33	0.05	0.43	0.19	1.35
1506	Ar1	1.05	2.24	0.92	0.37	0.05	0.41	0.22	1.01
1507	Ar1	1.66	2.07	0.49	0.29	0.05	0.43	0.19	1.96
1435	Ar1	0.4	2.41	0.29	0.75	0.17	0.95	0.24	1.73
1508	Ar1	1.33	2.43	0.75	0.28	0.07	0.37	0.13	2.16
1509	Ar1	1.06	2.26	0.88	0.26	0.05	0.37	0.15	2
1510	Ar1	2.21	2.13	0.44	0.24	0.06	0.37	0.13	1.77
1511	Ar1	1.59	2.05	0.71	0.28	0.03	0.34	0.17	2.02
1512	Ar1	1.12	2.41	0.83	0.24	0.05	0.3	0.13	1.8
1514	Ar1	1.07	2.39	0.89	0.19	0.03	0.24	0.11	1.93
1442	Ar1	0.71	2.33	0.22	0.75	0.12	0.9	0.41	2.07
1515	Ar1	1.26	2.37	0.75	0.23	0.05	0.28	0.11	1.66
1516	Ar1	1.15	1.74	0.89	0.22	0.03	0.26	0.13	1.17
1445	Ar1	0.37	2.17	0.13	0.66	0.23	0.95	0.24	1.58
1517	Ar1	1.34	1.92	0.7	0.21	0.04	0.28	0.13	1.5
1447	Ar1	0.63	1.96	0.26	0.68	0.08	0.86	0.5	2.09
1518	Ar1	1.28	2.43	0.93	0.23	0.04	0.28	0.13	1.46
1449	Ar1	0.27	1.94	0.14	0.6	0.11	0.75	0.45	1.87
1519	Ar1	1.88	2.24	0.58	0.22	0.04	0.28	0.11	1.53
1521	Ar1	1.53	1.94	1.01	0.22	0.04	0.32	0.11	1.66
1522	Ar1	1.95	2.22	1.62	0.29	0.09	0.73	0.19	1.44
1523	Ar1	1.91	2.24	1.53	0.35	0.08	0.5	0.13	1.8
1524	Ar1	2.53	2.41	1.76	0.37	0.08	0.47	0.17	1.83
1526	Ar1	1.44	2.11	1.28	0.32	0.07	0.45	0.13	1.82
1527	Ar1	1.56	2.11	1.07	0.33	0.1	0.5	0.09	1.57
1528	Ar1	2.28	2.2	0.75	0.28	0.03	0.32	0.19	2.05

1530	Ar1	1.89	0.45	1.22	0.32	0.11	0.43	0.11	0.81
1531	Ar1	2.19	1.66	1.1	0.22	0.05	0.34	0.11	1.41
1460	Ar1	1.42	2.11	0.64	0.27	0.04	0.34	0.15	1.82
1532	Ar1	1.67	1.14	1.52	0.34	0.05	0.45	0.13	1.3
1533	Ar1	1.69	2.2	1.5	0.28	0.06	0.41	0.15	0.89
1536	Ar1	1.69	2.73	1.42	0.46	0.06	0.56	0.26	2.03
1537	Ar1	1.54	2.35	1.24	0.42	0.09	0.5	0.13	2.09
1538	Ar1	1.52	2.09	1.43	0.35	0.07	0.5	0.19	1.82
1539	Ar1	1.67	2.24	0.81	0.43	0.07	0.56	0.24	2.01
1540	Ar1	1.37	2.22	1.02	0.49	0.12	0.58	0.13	1.92
1541	Ar1	1.25	1.53	1.11	0.46	0.15	0.6	0.17	1.41
1542	Ar1	1.38	2.26	1.23	0.55	0.1	0.67	0.24	1.66
1543	Ar1	1.74	2.13	1.03	0.5	0.15	0.67	0.17	1.72
1544	Ar1	1.14	2.2	0.83	0.48	0.15	0.65	0.06	1.75
1545	Ar1	1.65	2.13	0.65	0.45	0.04	0.56	0.28	2.11
1546	Ar1	1.44	2.2	1.15	0.54	0.08	0.62	0.28	1.77
1547	Ar1	1.23	2.17	1.03	0.35	0.07	0.69	0.26	1.98
1475	Ar1	0.9	2.3	0.61	0.4	0.08	0.5	0.19	2.07
1548	Ar1	1.13	2.22	0.89	0.39	0.17	0.8	0.09	1.68
1549	Ar1	1.42	1.94	0.96	0.48	0.09	0.67	0.3	1.62
1550	Ar1	1.34	2.45	1.17	0.57	0.13	0.69	0.17	1.89
1552	Ar1	1.46	2.52	0.96	0.53	0.16	0.69	0.17	1.91
1553	Ar1	1.23	2.37	0.77	0.56	0.22	0.82	0.09	1.74
1554	Ar1	1.29	2.39	0.92	0.57	0.14	0.73	0.24	1.97
1555	Ar1	1.77	2.3	0.56	0.29	0.07	0.45	0.15	1.56
1556	Ar1	1.24	2.33	0.81	0.27	0.15	0.47	0.06	1.23
1557	Ar1	1.47	2.02	0.8	0.31	0.09	0.47	0.13	1.78
1558	Ar1	1.68	2.2	0.7	0.33	0.09	0.5	0.11	1.38
1559	Ar1	1.21	2	1.01	0.37	0.09	0.5	0.17	1.56
1560	Ar1	1.11	2.09	0.84	0.35	0.09	0.47	0.15	1.4
1561	Ar1	1.15	2	0.79	0.32	0.1	0.47	0.09	1.65
1562	Ar1	1.41	2.05	0.71	0.36	0.08	0.47	0.15	1.76
1563	Ar1	1.09	2.26	0.98	0.24	0.13	0.5	0.04	1.78
1564	Ar1	2.02	2.2	0.49	0.3	0.07	0.45	0.13	2
1565	Ar1	0.98	2.13	0.94	0.34	0.07	0.41	0.09	1.74
1566	Ar1	1.11	1.96	0.84	0.31	0.12	0.5	0.06	1.39
1567	Ar1	1.33	2.11	0.68	0.32	0.07	0.43	0.15	1.59
1568	Ar1	1.46	2.11	0.69	0.42	0.04	0.47	0.3	2.11
1569	Ar1	1.16	2.35	0.85	0.4	0.06	0.5	0.22	1.88
1570	Ar1	1.03	2.67	0.8	0.35	0.11	0.47	0.15	1.84
1498	Ar1	1.39	1.74	1	0.31	0.06	0.39	0.13	1.75
1499	Ar1	1.35	1.83	0.95	0.28	0.08	0.41	0.13	1.89
1500	Ar1	1.2	2.26	1	0.31	0.08	0.45	0.06	1.76
1571	Ar1	1.2	2.26	0.69	0.35	0.08	0.47	0.15	1.84
1572	Ar1	1.46	2.24	0.59	0.35	0.09	0.45	0.17	1.88
1573	Ar1	1.15	2.33	0.74	0.39	0.09	0.54	0.19	2
1574	Ar1	0.97	1.77	0.87	0.46	0.07	0.56	0.26	1.81
1575	Ar1	1.03	2.2	0.84	0.34	0.12	0.65	0.22	1.89
1576	Ar1	0.99	1.64	0.83	0.44	0.12	0.62	0.17	1.31
1577	Ar1	1.21	2.48	0.79	0.36	0.11	0.52	0.11	1.82
1578	Ar1	1.37	2.35	0.54	0.39	0.08	0.5	0.15	2.12
1580	Ar1	1.64	2.26	0.64	0.46	0.09	0.56	0.19	1.92
1581	Ar1	1.6	2.2	0.65	0.24	0.04	0.32	0.17	1.78
1582	Ar1	1.52	2.33	0.93	0.2	0.04	0.28	0.13	2.11

1584	Ar1	1.5	2.26	0.77	0.3	0.06	0.39	0.11	2.27
1513	Ar1	1.37	2.05	0.69	0.27	0.04	0.37	0.17	1.8
1585	Ar1	1.85	1.59	0.89	0.38	0.06	0.47	0.19	1.74
1586	Ar1	0.78	4.67	0.44	0.33	0.05	0.45	0.24	2.08
1587	Ar1	1	2.43	0.59	0.35	0.04	0.43	0.26	2.15
1591	Ar1	0.98	5.56	0.33	0.23	0.05	0.3	0.11	2.19
1592	Ar1	0.88	5.66	0.47	0.22	0.06	0.3	0.09	2.4
1593	Ar1	1	2.54	0.65	0.23	0.04	0.32	0.15	2.1
1520	Ar1	1.97	2.41	0.63	0.23	0.03	0.3	0.15	1.72
1594	Ar1	0.91	2.22	0.62	0.25	0.04	0.37	0.19	2.19
1595	Ar1	0.83	2.33	0.62	0.27	0.05	0.37	0.15	2.5
1596	Ar1	0.43	2.24	0.36	0.24	0.05	0.34	0.13	1.89
1597	Ar1	0.9	2.37	0.33	0.31	0.07	0.39	0.15	2.08
1525	Ar1	2	1.98	1.86	0.22	0.04	0.28	0.13	1.65
1600	Ar1	0.58	2.37	0.33	0.25	0.06	0.34	0.13	1.92
1601	Ar1	0.59	2.11	0.36	0.23	0.05	0.32	0.15	1.98
1602	Ar1	0.56	2.15	0.35	0.28	0.03	0.3	0.17	1.82
1529	Ar1	1.72	1.85	1.33	0.33	0.06	0.43	0.17	1.63
1603	Ar1	0.64	2.33	0.13	0.25	0.05	0.34	0.15	2.02
1605	Ar1	0.52	2	0.22	0.27	0.04	0.32	0.17	1.87
1606	Ar1	1	2.43	0.25	0.28	0.07	0.41	0.15	1.94
1608	Ar1	0.89	2.33	0.23	0.28	0.05	0.34	0.15	2.26
1534	Ar1	1.63	2.05	1.5	0.27	0.04	0.3	0.13	1.61
1535	Ar1	1.7	2.67	1.31	0.19	0.02	0.22	0.13	1.03
1609	Ar1	0.62	2.35	0.35	0.29	0.05	0.37	0.17	2.27
1611	Ar1	0.58	2.63	0.31	0.37	0.06	0.47	0.17	2
1613	Ar1	0.45	2.63	0.14	0.33	0.06	0.39	0.17	2.5
1614	Ar1	0.85	2.26	0.35	0.23	0.04	0.3	0.13	1.48
1615	Ar1	0.65	1.92	0.21	0.25	0.04	0.3	0.13	1.74
1616	Ar1	0.63	1.79	0.35	0.24	0.04	0.3	0.17	1.82
1617	Ar1	0.99	2.45	0.22	0.27	0.04	0.32	0.17	2.06
1618	Ar1	0.82	2.22	0.29	0.27	0.04	0.32	0.13	1.86
1619	Ar1	0.28	5.4	0.22	0.24	0.04	0.32	0.19	1.48
1620	Ar1	0.74	2.05	0.07	0.25	0.06	0.34	0.11	2.45
1622	Ar1	0.93	1.85	0.09	0.36	0.08	0.5	0.22	2.06
1623	Ar1	0.76	2.3	0.25	0.32	0.09	0.5	0.11	1.86
1624	Ar1	0.59	3.96	0.23	0.3	0.08	0.39	0.17	1.75
1626	Ar1	0.55	4.89	0.293542	0.26	0.07	0.37	0.11	1.86
1627	Ar1	0.69	2.26	0.39	0.31	0.06	0.41	0.15	2.32
1551	Ar1	1.35	2.28	1.23	0.56	0.07	0.69	0.41	1.98
1628	Ar1	0.81	2.37	0.33	0.31	0.06	0.43	0.15	1.95
1629	Ar1	1.03	2.09	0.34	0.38	0.06	0.43	0.22	1.77
1630	Ar1	0.98	2.2	0.34	0.36	0.05	0.43	0.22	1.85
1631	Ar1	1.04	2.22	0.39	0.32	0.06	0.41	0.19	1.88
1632	Ar1	1.17	2.3	0.23	0.3	0.04	0.39	0.17	2.26
1634	Ar1	0.52	4.33	0.45	0.33	0.11	0.43	0.06	1.68
1635	Ar1	0.99	2.45	0.33	0.29	0.06	0.39	0.15	2.45
1636	Ar1	1.46	2.15	0.34	0.3	0.06	0.39	0.13	1.86
1638	Ar1	0.98	2.3	0.31	0.29	0.05	0.34	0.13	2.24
1639	Ar1	0.95	2.3	0.43	0.28	0.07	0.34	0.09	1.88
1642	Ar1	1.22	2.26	0.46	0.29	0.06	0.41	0.19	1.99
1643	Ar1	0.76	2.11	0.65	0.26	0.04	0.34	0.19	2.14
1644	Ar1	1.23	2.22	0.64	0.24	0.04	0.32	0.15	1.79
1645	Ar1	2.06	2.09	0.36	0.24	0.05	0.3	0.09	2.01

1646	Ar1	2.16	2.24	0.21	0.23	0.06	0.37	0.11	2.02
1648	Ar1	1.02	2.48	0.76	0.26	0.05	0.32	0.06	2.04
1649	Ar1	1.21	4.63	0.540468	0.22	0.07	0.3	0.11	1.51
1650	Ar1	0.85	2.3	0.59	0.24	0.03	0.32	0.17	1.87
1653	Ar1	1.43	1.77	0.38	0.22	0.03	0.28	0.13	1.56
1658	Ar1	0.87	2.22	0.62	0.23	0.05	0.32	0.13	1.38
1659	Ar1	0.82	2.39	0.43	0.22	0.04	0.26	0.15	1.76
1660	Ar1	0.69	2.11	0.52	0.21	0.06	0.32	0.06	1.7
1661	Ar1	1.28	1.96	0.15	0.23	0.04	0.34	0.17	1.31
1662	Ar1	0.68	2.76	0.38	0.24	0.04	0.32	0.17	1.25
1664	Ar1	0.81	2.5	0.49	0.08	0.03	0.24	0.02	2.21
1666	Ar1	0.98	2.09	0.27	0.2	0.03	0.32	0.15	1.97
1667	Ar1	0.98	1.83	0.5	0.23	0.06	0.34	0.09	1.43
1579	Ar1	1.34	2.11	0.7	0.44	0.06	0.6	0.19	2.22
1668	Ar1	0.74	2.37	0.52	0.25	0.05	0.32	0.13	1.41
1669	Ar1	0.84	2.02	0.5	0.27	0.06	0.37	0.13	1.23
1670	Ar1	0.96	2.37	0.53	0.27	0.07	0.39	0.13	1.67
1583	Ar1	1.54	5.1	0.67	0.3	0.06	0.41	0.15	2.01
1671	Ar1	0.92	2.15	0.72	0.3	0.09	0.45	0.13	1.4
1673	Ar1	1.14	2.5	0.37	0.32	0.07	0.45	0.19	1.89
1674	Ar1	1.1	2.43	0.44	0.31	0.06	0.39	0.17	2.15
1675	Ar1	0.75	2.3	0.51	0.33	0.06	0.43	0.13	2.07
1588	Ar1	0.86	4.78	0.42	0.28	0.06	0.39	0.17	1.59
1589	Ar1	0.91	4.69	0.38	0.29	0.07	0.39	0.11	1.97
1590	Ar1	0.92	4.78	0.4	0.27	0.06	0.39	0.13	2.08
1676	Ar1	0.93	2.24	0.56	0.31	0.05	0.39	0.19	1.85
1677	Ar1	0.72	2.52	0.56	0.21	0.02	0.28	0.15	2.16
1678	Ar1	1.08	2.35	0.51	0.25	0.05	0.32	0.13	2.06
1682	Ar1	0.83	2.07	0.57	0.29	0.04	0.37	0.19	2.06
1683	Ar1	0.57	4.22	0.540558	0.22	0.08	0.32	0.06	1.9
1684	Ar1	1.32	2.48	0.89	0.47	0.14	0.62	0.15	1.92
1685	Ar1	1.16	2.58	0.71	0.49	0.07	0.56	0.24	2.22
1598	Ar1	0.83	5.28	0.23	0.27	0.07	0.41	0.11	1.81
1599	Ar1	0.76	4.67	0.21	0.22	0.05	0.3	0.11	1.99
1687	Ar1	1.22	2.35	0.88	0.38	0.06	0.45	0.22	2.15
1688	Ar1	1.04	2.41	0.85	0.39	0.07	0.45	0.15	2.3
1689	Ar1	0.95	2.3	0.74	0.42	0.11	0.58	0.17	1.67
1690	Ar1	1.56	2.43	0.63	0.43	0.07	0.56	0.22	2.25
1604	Ar1	0.33	0.5	0.3	0.29	0.03	0.37	0.24	2.52
1691	Ar1	1.38	2.28	0.65	0.39	0.09	0.5	0.15	2.18
1692	Ar1	1.3	2.73	0.43	0.48	0.11	0.56	0.09	1.79
1607	Ar1	0.57	2.54	0.46	0.3	0.06	0.41	0.19	1.83
1693	Ar1	0.97	2.28	0.61	0.38	0.1	0.5	0.15	2.02
1694	Ar1	1.04	5.02	0.34	0.35	0.08	0.47	0.15	1.67
1610	Ar1	0.7	2.43	0.37	0.32	0.04	0.37	0.22	1.86
1695	Ar1	0.77	2.3	0.538716	0.37	0.08	0.47	0.19	2.1
1612	Ar1	0.58	2.37	0.12	0.41	0.09	0.54	0.09	1.92
1696	Ar1	1.17	2.2	0.69	0.32	0.11	0.45	0.11	1.74
1697	Ar1	1.01	2.28	0.67	0.31	0.08	0.43	0.15	1.96
1698	Ar1	1.02	2.54	0.6	0.31	0.07	0.43	0.17	2
1699	Ar1	2.08	2.52	0.63	0.39	0.08	0.5	0.13	2.14
1700	Ar1	1.57	2.24	0.71	0.31	0.08	0.43	0.13	1.85
1701	Ar1	0.94	2.15	0.61	0.37	0.09	0.45	0.13	1.76
1702	Ar1	0.87	2.17	0.51	0.33	0.09	0.47	0.17	1.9

1703	Ar1	0.81	2.2	0.66	0.34	0.09	0.45	0.13	1.68
1621	Ar1	0.74	2.05	0.07	0.25	0.06	0.34	0.11	2.45
1704	Ar1	1.06	2.15	0.36	0.28	0.09	0.5	0.11	1.92
1705	Ar1	0.88	2.07	0.35	0.37	0.06	0.47	0.17	1.81
1706	Ar1	1.04	2.41	0.23	0.34	0.06	0.43	0.15	2.05
1625	Ar1	0.53	2.56	0.35	0.33	0.06	0.47	0.15	1.82
1707	Ar1	0.76	2.28	0.45	0.34	0.05	0.45	0.19	1.85
1709	Ar1	0.68	2.37	0.51	0.35	0.09	0.43	0.17	1.24
1710	Ar1	1.22	2.17	0.34	0.33	0.06	0.45	0.19	1.92
1711	Ar1	0.85	1.72	0.54	0.32	0.06	0.41	0.19	1.72
1712	Ar1	0.68	2.17	0.57	0.4	0.06	0.47	0.28	1.85
1713	Ar1	0.79	2.41	0.53	0.38	0.07	0.47	0.22	2
1714	Ar1	1.23	2.43	0.27	0.35	0.08	0.47	0.19	2.22
1633	Ar1	0.78	3.75	0.34	0.3	0.03	0.34	0.19	2.27
1715	Ar1	0.44	2.09	0.36	0.36	0.08	0.54	0.13	2.2
1717	Ar1	0.79	2.26	0.33	0.37	0.07	0.5	0.24	2.07
1718	Ar1	0.79	2.41	0.19	0.35	0.03	0.41	0.26	2.32
1637	Ar1	1.63	2.13	0.28	0.28	0.08	0.43	0.13	1.87
1719	Ar1	0.77	2.41	0.220502	0.33	0.08	0.43	0.19	2
1720	Ar1	0.59	2.37	0.47	0.34	0.05	0.43	0.24	2.2
1640	Ar1	0.77	0.26	0.64	0.27	0.05	0.32	0.13	2.24
1641	Ar1	0.89	0.24	0.8	0.26	0.05	0.34	0.11	1.95
1723	Ar1	0.77	2.09	0.31	0.36	0.06	0.47	0.22	1.93
1724	Ar1	0.67	2.24	0.3	0.31	0.06	0.39	0.17	2.01
1725	Ar1	1.08	2.37	0.22	0.3	0.06	0.37	0.17	2.16
1726	Ar1	0.6	2.39	0.35	0.3	0.05	0.34	0.15	2.18
1727	Ar1	0.63	2.45	0.22	0.29	0.06	0.39	0.13	2.26
1647	Ar1	1.61	2.35	0.62	0.21	0.04	0.28	0.11	1.97
1728	Ar1	0.56	2.41	0.29	0.29	0.04	0.39	0.17	2.21
1729	Ar1	0.84	2.37	0.28	0.3	0.05	0.34	0.13	2.16
1730	Ar1	0.94	1.74	0.34	0.32	0.06	0.43	0.17	1.84
1651	Ar1	0.86	3.81	0.61	0.25	0.04	0.3	0.15	1.62
1652	Ar1	0.79	1.87	0.74	0.25	0.04	0.3	0.17	1.72
1732	eee	5.63	0.09	0.56	0.48	0.31	0.75	0.02	0.3
1654	Ar1	0.86	2.22	0.69	0.22	0.03	0.26	0.15	1.99
1655	Ar1	0.86	2.22	0.69	0.22	0.03	0.26	0.15	1.99
1656	Ar1	0.95	2.26	0.7	0.23	0.04	0.28	0.13	1.94
1657	Ar1	0.89	1.87	0.7	0.23	0.04	0.28	0.15	1.68
1853	eee	0.52	0.73	0.05	0.3	0.11	0.47	0.17	0.53
1859	eee	0.6	0.73	0.45	0.65	0.19	0.75	0.06	0.45
1860	eee	1.68	0.71	0.06	0.48	0.31	0.75	0.02	0.29
2545	Er	0.41	0.62	0.521254	0.15	0.03	0.22	0.13	0.38
2555	Ar2	0.63	0.22	0.6	0.16	0.04	0.24	0.09	0.31
1663	Ar1	0.78	2.26	0.27	0.27	0.06	0.37	0.13	1.41
2566	Er	0.56	0.28	0.52	0.13	0.01	0.17	0.09	0.26
1665	Ar1	1.08	1.85	0.24	0.21	0.04	0.28	0.11	1.82
2568	Ar2	0.84	0.19	0.46	0.16	0.02	0.17	0.09	0.25
2569	Er	0.96	0.82	0.27	0.13	0.02	0.17	0.06	0.28
2574	Er	1.02	0.62	0.29	0.14	0.02	0.19	0.06	0.37
2577	Er	1	0.62	0.4	0.15	0.02	0.17	0.06	0.38
2608	Ar2	1.09	0.69	0.14	0.15	0.01	0.17	0.09	0.43
2613	Ar2	0.56	0.9	0.51	0.13	0.01	0.15	0.11	0.6
1672	Ar1	0.91	0.8	0.83	0.31	0.06	0.45	0.17	1.51
2623	Ar2	0.26	1.12	0.18	0.2	0.03	0.24	0.13	0.47

2625	Ar2	0.39	0.84	0.24	0.18	0.04	0.24	0.06	0.62
2627	Er	0.43	0.82	0.21	0.15	0.04	0.24	0.11	0.52
2629	Ar2	0.63	0.71	0.08	0.19	0.01	0.22	0.15	0.57
2641	Ar2	0.65	0.62	0.06	0.2	0.04	0.22	0.09	0.42
2665	Ar2	0.51	0.69	0.23	0.13	0.02	0.15	0.09	0.33
1679	Ar1	0.77	2.11	0.7	0.24	0.03	0.34	0.19	2.04
1680	Ar1	1.04	4.07	0.38	0.25	0.05	0.3	0.06	1.79
1681	Ar1	0.84	4.07	0.34	0.26	0.05	0.34	0.13	1.48
2671	Ar2	0.49	0.69	0.14	0.14	0.02	0.19	0.11	0.37
2672	Ar2	0.58	0.78	0.06	0.14	0.03	0.19	0.09	0.44
2690	Ar2	0.33	0.65	0.17	0.14	0.02	0.17	0.11	0.4
2693	Er	0.46	0.71	0.15	0.15	0.02	0.17	0.11	0.47
2694	Er	0.48	0.6	0.15	0.13	0.02	0.19	0.11	0.36
2695	Er	0.32	0.28	0.29	0.15	0.02	0.17	0.11	0.37
2698	Er	0.44	0.62	0.26	0.14	0.02	0.17	0.11	0.44
2700	Er	0.27	0.22	0.420074	0.13	0.03	0.19	0.11	0.24
2704	Er	0.45	0.3	0.24	0.13	0.02	0.15	0.09	0.34
2719	Er	0.64	0.6	0.33	0.13	0.03	0.17	0.09	0.36
2721	Er	0.82	0.67	0.22	0.14	0.01	0.15	0.11	0.34
2735	Er	0.42	0.32	0.4	0.17	0.01	0.19	0.15	0.36
2761	Er	0.5	0.24	0.48	0.21	0.03	0.26	0.15	0.54
2762	Ar2	0.5	0.69	0.46	0.21	0.02	0.24	0.17	0.47
2790	Ar2	0.5	0.37	0.29	0.17	0.02	0.19	0.13	0.32
2857	Ar2	0.44	0.28	0.31	0.13	0.01	0.15	0.11	0.24
2923	Er	0.52	0.13	0.42	0.12	0.01	0.13	0.11	0.17
3040	Ar2	0.42	0.28	0.18	0.13	0.02	0.17	0.11	0.26
3054	Er	0.31	0.22	0.179606	0.12	0.02	0.13	0.09	0.22
3055	Er	0.19	0.22	0.262065	0.11	0.02	0.15	0.09	0.18
3056	Er	0.21	0.34	0.157427	0.11	0.01	0.13	0.09	0.21
3060	Er	0.2	0.22	0.13	0.1	0.02	0.13	0.06	0.26
3063	Er	0.29	0.13	0.28	0.12	0.02	0.15	0.06	0.25
3066	Er	0.36	0.54	0.19	0.11	0.02	0.15	0.09	0.29
3067	Er	0.09	0.6	0.387254	0.11	0.02	0.13	0.09	0.33
3070	Ar2	0.5	0.24	0.19	0.11	0.02	0.15	0.06	0.2
1708	Ar1	0.69	2.17	0.46	0.37	0.07	0.47	0.09	2.07
3071	Er	0.22	0.22	0.373299	0.11	0.01	0.13	0.09	0.2
3072	Er	0.2	0.11	0.31316	0.1	0.02	0.11	0.06	0.19
3074	Er	0.09	0.6	0.05	0.14	0.02	0.17	0.11	0.33
3075	Er	0.12	0.65	0.05	0.13	0.02	0.15	0.11	0.33
3076	Er	0.09	0.78	0.05	0.14	0.01	0.15	0.13	0.23
3077	Er	0.06	0.58	0.04	0.12	0.02	0.15	0.11	0.31
3078	Er	0.12	0.6	0.07	0.12	0.01	0.15	0.11	0.36
3080	Er	0.06	0.62	0.04	0.12	0.02	0.15	0.09	0.36
3081	Er	0.09	0.71	0.04	0.13	0.01	0.15	0.11	0.23
3082	Er	0.17	0.62	0.04	0.13	0.01	0.15	0.11	0.32
3083	Er	0.1	0.71	0.05	0.12	0.02	0.15	0.09	0.38
3085	Er	0.09	0.69	0.05	0.12	0.01	0.13	0.11	0.23
1721	Ar1	0.57	2.35	0.41	0.31	0.07	0.41	0.22	1.97
1722	Ar1	0.58	2.07	0.45	0.35	0.06	0.45	0.22	2.02
3086	Er	0.08	0.24	0.05	0.12	0.01	0.13	0.11	0.25
3088	Er	0.06	0.6	0.02	0.13	0	0.13	0.13	0.32
3089	Er	0.12	0.62	0.03	0.12	0.02	0.15	0.09	0.3
3090	Er	0.08	0.65	0.03	0.12	0.01	0.13	0.11	0.36
3091	Er	0.1	0.24	0.06	0.11	0.02	0.13	0.09	0.24

3092	Er	0.11	0.19	0.04	0.11	0.01	0.13	0.11	0.22
3094	Er	0.12	0.5	0.07	0.11	0.01	0.11	0.09	0.3
3096	Er	0.14	0.75	0.03	0.09	0.01	0.11	0.06	0.28
1731	eee	4.31	0.11	4.25	0.23	0.07	0.5	0.04	0.2
3097	Er	0.1	0.65	0.05	0.08	0.01	0.11	0.06	0.31
1733	eee	3.57	0.15	1.64	0.18	0.03	0.24	0.13	0.21
1734	eee	3.27	0.26	0.06	0.25	0.05	0.3	0.13	0.27
1735	eee	2.82	0.32	0.17	0.11	0.03	0.13	0.04	0.14
1736	eee	3.08	0.15	0.07	0.16	0.02	0.17	0.06	0.27
1737	eee	3.06	0.17	0.18	0.17	0.03	0.22	0.09	0.16
1738	Growl	3.13	0.11	2.19	0.1	0.02	0.13	0.02	0.11
1739	eee	3.56	0.19	1.79	0.17	0.05	0.26	0.06	0.17
1740	eee	3.18	0.09	2.19	0.1	0.02	0.15	0.04	0.16
1741	Growl	5.37	0.26	0.1	0.14	0.03	0.17	0.09	0.3
1742	eee	3.46	0.15	1.57	0.14	0.04	0.22	0.02	0.2
1743	eee	3.08	0.37	0.66	0.31	0.09	0.5	0.09	0.27
1744	Growl	4.67	0.13	0.08	0.12	0.03	0.17	0.06	0.25
1745	eee	2.66	0.15	1.61	0.17	0.03	0.22	0.09	0.19
1746	Growl	4.2	0.09	0.46	0.1	0.03	0.15	0.04	0.09
1747	eee	2.19	0.06	2.04	0.08	0.02	0.13	0.02	0.09
1748	eee	2.71	0.26	1.7	0.13	0.04	0.17	0.06	0.17
1749	eee	2.04	0.11	1.88	0.13	0.02	0.17	0.06	0.13
1750	eee	1.94	0.09	1.79	0.09	0.03	0.17	0.06	0.08
1751	Growl	2.44	0.22	1.39	0.14	0.03	0.17	0.06	0.16
1752	eee	1.71	0.15	1.5	0.16	0.03	0.24	0.09	0.15
1753	eee	1.59	0.19	1.31	0.2	0.03	0.24	0.11	0.21
1754	Growl	2.54	0.09	1.3	0.08	0.03	0.13	0.02	0.08
1755	Growl	1.78	0.09	1.54	0.14	0.06	0.26	0.04	0.1
1756	Growl	2.11	0.09	1.54	0.08	0.02	0.13	0.04	0.06
1757	Growl	4.68	0.09	1.7	0.09	0.03	0.15	0.04	0.07
1758	Growl	3.67	0.09	1.41	0.12	0.05	0.22	0.02	0.09
1759	Growl	2.66	0.11	2.11	0.1	0.03	0.15	0.02	0.09
1760	Growl	3.05	0.13	2.34	0.12	0.03	0.17	0.04	0.1
1761	Growl	3.45	0.45	0.18	0.17	0.04	0.22	0.09	0.26
1762	eee	2.27	0.43	1.02	0.14	0.03	0.17	0.06	0.27
1763	eee	2.49	0.15	0.93	0.16	0.02	0.22	0.09	0.16
1764	eee	1.64	0.13	1.39	0.15	0.03	0.19	0.11	0.21
1765	eee	1.54	0.15	0.82	0.17	0.04	0.26	0.11	0.2
1766	eee	2.36	0.47	0.97	0.17	0.03	0.24	0.09	0.28
1767	eee	2.59	0.37	0.74	0.15	0.03	0.22	0.09	0.22
1768	eee	2.41	0.15	1.41	0.15	0.02	0.19	0.09	0.16
1769	Growl	3.46	0.13	0.2	0.16	0.04	0.22	0.06	0.19
1770	Growl	3.56	0.15	0.07	0.14	0.02	0.17	0.06	0.21
1771	eee	2.02	0.15	1.18	0.16	0.03	0.19	0.06	0.22
1772	eee	1.76	0.37	0.3	0.14	0.03	0.22	0.09	0.18
1773	eee	2.01	0.15	0.14	0.15	0.02	0.17	0.06	0.15
1774	eee	1.67	0.22	0.31	0.17	0.03	0.22	0.09	0.15
1775	eee	1.73	0.28	0.06	0.2	0.05	0.26	0.11	0.23
1776	eee	1.27	0.17	0.09	0.19	0.03	0.24	0.13	0.28
1777	eee	1.52	0.17	0.05	0.14	0.02	0.19	0.06	0.12
1778	eee	1.78	0.17	0.98	0.17	0.03	0.26	0.11	0.16
1779	eee	2.77	0.11	0.1	0.12	0.03	0.17	0.06	0.14
1780	eee	1.67	0.13	0.89	0.13	0.02	0.17	0.06	0.13
1781	Growl	2.72	0.37	0.25	0.16	0.04	0.22	0.02	0.19

1782	eee	1.86	0.9	0.79	0.15	0.03	0.22	0.04	0.18
1783	Growl	3.05	0.43	0.17	0.16	0.05	0.24	0.09	0.29
1784	eee	1.76	0.15	1.22	0.13	0.03	0.17	0.06	0.16
1785	Growl	2.69	0.43	0.13	0.15	0.04	0.22	0.09	0.26
1786	eee	2.32	0.11	0.47	0.12	0.04	0.19	0.06	0.11
1787	eee	1.78	0.17	0.7	0.14	0.03	0.19	0.06	0.15
1788	eee	1.55	0.3	0.91	0.16	0.03	0.19	0.09	0.2
1789	eee	1.37	0.13	1.07	0.13	0.02	0.17	0.09	0.14
1790	eee	1.5	0.13	0.67	0.13	0.02	0.17	0.09	0.14
1791	eee	1.41	0.37	0.96	0.19	0.03	0.24	0.13	0.24
1792	eee	2	0.13	0.45	0.16	0.04	0.24	0.11	0.15
1793	Growl	1.46	0.17	0.83	0.18	0.03	0.24	0.13	0.16
1794	Growl	0.57	0.13	0.25	0.14	0.04	0.26	0.09	0.13
1795	eee	0.45	0.19	0.33	0.17	0.02	0.19	0.13	0.18
1796	Growl	0.85	0.13	0.16	0.13	0.03	0.19	0.04	0.14
1797	eee	0.52	0.09	0.35	0.1	0.04	0.15	0.04	0.13
1798	eee	0.76	0.15	0.19	0.17	0.02	0.22	0.13	0.18
1799	Growl	0.86	0.13	0.09	0.13	0.03	0.19	0.06	0.18
1800	eee	0.77	0.15	0.03	0.16	0.02	0.19	0.11	0.15
1801	Growl	0.21	0.37	0.11	0.16	0.05	0.24	0.09	0.18
1802	Growl	0.44	0.09	0.14	0.08	0.03	0.15	0.06	0.09
1803	eee	0.45	0.26	0.06	0.08	0.03	0.13	0.04	0.14
1804	Growl	0.41	0.13	0.06	0.14	0.03	0.19	0.09	0.13
1805	Growl	0.31	0.15	0.06	0.13	0.04	0.22	0.06	0.11
1806	Growl	0.22	0.3	0.07	0.14	0.04	0.19	0.09	0.15
1807	Growl	0.41	0.09	0.28	0.07	0.02	0.09	0.02	0.1
1808	Growl	0.44	0.09	0.17	0.07	0.02	0.11	0.02	0.06
1809	Growl	0.6	0.22	0.34	0.07	0.02	0.13	0.02	0.09
1810	Growl	0.59	0.09	0.51	0.08	0.03	0.19	0.04	0.07
1811	Growl	1.36	0.09	1.09	0.1	0.03	0.15	0.04	0.12
1812	Growl	1.82	0.09	0.84	0.08	0.02	0.15	0.02	0.07
1813	Growl	1.48	0.11	0.84	0.1	0.03	0.19	0.04	0.07
1814	Growl	0.87	0.32	0.79	0.15	0.04	0.19	0.06	0.19
1815	Growl	1.28	0.15	0.49	0.15	0.04	0.24	0.06	0.12
1816	Growl	1.12	0.11	0.9	0.1	0.03	0.17	0.04	0.09
1817	Growl	1.27	0.09	0.75	0.08	0.03	0.13	0.02	0.07
1818	Growl	1.23	0.09	0.57	0.09	0.03	0.13	0.02	0.07
1819	Growl	1.27	0.09	0.34	0.11	0.03	0.19	0.04	0.12
1820	Growl	1.29	0.09	0.36	0.1	0.03	0.17	0.04	0.09
1821	Growl	1.04	0.09	0.19	0.1	0.02	0.13	0.04	0.09
1822	Growl	1.4	0.09	0.01	0.08	0.03	0.15	0.02	0.07
1823	Growl	1.49	0.09	0.08	0.08	0.03	0.15	0.02	0.08
1824	Growl	1.08	0.13	0.41	0.12	0.02	0.17	0.09	0.12
1825	Growl	0.77	0.13	0.66	0.11	0.02	0.17	0.04	0.1
1826	Growl	1.72	0.11	0.03	0.08	0.02	0.15	0.04	0.07
1827	eee	0.74	0.13	0.58	0.14	0.03	0.19	0.04	0.21
1828	eee	0.87	0.34	0.57	0.09	0.02	0.11	0.04	0.12
1829	Growl	0.9	0.09	0.51	0.1	0.03	0.17	0.04	0.1
1830	eee	0.73	0.11	0.68	0.11	0.02	0.15	0.09	0.12
1831	eee	1.09	0.13	0.39	0.13	0.03	0.17	0.04	0.14
1832	eee	1.08	0.17	0.35	0.16	0.02	0.22	0.11	0.15
1833	eee	1.25	0.15	0.14	0.17	0.03	0.24	0.09	0.14
1834	Growl	1.31	0.41	0.09	0.13	0.02	0.17	0.04	0.15
1835	eee	1.14	0.28	0.09	0.11	0.03	0.15	0.04	0.19

1836	eee	0.67	0.13	0.09	0.13	0.01	0.15	0.11	0.12
1837	eee	0.74	0.28	0.1	0.12	0.03	0.15	0.04	0.16
1838	eee	0.79	0.37	0.41	0.19	0.03	0.24	0.13	0.23
1839	eee	0.82	0.24	0.51	0.17	0.04	0.22	0.06	0.15
1840	eee	1.06	0.17	0.41	0.18	0.03	0.24	0.11	0.16
1841	eee	1.09	0.15	0.18	0.14	0.03	0.19	0.06	0.15
1842	eee	0.81	0.15	0.42	0.16	0.02	0.19	0.13	0.16
1843	eee	0.89	0.37	0.11	0.16	0.03	0.19	0.09	0.18
1844	Growl	0.94	0.13	0.23	0.15	0.03	0.19	0.09	0.17
1845	eee	0.96	0.13	0.22	0.13	0.03	0.19	0.06	0.16
1846	eee	0.7	0.5	0.19	0.17	0.03	0.24	0.11	0.38
1847	eee	0.68	0.47	0.27	0.17	0.03	0.22	0.06	0.35
1848	eee	0.79	0.13	0.07	0.14	0.03	0.17	0.09	0.16
1849	eee	0.54	0.13	0.26	0.13	0.02	0.17	0.09	0.15
1850	eee	0.57	0.15	0.17	0.16	0.02	0.22	0.13	0.14
1851	eee	0.48	0.17	0.21	0.18	0.03	0.24	0.13	0.19
1852	eee	0.32	0.28	0.27	0.17	0.04	0.22	0.11	0.18
3098	Er	0.14	0.28	0.06	0.09	0.02	0.11	0.06	0.26
1854	Growl	0.36	0.45	0.16	0.24	0.03	0.3	0.17	0.31
1855	eee	1.05	0.39	0.07	0.36	0.07	0.47	0.09	0.29
1856	Growl	1.22	0.34	0.18	0.16	0.03	0.22	0.09	0.73
1857	Growl	1.06	0.34	0.07	0.16	0.03	0.24	0.09	0.25
1858	eee	0.9	0.43	0.51	0.15	0.04	0.24	0.06	0.28
3101	Er	0.12	0.88	0.08	0.14	0.02	0.17	0.11	0.42
3105	Er	0.12	0.65	0.07	0.11	0.02	0.13	0.09	0.33
1861	eee	1.52	1.4	0.5	0.23	0.11	0.6	0.04	0.42
1862	Growl	1.66	0.37	0.27	0.16	0.03	0.22	0.09	0.98
1863	eee	0.94	1.31	0.89	0.23	0.03	0.28	0.13	1.16
1864	eee	1.02	0.3	0.68	0.12	0.03	0.17	0.09	0.17
1865	eee	1.25	0.3	0.62	0.14	0.03	0.17	0.06	0.21
1866	Growl	1.51	0.24	0.33	0.14	0.03	0.19	0.06	0.16
1867	eee	1.12	0.15	0.63	0.12	0.03	0.15	0.06	0.15
1868	Growl	1.76	0.11	0.36	0.13	0.03	0.17	0.02	0.13
1869	Growl	1.82	0.09	0.29	0.1	0.03	0.15	0.02	0.12
1870	Growl	2.52	0.09	0.11	0.1	0.03	0.15	0.04	0.09
1871	Growl	2.06	0.13	0.58	0.14	0.03	0.19	0.06	0.16
1872	eee	1.67	0.15	0.93	0.17	0.04	0.24	0.06	0.19
1873	eee	1.31	0.15	0.97	0.11	0.03	0.15	0.04	0.14
1874	Growl	2.11	0.09	0.2	0.1	0.03	0.15	0.04	0.09
1875	eee	2.62	0.3	0.59	0.2	0.04	0.26	0.11	0.23
1876	Growl	1.89	0.09	0.9	0.12	0.03	0.15	0.04	0.14
1877	Growl	1.44	0.13	1.1	0.11	0.03	0.17	0.06	0.11
1878	eee	0.75	0.15	0.02	0.15	0.03	0.19	0.09	0.17
1879	eee	0.54	0.15	0.3	0.15	0.01	0.19	0.13	0.13
1880	eee	1.29	0.11	0.07	0.15	0.03	0.19	0.09	0.15
1881	eee	0.94	0.37	0.1	0.13	0.03	0.19	0.09	0.15
1882	eee	1.23	0.22	0.2	0.14	0.02	0.15	0.09	0.22
1883	eee	0.84	0.13	0.24	0.15	0.02	0.22	0.11	0.18
1884	eee	1.06	0.17	0.25	0.17	0.02	0.19	0.11	0.18
1885	eee	1.14	0.13	0.22	0.15	0.03	0.24	0.09	0.21
1886	eee	1.26	0.17	0.54	0.2	0.04	0.24	0.11	0.22
1887	eee	1.25	0.17	0.34	0.18	0.03	0.22	0.09	0.2
1888	eee	1.71	0.11	0.52	0.11	0.03	0.17	0.02	0.14
1889	Growl	2.45	0.34	0.07	0.15	0.02	0.17	0.09	0.26

1890	eee	1.25	0.15	0.9	0.18	0.03	0.22	0.09	0.19
1891	eee	1.42	0.17	0.56	0.17	0.03	0.22	0.11	0.17
1892	Growl	2.05	0.13	0.17	0.14	0.03	0.17	0.04	0.21
1893	eee	2.09	0.15	0.06	0.16	0.03	0.19	0.06	0.17
1894	Growl	1.73	0.13	0.32	0.14	0.04	0.19	0.04	0.17
1895	eee	2.26	1.25	0.09	0.12	0.06	0.28	0.02	0.23
1896	eee	1.44	0.39	0.55	0.38	0.06	0.5	0.09	0.32
1897	Growl	1.59	0.41	0.31	0.18	0.04	0.26	0.11	0.25
1898	eee	1.05	0.41	0.66	0.17	0.05	0.26	0.09	0.23
1899	Growl	1.85	0.13	0.19	0.14	0.03	0.19	0.06	0.16
1900	eee	2.05	0.15	0.13	0.15	0.03	0.24	0.06	0.13
1901	eee	1.38	0.41	0.67	0.16	0.03	0.22	0.09	0.2
1902	Chuff	0.29	0.17	0.09	0.22	0.06	0.37	0.15	0.49
1903	Chuff	0.29	0.24	0.2	0.21	0.05	0.3	0.13	0.9
1904	Chuff	0.34	0.3	0.01	0.21	0.08	0.3	0.11	0.66
1905	Chuff	0.18	0.17	0.02	0.18	0.03	0.26	0.15	1.02
1906	Chuff	0.25	0.19	0.07	0.19	0.04	0.26	0.13	0.31
1907	Chuff	0.27	0.19	0.13	0.19	0.03	0.26	0.15	0.25
1908	Chuff	0.3	0.19	0.16	0.18	0.04	0.28	0.09	0.33
1909	Chuff	0.44	0.24	0.22	0.2	0.05	0.28	0.11	0.88
1910	Chuff	0.23	0.17	0.09	0.2	0.04	0.3	0.15	0.73
1911	Chuff	0.22	0.17	0.09	0.2	0.04	0.28	0.15	0.95
1912	Chuff	0.25	0.19	0.16	0.17	0.04	0.24	0.09	0.97
1913	Chuff	0.21	0.26	0.08	0.26	0.06	0.37	0.17	0.6
1914	Chuff	0.21	0.17	0.09	0.2	0.02	0.24	0.17	1.29
1915	Chuff	0.28	0.28	0.16	0.28	0.07	0.39	0.17	1.16
1916	Chuff	0.3	0.28	0.16	0.28	0.06	0.41	0.17	0.96
1917	Chuff	0.37	1.85	0.24	0.22	0.06	0.3	0.09	0.7
1918	Chuff	0.32	0.47	0.03	0.25	0.03	0.28	0.19	0.41
1919	Chuff	0.25	0.37	0.13	0.19	0.06	0.28	0.09	0.59
1920	Chuff	0.2	0.17	0.09	0.18	0.04	0.26	0.11	0.49
1921	Chuff	0.35	0.19	0.17	0.19	0.02	0.26	0.15	0.41
1922	Chuff	0.29	0.15	0.04	0.13	0.05	0.24	0.04	0.31
1923	Chuff	0.22	0.19	0.1	0.19	0.04	0.24	0.11	0.38
1924	Chuff	0.23	0.04	0.09	0.17	0.04	0.24	0.11	0.2
1925	Chuff	0.24	0.32	0.15	0.2	0.01	0.22	0.17	0.37
1926	Chuff	0.4	0.17	0.26	0.16	0.04	0.26	0.06	0.29
1927	Chuff	0.33	0.19	0.11	0.22	0.05	0.3	0.06	0.38
1928	Chuff	0.39	0.45	0.15	0.17	0.04	0.28	0.09	0.29
1929	Chuff	0.31	0.19	0.04	0.18	0.03	0.28	0.11	0.43
1930	Chuff	0.39	0.3	0.11	0.21	0.04	0.26	0.13	0.45
1931	Chuff	0.3	0.32	0.11	0.19	0.05	0.26	0.06	0.41
1932	Chuff	0.23	0.39	0.14	0.34	0.07	0.39	0.15	0.46
1933	Chuff	0.24	0.19	0.03	0.24	0.04	0.32	0.19	0.7
1934	Chuff	0.32	0.34	0.14	0.23	0.07	0.32	0.11	0.45
1935	Chuff	0.26	0.32	0.13	0.24	0.06	0.32	0.13	0.47
1936	Chuff	0.48	0.17	0.03	0.19	0.05	0.28	0.11	0.35
1937	Chuff	0.36	0.15	0.14	0.16	0.03	0.22	0.13	0.64
1938	Chuff	0.31	0.34	0.14	0.18	0.03	0.22	0.13	0.42
1939	Chuff	0.32	0.15	0.18	0.2	0.04	0.24	0.11	0.34
1940	Chuff	0.3	0.19	0.16	0.21	0.03	0.28	0.15	0.41
1941	Chuff	0.4	0.15	0.18	0.2	0.05	0.28	0.11	0.34
1942	Chuff	0.39	0.19	0.16	0.2	0.05	0.28	0.11	0.42
1943	Chuff	0.24	0.8	0.02	0.21	0.04	0.28	0.15	0.37

1944	Chuff	0.23	0.41	0.11	0.2	0.03	0.28	0.15	0.63
1945	Chuff	0.33	0.19	0.15	0.2	0.04	0.28	0.15	0.56
1946	Chuff	0.31	0.22	0.13	0.2	0.04	0.28	0.13	0.65
1947	Chuff	0.43	0.22	0.2	0.19	0.04	0.24	0.11	0.52
1948	Chuff	0.32	0.22	0.23	0.2	0.06	0.28	0.06	0.52
1949	Chuff	0.27	0.17	0.16	0.19	0.04	0.28	0.15	0.51
1950	Chuff	0.33	0.15	0.24	0.21	0.04	0.28	0.15	0.65
1951	Chuff	0.27	0.28	0.05	0.24	0.08	0.41	0.11	0.33
1952	Chuff	0.29	0.17	0.09	0.22	0.06	0.37	0.15	0.44
1953	Chuff	0.4	0.19	0.18	0.24	0.05	0.32	0.17	0.29
1954	Chuff	0.29	0.45	0.15	0.2	0.07	0.28	0.13	0.39
1955	Chuff	0.21	0.24	0.16	0.25	0.05	0.32	0.15	0.48
1956	Chuff	0.3	0.34	0.13	0.21	0.05	0.28	0.15	0.38
1957	Chuff	0.29	0.37	0.08	0.22	0.04	0.26	0.15	0.41
1958	Chuff	0.21	0.17	0.08	0.22	0.05	0.28	0.15	0.36
1959	Chuff	0.33	0.22	0.11	0.22	0.04	0.28	0.13	0.28
1960	Chuff	0.32	0.15	0.17	0.21	0.04	0.28	0.15	0.39
1961	Chuff	0.31	0.22	0.1	0.2	0.05	0.28	0.11	0.44
1962	Chuff	0.41	0.45	0.16	0.19	0.04	0.24	0.13	0.44
1963	Chuff	0.34	0.19	0.24	0.22	0.03	0.28	0.15	0.45
1964	Chuff	0.39	0.17	0.11	0.19	0.05	0.26	0.11	0.34
1965	Chuff	0.35	0.15	0.15	0.18	0.04	0.24	0.13	0.41
1966	Chuff	0.37	0.39	0.16	0.22	0.04	0.28	0.13	0.33
1967	Chuff	0.3	0.19	0.23	0.21	0.04	0.28	0.15	0.41
1968	Chuff	0.49	0.13	0.19	0.17	0.07	0.28	0.04	0.2
1969	Chuff	0.46	0.17	0.34	0.2	0.03	0.28	0.13	0.37
1970	Chuff	0.39	0.15	0.18	0.21	0.05	0.28	0.09	0.38
1971	Chuff	0.3	0.15	0.08	0.15	0.03	0.22	0.11	0.55
1972	Chuff	0.23	0.17	0.15	0.16	0.06	0.26	0.06	0.41
1973	Chuff	0.3	0.17	0.17	0.18	0.03	0.22	0.13	0.29
1974	Chuff	0.3	0.37	0.1	0.18	0.03	0.24	0.13	0.26
1975	Chuff	0.36	0.3	0.04	0.17	0.04	0.22	0.11	0.44
1976	Chuff	0.33	0.17	0.17	0.18	0.03	0.24	0.11	0.38
1977	Chuff	0.22	0.34	0.06	0.18	0.04	0.24	0.13	0.39
1978	Chuff	0.24	0.17	0.04	0.18	0.02	0.26	0.15	0.23
1979	Chuff	0.25	0.19	0.02	0.2	0.04	0.28	0.13	0.2
1980	Chuff	0.27	0.17	0.02	0.17	0.04	0.24	0.11	0.3
1981	Chuff	0.25	0.15	0.07	0.16	0.02	0.19	0.13	0.21
1982	Chuff	0.24	0.41	0.07	0.18	0.03	0.24	0.11	0.24
1983	Chuff	0.29	0.19	0.07	0.19	0.02	0.22	0.13	0.28
1984	Chuff	0.3	0.17	0.1	0.15	0.04	0.24	0.09	0.32
1985	Chuff	0.31	0.15	0.08	0.16	0.03	0.24	0.09	0.24
1986	Chuff	0.24	0.15	0.11	0.16	0.02	0.22	0.13	0.22
1987	Chuff	0.35	0.34	0.21	0.1	0.03	0.15	0.02	0.27
1988	Chuff	0.39	0.15	0.19	0.14	0.05	0.19	0.02	0.44
1989	Chuff	0.4	0.17	0.18	0.17	0.04	0.24	0.09	0.44
1990	Chuff	0.32	0.43	0.17	0.14	0.03	0.19	0.09	0.38
1991	Chuff	0.2	0.17	0.02	0.18	0.03	0.28	0.15	0.22
1992	Chuff	0.19	0.28	0.02	0.21	0.04	0.26	0.17	0.26
1993	Chuff	0.2	0.17	0.09	0.17	0.04	0.24	0.11	0.32
1994	Chuff	0.28	0.15	0.11	0.16	0.03	0.22	0.13	0.26
1995	Chuff	0.31	0.22	0.1	0.18	0.02	0.22	0.15	0.34
1996	Chuff	0.31	0.15	0.03	0.17	0.02	0.22	0.11	0.26
1997	Chuff	0.23	0.47	0.04	0.15	0.02	0.19	0.11	0.35

1998	Chuff	0.5	0.19	0.3	0.2	0.05	0.28	0.02	0.25
1999	Chuff	0.47	0.15	0.25	0.17	0.03	0.22	0.13	0.23
2000	Chuff	0.5	0.15	0.23	0.16	0.03	0.19	0.09	0.32
2001	Chuff	0.39	0.15	0.26	0.17	0.05	0.24	0.06	0.23
2002	Chuff	0.32	0.13	0.26	0.17	0.06	0.26	0.06	0.19
2003	Chuff	0.42	0.39	0.21	0.17	0.05	0.24	0.09	0.28
2004	Chuff	0.45	0.17	0.16	0.16	0.04	0.24	0.04	0.32
2005	Chuff	0.33	0.15	0.17	0.15	0.02	0.19	0.13	0.27
2006	Chuff	0.28	0.15	0.16	0.13	0.03	0.22	0.09	0.21
2007	Chuff	0.32	0.28	0.14	0.16	0.03	0.19	0.11	0.27
2008	Chuff	0.38	0.15	0.1	0.15	0.02	0.19	0.11	0.2
2009	Chuff	0.39	0.13	0.1	0.15	0.03	0.19	0.11	0.2
2010	Chuff	0.45	0.52	0.26	0.13	0.03	0.15	0.06	0.26
2011	Chuff	0.33	0.13	0.18	0.13	0.03	0.19	0.06	0.29
2012	Chuff	0.37	0.13	0.18	0.13	0.03	0.19	0.06	0.29
2013	Chuff	0.26	0.13	0.1	0.15	0.04	0.19	0.09	0.22
2014	Chuff	0.23	0.37	0.09	0.14	0.03	0.19	0.11	0.27
2015	Chuff	0.24	0.17	0.1	0.18	0.03	0.24	0.09	0.24
2016	Chuff	0.36	0.17	0.02	0.16	0.02	0.24	0.13	0.22
2017	Chuff	0.3	0.28	0.03	0.14	0.03	0.22	0.11	0.26
2018	Chuff	0.31	0.34	0.02	0.16	0.02	0.22	0.11	0.25
2019	Chuff	0.29	0.15	0.02	0.16	0.02	0.22	0.13	0.23
2020	Chuff	0.2	0.19	0.09	0.2	0.03	0.26	0.17	0.27
2021	Chuff	0.25	0.17	0.02	0.19	0.03	0.26	0.15	0.2
2022	Chuff	0.22	0.19	0.08	0.19	0.03	0.24	0.15	0.21
2023	Chuff	0.22	0.17	0.09	0.19	0.03	0.24	0.13	0.26
2024	Chuff	0.22	0.17	0.1	0.17	0.03	0.24	0.13	0.25
2025	Chuff	0.18	0.17	0.1	0.17	0.05	0.26	0.06	0.28
2026	Chuff	0.34	0.32	0.11	0.13	0.03	0.17	0.04	0.31
2027	Chuff	0.32	0.24	0.16	0.15	0.03	0.19	0.11	0.2
2028	Chuff	0.29	0.13	0.1	0.15	0.04	0.22	0.09	0.26
2029	Chuff	0.4	0.15	0.16	0.16	0.02	0.22	0.11	0.27
2030	Chuff	0.4	0.13	0.1	0.16	0.02	0.19	0.13	0.36
2031	Chuff	0.32	0.17	0.09	0.16	0.04	0.22	0.06	0.33
2032	Chuff	0.26	0.3	0.14	0.19	0.03	0.22	0.13	0.45
2033	Chuff	0.29	0.17	0.15	0.18	0.04	0.24	0.09	0.3
2034	Chuff	0.4	0.17	0.12	0.19	0.04	0.26	0.11	0.24
2035	Chuff	0.32	0.17	0.11	0.18	0.04	0.24	0.11	0.3
2036	Chuff	0.34	0.17	0.11	0.2	0.05	0.28	0.06	0.29
2037	Chuff	0.29	0.17	0.17	0.17	0.02	0.24	0.13	0.38
2038	Chuff	0.47	0.28	0.02	0.18	0.02	0.22	0.13	0.3
2039	Chuff	0.42	0.13	0.14	0.18	0.03	0.22	0.13	0.3
2040	Chuff	0.43	0.19	0.11	0.17	0.03	0.22	0.11	0.26
2041	Chuff	0.32	0.19	0.1	0.2	0.02	0.24	0.17	0.29
2042	Chuff	0.32	0.39	0.09	0.19	0.03	0.26	0.15	0.23
2043	Chuff	0.3	0.22	0.09	0.21	0.04	0.28	0.15	0.29
2044	Chuff	0.31	0.24	0.16	0.24	0.02	0.28	0.19	0.35
2045	Chuff	0.38	0.22	0.1	0.22	0.04	0.28	0.13	0.29
2046	Chuff	0.32	0.17	0.08	0.19	0.03	0.3	0.13	0.26
2047	Chuff	0.28	0.47	0.1	0.19	0.03	0.24	0.15	0.25
2048	Chuff	0.22	0.19	0.1	0.19	0.05	0.3	0.09	0.2
2049	Chuff	0.29	0.19	0.08	0.2	0.03	0.28	0.17	0.22
2050	Chuff	0.51	0.28	0.2	0.14	0.02	0.17	0.09	0.23
2051	Chuff	0.41	0.3	0.13	0.14	0.02	0.19	0.11	0.18

2052	Chuff	0.31	0.28	0.11	0.13	0.03	0.17	0.09	0.24
2053	Chuff	0.38	0.28	0.17	0.15	0.02	0.19	0.13	0.26
2054	Chuff	0.32	0.11	0.18	0.15	0.04	0.22	0.06	0.21
2055	Chuff	0.39	0.13	0.17	0.15	0.02	0.19	0.11	0.18
2056	Chuff	0.39	0.43	0.12	0.15	0.02	0.19	0.11	0.22
2057	Chuff	0.4	0.15	0.19	0.15	0.03	0.22	0.09	0.17
2058	Chuff	0.37	0.32	0.1	0.14	0.02	0.17	0.11	0.2
2059	Chuff	0.43	0.15	0.12	0.15	0.03	0.19	0.11	0.17
2060	Chuff	0.29	0.13	0.03	0.15	0.03	0.19	0.11	0.21
2061	Chuff	0.21	0.17	0.09	0.18	0.02	0.22	0.15	0.21
2062	Chuff	0.23	0.47	0.08	0.15	0.02	0.22	0.13	0.21
2063	Chuff	0.24	0.15	0.09	0.15	0.03	0.22	0.11	0.26
2064	Chuff	0.31	0.13	0.08	0.13	0.02	0.22	0.09	0.17
2065	Chuff	0.26	0.28	0.08	0.14	0.03	0.19	0.09	0.23
2066	Chuff	0.31	0.3	0.1	0.14	0.02	0.19	0.11	0.24
2067	Chuff	0.23	0.19	0.09	0.19	0.02	0.24	0.15	0.19
2068	Chuff	0.28	0.15	0.03	0.16	0.02	0.22	0.13	0.17
2069	Chuff	0.23	0.15	0.1	0.16	0.03	0.19	0.09	0.25
2070	Chuff	0.35	0.17	0.12	0.18	0.02	0.24	0.15	0.21
2071	Chuff	0.31	0.19	0.16	0.19	0.02	0.24	0.15	0.21
2072	Chuff	0.31	0.17	0.09	0.17	0.03	0.24	0.13	0.18
2073	Chuff	0.29	0.41	0.16	0.18	0.03	0.24	0.11	0.23
2074	Chuff	0.37	0.22	0.1	0.19	0.04	0.24	0.09	0.19
2075	Chuff	0.29	0.17	0.17	0.17	0.02	0.22	0.13	0.31
2076	Chuff	0.35	0.28	0.09	0.17	0.03	0.22	0.13	0.26
2077	Chuff	0.27	0.15	0.15	0.17	0.03	0.22	0.13	0.23
2078	Chuff	0.3	0.19	0.12	0.19	0.03	0.24	0.13	0.23
2079	Chuff	0.28	0.13	0.1	0.16	0.03	0.22	0.13	0.27
2080	Chuff	0.37	0.3	0.11	0.19	0.03	0.22	0.13	0.24
2081	Chuff	0.45	0.28	0.16	0.17	0.04	0.22	0.09	0.25
2082	Chuff	0.41	0.43	0.14	0.17	0.02	0.22	0.13	0.24
2083	Chuff	0.31	0.13	0.15	0.16	0.04	0.22	0.09	0.24
2084	Chuff	0.4	0.13	0.13	0.16	0.04	0.24	0.11	0.19
2085	Chuff	0.3	0.22	0.17	0.22	0.03	0.28	0.15	0.4
2086	Chuff	0.29	0.17	0.16	0.21	0.04	0.28	0.15	0.35
2087	Chuff	0.35	0.22	0.15	0.23	0.03	0.28	0.17	0.33
2088	Chuff	0.38	0.15	0.26	0.22	0.06	0.3	0.13	0.31
2089	Chuff	0.28	0.41	0.14	0.23	0.06	0.3	0.13	0.29
2090	Chuff	0.22	0.17	0.17	0.23	0.04	0.28	0.17	0.53
2091	Chuff	0.25	0.13	0.1	0.19	0.07	0.26	0.09	0.26
2092	Chuff	0.29	0.26	0.05	0.2	0.08	0.3	0.04	0.23
2093	Chuff	0.22	0.19	0.16	0.2	0.03	0.28	0.17	0.23
2094	Chuff	0.23	0.22	0.15	0.21	0.04	0.28	0.15	0.37
2095	Chuff	0.25	0.15	0.1	0.2	0.05	0.28	0.13	0.28
2096	Chuff	0.27	0.34	0.06	0.22	0.03	0.28	0.17	0.28
2097	Chuff	0.36	0.22	0.09	0.22	0.03	0.28	0.17	0.27
2098	Chuff	0.25	0.39	0.08	0.22	0.05	0.28	0.13	0.32
2099	Chuff	0.19	0.26	0.15	0.27	0.04	0.32	0.19	0.29
2100	Chuff	0.32	0.47	0.16	0.25	0.04	0.3	0.17	0.39
2101	Chuff	0.34	0.47	0.09	0.24	0.03	0.3	0.17	0.39
2102	Chuff	0.55	0.17	0.41	0.22	0.05	0.3	0.09	0.33
2103	Chuff	0.39	0.13	0.34	0.2	0.06	0.3	0.11	0.19
2104	Chuff	0.47	0.17	0.35	0.21	0.04	0.28	0.15	0.27
2105	Chuff	0.48	0.15	0.28	0.2	0.04	0.26	0.13	0.37

2106	Chuff	0.3	0.15	0.24	0.18	0.03	0.26	0.15	0.23
2107	Chuff	0.32	0.17	0.16	0.19	0.05	0.24	0.09	0.21
2108	Chuff	0.31	0.24	0.22	0.19	0.06	0.24	0.06	0.32
2109	Chuff	0.39	0.22	0.17	0.2	0.04	0.24	0.11	0.23
2110	Chuff	0.34	0.15	0.19	0.19	0.06	0.28	0.09	0.24
2111	Chuff	0.42	0.22	0.21	0.19	0.04	0.28	0.13	0.25
2112	Chuff	0.4	0.17	0.18	0.21	0.04	0.28	0.15	0.22
2113	Chuff	0.37	0.22	0.18	0.22	0.03	0.28	0.13	0.32
2114	Chuff	0.4	0.19	0.16	0.21	0.03	0.28	0.15	0.26
2115	Chuff	0.34	0.19	0.17	0.18	0.02	0.24	0.15	0.28
2116	Chuff	0.37	0.22	0.11	0.22	0.03	0.3	0.09	0.3
2117	Chuff	0.33	0.39	0.12	0.2	0.02	0.24	0.17	0.33
2118	Chuff	0.3	0.24	0.15	0.19	0.03	0.26	0.15	0.21
2119	Chuff	0.32	0.17	0.04	0.2	0.03	0.28	0.13	0.21
2120	Chuff	0.3	0.13	0.1	0.16	0.05	0.28	0.06	0.21
2121	Chuff	0.3	0.17	0.1	0.17	0.03	0.26	0.13	0.26
2122	Chuff	0.32	0.17	0.09	0.18	0.03	0.26	0.13	0.19
2123	Chuff	0.36	0.24	0.17	0.21	0.05	0.26	0.09	0.26
2124	Chuff	0.36	0.24	0.18	0.22	0.04	0.28	0.09	0.23
2125	Chuff	0.41	0.22	0.1	0.19	0.04	0.28	0.09	0.2
2126	Chuff	0.31	0.19	0.18	0.21	0.02	0.26	0.17	0.22
2127	Chuff	0.36	0.13	0.06	0.16	0.07	0.28	0.06	0.25
2128	Chuff	0.37	0.17	0.26	0.17	0.02	0.22	0.13	0.2
2129	Chuff	0.33	0.15	0.21	0.15	0.03	0.22	0.11	0.15
2130	Chuff	0.36	0.13	0.25	0.14	0.02	0.19	0.13	0.18
2131	Chuff	0.37	0.13	0.33	0.15	0.03	0.22	0.09	0.17
2132	Chuff	0.47	0.15	0.32	0.17	0.03	0.22	0.11	0.19
2133	Chuff	0.46	0.15	0.21	0.15	0.02	0.17	0.11	0.21
2134	Chuff	0.39	0.15	0.31	0.15	0.02	0.19	0.11	0.17
2135	Chuff	0.41	0.15	0.31	0.15	0.02	0.22	0.09	0.2
2136	Chuff	0.48	0.15	0.25	0.18	0.05	0.28	0.06	0.18
2137	Chuff	0.43	0.15	0.29	0.17	0.04	0.24	0.13	0.2
2138	Chuff	0.55	0.22	0.27	0.2	0.03	0.28	0.13	0.21
2139	Chuff	0.47	0.15	0.25	0.2	0.04	0.28	0.13	0.27
2140	Chuff	0.43	0.34	0.2	0.21	0.05	0.28	0.06	0.25
2141	Chuff	0.42	0.3	0.14	0.15	0.04	0.22	0.11	0.23
2142	Chuff	0.29	0.11	0.24	0.17	0.05	0.26	0.09	0.2
2143	Chuff	0.32	0.15	0.15	0.14	0.05	0.22	0.06	0.2
2144	Chuff	0.47	0.26	0.09	0.17	0.04	0.22	0.09	0.2
2145	Chuff	0.38	0.13	0.22	0.15	0.03	0.22	0.09	0.23
2146	Chuff	0.5	0.11	0.17	0.16	0.04	0.22	0.11	0.19
2147	Chuff	0.34	0.37	0.24	0.17	0.03	0.22	0.11	0.28
2148	Chuff	0.31	0.15	0.27	0.17	0.02	0.22	0.13	0.26
2149	Chuff	0.39	0.15	0.18	0.16	0.03	0.22	0.11	0.18
2150	Chuff	0.45	0.28	0.17	0.17	0.03	0.22	0.13	0.19
2151	Chuff	0.33	0.24	0.18	0.22	0.04	0.28	0.06	0.23
2152	Chuff	0.31	0.17	0.24	0.18	0.02	0.22	0.13	0.21
2153	Chuff	0.34	0.39	0.12	0.17	0.04	0.22	0.09	0.21
2154	Chuff	0.41	0.15	0.19	0.17	0.03	0.24	0.06	0.17
2155	Chuff	0.31	0.11	0.19	0.14	0.05	0.22	0.06	0.15
2156	Chuff	0.32	0.13	0.18	0.15	0.05	0.22	0.09	0.13
2157	Chuff	0.31	0.17	0.18	0.17	0.02	0.22	0.13	0.18
2158	Chuff	0.35	0.15	0.16	0.16	0.03	0.22	0.11	0.2
2159	Chuff	0.35	0.11	0.2	0.12	0.02	0.15	0.06	0.16

2160	Chuff	0.31	0.13	0.2	0.13	0.03	0.17	0.06	0.14
2161	Chuff	0.4	0.13	0.17	0.11	0.03	0.17	0.06	0.13
2162	Chuff	0.37	0.28	0.17	0.13	0.03	0.17	0.06	0.24
2163	Chuff	0.37	0.13	0.19	0.14	0.03	0.19	0.06	0.17
2164	Chuff	0.3	0.13	0.2	0.11	0.04	0.22	0.04	0.09
2165	Chuff	0.32	0.15	0.19	0.14	0.03	0.19	0.06	0.17
2166	Chuff	0.36	0.13	0.26	0.14	0.02	0.17	0.09	0.22
2167	Chuff	0.43	0.28	0.16	0.13	0.02	0.17	0.06	0.19
2168	Chuff	0.47	0.28	0.19	0.13	0.02	0.15	0.06	0.19
2169	Chuff	0.29	0.13	0.1	0.12	0.03	0.17	0.02	0.18
2170	Chuff	0.3	0.15	0.09	0.13	0.02	0.17	0.06	0.15
2171	Chuff	0.28	0.26	0.14	0.14	0.03	0.17	0.06	0.22
2172	Chuff	0.38	0.24	0.08	0.1	0.04	0.17	0.06	0.14
2173	Chuff	0.44	0.17	0.16	0.16	0.02	0.19	0.11	0.2
2174	Chuff	0.41	0.28	0.09	0.14	0.03	0.19	0.09	0.22
2175	Chuff	0.25	0.13	0.03	0.13	0.02	0.17	0.11	0.13
2176	Chuff	0.24	0.15	0.08	0.14	0.02	0.17	0.11	0.17
2177	Chuff	0.23	0.3	0.08	0.15	0.02	0.22	0.09	0.2
2178	Chuff	0.2	0.19	0.03	0.18	0.04	0.22	0.06	0.16
2179	Chuff	0.2	0.17	0.11	0.18	0.01	0.19	0.15	0.19
2180	Chuff	0.35	0.13	0.15	0.14	0.02	0.19	0.09	0.22
2181	Chuff	0.27	0.15	0.17	0.15	0.02	0.17	0.11	0.16
2182	Chuff	0.33	0.3	0.15	0.14	0.02	0.17	0.11	0.23
2183	Chuff	0.31	0.15	0.15	0.15	0.02	0.22	0.11	0.22
2184	Chuff	0.31	0.15	0.15	0.13	0.04	0.24	0.06	0.16
2185	Chuff	0.3	0.28	0.08	0.14	0.03	0.19	0.06	0.22
2186	Chuff	0.29	0.15	0.08	0.15	0.02	0.19	0.11	0.17
2187	Chuff	0.26	0.15	0.11	0.15	0.02	0.19	0.13	0.19
2188	Chuff	0.3	0.13	0.13	0.14	0.02	0.19	0.13	0.17
2189	Chuff	0.33	0.28	0.13	0.17	0.02	0.19	0.11	0.2
2190	Chuff	0.32	0.3	0.14	0.15	0.03	0.19	0.11	0.16
2191	Chuff	0.28	0.17	0.1	0.15	0.02	0.22	0.13	0.16
2192	Chuff	0.28	0.15	0.09	0.15	0.02	0.19	0.13	0.17
2193	Chuff	0.31	0.19	0.09	0.19	0.02	0.22	0.15	0.2
2194	Chuff	0.33	0.19	0.01	0.16	0.05	0.26	0.06	0.14
2195	Chuff	0.24	0.37	0.06	0.15	0.03	0.19	0.09	0.22
2196	Chuff	0.24	0.28	0.07	0.13	0.03	0.19	0.06	0.2
2197	Chuff	0.22	0.15	0.08	0.14	0.02	0.17	0.09	0.16
2198	Chuff	0.25	0.28	0.02	0.13	0.02	0.15	0.06	0.19
2199	Chuff	0.21	0.28	0.03	0.15	0.03	0.19	0.06	0.26
2200	Chuff	0.32	0.28	0.04	0.14	0.02	0.17	0.11	0.18
2201	Chuff	0.29	0.15	0.1	0.17	0.03	0.22	0.11	0.25
2202	Chuff	0.41	0.32	0.02	0.16	0.02	0.22	0.13	0.24
2203	Chuff	0.23	0.15	0.11	0.17	0.03	0.19	0.09	0.18
2204	Chuff	0.24	0.13	0.11	0.14	0.03	0.19	0.11	0.19
2205	Chuff	0.27	0.17	0.16	0.17	0.02	0.19	0.13	0.17
2206	Chuff	0.27	0.13	0.1	0.16	0.04	0.22	0.09	0.21
2207	Chuff	0.27	0.17	0.08	0.16	0.02	0.24	0.13	0.16
2208	Chuff	0.27	0.15	0.05	0.17	0.02	0.22	0.13	0.19
2209	Chuff	0.23	0.32	0.03	0.17	0.02	0.19	0.13	0.22
2210	Chuff	0.28	0.15	0.03	0.15	0.03	0.19	0.09	0.17
2211	Chuff	0.32	0.09	0.03	0.11	0.04	0.17	0.06	0.16
2212	Chuff	0.25	0.45	0.07	0.2	0.03	0.26	0.15	0.24
2213	Chuff	0.28	0.43	0.08	0.2	0.03	0.26	0.15	0.24

2214	Chuff	0.22	0.17	0.1	0.19	0.04	0.26	0.13	0.21
2215	Chuff	0.28	0.22	0.09	0.2	0.04	0.24	0.13	0.18
2216	Chuff	0.34	0.22	0.08	0.21	0.02	0.24	0.17	0.21
2217	Chuff	0.29	0.22	0.09	0.2	0.03	0.28	0.15	0.22
2218	Chuff	0.24	0.22	0.09	0.21	0.03	0.26	0.15	0.21
2219	Chuff	0.32	0.28	0.07	0.21	0.05	0.28	0.11	0.22
2220	Chuff	0.25	0.22	0.15	0.2	0.03	0.28	0.13	0.23
2221	Chuff	0.24	0.17	0.04	0.19	0.02	0.24	0.17	0.24
2222	Chuff	0.18	0.17	0.02	0.17	0.03	0.24	0.13	0.18
2223	Chuff	0.22	0.19	0.08	0.19	0.02	0.24	0.15	0.2
2224	Chuff	0.14	0.6	0.1	0.18	0.03	0.26	0.13	0.29
2225	Chuff	0.25	0.37	0.1	0.19	0.02	0.22	0.17	0.25
2226	Chuff	0.22	0.37	0.09	0.18	0.03	0.28	0.15	0.19
2227	Chuff	0.27	0.41	0.08	0.19	0.02	0.22	0.15	0.22
2228	Chuff	0.2	0.37	0.08	0.17	0.03	0.24	0.13	0.24
2229	Chuff	0.23	0.37	0.1	0.18	0.02	0.24	0.15	0.19
2230	Chuff	0.26	0.45	0.07	0.19	0.02	0.22	0.13	0.26
2231	Chuff	0.23	0.22	0.11	0.21	0.01	0.22	0.19	0.23
2232	Chuff	0.22	0.41	0.03	0.18	0.03	0.24	0.15	0.23
2233	Chuff	0.25	0.43	0.03	0.19	0.02	0.24	0.15	0.31
2234	Chuff	0.27	0.17	0.1	0.18	0.02	0.24	0.15	0.17
2235	Chuff	0.3	0.19	0.12	0.18	0.02	0.24	0.15	0.17
2236	Chuff	0.25	0.17	0.11	0.16	0.03	0.24	0.11	0.15
2237	Chuff	0.33	0.17	0.1	0.17	0.02	0.22	0.13	0.16
2238	Chuff	0.25	0.19	0.15	0.2	0.02	0.26	0.15	0.24
2239	Chuff	0.23	0.19	0.14	0.19	0.03	0.24	0.15	0.21
2240	Chuff	0.23	0.17	0.03	0.17	0.02	0.24	0.13	0.19
2241	Chuff	0.27	0.19	0.05	0.18	0.02	0.22	0.13	0.22
2242	Chuff	0.32	0.39	0.07	0.19	0.01	0.22	0.17	0.21
2243	Chuff	0.31	0.15	0.09	0.16	0.02	0.22	0.13	0.17
2244	Chuff	0.25	0.19	0.08	0.19	0.03	0.28	0.15	0.18
2245	Chuff	0.31	0.24	0.06	0.18	0.04	0.28	0.13	0.17
2246	Chuff	0.27	0.24	0.1	0.22	0.03	0.28	0.13	0.22
2247	Chuff	0.23	0.19	0.09	0.19	0.03	0.28	0.15	0.17
2248	Chuff	0.26	0.19	0.16	0.19	0.03	0.26	0.17	0.19
2249	Chuff	0.27	0.17	0.16	0.19	0.03	0.28	0.17	0.18
2250	Chuff	0.26	0.15	0.2	0.18	0.04	0.28	0.13	0.16
2251	Chuff	0.34	0.22	0.16	0.22	0.02	0.26	0.17	0.21
2252	Chuff	0.23	0.17	0.16	0.18	0.01	0.19	0.15	0.17
2253	Chuff	0.24	0.17	0.18	0.17	0.03	0.24	0.09	0.16
2254	Chuff	0.33	0.15	0.12	0.16	0.03	0.22	0.13	0.17
2255	Chuff	0.2	0.19	0.15	0.18	0.03	0.26	0.13	0.22
2256	Chuff	0.2	0.17	0.16	0.19	0.02	0.24	0.15	0.22
2257	Chuff	0.32	0.19	0.09	0.19	0.02	0.24	0.13	0.2
2258	Chuff	0.3	0.28	0.09	0.16	0.03	0.22	0.13	0.23
2259	Chuff	0.28	0.19	0.09	0.2	0.02	0.24	0.15	0.2
2260	Chuff	0.23	0.19	0.15	0.19	0.02	0.24	0.15	0.2
2261	Chuff	0.36	0.41	0.21	0.21	0.03	0.3	0.17	0.28
2262	Chuff	0.31	0.17	0.19	0.2	0.05	0.28	0.13	0.26
2263	Chuff	0.31	0.19	0.16	0.21	0.03	0.28	0.15	0.3
2264	Chuff	0.32	0.15	0.19	0.22	0.05	0.28	0.13	0.22
2265	Chuff	0.35	0.24	0.19	0.22	0.02	0.26	0.19	0.26
2266	Chuff	0.31	0.52	0.24	0.16	0.04	0.22	0.13	0.27
2267	Chuff	0.51	0.22	0.24	0.21	0.02	0.28	0.15	0.26

2268	Chuff	0.29	0.15	0.18	0.19	0.05	0.24	0.11	0.24
2269	Chuff	0.31	0.19	0.21	0.21	0.02	0.24	0.17	0.23
2270	Chuff	0.45	0.22	0.14	0.2	0.03	0.28	0.15	0.24
2271	Chuff	0.36	0.19	0.16	0.2	0.03	0.28	0.15	0.22
2272	Chuff	0.35	0.22	0.22	0.22	0.02	0.28	0.15	0.24
2273	Chuff	0.32	0.11	0.17	0.19	0.08	0.28	0.06	0.2
2274	Chuff	0.3	0.19	0.23	0.19	0.03	0.26	0.15	0.2
2275	Chuff	0.3	0.19	0.16	0.21	0.04	0.26	0.11	0.25
2276	Chuff	0.48	0.22	0.2	0.22	0.02	0.26	0.17	0.23
2277	Chuff	0.36	0.19	0.18	0.21	0.03	0.26	0.15	0.21
2278	Chuff	0.41	0.24	0.16	0.23	0.02	0.26	0.17	0.22
2279	Chuff	0.35	0.17	0.24	0.18	0.03	0.26	0.13	0.12
2280	Chuff	0.46	0.19	0.18	0.21	0.02	0.26	0.13	0.23
2281	Chuff	0.44	0.17	0.13	0.18	0.02	0.24	0.15	0.21
2282	Chuff	0.47	0.19	0.09	0.21	0.02	0.26	0.13	0.2
2283	Chuff	0.4	0.19	0.23	0.21	0.03	0.28	0.15	0.21
2284	Chuff	0.31	0.19	0.22	0.2	0.04	0.28	0.15	0.21
2285	Chuff	0.37	0.22	0.16	0.22	0.04	0.3	0.11	0.24
2286	Chuff	0.3	0.41	0.23	0.22	0.02	0.26	0.17	0.24
2287	Chuff	0.33	0.22	0.22	0.22	0.03	0.26	0.15	0.25
2288	Chuff	0.32	0.19	0.25	0.21	0.03	0.28	0.15	0.25
2289	Chuff	0.37	0.22	0.16	0.22	0.04	0.28	0.09	0.23
2290	Chuff	0.34	0.47	0.17	0.2	0.04	0.28	0.13	0.26
2291	Chuff	0.39	0.15	0.19	0.2	0.06	0.28	0.13	0.18
2292	Chuff	0.46	0.15	0.33	0.22	0.05	0.28	0.13	0.21
2293	Chuff	0.5	0.15	0.27	0.21	0.05	0.3	0.13	0.19
2294	Chuff	0.38	0.43	0.27	0.23	0.03	0.28	0.19	0.29
2295	Chuff	0.34	0.19	0.17	0.22	0.04	0.3	0.17	0.21
2296	Chuff	0.35	0.24	0.2	0.24	0.02	0.28	0.19	0.23
2297	Chuff	0.35	0.24	0.24	0.23	0.03	0.28	0.17	0.26
2298	Chuff	0.3	0.3	0.25	0.24	0.03	0.28	0.17	0.27
2299	Chuff	0.44	0.15	0.18	0.22	0.12	0.52	0.02	0.19
2300	Chuff	0.43	0.28	0.12	0.29	0.03	0.32	0.22	0.26
2301	Chuff	0.37	0.28	0.17	0.25	0.03	0.3	0.19	0.26
2302	Chuff	0.34	0.41	0.13	0.22	0.04	0.28	0.15	0.33
2303	Chuff	0.2	0.41	0.08	0.24	0.04	0.3	0.19	0.29
2304	Chuff	0.27	0.41	0.07	0.21	0.03	0.26	0.17	0.27
2305	Chuff	0.28	0.39	0.14	0.22	0.04	0.3	0.17	0.22
2306	Chuff	0.29	0.19	0.13	0.21	0.04	0.28	0.15	0.28
2307	Chuff	0.33	0.22	0.1	0.21	0.03	0.26	0.15	0.26
2308	Chuff	0.39	0.17	0.1	0.2	0.04	0.28	0.13	0.2
2309	Chuff	0.31	0.39	0.11	0.19	0.03	0.24	0.15	0.31
2310	Chuff	0.31	0.19	0.16	0.19	0.02	0.24	0.17	0.2
2311	Chuff	0.32	0.17	0.2	0.18	0.03	0.24	0.15	0.19
2312	Chuff	0.29	0.22	0.16	0.2	0.02	0.24	0.17	0.23
2313	Chuff	0.36	0.22	0.1	0.2	0.03	0.28	0.15	0.2
2314	Chuff	0.2	0.39	0.01	0.22	0.02	0.26	0.17	0.27
2315	Chuff	0.24	0.39	0.06	0.22	0.02	0.26	0.19	0.28
2316	Chuff	0.32	0.17	0.1	0.19	0.03	0.26	0.15	0.2
2317	Chuff	0.32	0.37	0.11	0.2	0.02	0.24	0.17	0.29
2318	Chuff	0.32	0.24	0.17	0.24	0.02	0.28	0.17	0.25
2319	Chuff	0.35	0.5	0.05	0.19	0.04	0.28	0.13	0.24
2320	Chuff	0.35	0.24	0.1	0.23	0.04	0.28	0.13	0.22
2321	Chuff	0.33	0.22	0.16	0.21	0.03	0.26	0.17	0.22

2322	Chuff	0.28	0.17	0.17	0.21	0.03	0.26	0.15	0.21
2323	Chuff	0.31	0.17	0.12	0.2	0.03	0.28	0.15	0.25
2324	Chuff	0.39	0.24	0.09	0.22	0.03	0.28	0.15	0.21
2325	Chuff	0.32	0.24	0.15	0.23	0.02	0.28	0.17	0.26
2326	Chuff	0.33	0.24	0.13	0.23	0.03	0.28	0.17	0.25
2327	Hiss	0.7	0.56	0.24	0	0	0	0	1.18
2328	Hiss	0.62	0.69	0.31	0	0	0	0	0.6
2329	Hiss	0.53	0.73	0.38	0	0	0	0	0.58
2330	Hiss	0.39	0.19	0.14	0	0	0	0	0.72
2331	Hiss	0.57	0.62	0.23	0	0	0	0	0.6
2332	Hiss	0.42	1.06	0.18	0	0	0	0	0.73
2333	Hiss	0.33	0.69	0.24	0	0	0	0	0.62
2334	Hiss	0.47	2.09	0.27	0	0	0	0	0.98
2335	Hiss	0.27	0.62	0.07	0	0	0	0	0.5
2336	Hiss	0.34	0.75	0.15	0	0	0	0	0.67
2337	Hiss	0.18	1.85	0.1	0	0	0	0	0.85
2338	Hiss	0.61	0.22	0.43	0	0	0	0	0.29
2339	Hiss	0.73	0.28	0.15	0	0	0	0	0.52
2340	Hiss	0.64	0.62	0.2	0	0	0	0	0.54
2341	Hiss	0.49	0.39	0.41	0	0	0	0	0.46
2342	Hiss	0.66	0.3	0.2	0	0	0	0	0.49
2343	Hiss	0.68	0.09	0	0	0	0	0	0.72
2344	Hiss	0.23	0.13	0.04	0	0	0	0	0.74
2345	Hiss	0.34	0.43	0.08	0	0	0	0	0.49
2346	Hiss	0.38	0.52	0.1	0	0	0	0	0.5
2347	Hiss	0.4	0.37	0.13	0	0	0	0	0.44
2348	Hiss	0.56	0.39	0.15	0	0	0	0	0.42
2349	Hiss	0.48	0.37	0.16	0	0	0	0	0.38
2350	Hiss	0.39	0.28	0.25	0	0	0	0	0.32
2351	Hiss	0.57	0.24	0.08	0	0	0	0	0.51
2352	Hiss	0.37	0.71	0.13	0	0	0	0	0.68
2353	Hiss	0.31	0.3	0.14	0	0	0	0	0.59
2354	Hiss	1.12	0.28	0.65	0	0	0	0	0.57
2355	Hiss	1.11	0.41	0.49	0	0	0	0	0.41
2356	Hiss	0.97	0.69	0.44	0	0	0	0	0.45
2357	Hiss	0.93	0.37	0.52	0	0	0	0	0.36
2358	Hiss	1.23	0.65	0.51	0	0	0	0	0.51
2359	Hiss	0.75	0.62	0.45	0	0	0	0	0.49
2360	Hiss	0.85	0.5	0.58	0	0	0	0	0.36
2361	Hiss	0.93	0.24	0.48	0	0	0	0	0.22
2362	Hiss	1.19	1.14	0.6	0	0	0	0	0.74
2363	Hiss	0.94	1.77	0.43	0	0	0	0	0.88
2364	Hiss	0.78	1.98	0.46	0	0	0	0	0.9
2365	Hiss	0.58	2.61	0.49	0	0	0	0	0.54
2366	Hiss	0.77	1.29	0.34	0	0	0	0	0.78
2367	Hiss	0.81	1.36	0.31	0	0	0	0	0.48
2368	Hiss	0.76	1.31	0.45	0	0	0	0	0.67
2369	Hiss	1.03	0.39	0.32	0	0	0	0	0.52
2370	Hiss	0.95	0.17	0.39	0	0	0	0	0.48
2371	Hiss	0.7	1.72	0.4	0	0	0	0	0.62
2372	Hiss	0.75	1.12	0.42	0	0	0	0	0.77
2373	Hiss	0.58	0.37	0.27	0	0	0	0	0.91
2374	Hiss	0.56	1.44	0.37	0	0	0	0	0.73
2375	Hiss	0.67	0.58	0.34	0	0	0	0	0.57

2376	Hiss	0.62	0.09	0.19	0	0	0	0	0.99
2377	Hiss	0.79	0.28	0.55	0	0	0	0	0.26
2378	Hiss	0.82	0.37	0.6	0	0	0	0	0.27
2379	Hiss	1.37	0.26	0.57	0	0	0	0	0.21
2380	Hiss	0.96	0.45	0.65	0	0	0	0	0.3
2381	Hiss	0.81	0.45	0.67	0	0	0	0	0.25
2382	Hiss	0.84	0.24	0.77	0	0	0	0	0.3
2383	Hiss	0.94	0.43	0.69	0	0	0	0	0.25
2384	Hiss	1.38	0.6	0.49	0	0	0	0	0.4
2385	Hiss	0.9	0.34	0.75	0	0	0	0	0.32
2386	Hiss	1.15	0.39	0.63	0	0	0	0	0.34
2387	Hiss	0.95	0.37	0.68	0	0	0	0	0.3
2388	Hiss	1.39	0.65	0.6	0	0	0	0	0.36
2389	Hiss	1.13	0.43	0.71	0	0	0	0	0.31
2390	Hiss	0.81	0.06	0.63	0	0	0	0	0.34
2391	Hiss	0.79	0.22	0.68	0	0	0	0	0.22
2392	Hiss	0.8	0.34	0.5	0	0	0	0	0.41
2393	Hiss	0.77	0.34	0.52	0	0	0	0	0.29
2394	Hiss	0.76	0.22	0.57	0	0	0	0	0.32
2395	Hiss	0.9	0.28	0.5	0	0	0	0	0.3
2396	Hiss	0.76	0.41	0.5	0	0	0	0	0.38
2397	Hiss	0.85	0.69	0.7	0	0	0	0	0.35
2398	Hiss	0.87	0.34	0.64	0	0	0	0	0.38
2399	Hiss	0.85	0.54	0.66	0	0	0	0	0.33
2400	Hiss	0.83	0.28	0.7	0	0	0	0	0.26
2401	Hiss	1.06	0.09	0.39	0	0	0	0	0.17
2402	Hiss	0.69	0.22	0.61	0	0	0	0	0.22
2403	Hiss	0.67	0.19	0.57	0	0	0	0	0.23
2404	Hiss	0.75	0.26	0.65	0	0	0	0	0.34
2405	Hiss	0.94	0.34	0.58	0	0	0	0	0.26
2406	Hiss	0.81	0.06	0.6	0	0	0	0	0.23
2407	Hiss	0.85	0.24	0.56	0	0	0	0	0.27
2408	Hiss	0.95	0.17	0.54	0	0	0	0	0.21
2409	Hiss	1.43	0.32	1.07	0	0	0	0	0.33
2410	Hiss	1.44	0.26	0.92	0	0	0	0	0.43
2411	Hiss	1.15	0.19	1.03	0	0	0	0	0.34
2412	Hiss	1.51	0.09	1.25	0	0	0	0	0.16
2413	Hiss	1.44	0.32	0.88	0	0	0	0	0.22
2414	Hiss	1.49	0.09	0.72	0	0	0	0	0.22
2415	Hiss	1.14	0.37	0.81	0	0	0	0	0.31
2416	Hiss	1.61	0.26	1.53	0	0	0	0	0.48
2417	Hiss	1.71	0.75	1.44	0	0	0	0	0.56
2418	Hiss	1.28	1.27	1.06	0	0	0	0	0.7
2419	Hiss	1.19	0.54	0.96	0	0	0	0	0.97
2420	Hiss	0.69	0.26	0.47	0	0	0	0	0.39
2421	Hiss	0.75	0.32	0.45	0	0	0	0	0.32
2422	Hiss	0.64	0.28	0.52	0	0	0	0	0.26
2423	Hiss	0.69	0.15	0.57	0	0	0	0	0.35
2424	Hiss	0.69	0.11	0.54	0	0	0	0	0.25
2425	Hiss	0.75	0.37	0.34	0	0	0	0	0.4
2426	Hiss	0.72	0.32	0.33	0	0	0	0	0.29
2427	Hiss	0.76	0.3	0.32	0	0	0	0	0.28
2428	Hiss	0.67	0.65	0.37	0	0	0	0	0.44
2429	Hiss	0.67	0.6	0.35	0	0	0	0	0.43

2430	Hiss	0.51	0.5	0.45	0	0	0	0	0.41
2431	Hiss	0.66	0.28	0.34	0	0	0	0	0.42
2432	Hiss	0.84	0.28	0.33	0	0	0	0	0.34
2433	Hiss	0.64	0.3	0.42	0	0	0	0	0.41
2434	Hiss	0.71	0.43	0.35	0	0	0	0	0.37
2435	Hiss	0.76	0.39	0.13	0	0	0	0	0.43
2436	Hiss	0.64	0.62	0.15	0	0	0	0	0.47
2437	Hiss	0.86	0.24	0.13	0	0	0	0	0.37
2438	Hiss	0.96	0.19	0.08	0	0	0	0	0.43
2439	Hiss	0.49	0.32	0.34	0	0	0	0	0.56
2440	Hiss	0.59	0.32	0.33	0	0	0	0	0.4
2441	Hiss	0.61	0.34	0.32	0	0	0	0	0.38
2442	Hiss	0.64	0.19	0.2	0	0	0	0	0.2
2443	Hiss	0.8	0.22	0.14	0	0	0	0	0.26
2444	Hiss	0.57	0.56	0.26	0	0	0	0	0.48
2445	Hiss	0.65	0.34	0.23	0	0	0	0	0.36
2446	Hiss	0.47	0.39	0.32	0	0	0	0	0.35
2447	Hiss	0.55	0.32	0.19	0	0	0	0	0.45
2448	Hiss	0.57	0.54	0.2	0	0	0	0	0.43
2449	Hiss	0.32	0.13	0.05	0	0	0	0	0.41
2450	Hiss	0.47	0.37	0.18	0	0	0	0	0.42
2451	Hiss	0.8	0.15	0.03	0	0	0	0	0.32
2452	Hiss	0.2	0.11	0.02	0	0	0	0	0.16
2453	Hiss	0.22	0.22	0.05	0	0	0	0	0.17
2454	Hiss	0.35	0.3	0.09	0	0	0	0	0.29
2455	Hiss	0.31	0.39	0.08	0	0	0	0	0.31
2456	Hiss	0.25	0.13	0.1	0	0	0	0	0.17
2457	Hiss	0.22	0.37	0.06	0	0	0	0	0.38
2458	Hiss	0.57	0.19	0.02	0	0	0	0	0.27
2459	Hiss	0.39	0.22	0.18	0	0	0	0	0.25
2460	Hiss	0.6	0.26	0.03	0	0	0	0	0.24
2461	Hiss	0.43	0.43	0.18	0	0	0	0	0.33
2462	Hiss	0.46	0.34	0.13	0	0	0	0	0.27
2463	Hiss	0.31	0.32	0.19	0	0	0	0	0.3
2464	Hiss	0.5	0.3	0.08	0	0	0	0	0.31
2465	Hiss	0.36	0.41	0.12	0	0	0	0	0.26
2466	Hiss	0.31	0.34	0.07	0	0	0	0	0.35
2467	Hiss	0.54	0.34	0.02	0	0	0	0	0.32
2468	Hiss	0.38	0.39	0.13	0	0	0	0	0.25
2469	Hiss	0.45	0.34	0.05	0	0	0	0	0.34
2470	Hiss	0.31	0.37	0.21	0	0	0	0	0.34
2471	Hiss	0.32	0.6	0.16	0	0	0	0	0.43
2472	Hiss	0.57	0.62	0.1	0	0	0	0	0.35
2473	Hiss	0.54	0.3	0.21	0	0	0	0	0.36
2474	Hiss	0.52	0.15	0.2	0	0	0	0	0.26
2475	Hiss	0.51	0.37	0.12	0	0	0	0	0.35
2476	Hiss	0.51	0.6	0.17	0	0	0	0	0.36
2477	Hiss	0.54	0.02	0.48	0	0	0	0	0.14
2478	Hiss	0.65	0.24	0.3	0	0	0	0	0.16
2479	Hiss	0.75	0.24	0.18	0	0	0	0	0.18
2480	Hiss	0.61	0.28	0.33	0	0	0	0	0.25
2481	Hiss	0.63	0.22	0.4	0	0	0	0	0.15
2482	Hiss	0.63	0.37	0.26	0	0	0	0	0.3
2483	Hiss	0.63	0.34	0.33	0	0	0	0	0.31

2484	Hiss	0.63	0.28	0.3	0	0	0	0	0.29
2485	Hiss	0.73	0.24	0.27	0	0	0	0	0.19
2486	Hiss	0.88	0.06	0.19	0	0	0	0	0.15
2487	Hiss	0.83	0.19	0.17	0	0	0	0	0.16
2488	Hiss	0.84	0.24	0.14	0	0	0	0	0.24
2489	Hiss	0.88	0.24	0.11	0	0	0	0	0.21
2490	Hiss	0.49	0.47	0.34	0	0	0	0	0.37
2491	Hiss	0.53	0.47	0.36	0	0	0	0	0.35
2492	Hiss	0.64	0.43	0.28	0	0	0	0	0.29
2493	Hiss	0.67	0.24	0.29	0	0	0	0	0.25
2494	Hiss	0.53	0.22	0.37	0	0	0	0	0.22
2495	Hiss	0.78	0.32	0.19	0	0	0	0	0.23
2496	Hiss	0.47	0.39	0.3	0	0	0	0	0.33
2497	Hiss	0.54	0.6	0.26	0	0	0	0	0.31
2498	Hiss	0.74	0.24	0.14	0	0	0	0	0.28
2499	Hiss	0.63	0.32	0.23	0	0	0	0	0.26
2500	Hiss	0.59	0.26	0.13	0	0	0	0	0.26
2501	Hiss	0.47	0.26	0.21	0	0	0	0	0.28
2502	Hiss	0.66	0.3	0.18	0	0	0	0	0.23
2503	Hiss	0.85	0.22	0.03	0	0	0	0	0.18
2504	Hiss	0.6	0.28	0.18	0	0	0	0	0.28
2505	Hiss	0.73	0.17	0.15	0	0	0	0	0.2
2506	Hiss	0.51	0.39	0.19	0	0	0	0	0.29
2507	Hiss	0.54	0.41	0.18	0	0	0	0	0.32
2508	Hiss	0.56	0.37	0.2	0	0	0	0	0.33
2509	Hiss	0.53	0.37	0.22	0	0	0	0	0.3
2510	Hiss	1.08	0.24	0.12	0	0	0	0	0.25
2511	Hiss	0.76	0.3	0.35	0	0	0	0	0.42
2512	Hiss	0.79	0.3	0.33	0	0	0	0	0.3
2513	Hiss	0.87	0.22	0.28	0	0	0	0	0.29
2514	Hiss	0.63	0.32	0.5	0	0	0	0	0.32
2515	Hiss	0.67	0.3	0.45	0	0	0	0	0.35
2516	Hiss	0.61	0.41	0.49	0	0	0	0	0.35
2517	Hiss	0.68	0.28	0.47	0	0	0	0	0.29
2518	Hiss	0.75	0.3	0.41	0	0	0	0	0.3
2519	Hiss	0.74	0.52	0.43	0	0	0	0	0.46
2520	Hiss	0.62	0.28	0.56	0	0	0	0	0.33
2521	Hiss	0.76	0.3	0.45	0	0	0	0	0.31
2522	Hiss	0.63	0.37	0.41	0	0	0	0	0.26
2523	Hiss	0.82	0.43	0.36	0	0	0	0	0.19
2524	Hiss	0.74	0.37	0.39	0	0	0	0	0.21
2525	Hiss	0.89	0.37	0.34	0	0	0	0	0.19
2526	Hiss	0.93	0.19	0.32	0	0	0	0	0.17
2527	Hiss	0.65	0.3	0.34	0	0	0	0	0.27
2528	Hiss	0.53	0.34	0.43	0	0	0	0	0.3
2529	Hiss	0.69	0.43	0.31	0	0	0	0	0.32
2530	Hiss	0.64	0.39	0.34	0	0	0	0	0.36
2531	Hiss	0.54	0.47	0.4	0	0	0	0	0.36
2532	Hiss	0.67	0.45	0.37	0	0	0	0	0.28
2533	Hiss	0.64	0.39	0.41	0	0	0	0	0.26
2534	Hiss	0.72	0.45	0.4	0	0	0	0	0.38
2535	Hiss	0.72	0.43	0.43	0	0	0	0	0.37
2536	Hiss	0.6	0.34	0.47	0	0	0	0	0.33
2537	Hiss	0.61	0.32	0.46	0	0	0	0	0.27

2538	Ar2	1.4	0.17	0.08	0.16	0.04	0.24	0.11	0.12
2539	Haer	1.15	0.19	0.2	0.14	0.05	0.26	0.06	0.14
2540	Roar	0.93	0.24	0.27	0.16	0.03	0.19	0.11	0.25
2541	Ar2	1.27	0.75	0.05	0.15	0.02	0.19	0.09	0.37
2542	Ar2	1.27	0.28	0.1	0.17	0.02	0.19	0.11	0.25
2543	Ar2	0.61	0.43	0.47	0.14	0.02	0.17	0.09	0.41
2544	Haer	0.96	0.67	0.23	0.11	0.04	0.19	0.06	0.28
3111	Er	0.15	0.26	0.13	0.13	0.02	0.15	0.09	0.25
2546	Ar2	0.89	1.01	0.25	0.17	0.03	0.24	0.11	0.26
2547	Ar2	0.56	0.43	0.45	0.15	0.02	0.22	0.13	0.42
2548	Ar2	1.06	0.19	0.24	0.16	0.03	0.22	0.11	0.15
2549	Roar	1.13	0.56	0.17	0.19	0.02	0.22	0.11	0.24
2550	Haer	0.81	0.17	0.24	0.17	0.03	0.24	0.06	0.22
2551	Ar2	0.79	0.13	0.3	0.15	0.03	0.22	0.11	0.24
2552	Ar2	1.15	0.86	0.06	0.15	0.04	0.19	0.09	0.2
2553	Ar2	0.87	0.75	0.47	0.16	0.03	0.19	0.11	0.3
2554	Ar2	0.74	0.15	0.59	0.16	0.05	0.22	0.06	0.19
3112	Er	0.2	0.5	0.1	0.11	0.02	0.17	0.09	0.21
2556	Ar2	0.7	0.47	0.62	0.16	0.02	0.22	0.13	0.29
2557	Er	0.75	0.47	0.52	0.17	0.02	0.19	0.11	0.33
2558	Ar2	0.74	0.54	0.47	0.16	0.03	0.19	0.09	0.27
2559	Ar2	0.59	0.13	0.55	0.16	0.03	0.19	0.11	0.39
2560	Ar2	0.78	0.3	0.46	0.17	0.03	0.22	0.11	0.25
2561	Ar2	1.18	0.86	0.08	0.15	0.03	0.22	0.09	0.4
2562	Ar2	0.98	0.84	0.31	0.18	0.02	0.22	0.11	0.36
2563	Ar2	0.74	0.11	0.47	0.12	0.02	0.15	0.09	0.23
2564	Ar2	0.96	0.65	0.3	0.12	0.01	0.13	0.09	0.3
2565	Er	0.96	0.65	0.3	0.12	0.01	0.13	0.09	0.3
3113	Er	0.17	0.3	0.12	0.13	0.02	0.15	0.09	0.3
2567	Ar2	0.93	0.69	0.21	0.12	0.01	0.13	0.09	0.51
3115	Er	0.13	0.32	0.08	0.13	0.02	0.15	0.09	0.3
3116	Er	0.25	0.41	0.044062	0.13	0.01	0.15	0.11	0.24
2570	Ar2	0.74	0.37	0.45	0.14	0.02	0.17	0.06	0.26
2571	Ar2	1.53	0.28	0.15	0.17	0.03	0.19	0.11	0.22
2572	Ar2	1.29	0.69	0.26	0.12	0.03	0.22	0.06	0.47
2573	Haer	1.35	0.65	0.22	0.12	0.03	0.17	0.06	0.36
3117	Er	0.13	0.24	0.1	0.13	0.02	0.15	0.11	0.22
2575	Ar2	1.25	0.41	0.23	0.14	0.02	0.17	0.09	0.26
2576	Ar2	1.02	0.17	0.41	0.15	0.02	0.17	0.11	0.29
3121	Er	0.28	0.62	0.06	0.12	0.01	0.13	0.09	0.27
2578	Ar2	0.93	0.15	0.47	0.14	0.02	0.15	0.09	0.29
2579	Er	0.97	0.15	0.32	0.16	0.03	0.19	0.06	0.17
2580	Ar2	1.18	0.32	0.21	0.15	0.03	0.17	0.09	0.23
2581	Haer	0.91	0.47	0.39	0.15	0.03	0.22	0.06	0.23
2582	Er	1.4	0.82	0.06	0.14	0.03	0.17	0.09	0.33
2583	Ar2	1.54	0.22	0.1	0.13	0.02	0.15	0.09	0.17
2584	Ar2	1.45	0.26	0.13	0.14	0.02	0.17	0.09	0.21
2585	Ar2	1.79	0.15	0.04	0.14	0.02	0.17	0.09	0.34
2586	Ar2	1.21	0.39	0.32	0.14	0.03	0.19	0.06	0.29
2587	Ar2	1.13	0.84	0.29	0.14	0.02	0.17	0.06	0.77
2588	Ar2	0.89	0.88	0.47	0.14	0.03	0.17	0.09	0.37
2589	Ar2	1.54	1.1	0.08	0.13	0.02	0.17	0.11	0.27
2590	Ar2	1.15	1.36	0.32	0.15	0.02	0.17	0.09	0.26
2591	Ar2	0.97	0.41	0.53	0.14	0.03	0.19	0.09	0.49

2592	Ar2	0.72	0.45	0.66	0.13	0.03	0.17	0.06	0.36
2593	Ar2	0.98	0.15	0.34	0.16	0.03	0.24	0.11	0.21
2594	Ar2	0.79	0.73	0.41	0.15	0.04	0.22	0.06	0.59
2595	Haer	0.76	0.52	0.53	0.14	0.04	0.19	0.09	0.27
2596	Ar2	0.69	0.39	0.52	0.15	0.03	0.19	0.11	0.37
2597	Ar2	0.91	0.19	0.51	0.16	0.04	0.22	0.09	0.28
2598	Ar2	1.16	0.67	0.32	0.15	0.03	0.19	0.09	0.5
2599	Ar2	1.22	0.75	0.23	0.19	0.03	0.24	0.09	0.38
2600	Ar2	1.51	0.78	0.14	0.17	0.04	0.24	0.09	0.42
2601	Ar2	1.75	0.22	0.05	0.17	0.02	0.19	0.11	0.39
2602	Ar2	1.54	0.69	0.13	0.16	0.03	0.19	0.09	0.44
2603	Ar2	1.6	0.45	0.12	0.18	0.02	0.26	0.11	0.4
2604	Ar2	1.48	0.15	0.17	0.17	0.02	0.22	0.13	0.34
2605	Ar2	0.66	1.06	0.36	0.13	0.01	0.17	0.11	0.19
2606	Ar2	1.03	0.75	0.12	0.12	0.02	0.15	0.09	0.45
2607	Ar2	0.58	0.13	0.42	0.14	0.03	0.19	0.06	0.26
3122	Er	0.16	0.15	0.1	0.12	0.02	0.15	0.09	0.2
2609	Ar2	1.01	0.84	0.13	0.13	0.03	0.19	0.06	0.28
2610	Ar2	1.07	0.32	0.19	0.15	0.02	0.19	0.13	0.29
2611	Ar2	0.61	0.15	0.45	0.14	0.02	0.22	0.09	0.31
2612	Ar2	1.17	0.73	0.05	0.14	0.02	0.19	0.09	0.4
3123	Er	0.31	0.26	0.032313	0.12	0.01	0.13	0.11	0.24
2614	Ar2	0.66	0.71	0.37	0.13	0.02	0.15	0.09	0.58
2615	Ar2	0.93	0.95	0.3	0.16	0.02	0.19	0.11	0.38
2616	Ar2	0.79	0.88	0.31	0.14	0.03	0.17	0.09	0.64
2617	Ar2	0.57	0.39	0.5	0.14	0.02	0.17	0.11	0.68
2618	Ar2	0.83	0.78	0.28	0.17	0.02	0.22	0.11	0.49
2619	Ar2	1.05	0.78	0.23	0.18	0.02	0.22	0.11	0.4
2620	Ar2	0.41	1.03	0.17	0.17	0.03	0.22	0.13	0.7
2621	Ar2	0.41	1.08	0.21	0.18	0.02	0.24	0.15	0.59
2622	Ar2	0.34	0.99	0.18	0.18	0.02	0.22	0.11	0.7
3124	Er	0.1	0.5	0.06	0.13	0.01	0.13	0.11	0.3
2624	Ar2	0.35	1.12	0.19	0.2	0.02	0.24	0.15	0.35
3125	Er	0.18	0.58	0.04	0.11	0.01	0.13	0.11	0.2
2626	Roar	0.41	0.78	0.23	0.17	0.05	0.24	0.09	0.46
3126	Er	0.1	0.54	0.06	0.1	0.02	0.13	0.09	0.37
2628	Roar	0.44	0.73	0.2	0.19	0.03	0.26	0.09	0.58
3127	Er	0.1	0.5	0.06	0.11	0.02	0.13	0.09	0.3
2630	Ar2	0.56	0.8	0.06	0.16	0.05	0.24	0.09	0.42
2631	Haer	0.35	0.13	0.21	0.18	0.05	0.28	0.09	0.47
2632	Ar2	0.33	0.6	0.17	0.18	0.04	0.24	0.11	0.44
2633	Ar2	0.57	0.19	0.17	0.18	0.04	0.26	0.11	0.32
2634	Ar2	0.7	0.22	0.09	0.2	0.05	0.26	0.13	0.29
2635	Roar	0.45	0.93	0.12	0.15	0.04	0.22	0.06	0.19
2636	Ar2	0.44	0.82	0.17	0.14	0.02	0.19	0.11	0.58
2637	Roar	0.64	0.88	0.14	0.16	0.03	0.22	0.09	0.34
2638	Ar2	0.5	0.69	0.22	0.16	0.02	0.19	0.11	0.58
2639	Roar	0.66	0.9	0.11	0.16	0.03	0.22	0.09	0.49
2640	Ar2	0.41	0.41	0.31	0.16	0.02	0.22	0.15	0.53
3129	Er	0.18	0.28	0.04	0.14	0.01	0.17	0.13	0.3
2642	Ar2	0.67	0.22	0.19	0.21	0.03	0.28	0.09	0.24
2643	Ar2	0.46	0.6	0.36	0.17	0.03	0.26	0.11	0.44
2644	Roar	0.67	0.65	0.2	0.16	0.05	0.24	0.06	0.46
2645	Ar2	0.54	0.9	0.24	0.17	0.04	0.24	0.09	0.66

2646	Ar2	0.64	0.86	0.17	0.18	0.03	0.24	0.11	0.44
2647	Ar2	0.77	0.75	0.1	0.18	0.03	0.22	0.13	0.59
2648	Haer	0.66	0.26	0.28	0.19	0.03	0.24	0.11	0.28
2649	Ar2	0.91	0.17	0.09	0.15	0.04	0.24	0.09	0.32
2650	Ar2	1	1.03	0.09	0.14	0.03	0.17	0.11	0.45
2651	Er	1.13	1.06	0.03	0.18	0.05	0.24	0.06	0.2
2652	Ar2	0.5	1.03	0.18	0.15	0.04	0.22	0.09	0.47
2653	Ar2	0.1	0.34	0.53	0.17	0.03	0.24	0.09	0.45
2654	Ar2	0.48	1.1	0.34	0.15	0.02	0.19	0.11	0.35
2655	Ar2	0.55	0.97	0.3	0.15	0.02	0.19	0.11	0.75
2656	Ar2	0.79	1.08	0.2	0.14	0.02	0.17	0.09	0.33
2657	Ar2	0.77	0.82	0.17	0.14	0.03	0.17	0.06	0.49
2658	Ar2	0.73	0.32	0.3	0.16	0.01	0.17	0.13	0.31
2659	Ar2	0.55	0.99	0.29	0.1	0.02	0.13	0.09	0.56
2660	Ar2	0.87	0.09	0.07	0.17	0.03	0.22	0.06	0.24
2661	Ar2	0.92	0.13	0.06	0.14	0.02	0.17	0.09	0.37
2662	Ar2	0.65	0.8	0.15	0.12	0.02	0.15	0.09	0.36
2663	Ar2	0.46	0.84	0.23	0.13	0.02	0.17	0.09	0.46
2664	Ar2	0.87	0.34	0.11	0.14	0.02	0.15	0.09	0.28
3131	Er	0.11	0.52	0.04	0.14	0.02	0.17	0.11	0.33
2666	Ar2	0.79	0.78	0.07	0.11	0.03	0.17	0.04	0.39
2667	Ar2	0.44	0.3	0.28	0.17	0.02	0.22	0.13	0.35
2668	Ar2	0.61	1.27	0.11	0.15	0.02	0.19	0.13	0.39
2669	Ar2	0.75	0.24	0.06	0.16	0.03	0.22	0.11	0.3
2670	Ar2	0.45	0.15	0.26	0.15	0.02	0.22	0.11	0.26
3132	Er	0.14	0.58	0.03	0.15	0.01	0.17	0.13	0.29
3134	Er	0.14	0.62	0.05	0.15	0.01	0.15	0.13	0.31
2673	Ar2	0.77	0.13	0.09	0.16	0.03	0.22	0.11	0.32
2674	Ar2	0.55	0.15	0.2	0.15	0.03	0.19	0.09	0.23
2675	Haer	0.69	1.68	0.16	0.14	0.03	0.17	0.09	0.19
2676	Ar2	0.62	1.14	0.16	0.13	0.03	0.19	0.09	0.34
2677	Ar2	0.44	0.13	0.31	0.14	0.02	0.19	0.11	0.34
2678	Ar2	0.73	1.27	0.13	0.14	0.02	0.17	0.06	0.41
2679	Ar2	0.56	0.86	0.2	0.12	0.02	0.15	0.09	0.5
2680	Ar2	0.35	0.47	0.29	0.15	0.02	0.19	0.09	0.6
2681	Ar2	0.44	1.08	0.18	0.14	0.03	0.19	0.06	0.63
2682	Ar2	0.37	0.47	0.31	0.16	0.01	0.17	0.15	0.51
2683	Ar2	0.38	0.97	0.26	0.16	0.01	0.17	0.13	0.86
2684	Ar2	0.6	1.27	0.14	0.17	0.02	0.22	0.11	0.34
2685	Ar2	0.32	0.86	0.25	0.14	0.02	0.17	0.11	0.82
2686	Ar2	0.47	1.18	0.16	0.15	0.02	0.19	0.11	0.64
2687	Er	0.52	0.65	0.16	0.14	0.02	0.15	0.09	0.37
2688	Ar2	0.44	0.86	0.16	0.14	0.03	0.22	0.06	0.2
2689	Ar2	0.61	0.65	0.11	0.15	0.02	0.17	0.09	0.37
3135	Er	0.08	0.6	0.06	0.14	0.01	0.15	0.13	0.32
2691	Roar	0.33	0.65	0.18	0.14	0.02	0.17	0.09	0.54
2692	Roar	0.4	0.6	0.18	0.13	0.03	0.17	0.09	0.49
3136	Er	0.12	0.28	0.08	0.12	0.04	0.15	0.06	0.3
3137	Er	0.14	0.56	0.09	0.13	0.02	0.15	0.09	0.33
3138	Er	0.27	0.22	0.031073	0.14	0.03	0.19	0.11	0.23
2696	Ar2	0.52	0.15	0.21	0.15	0.04	0.22	0.09	0.19
2697	Ar2	0.75	0.75	0.07	0.14	0.02	0.17	0.11	0.31
3139	Er	0.14	0.45	0.08	0.14	0.04	0.19	0.06	0.31
2699	Er	0.48	0.6	0.24	0.14	0.03	0.19	0.09	0.36

3140	Er	0.21	0.58	0.05	0.14	0.01	0.15	0.13	0.37
2701	Ar2	0.55	0.58	0.19	0.14	0.02	0.17	0.11	0.48
2702	Roar	0.4	0.43	0.22	0.15	0.03	0.19	0.11	0.27
2703	Ar2	0.37	0.15	0.28	0.14	0.05	0.26	0.06	0.14
3141	Er	0.06	0.22	0.04	0.11	0	0.11	0.11	0.22
2705	Ar2	0.58	0.37	0.17	0.12	0.02	0.15	0.09	0.29
2706	Haer	0.66	0.67	0.08	0.1	0.03	0.15	0.06	0.28
2707	Ar2	0.75	0.78	0.08	0.11	0.03	0.19	0.06	0.3
2708	Er	0.61	0.73	0.22	0.12	0.02	0.17	0.09	0.34
2709	Haer	0.54	0.67	0.26	0.11	0.03	0.17	0.06	0.34
2710	Er	0.38	0.43	0.36	0.15	0.01	0.17	0.13	0.23
2711	Haer	0.56	0.65	0.17	0.12	0.03	0.19	0.06	0.39
2712	Haer	0.53	0.13	0.25	0.14	0.04	0.22	0.06	0.25
2713	Er	0.47	0.97	0.2	0.13	0.02	0.17	0.09	0.38
2714	Ar2	0.89	0.15	0.1	0.15	0.03	0.19	0.09	0.16
2715	Ar2	0.63	0.82	0.22	0.13	0.02	0.17	0.06	0.23
2716	Ar2	0.55	0.11	0.36	0.11	0.03	0.17	0.06	0.16
2717	Ar2	0.56	0.58	0.3	0.13	0.03	0.17	0.09	0.35
2718	Haer	0.57	0.54	0.25	0.14	0.03	0.19	0.06	0.32
3142	Er	0.08	0.19	0.04	0.1	0.01	0.11	0.09	0.21
2720	Er	0.61	0.71	0.31	0.15	0.02	0.17	0.09	0.43
3143	Er	0.14	0.11	0.05	0.11	0.01	0.15	0.09	0.15
2722	Ar2	0.6	0.43	0.34	0.12	0.02	0.15	0.09	0.38
2723	Ar2	1.23	0.22	0.05	0.19	0.04	0.26	0.11	0.18
2724	Ar2	1.12	0.19	0.12	0.18	0.04	0.28	0.11	0.18
2725	Ar2	0.7	0.17	0.26	0.2	0.04	0.24	0.11	0.25
2726	Ar2	0.89	0.19	0.21	0.17	0.04	0.26	0.11	0.28
2727	Er	0.92	0.09	0.14	0.18	0.05	0.28	0.06	0.21
2728	Er	0.85	0.75	0.11	0.21	0.02	0.24	0.15	0.26
2729	Ar2	0.81	0.22	0.11	0.17	0.05	0.26	0.09	0.15
2730	Ar2	1.01	0.19	0.07	0.2	0.02	0.24	0.17	0.24
2731	Ar2	0.68	0.24	0.25	0.2	0.04	0.28	0.09	0.2
2732	Ar2	0.82	0.15	0.13	0.15	0.04	0.26	0.09	0.14
2733	Ar2	0.7	0.34	0.23	0.17	0.02	0.22	0.13	0.31
2734	Haer	0.76	0.11	0.23	0.15	0.04	0.24	0.09	0.17
3144	Er	0.09	0.34	0.05	0.11	0	0.11	0.11	0.23
2736	Ar2	1	0.26	0.07	0.18	0.03	0.22	0.11	0.22
2737	Ar2	0.61	0.17	0.35	0.18	0.03	0.22	0.11	0.24
2738	Ar2	0.6	0.75	0.34	0.17	0.02	0.19	0.11	0.43
2739	Haer	0.78	0.24	0.27	0.17	0.05	0.24	0.09	0.19
2740	Ar2	0.94	0.19	0.22	0.18	0.03	0.22	0.11	0.29
2741	Ar2	0.69	0.15	0.42	0.17	0.02	0.22	0.13	0.35
2742	Ar2	0.76	0.78	0.21	0.16	0.04	0.24	0.09	0.4
2743	Roar	0.63	0.62	0.27	0.16	0.04	0.22	0.09	0.45
2744	Ar2	0.67	0.22	0.28	0.17	0.03	0.26	0.13	0.2
2745	Ar2	0.47	0.17	0.43	0.18	0.03	0.26	0.13	0.19
2746	Ar2	0.95	0.15	0.17	0.15	0.03	0.24	0.09	0.2
2747	Haer	0.63	0.15	0.29	0.16	0.04	0.22	0.11	0.18
2748	Ar2	0.71	0.32	0.22	0.18	0.03	0.19	0.11	0.28
2749	Ar2	0.66	0.45	0.26	0.18	0.04	0.24	0.09	0.53
2750	Ar2	0.93	0.15	0.24	0.18	0.03	0.26	0.15	0.32
2751	Ar2	0.72	0.86	0.33	0.18	0.03	0.22	0.13	0.36
2752	Ar2	0.83	1.1	0.24	0.18	0.04	0.26	0.13	0.37
2753	Ar2	0.63	0.62	0.344731	0.19	0.04	0.3	0.11	0.37

2754	Ar2	0.49	0.15	0.42	0.18	0.06	0.3	0.11	0.24
2755	Ar2	0.64	0.22	0.34	0.21	0.03	0.26	0.15	0.25
2756	Ar2	0.61	0.84	0.29	0.2	0.03	0.24	0.15	0.57
2757	Er	1.08	0.15	0.2	0.18	0.04	0.26	0.13	0.27
2758	Haer	1.09	0.15	0.25	0.2	0.06	0.3	0.09	0.21
2759	Ar2	0.92	0.62	0.35	0.2	0.03	0.24	0.13	0.39
2760	Ar2	1.03	1.08	0.13	0.21	0.05	0.28	0.11	0.22
3145	Er	0.13	0.26	0.08	0.12	0.01	0.13	0.09	0.23
3146	Er	0.15	0.37	0.070509	0.11	0.02	0.13	0.06	0.16
2763	Haer	0.63	0.26	0.2	0.15	0.04	0.19	0.06	0.15
2764	Haer	0.57	0.22	0.27	0.15	0.04	0.19	0.06	0.17
2765	Haer	0.75	0.22	0.17	0.14	0.04	0.17	0.09	0.14
2766	Er	0.69	0.17	0.2	0.13	0.04	0.19	0.06	0.12
2767	Er	1	0.15	0.06	0.15	0.02	0.19	0.11	0.19
2768	Ar2	0.62	0.24	0.26	0.14	0.03	0.19	0.09	0.16
2769	Er	0.77	0.11	0.2	0.14	0.03	0.19	0.09	0.16
2770	Er	0.92	0.15	0.1	0.15	0.03	0.22	0.06	0.13
2771	Er	0.87	0.15	0.18	0.15	0.02	0.19	0.11	0.19
2772	Er	0.48	0.28	0.39	0.16	0.03	0.19	0.09	0.16
2773	Er	1.09	0.15	0.05	0.16	0.03	0.22	0.09	0.16
2774	Ar2	0.77	0.13	0.2	0.15	0.04	0.24	0.06	0.15
2775	Ar2	1.12	0.19	0.05	0.16	0.02	0.19	0.09	0.18
2776	Er	1.04	0.19	0.11	0.15	0.04	0.19	0.06	0.14
2777	Er	0.6	0.13	0.18	0.15	0.03	0.19	0.06	0.17
2778	Haer	0.64	0.24	0.13	0.14	0.03	0.17	0.06	0.15
2779	Haer	0.57	0.24	0.23	0.14	0.03	0.17	0.09	0.16
2780	Ar2	0.49	0.28	0.31	0.14	0.02	0.17	0.11	0.25
2781	Haer	0.73	0.22	0.14	0.13	0.04	0.17	0.06	0.15
2782	Er	0.93	0.17	0.03	0.15	0.03	0.19	0.09	0.14
2783	Haer	0.73	0.24	0.14	0.15	0.04	0.19	0.06	0.13
2784	Er	1.02	0.13	0.06	0.15	0.02	0.19	0.09	0.12
2785	Er	0.69	0.13	0.27	0.14	0.02	0.19	0.09	0.12
2786	Haer	0.45	0.15	0.25	0.14	0.04	0.19	0.06	0.11
2787	Er	0.68	0.11	0.19	0.13	0.03	0.24	0.09	0.11
2788	Er	0.82	0.11	0.13	0.13	0.02	0.22	0.11	0.13
2789	Haer	0.51	0.45	0.26	0.14	0.04	0.22	0.06	0.19
3147	Er	0.12	0.19	0.05	0.11	0.01	0.13	0.09	0.19
2791	Er	0.41	0.47	0.38	0.16	0.01	0.17	0.15	0.28
2792	Haer	0.54	0.43	0.21	0.15	0.03	0.24	0.09	0.2
2793	Ar2	0.5	0.15	0.29	0.15	0.01	0.22	0.13	0.17
2794	Ar2	0.41	0.37	0.25	0.17	0.02	0.22	0.13	0.24
2795	Ar2	0.36	0.47	0.25	0.15	0.02	0.22	0.13	0.2
2796	Haer	0.75	0.43	0.12	0.15	0.03	0.22	0.09	0.2
2797	Ar2	0.8	0.37	0.08	0.14	0.03	0.19	0.11	0.22
2798	Ar2	0.8	0.13	0.07	0.15	0.02	0.19	0.13	0.22
2799	Haer	0.71	0.19	0.11	0.16	0.03	0.22	0.11	0.17
2800	Haer	1.12	0.19	0.06	0.15	0.05	0.26	0.04	0.14
2801	Ar2	1.1	0.17	0.09	0.18	0.02	0.24	0.13	0.18
2802	Haer	0.82	0.22	0.23	0.15	0.05	0.26	0.06	0.12
2803	Ar2	0.45	0.13	0.42	0.15	0.02	0.19	0.13	0.23
2804	Ar2	0.62	0.47	0.3	0.15	0.03	0.22	0.11	0.22
2805	Er	0.99	0.19	0.06	0.16	0.04	0.22	0.06	0.21
2806	Ar2	0.91	0.17	0.15	0.17	0.02	0.22	0.13	0.21
2807	Haer	0.68	0.34	0.28	0.14	0.04	0.22	0.09	0.14

2808	Ar2	0.87	0.19	0.14	0.19	0.03	0.24	0.11	0.19
2809	Haer	0.9	0.26	0.07	0.15	0.04	0.22	0.11	0.15
2810	Haer	0.67	0.22	0.18	0.19	0.04	0.26	0.09	0.15
2811	Ar2	0.59	0.17	0.23	0.19	0.03	0.24	0.11	0.19
2812	Ar2	0.77	0.13	0.11	0.15	0.04	0.24	0.09	0.13
2813	Haer	0.61	0.19	0.15	0.17	0.05	0.24	0.06	0.15
2814	Er	0.56	0.17	0.24	0.16	0.03	0.22	0.09	0.15
2815	Ar2	0.75	0.15	0.12	0.16	0.03	0.22	0.09	0.18
2816	Haer	0.62	0.15	0.19	0.16	0.02	0.22	0.11	0.15
2817	Haer	0.62	0.24	0.21	0.15	0.04	0.19	0.09	0.16
2818	Er	0.47	0.28	0.34	0.16	0.03	0.19	0.09	0.26
2819	Er	0.52	0.34	0.28	0.17	0.02	0.19	0.11	0.3
2820	Er	0.62	0.15	0.27	0.15	0.03	0.22	0.11	0.16
2821	Haer	0.72	0.22	0.21	0.16	0.04	0.19	0.09	0.16
2822	Haer	0.59	0.22	0.14	0.16	0.04	0.19	0.06	0.17
2823	Er	0.65	0.15	0.12	0.14	0.02	0.19	0.11	0.18
2824	Ar2	0.43	0.09	0.22	0.12	0.05	0.19	0.06	0.13
2825	Haer	0.66	0.32	0.09	0.16	0.03	0.22	0.09	0.16
2826	Ar2	0.49	0.15	0.23	0.16	0.01	0.17	0.15	0.15
2827	Er	0.44	0.13	0.11	0.13	0.03	0.22	0.09	0.12
2828	Er	0.38	0.28	0.16	0.14	0.02	0.17	0.11	0.22
2829	Er	0.58	0.13	0.08	0.14	0.03	0.22	0.09	0.14
2830	Haer	0.58	0.15	0.07	0.15	0.04	0.22	0.06	0.13
2831	Ar2	0.56	0.26	0.17	0.17	0.02	0.19	0.13	0.17
2832	Er	0.6	0.15	0.16	0.15	0.02	0.26	0.13	0.15
2833	Haer	0.57	0.26	0.12	0.16	0.03	0.19	0.11	0.17
2834	Haer	0.57	0.22	0.14	0.19	0.04	0.24	0.09	0.17
2835	Haer	0.48	0.17	0.2	0.17	0.04	0.26	0.09	0.13
2836	Ar2	0.52	0.26	0.1	0.16	0.02	0.19	0.13	0.18
2837	Ar2	0.39	0.28	0.18	0.17	0.02	0.22	0.13	0.19
2838	Ar2	0.65	0.24	0.08	0.17	0.03	0.22	0.11	0.17
2839	Ar2	0.47	0.15	0.13	0.16	0.03	0.22	0.11	0.15
2840	Roar	0.43	0.17	0.18	0.15	0.04	0.22	0.09	0.15
2841	Ar2	0.56	0.26	0.1	0.17	0.02	0.19	0.13	0.28
2842	Er	0.68	0.19	0.06	0.16	0.04	0.26	0.09	0.16
2843	Ar2	0.46	0.22	0.18	0.2	0.02	0.22	0.13	0.18
2844	Haer	0.5	0.17	0.14	0.15	0.04	0.26	0.09	0.13
2845	Er	0.31	0.09	0.27	0.1	0.02	0.15	0.06	0.14
2846	Er	0.41	0.3	0.19	0.11	0.01	0.13	0.09	0.19
2847	Er	0.54	0.22	0.18	0.1	0.02	0.13	0.06	0.15
2848	Haer	0.46	0.22	0.18	0.1	0.03	0.13	0.04	0.11
2849	Haer	0.42	0.11	0.21	0.11	0.02	0.13	0.06	0.12
2850	Haer	0.4	0.17	0.24	0.12	0.03	0.15	0.06	0.13
2851	Haer	0.51	0.13	0.14	0.12	0.03	0.15	0.06	0.11
2852	Er	0.67	0.13	0.05	0.13	0.03	0.19	0.09	0.12
2853	Er	0.33	0.13	0.22	0.13	0.03	0.17	0.09	0.12
2854	Er	0.83	0.17	0.04	0.12	0.03	0.17	0.06	0.14
2855	Ar2	0.56	0.09	0.21	0.1	0.04	0.19	0.04	0.1
2856	Ar2	0.64	0.26	0.19	0.13	0.01	0.13	0.09	0.23
3148	Er	0.12	0.58	0.06	0.11	0	0.11	0.11	0.2
2858	Ar2	0.45	0.15	0.39	0.15	0.01	0.15	0.11	0.15
2859	Haer	0.55	0.24	0.26	0.13	0.03	0.15	0.06	0.17
2860	Er	0.46	0.17	0.3	0.14	0.03	0.17	0.06	0.16
2861	Roar	0.27	0.13	0.23	0.12	0.03	0.17	0.06	0.11

2862	Ar2	0.63	0.13	0.13	0.13	0.01	0.19	0.09	0.13
2863	Ar2	0.34	0.13	0.3	0.14	0.01	0.15	0.11	0.14
2864	Ar2	0.39	0.26	0.21	0.14	0.02	0.15	0.09	0.23
2865	Ar2	0.44	0.28	0.2	0.13	0.02	0.17	0.09	0.2
2866	Ar2	0.82	0.47	0.05	0.13	0.02	0.15	0.09	0.22
2867	Ar2	0.62	0.26	0.13	0.14	0.03	0.17	0.06	0.2
2868	Roar	0.4	0.13	0.24	0.13	0.03	0.19	0.06	0.15
2869	Haer	0.62	0.22	0.18	0.14	0.03	0.17	0.09	0.16
2870	Ar2	0.48	0.13	0.28	0.13	0.02	0.17	0.09	0.17
2871	Er	0.74	0.24	0.09	0.13	0.02	0.17	0.09	0.22
2872	Er	1.3	0.13	0.28	0.12	0.02	0.15	0.06	0.12
2873	Er	1.31	0.13	0.23	0.12	0.02	0.17	0.06	0.11
2874	Er	1.6	0.15	0.08	0.14	0.03	0.19	0.06	0.12
2875	Er	0.87	0.11	0.42	0.14	0.04	0.19	0.06	0.11
2876	Er	0.91	0.09	0.43	0.11	0.04	0.17	0.06	0.1
2877	Haer	1.1	0.09	0.21	0.1	0.03	0.22	0.04	0.08
2878	Haer	1.28	0.11	0.12	0.11	0.04	0.19	0.02	0.09
2879	Ar2	1.37	0.26	0.06	0.13	0.01	0.15	0.06	0.22
2880	Ar2	0.79	0.28	0.46	0.13	0.01	0.15	0.09	0.21
2881	Ar2	1.31	0.28	0.13	0.13	0.01	0.15	0.09	0.26
2882	Er	0.9	0.11	0.41	0.12	0.03	0.17	0.06	0.15
2883	Ar2	1.25	0.22	0.2	0.12	0.02	0.15	0.09	0.18
2884	Ar2	1.16	0.11	0.3	0.1	0.03	0.22	0.04	0.1
2885	Ar2	0.99	0.11	0.36	0.12	0.02	0.17	0.06	0.14
2886	Ar2	0.72	0.09	0.52	0.1	0.03	0.17	0.04	0.09
2887	Er	0.63	0.19	0.53	0.12	0.02	0.13	0.09	0.18
2888	Er	0.71	0.37	0.44	0.11	0.02	0.13	0.06	0.35
2889	Er	1.27	0.11	0.13	0.11	0.03	0.17	0.06	0.18
2890	Er	1.09	0.26	0.2	0.25	0.05	0	0	0.16
2891	Er	1.32	0.11	0.15	0.11	0.02	0.17	0.06	0.13
2892	Ar2	1.41	0.15	0.16	0.14	0.02	0.19	0.09	0.16
2893	Haer	1.18	0.22	0.25	0.13	0.04	0.17	0.06	0.14
2894	Ar2	1.51	0.32	0.07	0.13	0.03	0.17	0.09	0.19
2895	Ar2	1.18	0.43	0.25	0.13	0.02	0.17	0.06	0.31
2896	Er	1.32	0.41	0.18	0.12	0.03	0.19	0.06	0.15
2897	Er	0.65	0.32	0.47	0.15	0.02	0.22	0.06	0.27
2898	Er	0.63	0.13	0.53	0.15	0.03	0.19	0.06	0.19
2899	Er	0.66	0.32	0.51	0.16	0.02	0.17	0.09	0.25
2900	Ar2	0.83	0.11	0.45	0.13	0.03	0.17	0.09	0.18
2901	Ar2	0.87	0.15	0.43	0.16	0.03	0.19	0.09	0.18
2902	Ar2	0.86	0.11	0.41	0.12	0.05	0.22	0.06	0.15
2903	Ar2	1.38	0.28	0.09	0.14	0.02	0.17	0.09	0.25
2904	Ar2	1.47	0.17	0.04	0.16	0.02	0.17	0.09	0.29
2905	Haer	1.07	0.15	0.26	0.15	0.04	0.22	0.06	0.15
2906	Ar2	1.29	0.13	0.14	0.14	0.03	0.19	0.09	0.18
2907	Ar2	1.02	0.13	0.16	0.13	0.03	0.17	0.09	0.22
2908	Er	0.67	0.15	0.46	0.15	0.02	0.19	0.11	0.14
2909	Er	0.87	0.11	0.27	0.14	0.04	0.24	0.06	0.14
2910	Haer	1.14	0.19	0.15	0.14	0.04	0.17	0.09	0.15
2911	Haer	1.04	0.15	0.2	0.16	0.03	0.22	0.06	0.21
2912	Haer	0.91	0.45	0.13	0.14	0.03	0.24	0.06	0.21
2913	Haer	0.59	0.15	0.36	0.14	0.04	0.22	0.06	0.13
2914	Er	0.81	0.45	0.28	0.14	0.02	0.19	0.11	0.17
2915	Ar2	0.79	0.15	0.37	0.15	0.03	0.22	0.09	0.15

2916	Ar2	1.25	0.15	0.03	0.17	0.03	0.22	0.06	0.22
2917	Ar2	0.93	0.34	0.24	0.12	0.01	0.15	0.11	0.35
2918	Ar2	0.63	0.13	0.37	0.12	0.03	0.19	0.06	0.13
2919	Roar	0.55	0.45	0.38	0.11	0.03	0.15	0.06	0.32
2920	Ar2	0.56	0.37	0.38	0.13	0.03	0.19	0.06	0.18
2921	Ar2	0.55	0.41	0.37	0.12	0.02	0.15	0.09	0.23
2922	Er	0.72	0.37	0.24	0.13	0.02	0.15	0.09	0.24
3149	Er	0.11	0.58	0.06	0.11	0	0.11	0.11	0.22
2924	Ar2	0.59	0.11	0.31	0.11	0.02	0.17	0.09	0.12
2925	Ar2	0.86	0.45	0.13	0.11	0.03	0.17	0.06	0.24
2926	Ar2	0.54	0.13	0.36	0.13	0.02	0.19	0.09	0.18
2927	Ar2	0.77	0.26	0.16	0.15	0.02	0.22	0.06	0.21
2928	Ar2	0.96	0.15	0.25	0.15	0.02	0.19	0.09	0.14
2929	Er	0.95	0.13	0.23	0.14	0.02	0.19	0.11	0.14
2930	Haer	0.88	0.24	0.33	0.14	0.03	0.17	0.09	0.17
2931	Er	0.61	0.3	0.47	0.16	0.01	0.19	0.13	0.21
2932	Ar2	0.97	0.13	0.29	0.14	0.03	0.17	0.06	0.15
2933	Er	1.27	0.09	0.14	0.12	0.02	0.17	0.11	0.13
2934	Ar2	0.9	0.39	0.29	0.13	0.01	0.17	0.11	0.24
2935	Er	0.81	0.28	0.33	0.15	0.02	0.17	0.09	0.25
2936	Ar2	0.69	0.11	0.36	0.11	0.03	0.17	0.06	0.1
2937	Er	1.32	0.13	0.04	0.13	0.02	0.15	0.09	0.24
2938	Er	0.87	0.06	0.29	0.11	0.04	0.19	0.04	0.12
2939	Ar2	0.64	0.22	0.22	0.11	0.02	0.15	0.09	0.17
2940	Er	0.54	0.24	0.32	0.11	0.02	0.13	0.09	0.17
2941	Ar2	0.71	0.13	0.28	0.13	0.03	0.19	0.06	0.13
2942	Er	0.82	0.09	0.14	0.11	0.04	0.19	0.04	0.1
2943	Haer	0.82	0.09	0.16	0.09	0.02	0.13	0.06	0.12
2944	Haer	0.62	0.17	0.3	0.07	0.04	0.13	0.04	0.07
2945	Ar2	0.62	0.15	0.32	0.13	0.02	0.15	0.06	0.16
2946	Haer	0.65	0.24	0.27	0.12	0.02	0.15	0.06	0.16
2947	Ar2	0.64	0.09	0.29	0.1	0.02	0.13	0.04	0.08
2948	Er	1.07	0.13	0.13	0.12	0.02	0.15	0.06	0.1
2949	Haer	0.91	0.09	0.23	0.11	0.04	0.17	0.04	0.09
2950	Ar2	1.01	0.13	0.21	0.14	0.02	0.19	0.06	0.13
2951	Er	1.14	0.15	0.11	0.14	0.03	0.17	0.06	0.16
2952	Ar2	0.7	0.22	0.39	0.11	0.01	0.13	0.09	0.2
2953	Er	0.61	0.26	0.37	0.12	0.02	0.13	0.06	0.19
2954	Ar2	1.12	0.24	0.16	0.24	0.03	0.3	0.17	0.22
2955	Ar2	0.95	0.26	0.3	0.25	0.01	0.28	0.22	0.24
2956	Ar2	0.74	0.26	0.3	0.24	0.03	0.3	0.17	0.22
2957	Ar2	0.71	0.15	0.32	0.22	0.05	0.3	0.13	0.19
2958	Ar2	1.21	0.24	0.06	0.24	0.04	0.28	0.15	0.28
2959	Haer	0.95	0.22	0.22	0.24	0.04	0.28	0.15	0.19
2960	Ar2	1.14	0.28	0.15	0.27	0.04	0.34	0.17	0.26
2961	Ar2	0.69	0.3	0.51	0.26	0.04	0.3	0.15	0.24
2962	Ar2	0.66	0.43	0.36	0.22	0.03	0.26	0.17	0.42
2963	Ar2	0.53	0.15	0.37	0.19	0.05	0.28	0.11	0.2
2964	Ar2	0.83	0.26	0.22	0.2	0.04	0.28	0.13	0.17
2965	Ar2	0.78	0.24	0.27	0.24	0.04	0.3	0.11	0.24
2966	Ar2	0.82	0.28	0.31	0.23	0.07	0.37	0.13	0.2
2967	Ar2	0.79	0.32	0.26	0.27	0.06	0.39	0.09	0.26
2968	Ar2	1.02	0.3	0.16	0.28	0.04	0.34	0.11	0.31
2969	Er	0.92	0.39	0.36	0.19	0.02	0.22	0.17	0.32

2970	Haer	0.89	0.22	0.33	0.18	0.05	0.26	0.09	0.15
2971	Er	1.23	0.34	0.27	0.19	0.03	0.26	0.15	0.24
2972	Ar2	1.06	0.24	0.43	0.21	0.03	0.26	0.13	0.22
2973	Ar2	1.52	0.15	0.07	0.2	0.04	0.26	0.11	0.22
2974	Ar2	0.93	0.28	0.39	0.17	0.05	0.26	0.09	0.16
2975	Ar2	1.45	0.17	0.03	0.2	0.05	0.26	0.11	0.2
2976	Ar2	1.24	0.3	0.2	0.21	0.03	0.24	0.15	0.24
2977	Ar2	0.88	0.17	0.44	0.18	0.05	0.28	0.11	0.17
2978	Ar2	0.9	0.45	0.39	0.21	0.03	0.26	0.17	0.27
2979	Ar2	0.85	0.6	0.38	0.22	0.04	0.26	0.15	0.23
2980	Ar2	0.65	0.26	0.52	0.24	0.05	0.3	0.11	0.25
2981	Er	1.31	0.19	0.08	0.21	0.07	0.34	0.06	0.18
2982	Ar2	0.73	0.19	0.42	0.2	0.03	0.24	0.13	0.23
2983	Ar2	0.69	0.15	0.43	0.18	0.04	0.26	0.15	0.17
2984	Ar2	1.22	0.17	0.22	0.19	0.04	0.26	0.13	0.2
2985	Ar2	0.64	0.19	0.51	0.2	0.04	0.3	0.11	0.21
2986	Ar2	1.09	0.45	0.21	0.21	0.03	0.24	0.11	0.39
2987	Er	1.12	0.19	0.32	0.19	0.02	0.22	0.13	0.2
2988	Ar2	1.19	0.15	0.26	0.16	0.03	0.24	0.11	0.14
2989	Er	0.99	0.13	0.27	0.15	0.04	0.24	0.06	0.12
2990	Haer	1.28	0.26	0.14	0.15	0.03	0.22	0.11	0.15
2991	Ar2	1.48	0.19	0.05	0.16	0.02	0.24	0.13	0.15
2992	Er	1.01	0.15	0.35	0.16	0.02	0.24	0.11	0.17
2993	Haer	1.12	0.17	0.23	0.13	0.05	0.24	0.06	0.12
2994	Ar2	0.76	0.24	0.48	0.18	0.03	0.22	0.09	0.18
2995	Er	0.86	0.3	0.41	0.15	0.02	0.19	0.11	0.23
2996	Ar2	1.28	0.41	0.1	0.18	0.04	0.24	0.04	0.25
2997	Haer	1.3	0.43	0.15	0.15	0.04	0.24	0.06	0.23
2998	Ar2	1.11	0.19	0.3	0.17	0.03	0.22	0.11	0.19
2999	Haer	1.47	0.19	0.09	0.17	0.03	0.24	0.09	0.2
3000	Ar2	1.43	0.17	0.13	0.17	0.03	0.24	0.09	0.21
3001	Er	1.02	0.41	0.35	0.16	0.02	0.19	0.13	0.22
3002	Er	0.95	0.47	0.39	0.17	0.02	0.19	0.13	0.34
3003	Ar2	1.18	0.22	0.16	0.18	0.03	0.22	0.11	0.19
3004	Ar2	0.61	0.17	0.51	0.17	0.03	0.24	0.09	0.19
3005	Ar2	0.68	0.13	0.44	0.15	0.04	0.22	0.09	0.16
3006	Ar2	0.68	0.24	0.44	0.15	0.04	0.19	0.09	0.17
3007	Ar2	0.76	0.19	0.44	0.18	0.03	0.22	0.13	0.16
3008	Ar2	0.72	0.43	0.38	0.19	0.03	0.24	0.11	0.23
3009	Ar2	1.01	0.09	0.19	0.18	0.03	0.24	0.09	0.32
3010	Er	0.84	0.43	0.29	0.18	0.03	0.22	0.09	0.27
3011	Ar2	0.81	0.22	0.28	0.22	0.02	0.26	0.15	0.23
3012	Ar2	1.02	0.24	0.17	0.21	0.02	0.3	0.15	0.2
3013	Ar2	0.75	0.15	0.31	0.18	0.06	0.28	0.09	0.16
3014	Ar2	0.99	0.32	0.22	0.22	0.03	0.28	0.15	0.2
3015	Haer	1.01	0.13	0.12	0.16	0.04	0.26	0.13	0.15
3016	Ar2	0.89	0.32	0.19	0.21	0.02	0.24	0.13	0.22
3017	Er	0.52	0.22	0.37	0.21	0.02	0.24	0.17	0.19
3018	Ar2	0.41	0.13	0.37	0.17	0.05	0.26	0.11	0.15
3019	Haer	0.63	0.22	0.26	0.2	0.04	0.26	0.13	0.17
3020	Er	0.59	0.15	0.34	0.17	0.02	0.22	0.15	0.16
3021	Ar2	0.83	0.19	0.16	0.17	0.03	0.22	0.13	0.13
3022	Er	0.64	0.17	0.34	0.15	0.03	0.24	0.11	0.14
3023	Er	0.64	0.15	0.29	0.15	0.04	0.26	0.09	0.13

3024	Haer	0.57	0.22	0.39	0.15	0.04	0.19	0.09	0.14
3025	Er	1.04	0.15	0.14	0.17	0.04	0.24	0.06	0.14
3026	Er	0.86	0.15	0.32	0.16	0.02	0.22	0.13	0.17
3027	Er	0.62	0.28	0.38	0.19	0.03	0.22	0.09	0.23
3028	Haer	0.88	0.15	0.25	0.16	0.03	0.22	0.11	0.16
3029	Ar2	0.79	0.17	0.34	0.16	0.03	0.22	0.11	0.16
3030	Er	0.58	0.17	0.48	0.17	0.02	0.22	0.13	0.19
3031	Er	0.53	0.13	0.5	0.18	0.02	0.24	0.13	0.21
3032	Haer	1.12	0.22	0.16	0.17	0.04	0.24	0.13	0.13
3033	Ar2	0.82	0.22	0.34	0.18	0.03	0.22	0.13	0.17
3034	Haer	0.78	0.17	0.3	0.16	0.05	0.28	0.06	0.13
3035	Haer	0.52	0.17	0.06	0.15	0.04	0.24	0.06	0.18
3036	Er	0.54	0.45	0.056528	0.14	0.02	0.15	0.11	0.34
3037	Ar2	0.33	0.71	0.12	0.15	0.02	0.17	0.09	0.16
3038	Roar	0.55	0.11	0.15	0.15	0.03	0.19	0.11	0.18
3039	Er	0.35	0.15	0.27	0.14	0.02	0.17	0.11	0.18
3150	Er	0.15	0.62	0.03	0.12	0.01	0.13	0.11	0.2
3041	Ar2	0.48	0.11	0.14	0.13	0.03	0.22	0.09	0.14
3042	Ar2	0.31	0.15	0.2	0.16	0.02	0.24	0.11	0.15
3043	Ar2	0.28	0.15	0.14	0.13	0.03	0.19	0.09	0.13
3044	Haer	0.33	0.11	0.13	0.12	0.04	0.19	0.06	0.11
3045	Er	0.31	0.34	0.1	0.14	0.02	0.17	0.11	0.29
3046	Er	0.34	0.43	0.151275	0.14	0.02	0.17	0.11	0.21
3047	Er	0.45	0.43	0.06	0.13	0.03	0.17	0.09	0.18
3048	Roar	0.37	0.13	0.13	0.13	0.03	0.19	0.11	0.15
3049	Er	0.39	0.39	0.08	0.13	0.01	0.15	0.11	0.25
3050	Er	0.27	0.34	0.18	0.11	0.02	0.15	0.09	0.22
3051	Er	0.25	0.37	0.19	0.12	0.01	0.15	0.11	0.29
3052	Er	0.39	0.41	0.11	0.11	0.01	0.13	0.09	0.25
3053	Ar2	0.38	0.09	0.15	0.12	0.03	0.15	0.06	0.14
3151	Er	0.07	0.22	0.04	0.12	0.01	0.13	0.11	0.22
3152	Er	0.09	0.37	0.04	0.13	0.01	0.13	0.11	0.23
3153	Er	0.08	0.5	0.04	0.12	0.01	0.13	0.11	0.19
3057	Ar2	0.36	0.67	0.06	0.12	0.02	0.17	0.06	0.21
3058	Er	0.28	0.58	0.1	0.08	0.02	0.11	0.04	0.13
3059	Ar2	0.28	0.26	0.14	0.11	0.02	0.13	0.09	0.22
3154	Er	0.09	0.56	0.03	0.13	0.01	0.15	0.13	0.24
3061	Er	0.2	0.24	0.15	0.11	0.02	0.15	0.06	0.21
3062	Er	0.2	0.24	0.15	0.11	0.02	0.15	0.06	0.21
3155	Er	0.07	0.6	0.02	0.13	0.01	0.15	0.13	0.19
3064	Ar2	0.39	0.88	0.22	0.09	0.01	0.13	0.09	0.13
3065	Roar	0.35	0.54	0.22	0.08	0.02	0.13	0.06	0.36
3156	Er	0.06	0.6	0.03	0.12	0.01	0.13	0.11	0.23
3157	Er	0.05	0.41	0.03	0.12	0.01	0.13	0.11	0.27
3068	Ar2	0.54	0.65	0.08	0.11	0.02	0.17	0.06	0.21
3069	Haer	0.7	0.65	0.06	0.11	0.03	0.17	0.06	0.22
3158	Er	0.11	0.41	0.04	0.13	0	0.13	0.13	0.32
3159	Er	0.08	0.67	0.03	0.13	0.01	0.13	0.11	0.22
3162	Er	0.1	0.37	0.06	0.13	0.03	0.15	0.09	0.28
3073	Haer	0.58	0.19	0.1	0.11	0.02	0.13	0.04	0.16
3163	Er	0.17	0.54	0.04	0.13	0.01	0.15	0.13	0.29
3165	Er	0.11	0.56	0.02	0.13	0.01	0.15	0.13	0.24
3166	Er	0.1	0.56	0.04	0.13	0.01	0.15	0.13	0.25
3167	Er	0.11	0.22	0.02	0.13	0.02	0.17	0.11	0.2

3169	Er	0.12	0.19	0.06	0.18	0.01	0.19	0.17	0.21
3079	Roar	0.16	0.69	0.05	0.11	0.02	0.13	0.09	0.34
3170	Er	0.11	0.65	0.03	0.17	0.01	0.17	0.15	0.27
3173	Er	0.15	0.39	0.06	0.18	0.01	0.19	0.17	0.34
3174	Er	0.1	0.54	0.04	0.18	0.01	0.19	0.17	0.33
3177	Er	0.07	0.54	0.04	0.17	0	0.17	0.17	0.22
3084	Er	0.08	0.22	0.05	0.12	0.01	0.13	0.11	0.3
3178	Er	0.08	0.52	0.05	0.17	0.01	0.17	0.15	0.21
3179	Er	0.1	0.47	0.02	0.15	0.01	0.17	0.15	0.2
3087	Ar2	0.14	0.15	0.04	0.13	0.03	0.17	0.09	0.13
3180	Er	0.07	0.47	0.02	0.16	0.01	0.17	0.15	0.22
3181	Er	0.16	0.34	0.02	0.17	0	0.17	0.17	0.2
3183	Er	0.14	0.56	0.05	0.19	0.01	0.19	0.17	0.32
3184	Er	0.1	0.52	0.06	0.17	0.02	0.19	0.15	0.28
3186	Er	0.14	0.6	0.04	0.15	0.01	0.17	0.15	0.27
3093	Er	0.1	0.22	0.05	0.11	0.02	0.13	0.09	0.18
3188	Er	0.11	0.19	0.07	0.18	0.02	0.19	0.15	0.21
3095	Ar2	0.17	0.22	0.06	0.12	0.02	0.15	0.09	0.2
3189	Er	0.14	0.47	0.03	0.16	0.01	0.19	0.15	0.34
3192	Er	0.07	0.19	0.06	0.2	0.01	0.22	0.19	0.19
3195	Er	0.05	0.19	0.04	0.2	0.01	0.22	0.19	0.2
3099	Ar2	0.14	0.09	0.09	0.08	0.03	0.13	0.06	0.08
3100	Ar2	0.13	0.8	0.09	0.11	0.01	0.13	0.09	0.65
3203	Er	0.12	0.19	0.05	0.19	0.02	0.22	0.15	0.23
3102	Ar2	0.16	0.88	0.1	0.13	0.02	0.15	0.11	0.34
3103	Ar2	0.3	0.13	0.08	0.14	0.02	0.19	0.09	0.21
3104	Ar2	0.2	0.11	0.07	0.11	0.01	0.13	0.11	0.27
3207	Ar2	0.09	0.17	0.05	0.18	0.01	0.19	0.17	0.21
3106	Roar	0.15	0.54	0.05	0.12	0.03	0.15	0.06	0.17
3107	Er	0.17	0.15	0.14	0.14	0.02	0.19	0.11	0.16
3108	Roar	0.26	0.45	0.07	0.13	0.03	0.22	0.06	0.18
3109	Er	0.21	0.3	0.042408	0.14	0.03	0.17	0.09	0.24
3110	Ar2	0.18	0.15	0.06	0.14	0.03	0.17	0.09	0.18
3270	Er	0.18	0.24	0.06	0.11	0.01	0.13	0.09	0.18
3272	Er	0.14	0.22	0.1	0.11	0.01	0.13	0.11	0.2
3308	Er	0.12	0.22	0.02	0.08	0.01	0.11	0.06	0.15
3114	Er	0.17	0.45	0.12	0.14	0.01	0.15	0.11	0.25
3313	Er	0.1	0.24	0.05	0.1	0.01	0.11	0.09	0.2
3317	Er	0.08	0.19	0.05	0.11	0.01	0.13	0.11	0.23
3318	Er	0.07	0.11	0.05	0.11	0.01	0.13	0.11	0.15
3118	Er	0.14	0.45	0.08	0.13	0.02	0.15	0.09	0.18
3119	Roar	0.25	0.13	0.1	0.14	0.02	0.19	0.09	0.25
3120	Er	0.14	0.56	0.08	0.12	0.02	0.15	0.06	0.43
3325	Er	0.12	0.26	0.06	0.14	0.02	0.17	0.11	0.22
3328	Er	0.15	0.32	0.07	0.14	0.01	0.15	0.11	0.25
3329	Er	0.14	0.39	0.08	0.13	0.01	0.15	0.13	0.24
3334	Er	0.2	0.26	0.07	0.11	0.01	0.13	0.09	0.22
3335	Er	0.2	0.28	0.04	0.12	0.01	0.13	0.11	0.23
3338	Er	0.06	0.39	0.03	0.12	0.01	0.13	0.11	0.25
3339	Er	0.07	0.39	0.03	0.12	0.01	0.13	0.11	0.18
3128	Ar2	0.13	0.17	0.08	0.15	0.02	0.17	0.11	0.19
3340	Er	0.08	0.39	0.03	0.13	0.01	0.15	0.11	0.21
3130	Er	0.12	0.13	0.08	0.14	0.03	0.17	0.09	0.29
3341	Er	0.07	0.15	0.02	0.13	0.01	0.15	0.13	0.18

3359	Er	0.35	0.67	0.27	0.2	0.02	0.24	0.17	0.38
3133	Er	0.07	0.6	0.02	0.15	0.01	0.15	0.13	0.32
3362	Er	0.44	0.62	0.28	0.18	0.02	0.22	0.15	0.37
3369	Ar2	0.53	0.67	0.05	0.17	0.02	0.19	0.11	0.55
3378	Ar2	0.3	0.65	0.14	0.2	0.01	0.22	0.17	0.42
3383	Ar2	0.25	0.22	0.16	0.21	0.02	0.26	0.17	0.35
3426	Ar2	0.22	0.47	0.19	0.19	0.03	0.24	0.13	0.42
3428	Er	0.24	0.69	0.21	0.2	0.02	0.26	0.17	0.38
3432	Er	0.31	0.47	0.12	0.23	0.02	0.26	0.17	0.35
3435	Er	0.28	0.65	0.23	0.16	0.01	0.19	0.15	0.31
3436	Ar2	0.47	0.22	0.2	0.19	0.02	0.22	0.15	0.27
3440	Er	0.32	0.62	0.18	0.2	0.01	0.22	0.17	0.37
3447	Ar2	0.48	0.19	0.16	0.2	0.02	0.26	0.17	0.23
3453	Er	0.39	0.8	0.27	0.19	0.01	0.39	0.19	0.44
3458	Ar2	0.35	0.73	0.29	0.23	0.02	0.32	0.19	0.47
3459	Er	0.42	0.62	0.194975	0.24	0.05	0.32	0.13	0.41
3461	Er	0.46	0.67	0.16	0.26	0.03	0.3	0.19	0.41
3463	Er	0.33	0.56	0.321607	0.25	0.05	0.32	0.17	0.35
3487	Er	0.11	0.39	0.420711	0.23	0.04	0.26	0.19	0.35
3492	Er	0.19	0.69	0.15	0.31	0.03	0.34	0.24	0.39
3494	Ar2	0.33	0.65	0.11	0.32	0.03	0.34	0.22	0.41
3496	Er	0.14	0.71	0.12	0.28	0.03	0.3	0.24	0.4
3498	Er	0.11	0.73	0.05	0.27	0.02	0.28	0.24	0.45
3499	Er	0.1	0.69	0.05	0.29	0.03	0.3	0.22	0.4
3501	Er	0.09	0.73	0.06	0.26	0.02	0.28	0.22	0.33
3507	Ar2	0.23	0.86	0.18	0.24	0.03	0.3	0.19	0.61
3512	Er	0.17	0.73	0.12	0.23	0.03	0.28	0.17	0.44
3513	Ar2	0.21	0.67	0.08	0.22	0.02	0.28	0.19	0.49
3160	Er	0.06	0.15	0.02	0.15	0.01	0.15	0.13	0.17
3161	Er	0.08	0.15	0.04	0.14	0.01	0.15	0.13	0.16
3517	Er	0.15	0.56	0.11	0.18	0.02	0.22	0.15	0.36
3518	Ar2	0.17	0.75	0.07	0.19	0.01	0.19	0.17	0.43
3164	Er	0.12	0.45	0.03	0.14	0.01	0.15	0.13	0.34
3519	Er	0.07	0.52	0.05	0.17	0.05	0.22	0.09	0.43
3520	Er	0.16	0.45	0.07	0.21	0.01	0.24	0.19	0.41
3522	Er	0.08	0.65	0.06	0.2	0.01	0.22	0.19	0.47
3168	Er	0.1	0.17	0.04	0.16	0.03	0.17	0.09	0.18
3523	Er	0.09	0.62	0.06	0.2	0.01	0.22	0.19	0.33
3525	Er	0.16	0.62	0.05	0.18	0.03	0.22	0.15	0.48
3171	Ar2	0.09	0.5	0.06	0.17	0.01	0.19	0.15	0.34
3172	Er	0.07	0.54	0.02	0.19	0.01	0.19	0.17	0.35
3526	Er	0.09	0.67	0.04	0.18	0.03	0.24	0.15	0.4
3527	Ar2	0.18	0.93	0.11	0.22	0.02	0.24	0.17	0.74
3175	Ar2	0.11	0.65	0.04	0.17	0.02	0.22	0.15	0.34
3176	Er	0.08	0.62	0.03	0.17	0.02	0.19	0.15	0.42
3528	Er	0.17	0.95	0.11	0.2	0.02	0.24	0.17	0.55
3529	Er	0.1	0.73	0.07	0.22	0.03	0.24	0.15	0.65
3530	Ar2	0.24	0.71	0.04	0.23	0.03	0.28	0.15	0.52
3531	Er	0.14	0.88	0.06	0.22	0.03	0.28	0.17	0.34
3534	Er	0.12	0.6	0.03	0.2	0.02	0.26	0.19	0.43
3182	Ar2	0.16	0.17	0.07	0.18	0.02	0.22	0.15	0.2
3536	Er	0.08	0.24	0.06	0.22	0.04	0.28	0.17	0.27
3537	Ar2	0.14	0.26	0.07	0.23	0.04	0.28	0.17	0.29
3185	Er	0.12	0.5	0.07	0.14	0.03	0.19	0.11	0.19

3538	Er	0.12	0.75	0.07	0.2	0.03	0.24	0.15	0.61
3187	Er	0.09	0.17	0.06	0.16	0.02	0.19	0.15	0.17
3539	Er	0.12	0.9	0.08	0.22	0.01	0.24	0.19	0.47
3540	Er	0.1	0.82	0.06	0.19	0.02	0.22	0.17	0.61
3190	Ar2	0.11	0.34	0.06	0.2	0.02	0.22	0.15	0.26
3191	Er	0.1	0.17	0.06	0.17	0.04	0.24	0.13	0.17
3541	Er	0.13	0.75	0.07	0.23	0.02	0.26	0.19	0.45
3193	Er	0.09	0.22	0.05	0.2	0.02	0.22	0.17	0.19
3194	Er	0.08	0.28	0.04	0.19	0.02	0.22	0.17	0.22
3542	Er	0.12	0.75	0.07	0.2	0.03	0.24	0.17	0.55
3196	Er	0.14	0.3	0.09	0.18	0.02	0.24	0.17	0.19
3197	Er	0.1	0.39	0.07	0.2	0.01	0.22	0.17	0.3
3198	Er	0.08	0.17	0.06	0.2	0.02	0.22	0.17	0.21
3199	Er	0.11	0.37	0.03	0.2	0.03	0.24	0.13	0.23
3200	Er	0.14	0.52	0.03	0.18	0.02	0.19	0.15	0.21
3201	Er	0.12	0.37	0.05	0.18	0.02	0.19	0.15	0.31
3202	Er	0.09	0.43	0.06	0.18	0.02	0.22	0.15	0.18
3543	Er	0.15	0.69	0.05	0.22	0.03	0.26	0.13	0.64
3204	Er	0.09	0.47	0.04	0.18	0.02	0.19	0.15	0.25
3205	Er	0.09	0.43	0.03	0.16	0.02	0.19	0.15	0.35
3206	Er	0.1	0.17	0.03	0.17	0.02	0.19	0.15	0.2
3547	Er	0.07	0.26	0.04	0.25	0.03	0.32	0.24	0.3
3208	Er	0.09	0.19	0.05	0.18	0.01	0.19	0.17	0.2
3209	Er	0.09	0.26	0.06	0.18	0.01	0.19	0.17	0.22
3210	Er	0.11	0.26	0.06	0.17	0.02	0.19	0.15	0.2
3211	Er	0.09	0.26	0.03	0.16	0.03	0.19	0.13	0.22
3212	Ar2	0.26	0.41	0.14	0.15	0.02	0.19	0.13	0.19
3213	Er	0.23	0.5	0.13	0.16	0.02	0.19	0.13	0.2
3214	Roar	0.27	0.17	0.12	0.16	0.03	0.19	0.11	0.17
3215	Roar	0.26	0.13	0.09	0.14	0.04	0.22	0.09	0.13
3216	Er	0.3	0.47	0.06	0.16	0.02	0.19	0.11	0.16
3217	Ar2	0.19	0.13	0.14	0.14	0.02	0.17	0.13	0.16
3218	Haer	0.31	0.13	0.07	0.15	0.03	0.22	0.11	0.14
3219	Er	0.2	0.3	0.07	0.14	0.04	0.17	0.09	0.23
3220	Haer	0.3	0.32	0.05	0.16	0.02	0.19	0.13	0.21
3221	Er	0.24	0.17	0.09	0.17	0.02	0.19	0.13	0.19
3222	Ar2	0.21	0.28	0.1	0.15	0.02	0.19	0.13	0.25
3223	Er	0.14	0.39	0.1	0.18	0.01	0.19	0.17	0.34
3224	Er	0.16	0.22	0.1	0.2	0.03	0.22	0.11	0.19
3225	Er	0.19	0.41	0.12	0.19	0.01	0.22	0.17	0.19
3226	Er	0.26	0.34	0.04	0.16	0.01	0.19	0.15	0.28
3227	Ar2	0.13	0.32	0.09	0.15	0.02	0.17	0.13	0.21
3228	Er	0.13	0.32	0.05	0.14	0.03	0.19	0.11	0.25
3229	Ar2	0.17	0.17	0.07	0.17	0.02	0.19	0.13	0.17
3230	Ar2	0.19	0.15	0.08	0.16	0.03	0.22	0.13	0.14
3231	Ar2	0.18	0.19	0.08	0.16	0.03	0.22	0.13	0.19
3232	Ar2	0.22	0.11	0.09	0.18	0.02	0.22	0.15	0.14
3233	Er	0.12	0.41	0.06	0.17	0.01	0.17	0.15	0.26
3234	Er	0.13	0.45	0.06	0.15	0.03	0.22	0.11	0.22
3235	Er	0.16	0.45	0.07	0.18	0.01	0.19	0.15	0.26
3236	Er	0.24	0.17	0.07	0.17	0.01	0.19	0.15	0.16
3237	Er	0.17	0.17	0.1	0.17	0.01	0.17	0.15	0.15
3238	Ar2	0.23	0.17	0.08	0.16	0.01	0.19	0.15	0.14
3239	Er	0.27	0.13	0.04	0.16	0.02	0.19	0.11	0.15

3240	Er	0.19	0.22	0.08	0.15	0.03	0.19	0.09	0.14
3241	Er	0.27	0.17	0.12	0.16	0.02	0.19	0.13	0.19
3242	Er	0.26	0.15	0.08	0.15	0.02	0.19	0.13	0.16
3243	Haer	0.28	0.15	0.06	0.16	0.03	0.19	0.11	0.17
3244	Ar2	0.21	0.17	0.09	0.18	0.03	0.24	0.11	0.16
3245	Ar2	0.2	0.19	0.14	0.18	0.03	0.24	0.11	0.15
3246	Er	0.21	0.15	0.06	0.17	0.02	0.22	0.13	0.15
3247	Er	0.29	0.19	0.05	0.19	0.02	0.22	0.11	0.17
3248	Ar2	0.17	0.19	0.1	0.17	0.02	0.22	0.13	0.15
3249	Er	0.31	0.15	0.13	0.14	0.02	0.17	0.11	0.16
3250	Er	0.44	0.15	0.08	0.14	0.02	0.17	0.11	0.15
3251	Er	0.46	0.28	0.04	0.14	0.01	0.17	0.11	0.22
3252	Er	0.46	0.24	0.04	0.14	0.01	0.17	0.11	0.19
3253	Ar2	0.42	0.17	0.12	0.16	0.02	0.19	0.11	0.15
3254	Er	0.44	0.15	0.08	0.16	0.02	0.19	0.11	0.13
3255	Er	0.37	0.24	0.12	0.15	0.02	0.17	0.13	0.15
3256	Er	0.33	0.13	0.07	0.15	0.02	0.19	0.11	0.13
3257	Er	0.22	0.15	0.16	0.16	0.02	0.17	0.13	0.16
3258	Ar2	0.26	0.17	0.14	0.16	0.02	0.19	0.11	0.12
3259	Ar2	0.38	0.19	0.1	0.18	0.03	0.22	0.11	0.17
3260	Ar2	0.4	0.22	0.09	0.17	0.03	0.22	0.09	0.15
3261	Er	0.35	0.19	0.06	0.16	0.05	0.24	0.04	0.12
3262	Ar2	0.26	0.19	0.19	0.2	0.01	0.22	0.17	0.19
3263	Er	0.33	0.17	0.14	0.18	0.01	0.22	0.17	0.17
3264	Haer	0.41	0.24	0.09	0.18	0.03	0.24	0.13	0.17
3265	Er	0.28	0.15	0.21	0.17	0.03	0.24	0.13	0.16
3266	Er	0.25	0.15	0.11	0.15	0.01	0.15	0.13	0.14
3267	Er	0.23	0.24	0.1	0.14	0.01	0.15	0.13	0.17
3268	Ar2	0.21	0.13	0.09	0.14	0.02	0.17	0.11	0.14
3269	Er	0.12	0.13	0.1	0.13	0.02	0.17	0.11	0.13
3550	Er	0.19	0.28	0.13	0.25	0.03	0.3	0.22	0.23
3271	Er	0.13	0.11	0.07	0.12	0.02	0.17	0.09	0.11
3562	Er	0.09	0.58	0.05	0.21	0.02	0.24	0.19	0.45
3273	Roar	0.21	0.13	0.1	0.13	0.01	0.15	0.11	0.14
3274	Er	0.11	0.22	0.05	0.12	0.01	0.13	0.11	0.13
3275	Er	0.1	0.13	0.08	0.13	0	0.13	0.13	0.12
3276	Er	0.26	0.22	0.03	0.12	0.01	0.13	0.11	0.13
3277	Ar2	0.28	0.13	0.09	0.11	0.01	0.13	0.11	0.11
3278	Er	0.2	0.11	0.1	0.11	0.01	0.13	0.11	0.13
3279	Er	0.42	0.26	0.04	0.13	0.02	0.17	0.06	0.17
3280	Ar2	0.22	0.13	0.19	0.13	0.01	0.15	0.11	0.2
3281	Er	0.39	0.28	0.08	0.12	0.02	0.15	0.11	0.15
3282	Er	0.38	0.09	0.03	0.12	0.03	0.15	0.06	0.12
3283	Er	0.19	0.22	0.14	0.12	0.01	0.13	0.11	0.21
3284	Er	0.27	0.15	0.09	0.14	0.02	0.15	0.09	0.16
3285	Ar2	0.34	0.06	0.08	0.09	0.03	0.15	0.02	0.09
3286	Haer	0.29	0.17	0.09	0.08	0.02	0.13	0.04	0.1
3287	Ar2	0.27	0.11	0.16	0.09	0.01	0.13	0.06	0.09
3288	Ar2	0.37	0.11	0.08	0.12	0.02	0.15	0.06	0.1
3289	Er	0.23	0.11	0.19	0.12	0.01	0.13	0.11	0.12
3290	Er	0.06	0.15	0.04	0.15	0	0.15	0.15	0.15
3291	Er	0.07	0.24	0.03	0.15	0	0.15	0.15	0.22
3292	Er	0.1	0.15	0.07	0.15	0.01	0.15	0.13	0.15
3293	Er	0.11	0.13	0.08	0.15	0.01	0.17	0.13	0.16

3294	Er	0.11	0.15	0.08	0.15	0.01	0.15	0.13	0.17
3295	Er	0.12	0.15	0.06	0.15	0.01	0.15	0.13	0.14
3296	Er	0.09	0.13	0.05	0.13	0.02	0.15	0.11	0.15
3297	Er	0.11	0.28	0.03	0.14	0.01	0.15	0.13	0.18
3298	Er	0.1	0.24	0.03	0.13	0.01	0.15	0.11	0.2
3299	Er	0.12	0.13	0.07	0.14	0.02	0.17	0.13	0.15
3300	Er	0.19	0.15	0.02	0.14	0.02	0.19	0.13	0.16
3301	Er	0.12	0.3	0.05	0.16	0.01	0.17	0.15	0.14
3302	Er	0.09	0.13	0.05	0.14	0.02	0.17	0.13	0.18
3303	Er	0.1	0.34	0.02	0.15	0.01	0.17	0.13	0.21
3304	Er	0.08	0.15	0.04	0.15	0.01	0.17	0.15	0.15
3305	Er	0.12	0.26	0.04	0.14	0.02	0.17	0.13	0.17
3306	Er	0.08	0.17	0.06	0.15	0.02	0.17	0.13	0.15
3307	Er	0.11	0.17	0.05	0.15	0.02	0.17	0.13	0.15
3563	Er	0.11	0.6	0.06	0.22	0.02	0.24	0.19	0.34
3309	Er	0.1	0.19	0.06	0.08	0.01	0.11	0.06	0.17
3310	Er	0.08	0.19	0.03	0.1	0.01	0.11	0.09	0.13
3311	Er	0.1	0.17	0.03	0.11	0.01	0.11	0.09	0.15
3312	Er	0.1	0.11	0.07	0.1	0.01	0.11	0.09	0.11
3565	Er	0.1	0.45	0.04	0.22	0.03	0.24	0.17	0.3
3314	Er	0.11	0.11	0.07	0.11	0.01	0.13	0.09	0.18
3315	Ar2	0.07	0.43	0.02	0.11	0	0.11	0.11	0.17
3316	Er	0.08	0.11	0.04	0.12	0.01	0.13	0.11	0.14
3566	Er	0.09	0.22	0.07	0.22	0.01	0.24	0.22	0.36
3567	Er	0.11	0.47	0.03	0.21	0.02	0.24	0.19	0.33
3319	Er	0.13	0.24	0.07	0.12	0.02	0.15	0.11	0.21
3320	Er	0.11	0.34	0.06	0.12	0.02	0.15	0.11	0.23
3321	Er	0.14	0.26	0.08	0.13	0.02	0.15	0.11	0.23
3322	Er	0.15	0.43	0.04	0.13	0.02	0.17	0.11	0.14
3323	Er	0.1	0.24	0.07	0.12	0.03	0.15	0.09	0.22
3324	Er	0.1	0.37	0.04	0.13	0.02	0.15	0.11	0.18
3568	Er	0.06	0.28	0.04	0.25	0.02	0.28	0.22	0.28
3326	Er	0.12	0.34	0.06	0.14	0.02	0.15	0.11	0.19
3327	Er	0.11	0.34	0.08	0.13	0.02	0.15	0.11	0.34
3569	Er	0.07	0.62	0.04	0.23	0.02	0.26	0.22	0.44
3570	Er	0.11	0.56	0.06	0.24	0.03	0.28	0.19	0.38
3330	Ar2	0.23	0.13	0.05	0.12	0.02	0.17	0.11	0.11
3331	Ar2	0.26	0.17	0.05	0.16	0.02	0.17	0.09	0.18
3332	Er	0.11	0.13	0.08	0.12	0.02	0.15	0.09	0.13
3333	Ar2	0.17	0.26	0.07	0.13	0.02	0.15	0.09	0.16
3576	Er	0.19	0.24	0.16	0.23	0.01	0.26	0.22	0.25
3585	Er	0.19	0.6	0.05	0.2	0.01	0.22	0.19	0.42
3336	Ar2	0.15	0.26	0.07	0.11	0.02	0.13	0.09	0.18
3337	Er	0.16	0.34	0.06	0.12	0.01	0.13	0.09	0.25
3587	Er	0.18	0.58	0.08	0.19	0.02	0.22	0.15	0.32
3588	Er	0.17	0.58	0.08	0.19	0.01	0.22	0.17	0.3
3591	Er	0.07	0.24	0.06	0.22	0.02	0.24	0.19	0.21
3592	Er	0.07	0.19	0.04	0.21	0.02	0.24	0.19	0.23
3342	Er	0.06	0.39	0.02	0.13	0.01	0.15	0.13	0.17
3343	Ar2	0.61	0.9	0.19	0.19	0.03	0.24	0.13	0.55
3344	Roar	0.45	0.73	0.23	0.18	0.03	0.26	0.11	0.64
3345	Roar	0.5	0.69	0.17	0.18	0.03	0.26	0.13	0.56
3346	Ar2	0.47	0.71	0.19	0.19	0.03	0.28	0.11	0.47
3347	Roar	0.47	0.78	0.15	0.18	0.04	0.24	0.11	0.38

3348	Roar	0.41	0.69	0.15	0.17	0.04	0.26	0.11	0.46
3349	Ar2	0.42	0.15	0.31	0.18	0.02	0.24	0.15	0.36
3350	Ar2	0.61	0.73	0.15	0.17	0.03	0.22	0.11	0.43
3351	Ar2	0.5	0.86	0.26	0.18	0.03	0.24	0.13	0.25
3352	Ar2	0.42	0.73	0.3	0.16	0.04	0.22	0.09	0.4
3353	Ar2	0.68	0.19	0.23	0.19	0.03	0.26	0.13	0.32
3354	Ar2	0.74	0.22	0.13	0.19	0.04	0.24	0.11	0.27
3355	Ar2	0.77	0.86	0.07	0.17	0.04	0.22	0.11	0.33
3356	Ar2	0.48	0.78	0.23	0.19	0.04	0.26	0.11	0.32
3357	Ar2	0.68	0.22	0.16	0.22	0.03	0.28	0.13	0.28
3358	Ar2	0.57	0.22	0.19	0.18	0.05	0.28	0.11	0.23
3593	Er	0.06	0.32	0.03	0.21	0.01	0.22	0.19	0.36
3360	Haer	0.51	0.22	0.19	0.2	0.04	0.28	0.13	0.23
3361	Ar2	0.48	0.45	0.24	0.17	0.04	0.22	0.11	0.46
3594	Er	0.09	0.43	0.06	0.2	0.02	0.22	0.17	0.25
3363	Ar2	0.49	0.88	0.25	0.19	0.03	0.24	0.11	0.2
3364	Ar2	0.58	0.56	0.12	0.16	0.03	0.19	0.13	0.48
3365	Ar2	0.47	0.22	0.22	0.19	0.04	0.26	0.11	0.19
3366	Roar	0.35	0.56	0.23	0.16	0.06	0.26	0.06	0.28
3367	Haer	0.39	0.15	0.21	0.17	0.04	0.24	0.09	0.27
3368	Ar2	0.47	0.17	0.23	0.17	0.04	0.26	0.11	0.21
3595	Er	0.15	0.19	0.05	0.2	0.02	0.24	0.17	0.2
3370	Ar2	0.32	0.65	0.18	0.17	0.03	0.22	0.11	0.36
3371	Ar2	0.32	0.15	0.25	0.19	0.04	0.24	0.13	0.23
3372	Ar2	0.52	0.71	0.09	0.17	0.02	0.22	0.13	0.52
3373	Ar2	0.32	0.82	0.15	0.13	0.04	0.19	0.11	0.6
3374	Ar2	0.29	0.71	0.2	0.17	0.03	0.24	0.11	0.39
3375	Roar	0.38	0.75	0.11	0.16	0.04	0.22	0.09	0.49
3376	Ar2	0.28	0.54	0.13	0.17	0.03	0.22	0.11	0.46
3377	Roar	0.31	0.8	0.12	0.18	0.02	0.22	0.13	0.44
3598	Er	0.1	0.43	0.06	0.18	0.04	0.22	0.13	0.36
3379	Roar	0.27	1.01	0.13	0.17	0.04	0.24	0.11	0.34
3380	Roar	0.29	0.24	0.13	0.2	0.05	0.28	0.13	0.21
3381	Ar2	0.32	0.69	0.09	0.18	0.05	0.3	0.11	0.26
3382	Ar2	0.28	0.88	0.14	0.19	0.02	0.26	0.17	0.27
3599	Er	0.11	0.54	0.05	0.2	0.02	0.22	0.17	0.32
3384	Ar2	0.24	0.88	0.1	0.18	0.04	0.28	0.15	0.24
3385	Ar2	0.43	0.19	0.14	0.2	0.04	0.3	0.11	0.22
3386	Haer	0.28	0.15	0.2	0.2	0.04	0.3	0.11	0.2
3387	Roar	0.37	0.13	0.15	0.2	0.05	0.28	0.11	0.23
3388	Roar	0.25	0.58	0.12	0.16	0.06	0.28	0.09	0.18
3389	Ar2	0.43	0.24	0.16	0.2	0.04	0.26	0.15	0.24
3390	Er	0.24	0.22	0.27	0.21	0.02	0.28	0.17	0.49
3391	Ar2	0.51	0.82	0.09	0.21	0.04	0.26	0.13	0.34
3392	Ar2	0.39	0.22	0.19	0.2	0.03	0.24	0.15	0.38
3393	Roar	0.3	0.75	0.12	0.2	0.06	0.28	0.02	0.47
3394	Ar2	0.34	0.69	0.12	0.22	0.02	0.24	0.17	0.52
3395	Ar2	0.41	0.97	0.08	0.18	0.03	0.26	0.13	0.56
3396	Roar	0.32	0.75	0.16	0.19	0.04	0.26	0.13	0.37
3397	Ar2	0.3	0.17	0.11	0.22	0.04	0.26	0.13	0.19
3398	Ar2	0.21	0.19	0.19	0.2	0.05	0.26	0.13	0.16
3399	Ar2	0.51	0.15	0.06	0.19	0.05	0.26	0.13	0.2
3400	Ar2	0.29	0.15	0.18	0.21	0.04	0.28	0.15	0.19
3401	Ar2	0.22	0.22	0.17	0.21	0.02	0.24	0.15	0.19

3402	Roar	0.27	0.24	0.09	0.2	0.05	0.26	0.09	0.18
3403	Roar	0.31	0.17	0.13	0.19	0.04	0.28	0.13	0.18
3404	Haer	0.32	0.24	0.06	0.21	0.03	0.26	0.15	0.21
3405	Ar2	0.23	0.26	0.14	0.22	0.03	0.26	0.17	0.19
3406	Ar2	0.32	0.15	0.06	0.16	0.04	0.24	0.09	0.22
3407	Er	0.29	0.45	0.08	0.16	0.03	0.24	0.13	0.2
3408	Ar2	0.41	0.39	0.02	0.18	0.03	0.19	0.13	0.28
3409	Er	0.22	0.17	0.18	0.18	0.02	0.22	0.15	0.21
3410	Roar	0.19	0.26	0.15	0.17	0.03	0.22	0.13	0.19
3411	Er	0.39	0.39	0.11	0.19	0.01	0.22	0.17	0.29
3412	Er	0.27	0.37	0.17	0.17	0.03	0.19	0.13	0.32
3413	Ar2	0.27	0.15	0.22	0.17	0.04	0.24	0.11	0.16
3414	Er	0.36	0.15	0.18	0.17	0.02	0.22	0.15	0.2
3415	Er	0.46	0.13	0.09	0.16	0.03	0.24	0.13	0.18
3416	Haer	0.52	0.19	0.13	0.18	0.04	0.28	0.09	0.17
3417	Ar2	0.4	0.15	0.12	0.15	0.05	0.26	0.09	0.14
3418	Er	0.58	0.34	0.06	0.17	0.02	0.22	0.15	0.25
3419	Er	0.36	0.39	0.17	0.18	0.02	0.22	0.13	0.31
3420	Haer	0.38	0.19	0.2	0.18	0.03	0.24	0.15	0.17
3421	Ar2	0.24	0.19	0.16	0.19	0.02	0.24	0.17	0.22
3422	Ar2	0.19	0.39	0.08	0.15	0.07	0.26	0.06	0.18
3423	Ar2	0.26	0.15	0.09	0.19	0.04	0.26	0.13	0.2
3424	Er	0.26	0.41	0.07	0.19	0.03	0.22	0.15	0.23
3425	Ar2	0.24	0.19	0.07	0.18	0.04	0.26	0.13	0.19
3600	Er	0.13	0.45	0.03	0.17	0.04	0.24	0.13	0.36
3427	Roar	0.31	0.65	0.12	0.17	0.03	0.22	0.13	0.54
3610	Er	0.12	0.39	0.04	0.21	0.02	0.24	0.19	0.24
3429	Roar	0.28	0.6	0.13	0.19	0.03	0.24	0.13	0.58
3430	Ar2	0.29	0.22	0.22	0.22	0.02	0.24	0.17	0.23
3431	Ar2	0.31	0.15	0.15	0.19	0.04	0.28	0.13	0.19
3618	Ar2	0.23	0.73	0.08	0.18	0.03	0.22	0.11	0.69
3433	Roar	0.24	0.54	0.09	0.2	0.02	0.26	0.15	0.37
3434	Roar	0.26	0.6	0.09	0.17	0.03	0.24	0.13	0.43
3619	Ar2	0.43	0.24	0.09	0.2	0.04	0.28	0.15	0.63
3620	Ar2	0.49	0.17	0.12	0.2	0.04	0.26	0.13	0.46
3437	Ar2	0.41	0.15	0.2	0.18	0.03	0.22	0.13	0.22
3438	Haer	0.35	0.43	0.08	0.17	0.03	0.22	0.11	0.27
3439	Ar2	0.26	0.17	0.2	0.18	0.03	0.24	0.11	0.19
3622	Er	0.12	0.45	0.06	0.22	0.01	0.24	0.22	0.35
3441	Ar2	0.49	0.32	0.07	0.18	0.02	0.22	0.15	0.31
3442	Ar2	0.26	0.19	0.17	0.18	0.05	0.28	0.09	0.17
3443	Ar2	0.57	0.28	0.08	0.22	0.04	0.26	0.15	0.26
3444	Ar2	0.47	0.69	0.1	0.24	0.02	0.28	0.17	0.3
3445	Ar2	0.33	0.24	0.23	0.21	0.04	0.26	0.15	0.2
3446	Ar2	0.25	0.54	0.21	0.2	0.04	0.28	0.13	0.25
3623	Ar2	0.08	0.73	0.03	0.2	0.01	0.22	0.19	0.57
3448	Er	0.4	0.15	0.2	0.17	0.05	0.28	0.11	0.18
3449	Ar2	0.46	0.17	0.14	0.18	0.05	0.26	0.11	0.17
3450	Ar2	0.9	0.24	0.03	0.23	0.03	0.28	0.17	0.24
3451	Haer	0.55	0.32	0.14	0.22	0.04	0.28	0.11	0.26
3452	Ar2	0.8	0.24	0.06	0.21	0.04	0.26	0.13	0.2
3626	Er	0.09	0.99	0.04	0.2	0.03	0.26	0.17	0.33
3454	Ar2	0.4	0.73	0.3	0.25	0.02	0.26	0.22	0.41
3455	Ar2	0.6	0.78	0.17	0.2	0.06	0.3	0.11	0.28

3456	Er	0.66	0.75	0.08	0.2	0.06	0.32	0.11	0.39
3457	Roar	0.45	0.6	0.18	0.23	0.04	0.28	0.15	0.51
3629	Ar2	0.1	0.93	0.04	0.18	0.03	0.26	0.11	0.34
3631	Ar2	0.23	1.49	0.14	0.23	0.02	0.26	0.17	0.67
3460	Ar2	0.43	0.52	0.19	0.25	0.02	0.26	0.22	0.41
3632	Ar2	0.25	1.57	0.14	0.24	0.01	0.28	0.22	0.4
3462	Ar2	0.57	0.54	0.07	0.24	0.04	0.37	0.15	0.37
3645	Ar2	0.21	0.3	0.18	0.16	0.02	0.22	0.15	0.33
3464	Ar2	0.68	0.37	0.13	0.22	0.08	0.41	0.13	0.21
3465	Ar2	0.41	0.26	0.36	0.25	0.03	0.28	0.19	0.28
3466	Ar2	0.56	0.34	0.26	0.24	0.03	0.28	0.19	0.32
3467	Ar2	0.62	0.24	0.21	0.22	0.04	0.3	0.17	0.2
3468	Ar2	0.46	0.37	0.25	0.24	0.03	0.28	0.17	0.32
3469	Ar2	1.02	0.19	0.06	0.22	0.05	0.3	0.13	0.31
3470	Ar2	1.01	0.6	0.11	0.24	0.04	0.28	0.15	0.32
3471	Ar2	0.84	0.43	0.12	0.24	0.05	0.3	0.17	0.28
3472	Ar2	0.48	0.73	0.34	0.25	0.02	0.26	0.22	0.39
3473	Ar2	0.71	0.75	0.22	0.24	0.05	0.34	0.13	0.32
3474	Roar	0.37	0.24	0.17	0.19	0.06	0.26	0.11	0.17
3475	Ar2	0.51	0.47	0.18	0.21	0.02	0.24	0.17	0.33
3476	Ar2	0.6	0.19	0.16	0.2	0.04	0.26	0.13	0.19
3477	Er	0.54	0.24	0.26	0.23	0.02	0.26	0.15	0.23
3478	Haer	0.63	0.26	0.15	0.22	0.05	0.32	0.06	0.19
3479	Ar2	0.42	0.24	0.21	0.18	0.04	0.28	0.13	0.16
3480	Haer	0.69	0.22	0.08	0.2	0.04	0.26	0.13	0.17
3481	Haer	0.55	0.22	0.15	0.2	0.04	0.26	0.11	0.16
3482	Ar2	0.78	0.32	0.07	0.3	0.03	0.32	0.24	0.37
3483	Ar2	0.59	0.32	0.18	0.26	0.05	0.32	0.17	0.23
3484	Haer	0.58	0.26	0.17	0.24	0.05	0.34	0.17	0.21
3485	Er	0.3	0.45	0.24	0.25	0.04	0.3	0.17	0.38
3486	Ar2	0.36	0.47	0.23	0.25	0.03	0.3	0.17	0.35
3653	Ar2	0.38	0.88	0.05	0.17	0.04	0.22	0.09	0.55
3488	Ar2	0.49	0.28	0.22	0.21	0.06	0.32	0.11	0.18
3489	Haer	0.6	0.28	0.13	0.21	0.05	0.34	0.13	0.19
3490	Ar2	0.32	0.28	0.21	0.22	0.07	0.34	0.11	0.21
3491	Ar2	0.22	0.62	0.12	0.26	0.03	0.34	0.19	0.51
3660	Ar2	0.5	0.84	0.12	0.13	0.02	0.15	0.11	0.36
3493	Ar2	0.22	0.65	0.13	0.26	0.07	0.34	0.15	0.37
3670	Er	0.09	0.67	0.02	0.15	0.03	0.19	0.13	0.39
3495	Ar2	0.33	0.65	0.11	0.32	0.03	0.34	0.22	0.41
3671	Er	0.07	0.71	0.05	0.18	0.01	0.19	0.17	0.45
3497	Roar	0.23	0.8	0.07	0.26	0.03	0.3	0.19	0.47
3672	Er	0.06	0.73	0.02	0.16	0.02	0.19	0.15	0.49
3674	Er	0.13	0.32	0.05	0.16	0.02	0.19	0.13	0.33
3500	Er	0.09	0.82	0.06	0.26	0.02	0.28	0.24	0.5
3675	Er	0.14	0.6	0.05	0.15	0.02	0.17	0.13	0.29
3502	Ar2	0.33	0.73	0.1	0.25	0.04	0.3	0.17	0.47
3503	Roar	0.26	0.95	0.08	0.23	0.05	0.32	0.15	0.25
3504	Ar2	0.28	0.88	0.19	0.27	0.04	0.32	0.19	0.36
3505	Ar2	0.28	0.26	0.23	0.23	0.05	0.32	0.19	0.48
3506	Ar2	0.24	0.26	0.22	0.25	0.02	0.3	0.22	0.48
3676	Er	0.09	0.75	0.03	0.16	0.01	0.17	0.15	0.34
3508	Roar	0.24	0.75	0.09	0.25	0.05	0.3	0.15	0.68
3509	Er	0.19	0.52	0.11	0.21	0.07	0.28	0.06	0.43

3510	Ar2	0.26	0.69	0.21	0.23	0.03	0.28	0.17	0.37
3511	Ar2	0.3	0.69	0.16	0.22	0.01	0.26	0.19	0.54
3677	Er	0.11	0.73	0.03	0.16	0.02	0.19	0.15	0.37
3692	Ar2	0.29	0.65	0.07	0.17	0.02	0.22	0.13	0.37
3514	Ar2	0.3	0.24	0.13	0.24	0.04	0.32	0.17	0.24
3515	Ar2	0.35	0.47	0.06	0.25	0.03	0.28	0.19	0.34
3516	Ar2	0.33	0.47	0.05	0.25	0.02	0.26	0.22	0.42
3694	Er	0.19	0.65	0.15	0.17	0.01	0.17	0.15	0.4
3696	Er	0.22	0.32	0.11	0.16	0.02	0.19	0.13	0.3
3697	Er	0.3	0.17	0.13	0.18	0.01	0.19	0.15	0.22
3698	Er	0.15	0.54	0.1	0.17	0.01	0.19	0.15	0.43
3521	Ar2	0.16	0.45	0.07	0.21	0.01	0.24	0.19	0.41
3699	Er	0.22	0.47	0.09	0.17	0.02	0.19	0.13	0.35
3701	Er	0.21	0.67	0.13	0.17	0.01	0.17	0.15	0.43
3524	Ar2	0.12	0.22	0.03	0.22	0.03	0.26	0.15	0.27
3702	Er	0.17	0.58	0.12	0.15	0.02	0.19	0.13	0.46
3703	Er	0.23	0.71	0.12	0.17	0.01	0.17	0.15	0.36
3704	Ar2	0.2	0.75	0.1	0.18	0.02	0.19	0.11	0.38
3708	Er	0.23	0.45	0.08	0.15	0.02	0.19	0.11	0.35
3709	Er	0.15	0.3	0.11	0.16	0.03	0.19	0.09	0.36
3710	Er	0.14	0.5	0.08	0.15	0.02	0.17	0.13	0.37
3712	Er	0.2	0.71	0.06686	0.15	0.03	0.22	0.09	0.33
3532	Ar2	0.18	1.16	0.06	0.22	0.02	0.26	0.22	0.28
3533	Ar2	0.28	0.17	0.06	0.25	0.03	0.28	0.22	0.22
3714	Er	0.11	0.65	0.07	0.18	0.05	0.22	0.09	0.42
3535	Er	0.1	0.69	0.03	0.23	0.03	0.26	0.17	0.43
3724	Ar2	0.33	0.67	0.13	0.2	0.01	0.22	0.17	0.52
3726	Ar2	0.24	0.69	0.12	0.2	0.02	0.24	0.15	0.44
3735	Ar2	0.22	0.69	0.04	0.2	0.02	0.22	0.15	0.43
3737	Er	0.08	0.67	0.03	0.2	0.01	0.22	0.19	0.34
3741	Er	0.34	0.65	0.03	0.15	0.02	0.17	0.09	0.51
3747	Er	0.38	0.8	0.13	0.12	0.02	0.15	0.09	0.38
3748	Er	0.33	0.84	0.15	0.14	0.01	0.15	0.11	0.33
3749	Er	0.3	0.6	0.24	0.14	0.02	0.17	0.09	0.3
3544	Er	0.09	0.26	0.07	0.28	0.02	0.3	0.26	0.35
3545	Er	0.07	0.47	0.03	0.25	0.02	0.28	0.24	0.35
3546	Er	0.09	0.26	0.06	0.28	0.03	0.34	0.26	0.41
3757	Ar2	0.44	0.86	0.1	0.13	0.02	0.15	0.09	0.44
3548	Er	0.24	0.5	0.07	0.25	0.02	0.28	0.22	0.31
3549	Er	0.16	0.26	0.14	0.26	0.02	0.28	0.22	0.32
3759	Er	0.14	0.71	0.08	0.15	0.01	0.15	0.13	0.5
3551	Er	0.16	0.37	0.07	0.25	0.02	0.28	0.22	0.34
3552	Ar2	0.31	0.22	0.05	0.23	0.02	0.26	0.22	0.22
3553	Ar2	0.36	0.22	0.05	0.24	0.02	0.26	0.22	0.23
3554	Roar	0.32	0.26	0.06	0.23	0.07	0.37	0.11	0.19
3555	Ar2	0.3	0.26	0.2	0.26	0.05	0.37	0.17	0.23
3556	Ar2	0.24	0.41	0.08	0.26	0.07	0.32	0.13	0.3
3557	Roar	0.23	0.41	0.16	0.26	0.05	0.3	0.17	0.33
3558	Er	0.12	0.34	0.11	0.31	0.04	0.34	0.24	0.27
3559	Ar2	0.19	0.34	0.17	0.29	0.04	0.32	0.22	0.28
3560	Er	0.22	0.3	0.08	0.26	0.06	0.34	0.15	0.23
3561	Ar2	0.26	0.65	0.04	0.22	0.01	0.26	0.22	0.24
3769	Ar2	0.23	0.88	0.08	0.16	0.02	0.19	0.11	0.33
3772	Er	0.23	0.86	0.14	0.13	0.01	0.17	0.11	0.52

3564	Er	0.13	0.22	0.03	0.23	0.02	0.26	0.22	0.26
3774	Ar2	0.32	0.75	0.08	0.16	0.02	0.17	0.11	0.52
3792	eee	2.6	0.09	2.13	0.25	0.28	0.75	0.02	0.18
3795	Ar2	1.96	1.64	1.44	0.57	0.17	0.78	0.19	0.97
4056	Ar2	1.02	0.06	0.69	0.14	0.03	0.17	0.09	0.29
4061	Er	0.85	0.75	0.65	0.16	0.02	0.19	0.11	0.43
4090	Er	1.06	0.78	0.48	0.12	0.01	0.13	0.06	0.38
3571	Er	0.09	0.45	0.06	0.21	0.03	0.28	0.19	0.35
3572	Roar	0.12	0.24	0.08	0.22	0.05	0.28	0.15	0.21
3573	Ar2	0.2	0.28	0.08	0.24	0.05	0.28	0.17	0.22
3574	Ar2	0.27	0.22	0.06	0.23	0.02	0.26	0.22	0.23
3575	Ar2	0.29	0.43	0.06	0.22	0.01	0.26	0.22	0.27
4153	Ar2	1.03	0.19	0.99	0.18	0.04	0.22	0.09	0.34
3577	Ar2	0.25	0.22	0.14	0.22	0.01	0.26	0.22	0.24
3578	Ar2	0.4	0.65	0.06	0.23	0.02	0.26	0.22	0.24
3579	Ar2	0.39	0.43	0.04	0.23	0.02	0.26	0.22	0.33
3580	Ar2	0.34	0.43	0.04	0.22	0.02	0.26	0.22	0.28
3581	Ar2	0.43	0.22	0.04	0.24	0.04	0.3	0.19	0.24
3582	Ar2	0.37	0.47	0.05	0.25	0.02	0.26	0.22	0.33
3583	Ar2	0.36	0.47	0.05	0.24	0.02	0.26	0.22	0.35
3584	Er	0.23	0.41	0.13	0.22	0.03	0.3	0.19	0.4
4164	Er	1.22	0.93	0.8	0.23	0.02	0.26	0.19	0.43
3586	Ar2	0.27	0.43	0.07	0.22	0	0.22	0.22	0.26
4166	Ar2	1.02	1.94	0.81	0.19	0.04	0.24	0.15	1.08
4167	Ar2	1.44	1.72	0.46	0.22	0.05	0.32	0.06	1.48
3589	Er	0.14	0.45	0.06	0.2	0.02	0.22	0.17	0.26
3590	Ar2	0.1	0.52	0.07	0.19	0.01	0.22	0.17	0.33
4169	Ar2	0.91	2	0.7	0.23	0.04	0.28	0.11	1.41
4170	Ar2	1.3	2.15	0.5	0.22	0.05	0.3	0.11	1.13
4171	Ar2	1.25	2.13	0.61	0.24	0.07	0.34	0.09	1.18
4173	Ar2	1.08	2.45	0.7	0.25	0.04	0.32	0.13	1.35
4174	Ar2	1.06	2.33	0.8	0.24	0.04	0.3	0.11	1.79
3596	Er	0.09	0.17	0.08	0.19	0.03	0.24	0.15	0.22
3597	Ar2	0.2	0.15	0.05	0.2	0.04	0.26	0.15	0.29
4175	Ar2	1.38	1.92	0.65	0.25	0.06	0.34	0.13	1.69
4181	Ar2	1.69	2.3	0.61	0.22	0.03	0.3	0.13	1.39
4182	Ar2	2.35	2.13	0.48	0.22	0.02	0.24	0.15	1.87
3601	Er	0.1	0.47	0.07	0.23	0.02	0.28	0.19	0.25
3602	Er	0.07	0.32	0.05	0.22	0.01	0.24	0.22	0.32
3603	Er	0.12	0.45	0.04	0.24	0.01	0.26	0.22	0.32
3604	Ar2	0.16	0.26	0.03	0.26	0	0.26	0.26	0.25
3605	Er	0.06	0.47	0.04	0.24	0.01	0.26	0.24	0.24
3606	Er	0.13	0.24	0.09	0.22	0.02	0.24	0.19	0.22
3607	Ar2	0.14	0.19	0.09	0.21	0.03	0.26	0.17	0.21
3608	Er	0.08	0.17	0.06	0.19	0.03	0.24	0.17	0.29
3609	Er	0.1	0.41	0.04	0.2	0.02	0.24	0.17	0.28
4183	Ar2	1.63	1.64	0.96	0.23	0.05	0.32	0.09	1.41
3611	Er	0.07	0.37	0.05	0.19	0.03	0.24	0.17	0.34
3612	Ar2	0.21	1.08	0.12	0.21	0.03	0.24	0.09	0.75
3613	Ar2	0.35	1.06	0.11	0.17	0.02	0.19	0.13	0.91
3614	Ar2	0.31	1.27	0.1	0.18	0.03	0.24	0.13	0.91
3615	Ar2	0.19	1.18	0.1	0.2	0.03	0.24	0.15	0.72
3616	Ar2	0.3	1.53	0.21	0.19	0.03	0.24	0.15	0.37
3617	Ar2	0.35	1.14	0.17	0.18	0.03	0.26	0.11	0.56

4184	Ar2	1.78	2.09	0.76	0.25	0.03	0.3	0.17	1.54
4185	Ar2	1.49	1.89	0.95	0.23	0.08	0.39	0.06	1.4
4186	Ar2	1.4	1.57	0.99	0.24	0.05	0.34	0.15	1.52
3621	Ar2	0.19	0.15	0.2	0.19	0.04	0.26	0.11	0.6
4187	Ar2	1.76	2.17	0.74	0.28	0.05	0.39	0.13	1.34
4188	Ar2	1.47	0.99	0.61	0.32	0.07	0.5	0.17	0.84
3624	Ar2	0.13	1.21	0.09	0.19	0.02	0.24	0.17	0.35
3625	Ar2	0.1	1.18	0.02	0.21	0.01	0.22	0.19	0.45
4189	Ar2	1.32	0.9	0.77	0.36	0.06	0.56	0.22	0.65
3627	Ar2	0.09	0.99	0.04	0.2	0.03	0.26	0.17	0.33
3628	Haer	0.34	0.47	0.06	0.14	0.04	0.19	0.06	0.31
4194	Ar2	1.24	1.25	0.7	0.26	0.08	0.47	0.11	0.61
3630	Ar2	0.14	1.31	0.06	0.15	0.05	0.19	0.09	0.62
4199	Ar2	1.44	1.42	1.08	0.3	0.04	0.37	0.17	0.95
4201	Ar2	1.34	2.05	0.58	0.27	0.08	0.41	0.13	1.28
3633	Ar2	0.15	1.25	0.05	0.17	0.03	0.19	0.13	1.2
3634	Ar2	0.11	1.34	0.08	0.14	0.03	0.17	0.09	1.28
3635	Ar2	0.32	1.7	0.05	0.17	0.02	0.24	0.15	0.63
3636	Ar2	0.11	0.86	0.04	0.15	0.03	0.17	0.09	0.22
3637	Ar2	0.12	0.56	0.06	0.12	0.02	0.15	0.11	0.41
3638	Ar2	0.08	0.5	0.05	0.13	0.02	0.15	0.11	0.46
3639	Er	0.15	0.41	0.07	0.14	0.01	0.15	0.13	0.36
3640	Ar2	0.2	0.15	0.09	0.16	0.03	0.19	0.09	0.23
3641	Ar2	0.46	0.22	0.1	0.1	0.02	0.13	0.06	0.21
3642	Ar2	0.44	0.65	0.07	0.18	0.03	0	0	0.42
3643	Er	0.08	0.11	0.05	0.08	0.02	0.11	0.06	0.15
3644	Ar2	0.17	0.09	0.06	0.08	0.02	0.11	0.04	0.08
4202	Ar2	1.06	2.17	0.9	0.34	0.09	0.45	0.13	0.65
3646	Ar2	0.21	0.3	0.18	0.16	0.02	0.22	0.15	0.33
3647	Ar2	0.18	1.12	0.11	0.16	0.02	0.19	0.13	0.4
3648	Ar2	0.25	1.08	0.08	0.17	0.02	0.19	0.13	0.46
3649	Ar2	0.14	0.88	0.1	0.17	0.03	0.22	0.11	0.49
3650	Ar2	0.36	0.19	0.19	0.2	0.02	0.24	0.13	0.26
3651	Ar2	0.38	0.19	0.12	0.17	0.04	0.22	0.11	0.36
3652	Ar2	0.46	1.14	0.05	0.18	0.02	0.24	0.13	0.44
4203	Ar2	1.21	1.98	0.83	0.25	0.13	0.45	0.06	0.98
3654	Ar2	0.37	1.18	0.08	0.18	0.03	0.22	0.11	0.29
3655	Ar2	0.37	1.06	0.09	0.13	0.03	0.19	0.09	0.69
3656	Roar	0.3	1.01	0.14	0.14	0.05	0.22	0.06	0.46
3657	Ar2	0.41	0.45	0.25	0.13	0.02	0.15	0.09	0.4
3658	Ar2	0.47	0.78	0.16	0.13	0.03	0.15	0.04	0.53
3659	Ar2	0.47	0.71	0.17	0.12	0.02	0.17	0.09	0.33
4204	Ar2	1.17	0.39	0.94	0.35	0.07	0.41	0.17	1.02
3661	Ar2	0.45	0.73	0.11	0.14	0.01	0.15	0.11	0.55
3662	Ar2	0.35	1.12	0.13	0.12	0.02	0.17	0.09	0.49
3663	Roar	0.31	1.18	0.14	0.12	0.02	0.17	0.09	0.48
3664	Er	0.11	1.12	0.04	0.11	0.03	0.13	0.04	0.49
3665	Ar2	0.25	1.21	0.09	0.14	0.02	0.17	0.11	0.36
3666	Haer	0.14	0.17	0.13	0.13	0.05	0.22	0.06	0.31
3667	Ar2	0.57	0.11	0.11	0.16	0.04	0.24	0.09	0.23
3668	Ar2	0.33	0.13	0.09	0.16	0.02	0.22	0.09	0.58
3669	Ar2	0.1	0.37	0.18	0.16	0.03	0.19	0.09	0.73
4206	Ar2	1.05	1.34	0.72	0.3	0.11	0.45	0.09	1.03
4207	Ar2	1.08	1.46	0.69	0.31	0.08	0.43	0.13	1.09

4210	Ar2	1.12	1.85	0.89	0.35	0.06	0.5	0.19	0.99
3673	Ar2	0.17	0.15	0.06	0.17	0.03	0.22	0.11	0.18
4212	Ar2	1.53	2.13	0.96	0.36	0.06	0.47	0.19	1.24
4214	Ar2	2.42	2.13	0.44	0.34	0.05	0.45	0.24	1.45
4215	Ar2	1.66	2.2	1.01	0.36	0.08	0.47	0.19	0.78
4217	Ar2	1.36	2.11	0.82	0.29	0.08	0.41	0.09	1.31
3678	Roar	0.24	0.13	0.05	0.14	0.03	0.19	0.09	0.19
3679	Er	0.28	0.15	0.03	0.15	0.03	0.22	0.09	0.16
3680	Ar2	0.15	0.17	0.09	0.16	0.02	0.19	0.15	0.2
3681	Ar2	0.14	0.26	0.09	0.15	0.02	0.19	0.13	0.18
3682	Roar	0.26	0.56	0.11	0.14	0.03	0.19	0.09	0.55
3683	Ar2	0.28	0.5	0.12	0.15	0.03	0.19	0.09	0.39
3684	Roar	0.38	0.13	0.11	0.15	0.04	0.24	0.09	0.17
3685	Er	0.22	0.34	0.16	0.17	0.04	0.22	0.11	0.23
3686	Ar2	0.36	0.19	0.11	0.17	0.03	0.24	0.11	0.22
3687	Ar2	0.45	0.78	0.05	0.18	0.02	0.22	0.13	0.39
3688	Roar	0.37	0.58	0.12	0.16	0.03	0.22	0.11	0.46
3689	Roar	0.35	0.56	0.07	0.16	0.05	0.22	0.06	0.41
3690	Er	0.35	0.13	0.07	0.16	0.04	0.26	0.13	0.22
3691	Ar2	0.27	0.62	0.11	0.16	0.04	0.24	0.11	0.28
4219	Ar2	1.86	1.96	0.87	0.25	0.06	0.39	0.15	1.07
3693	Ar2	0.23	0.75	0.12	0.18	0.03	0.24	0.11	0.2
4220	Ar2	1.23	1.51	0.92	0.25	0.1	0.39	0.04	1.33
3695	Ar2	0.36	0.6	0.1	0.16	0.01	0.17	0.15	0.31
4221	Ar2	0.89	1.72	0.66	0.23	0.05	0.34	0.13	0.83
4224	Ar2	0.9	1.96	0.66	0.24	0.04	0.3	0.13	0.52
4225	Ar2	0.85	2.22	0.67	0.22	0.04	0.28	0.13	0.9
4226	Ar2	0.92	1.81	0.51	0.23	0.02	0.28	0.17	0.91
3700	Ar2	0.22	0.47	0.09	0.17	0.02	0.19	0.13	0.35
4229	Ar2	1.22	1.77	0.34	0.24	0.05	0.37	0.13	0.86
4230	Ar2	0.78	1.38	0.66	0.22	0.05	0.32	0.13	1.15
4232	Ar2	0.91	2.11	0.57	0.23	0.04	0.3	0.13	0.76
4234	Ar2	0.98	1.46	0.4	0.24	0.05	0.32	0.13	0.74
3705	Er	0.18	0.71	0.11	0.17	0.02	0.19	0.13	0.31
3706	Er	0.14	0.47	0.075011	0.15	0.02	0.19	0.13	0.38
3707	Haer	0.26	0.24	0.05	0.14	0.04	0.22	0.09	0.28
4235	Ar2	1.17	1.7	0.25	0.24	0.05	0.3	0.11	1.09
4239	Ar2	0.81	1.81	0.47	0.19	0.05	0.28	0.09	0.92
4240	Ar2	0.82	1.57	0.51	0.22	0.02	0.26	0.17	1.03
3711	Ar2	0.3	0.45	0.04	0.15	0.01	0.22	0.15	0.3
4243	Ar2	0.88	1.27	0.57	0.23	0.06	0.3	0.09	0.82
3713	Roar	0.22	0.56	0.06	0.15	0.04	0.24	0.09	0.32
4247	Ar2	0.94	1.64	0.6	0.24	0.07	0.32	0.09	0.61
3715	Ar2	0.31	0.37	0.12	0.19	0.03	0.24	0.13	0.25
3716	Ar2	0.29	0.62	0.08	0.17	0.04	0.26	0.11	0.27
3717	Haer	0.32	0.19	0.04	0.19	0.03	0.28	0.11	0.27
3718	Roar	0.32	0.22	0.1	0.19	0.03	0.26	0.11	0.26
3719	Er	0.21	0.15	0.17	0.19	0.04	0.24	0.13	0.26
3720	Ar2	0.37	0.19	0.08	0.18	0.04	0.26	0.13	0.2
3721	Ar2	0.42	0.26	0.05	0.19	0.04	0.24	0.13	0.29
3722	Ar2	0.22	0.45	0.16	0.18	0.02	0.24	0.15	0.53
3723	Roar	0.23	0.88	0.11	0.15	0.06	0.28	0.06	0.23
4253	Ar2	0.94	1.57	0.66	0.22	0.03	0.28	0.13	0.71
3725	Ar2	0.22	0.9	0.09	0.15	0.05	0.19	0.09	0.61

4255	Ar2	0.88	1.96	0.51	0.21	0.07	0.34	0.06	0.71
3727	Ar2	0.25	0.52	0.11	0.18	0.02	0.22	0.15	0.52
3728	Ar2	0.23	0.84	0.1	0.17	0.03	0.22	0.11	0.4
3729	Roar	0.23	0.71	0.05	0.16	0.05	0.22	0.06	0.57
3730	Roar	0.33	0.93	0.13	0.17	0.03	0.22	0.13	0.4
3731	Roar	0.36	0.86	0.07	0.18	0.03	0.22	0.11	0.58
3732	Ar2	0.15	0.99	0.09	0.17	0.03	0.22	0.11	0.22
3733	Ar2	0.14	0.82	0.11	0.19	0.01	0.19	0.17	0.36
3734	Haer	0.2	0.45	0.04	0.16	0.04	0.28	0.13	0.28
4256	Ar2	1.03	1.23	0.53	0.2	0.04	0.3	0.13	0.94
4258	Ar2	0.88	2.13	0.4	0.29	0.03	0.32	0.17	1.67
4259	Ar2	0.94	1.94	0.45	0.26	0.05	0.37	0.17	1.51
3738	Ar2	0.09	1.18	0.04	0.16	0.03	0.22	0.11	0.18
3739	Ar2	0.38	0.15	0.08	0.16	0.03	0.22	0.11	0.19
3740	Roar	0.25	0.75	0.1	0.12	0.04	0.19	0.06	0.24
4261	Ar2	0.8	1.96	0.5	0.29	0.07	0.39	0.09	1.47
3742	Roar	0.2	0.58	0.11	0.12	0.03	0.17	0.06	0.55
3743	Roar	0.33	0.71	0.08	0.13	0.03	0.17	0.09	0.3
3744	Roar	0.22	0.54	0.13	0.13	0.04	0.19	0.06	0.24
3745	Ar2	0.36	0.67	0.07	0.1	0.04	0.19	0.06	0.23
3746	Ar2	0.41	0.86	0.12	0.13	0.02	0.15	0.09	0.5
4262	Ar2	0.85	2.02	0.51	0.26	0.06	0.41	0.13	1.23
4263	Ar2	1	1.98	0.36	0.27	0.07	0.37	0.13	1.24
4264	Ar2	0.97	1.81	0.43	0.27	0.08	0.37	0.11	1.13
3750	Haer	0.37	0.19	0.19	0.14	0.04	0.19	0.06	0.19
3751	Ar2	0.3	0.8	0.11	0.09	0.01	0.11	0.06	0.41
3752	Ar2	0.33	0.32	0.16	0.11	0.01	0.13	0.09	0.23
3753	Ar2	0.34	0.09	0.11	0.1	0.02	0.15	0.06	0.45
3754	Ar2	0.31	1.03	0.1	0.13	0.02	0.15	0.09	0.31
3755	Ar2	0.33	0.34	0.12	0.13	0.02	0.15	0.11	0.3
3756	Ar2	0.32	0.28	0.23	0.13	0.02	0.17	0.11	0.34
4265	Ar2	0.84	1.68	0.67	0.29	0.05	0.37	0.19	1.09
3758	Ar2	0.37	1.03	0.1	0.13	0.02	0.15	0.09	0.39
4266	Ar2	0.88	1.64	0.54	0.28	0.05	0.32	0.17	1.55
3760	Er	0.16	0.39	0.09	0.18	0.03	0.22	0.11	0.28
3761	Ar2	0.21	0.19	0.14	0.17	0.02	0.19	0.11	0.27
3762	Ar2	0.26	0.17	0.07	0.17	0.04	0.22	0.09	0.23
3763	Haer	0.34	0.17	0.05	0.16	0.03	0.24	0.09	0.21
3764	Ar2	0.36	0.69	0.12	0.16	0.02	0.19	0.13	0.45
3765	Ar2	0.34	0.97	0.13	0.14	0.02	0.19	0.11	0.45
3766	Ar2	0.43	0.17	0.11	0.18	0.03	0.24	0.11	0.28
3767	Haer	0.42	0.32	0.12	0.17	0.03	0.19	0.11	0.25
3768	Ar2	0.28	0.75	0.16	0.15	0.04	0.22	0.06	0.27
4267	Ar2	0.85	1.96	0.56	0.25	0.04	0.37	0.17	1.37
3770	Ar2	0.29	0.88	0.07	0.15	0.03	0.19	0.09	0.39
3771	Ar2	0.22	0.9	0.12	0.16	0.02	0.19	0.13	0.38
4268	Ar2	0.89	2.13	0.47	0.29	0.1	0.43	0.09	1
3773	Roar	0.31	0.9	0.07	0.13	0.03	0.17	0.09	0.35
4269	Ar2	1.08	1.42	0.48	0.3	0.06	0.43	0.19	1.19
3775	Roar	0.23	1.16	0.08	0.15	0.03	0.19	0.11	0.21
3776	Ar2	2.8	0.39	1.63	0.15	0.03	0.19	0.09	0.25
3777	Ar2	2.73	0.54	1.89	0.19	0.02	0.24	0.13	0.29
3778	Ar2	2.78	2.05	1.73	0.18	0.02	0.24	0.13	0.21
3779	Ar2	2.79	0.34	1.59	0.17	0.02	0.19	0.11	0.25

3780	Ar2	2.91	0.28	1.62	0.15	0.02	0.17	0.09	0.24
3781	Er	3.26	0.3	1.04	0.18	0.05	0.28	0.11	0.26
3782	Ar2	2.23	0.78	1.73	0.2	0.03	0.24	0.11	0.21
3783	Ar2	2.06	0.15	1.9	0.18	0.04	0.26	0.13	0.21
3784	Er	2.39	0.26	2.23	0.12	0.02	0.15	0.09	0.21
3785	Er	2.57	0.28	2.07	0.14	0.02	0.17	0.09	0.27
3786	Ar2	2.62	0.41	2.15	0.13	0.03	0.17	0.06	0.28
3787	Ar2	3.2	0.5	1.63	0.1	0.02	0.15	0.04	0.22
3788	Ar2	2.69	0.32	2.31	0.17	0.02	0.22	0.13	0.27
3789	Ar2	2.69	0.41	2.37	0.15	0.02	0.17	0.09	0.35
3790	Ar2	2.77	0.52	2.42	0.15	0.02	0.19	0.09	0.31
3791	Ar2	3.14	0.5	2.12	0.16	0.03	0.19	0.06	0.39
4270	Ar2	0.94	2.09	0.49	0.29	0.07	0.39	0.15	1.33
3793	Ar2	2.24	1.23	2.06	0.39	0.12	0.54	0.13	0.41
3794	Ar2	2.49	1.94	1.8	0.32	0.11	0.5	0.13	0.59
4271	Ar2	1.06	2	0.45	0.32	0.07	0.41	0.09	1.29
3796	Ar2	1.77	0.43	1.46	0.48	0.16	0.84	0.13	0.48
3797	Ar2	2.79	0.43	1.32	0.4	0.07	0.54	0.22	0.43
3798	Ar2	2.47	1.25	2.01	0.19	0.03	0.24	0.11	1.02
3799	Ar2	2.5	1.42	2.24	0.16	0.02	0.19	0.09	0.88
3800	Ar2	3.08	1.23	1.91	0.18	0.02	0.22	0.11	0.9
3801	Ar2	2.42	0.45	2.26	0.19	0.03	0.24	0.11	0.66
3802	Ar2	2.63	2.28	2.18	0.23	0.06	0.34	0.11	1.3
3803	Ar2	2.19	1.44	1.75	0.19	0.03	0.24	0.06	1.13
3804	Ar2	3.23	1.49	1.53	0.23	0.03	0.28	0.15	1.04
3805	Ar2	2.65	1.06	1.51	0.16	0.03	0.19	0.09	0.43
3806	Ar2	2.88	0.8	1.29	0.15	0.04	0.19	0.06	0.57
3807	Ar2	1.86	0.19	1.72	0.17	0.03	0.22	0.13	0.55
3808	Ar2	2.13	0.47	1.64	0.17	0.04	0.24	0.06	0.42
3809	Er	2.01	0.39	1.64	0.13	0.02	0.17	0.09	0.38
3810	Ar2	1.99	0.65	1.73	0.14	0.02	0.17	0.09	0.35
3811	Ar2	2.34	0.54	2.01	0.16	0.02	0.19	0.11	0.57
3812	Ar2	2.29	0.95	1.91	0.15	0.03	0.19	0.09	0.57
3813	Ar2	2.1	1.23	1.77	0.19	0.04	0.24	0.06	0.6
3814	Ar2	2.23	0.43	1.78	0.18	0.03	0.22	0.06	0.49
3815	Ar2	3.15	1.51	1.05	0.16	0.03	0.19	0.04	0.54
3816	Ar2	2.1	0.22	1.79	0.15	0.03	0.19	0.11	0.38
3817	Ar2	1.99	0.88	1.91	0.2	0.02	0.22	0.13	0.58
3818	Ar2	2.12	1.34	1.62	0.13	0.03	0.17	0.09	0.89
3819	Ar2	3.06	2.15	1.46	0.17	0.02	0.19	0.11	0.41
3820	Ar2	2.89	1.23	1.42	0.16	0.05	0.24	0.02	1.08
3821	Ar2	2	1.27	1.46	0.14	0.04	0.22	0.04	0.55
3822	Ar2	3.39	1.53	0.53	0.17	0.03	0.24	0.06	0.66
3823	Ar2	1.77	1.4	1.51	0.16	0.02	0.19	0.11	0.75
3824	Ar2	2.04	1.31	1.64	0.22	0.03	0.26	0.15	1
3825	Ar2	2.53	1.31	1.28	0.22	0.02	0.28	0.11	0.57
3826	Er	12.51	0.67	8.96	0.09	0.03	0.13	0.02	0.29
3827	Er	7.05	0.28	4.25	0.24	0.05	0.41	0.15	0.23
3828	Er	4.96	0.15	2.88	0.2	0.03	0.24	0.09	0.24
3829	Ar2	5.3	0.8	2.21	0.11	0.02	0.13	0.06	0.23
3830	Ar2	4.09	0.84	3.91	0.23	0.03	0.26	0.13	0.56
3831	Ar2	5.44	0.8	3.71	0.19	0.03	0.22	0.11	0.61
3832	Er	3.78	0.45	2.57	0.16	0.02	0.17	0.11	0.22
3833	Ar2	3.88	0.37	2.43	0.18	0.03	0.24	0.09	0.3

3834	Ar2	5.53	0.52	1.06	0.15	0.02	0.17	0.06	0.29
3835	Er	5.96	0.26	1.08	0.12	0.03	0.15	0.06	0.27
3836	Ar2	3.33	0.67	2.5	0.11	0.02	0.15	0.06	0.43
3837	Er	2.89	0.41	2.63	0.12	0.02	0.15	0.06	0.21
3838	Er	3.17	0.43	2.61	0.17	0.03	0.22	0.09	0.28
3839	Er	2.95	0.15	2.57	0.19	0.04	0.24	0.06	0.23
3840	Ar2	3.14	1.27	2.6	0.14	0.03	0.17	0.09	0.89
3841	Ar2	3.25	1.4	2.53	0.21	0.03	0.24	0.15	0.35
3842	Ar2	2.9	0.93	2.73	0.19	0.05	0.26	0.11	0.77
3843	Ar2	4.49	2.13	2.72	0.19	0.03	0.24	0.11	1.23
3844	Ar2	3.66	1.36	3.1	0.17	0.02	0.22	0.11	0.32
3845	Ar2	3.77	1.46	3.53	0.16	0.03	0.26	0.04	0.72
3846	Ar2	2.29	0.45	1.07	0.14	0.02	0.17	0.09	0.3
3847	Er	1.79	0.43	1.19	0.16	0.04	0.22	0.04	0.34
3848	Ar2	1.95	0.67	1.14	0.18	0.03	0.22	0.11	0.34
3849	Ar2	1.71	0.34	1.37	0.19	0.04	0.26	0.04	0.34
3850	Ar2	1.7	0.3	1.18	0.15	0.03	0.17	0.09	0.25
3851	Ar2	1.86	0.43	1.08	0.16	0.03	0.19	0.11	0.25
3852	Ar2	1.63	0.37	1.23	0.16	0.02	0.19	0.11	0.32
3853	Er	1.73	0.11	1.28	0.09	0.03	0.13	0.06	0.18
3854	Er	1.87	0.41	1.05	0.12	0.03	0.15	0.06	0.3
3855	Er	2.25	0.19	0.92	0.18	0.02	0.24	0.09	0.2
3856	Er	2.1	0.13	1.03	0.15	0.05	0.24	0.06	0.16
3857	Er	1.77	0.39	1.32	0.13	0.03	0.19	0.09	0.16
3858	Ar2	2.03	0.34	1.17	0.17	0.04	0.24	0.06	0.24
3859	Er	1.6	0.17	1.55	0.1	0.01	0.11	0.06	0.25
3860	Er	1.87	0.24	1.29	0.12	0.02	0.15	0.06	0.15
3861	Er	2.23	0.32	1.18	0.14	0.03	0.17	0.06	0.25
3862	Er	3	0.11	0.79	0.13	0.03	0.17	0.04	0.17
3863	Er	2.21	0.37	1.3	0.13	0.02	0.17	0.06	0.29
3864	Ar2	2.19	0.5	1.34	0.13	0.02	0.15	0.09	0.2
3865	Ar2	2.01	0.32	1.5	0.14	0.02	0.17	0.09	0.3
3866	Ar2	3.27	0.09	0.82	0.15	0.02	0.19	0.11	0.28
3867	Er	2.17	0.28	1.53	0.13	0.03	0.17	0.06	0.23
3868	Ar2	3.44	0.41	0.86	0.14	0.03	0.17	0.09	0.32
3869	Ar2	3.36	0.32	1.01	0.14	0.03	0.17	0.06	0.28
3870	Er	3.6	0.15	0.64	0.15	0.03	0.19	0.09	0.16
3871	Ar2	2.04	0.39	1.65	0.17	0.03	0.22	0.11	0.22
3872	Ar2	2.13	0.41	1.55	0.15	0.02	0.17	0.11	0.24
3873	Ar2	1.85	0.24	1.66	0.15	0.03	0.22	0.09	0.19
3874	Ar2	3.86	0.52	0.38	0.17	0.06	0.32	0.06	0.32
3875	Ar2	1.87	0.71	1.5	0.2	0.02	0.22	0.15	0.52
3876	Ar2	2.15	0.56	1.29	0.19	0.03	0.22	0.13	0.4
3877	Ar2	2.04	0.69	1.25	0.18	0.03	0.22	0.09	0.32
3878	Ar2	1.82	0.26	1.55	0.22	0.05	0.3	0.15	0.25
3879	Ar2	2.05	0.28	1.32	0.24	0.05	0.3	0.15	0.25
3880	Er	1.79	0.13	1.63	0.18	0.03	0.24	0.11	0.28
3881	Ar2	1.75	0.28	1.58	0.17	0.03	0.19	0.11	0.26
3882	Er	2.38	0.24	1.18	0.22	0.02	0.3	0.17	0.21
3883	Er	2.06	0.39	1.29	0.18	0.03	0.24	0.11	0.27
3884	Ar2	1.47	0.84	1.22	0.11	0.02	0.15	0.06	0.55
3885	Ar2	1.82	0.8	0.98	0.14	0.03	0.17	0.04	0.68
3886	Ar2	1.7	0.43	1.19	0.18	0.02	0.22	0.13	0.29
3887	Ar2	2.62	0.47	0.69	0.17	0.03	0.19	0.11	0.24

3888	Ar2	3.19	0.69	0.26	0.13	0.03	0.17	0.06	0.57
3889	Ar2	1.59	0.5	1.06	0.17	0.04	0.28	0.11	0.35
3890	Ar2	1.58	0.69	1.02	0.16	0.03	0.19	0.11	0.4
3891	Ar2	1.75	0.62	0.89	0.16	0.03	0.24	0.09	0.5
3892	Ar2	2.15	0.47	0.59	0.18	0.03	0.24	0.11	0.38
3893	Ar2	2.44	0.6	0.49	0.19	0.05	0.24	0.11	0.38
3894	Ar2	3	0.37	0.14	0.2	0.04	0.24	0.06	0.36
3895	Ar2	2.83	0.8	0.18	0.19	0.03	0.24	0.11	0.35
3896	Ar2	1.52	0.86	0.94	0.15	0.02	0.19	0.09	0.57
3897	Ar2	1.85	0.93	0.83	0.14	0.03	0.19	0.06	0.41
3898	Ar2	1.62	0.75	0.83	0.13	0.02	0.17	0.06	0.41
3899	Ar2	1.57	0.65	0.85	0.16	0.03	0.22	0.06	0.55
3900	Ar2	1.5	0.15	1.23	0.15	0.02	0.17	0.09	0.23
3901	Ar2	1.56	0.65	1.01	0.11	0.03	0.15	0.04	0.33
3902	Ar2	3.04	0.3	0.12	0.15	0.02	0.17	0.11	0.33
3903	Ar2	2.94	0.17	0.32	0.13	0.02	0.15	0.09	0.4
3904	Ar2	2.44	0.62	0.3	0.34	0.04	0.39	0.26	0.57
3905	Ar2	1.41	0.58	1	0.3	0.06	0.37	0.19	0.31
3906	Ar2	1.31	0.34	0.94	0.24	0.06	0.37	0.17	0.33
3907	Ar2	1.18	0.39	1.09	0.28	0.05	0.32	0.15	0.36
3908	Ar2	1.63	0.54	1.12	0.21	0.03	0.26	0.15	0.32
3909	Ar2	1.43	0.28	1.41	0.22	0.03	0.26	0.13	0.28
3910	Er	2.06	0.28	0.92	0.21	0.05	0.28	0.09	0.22
3911	Ar2	1.37	0.19	0.95	0.19	0.05	0.28	0.11	0.18
3912	Ar2	1.42	0.24	0.92	0.22	0.03	0.28	0.15	0.21
3913	Ar2	2.03	0.34	0.81	0.23	0.03	0.26	0.17	0.21
3914	Er	1.98	0.28	0.78	0.24	0.03	0.28	0.17	0.28
3915	Ar2	1.22	0.15	1.19	0.23	0.04	0.3	0.15	0.23
3916	Ar2	1.3	0.22	1.18	0.2	0.02	0.26	0.15	0.2
3917	Ar2	1.6	0.43	1.03	0.22	0.03	0.26	0.15	0.29
3918	Ar2	1.49	0.41	1.09	0.2	0.03	0.24	0.13	0.3
3919	Ar2	1.4	0.3	1.09	0.15	0.02	0.19	0.11	0.21
3920	Er	1.53	0.15	0.98	0.15	0.03	0.19	0.09	0.18
3921	Ar2	1.85	0.09	0.9	0.17	0.03	0.24	0.13	0.2
3922	Ar2	1.49	0.37	1.14	0.18	0.02	0.22	0.09	0.32
3923	Er	2.41	0.15	0.76	0.15	0.02	0.24	0.09	0.14
3924	Er	1.53	0.13	1.17	0.13	0.04	0.26	0.06	0.1
3925	Ar2	1.74	0.19	1.08	0.19	0.03	0.24	0.09	0.17
3926	Ar2	2.46	0.15	0.74	0.14	0.02	0.17	0.09	0.17
3927	Ar2	2.45	0.24	0.7	0.12	0.02	0.15	0.09	0.16
3928	Er	1.95	0.45	0.91	0.14	0.02	0.17	0.11	0.23
3929	Ar2	1.69	0.17	1.08	0.15	0.03	0.17	0.06	0.22
3930	Ar2	2.08	0.3	0.84	0.16	0.02	0.19	0.09	0.23
3931	Ar2	1.53	0.24	1.06	0.12	0.01	0.15	0.11	0.19
3932	Er	1.76	0.13	0.85	0.12	0.02	0.17	0.06	0.12
3933	Er	1.68	0.09	0.83	0.13	0.02	0.17	0.06	0.12
3934	Er	1.71	0.15	0.83	0.12	0.03	0.15	0.06	0.11
3935	Er	1.41	0.09	1.24	0.08	0.02	0.13	0.02	0.11
3936	Ar2	1.5	0.13	1.11	0.12	0.03	0.17	0.04	0.13
3937	Er	1.43	0.11	1.23	0.12	0.02	0.17	0.04	0.13
3938	Ar2	1.54	0.3	1.17	0.13	0.02	0.15	0.06	0.31
3939	Er	2.58	0.15	0.55	0.17	0.04	0.24	0.06	0.19
3940	Ar2	2.68	0.34	0.4	0.17	0.03	0.22	0.09	0.25
3941	Ar2	1.83	0.43	0.79	0.2	0.05	0.26	0.06	0.27

3942	Ar2	1.51	0.39	0.94	0.19	0.03	0.24	0.13	0.35
3943	Ar2	2.77	0.32	0.19	0.19	0.03	0.22	0.09	0.3
3944	Ar2	1.36	0.43	1.08	0.16	0.02	0.19	0.11	0.31
3945	Ar2	1.51	0.15	1.07	0.17	0.03	0.22	0.09	0.28
3946	Er	3.08	0.28	0.1	0.13	0.02	0.15	0.06	0.3
3947	Ar2	1.44	0.28	1.05	0.14	0.02	0.17	0.06	0.21
3948	Er	1.56	0.11	0.94	0.11	0.03	0.15	0.06	0.16
3949	Er	1.69	0.39	0.85	0.1	0.02	0.13	0.06	0.27
3950	Er	2.25	0.3	0.35	0.12	0.02	0.15	0.09	0.21
3951	Er	1.8	0.06	0.68	0.12	0.03	0.17	0.09	0.17
3952	Ar2	1.35	0.41	0.93	0.13	0.02	0.17	0.06	0.26
3953	Ar2	1.23	0.37	0.97	0.12	0.02	0.15	0.09	0.28
3954	Ar2	1.34	0.37	1.06	0.15	0.02	0.17	0.09	0.27
3955	Er	2.32	0.28	0.41	0.17	0.02	0.19	0.09	0.25
3956	Er	1.09	0.15	0.61	0.16	0.03	0.22	0.11	0.15
3957	Ar2	1.07	0.26	0.69	0.15	0.02	0.17	0.13	0.25
3958	Ar2	0.9	0.34	0.72	0.15	0.02	0.17	0.11	0.25
3959	Er	1.33	0.11	0.44	0.14	0.03	0.19	0.09	0.13
3960	Er	1.3	0.15	0.43	0.15	0.03	0.22	0.09	0.13
3961	Haer	1.3	0.19	0.45	0.12	0.03	0.15	0.09	0.12
3962	Er	1.47	0.13	0.4	0.14	0.03	0.22	0.09	0.12
3963	Er	1.41	0.13	0.42	0.13	0.03	0.17	0.09	0.11
3964	Er	1.96	0.13	0.05	0.12	0.04	0.19	0.04	0.11
3965	Ar2	1.51	0.15	0.24	0.14	0.02	0.15	0.09	0.23
3966	Haer	1.62	0.06	0.2	0.13	0.03	0.17	0.09	0.12
3967	Ar2	2.15	0.17	0.12	0.17	0.02	0.19	0.13	0.18
3968	Ar2	2.05	0.17	0.07	0.19	0.02	0.22	0.11	0.22
3969	Haer	1.55	0.17	0.33	0.13	0.05	0.26	0.04	0.12
3970	Er	1.02	0.32	0.54	0.16	0.02	0.19	0.09	0.28
3971	Ar2	1.26	0.15	0.42	0.15	0.03	0.19	0.11	0.2
3972	Ar2	1.47	0.3	0.35	0.14	0.02	0.17	0.11	0.24
3973	Er	1.22	0.39	0.55	0.14	0.03	0.19	0.09	0.23
3974	Er	1.22	0.15	0.62	0.09	0.02	0.11	0.06	0.13
3975	Ar2	1.59	0.09	0.52	0.1	0.02	0.17	0.04	0.09
3976	Er	1.44	0.11	0.38	0.11	0.02	0.15	0.06	0.1
3977	Er	0.86	0.15	0.8	0.12	0.02	0.17	0.04	0.12
3978	Ar2	1.61	0.06	0.35	0.11	0.03	0.17	0.04	0.1
3979	Er	1.1	0.13	0.82	0.15	0.02	0.19	0.11	0.12
3980	Er	1.01	0.11	0.95	0.12	0.02	0.15	0.09	0.11
3981	Ar2	1.11	0.24	0.77	0.13	0.02	0.15	0.11	0.21
3982	Er	1.53	0.09	0.49	0.12	0.04	0.19	0.06	0.12
3983	Ar2	1.15	0.15	0.73	0.15	0.02	0.19	0.06	0.14
3984	Er	1.48	0.17	0.57	0.13	0.03	0.17	0.06	0.14
3985	Er	0.99	0.17	0.72	0.14	0.03	0.19	0.06	0.16
3986	Ar2	1.78	0.3	0.3	0.15	0.03	0.19	0.04	0.22
3987	Er	0.87	0.06	0.6	0.17	0.05	0.28	0.11	0.14
3988	Er	1.18	0.26	0.36	0.25	0.03	0.32	0.15	0.17
3989	Ar2	1.28	0.32	0.46	0.2	0.03	0.24	0.15	0.27
3990	Ar2	0.95	0.19	0.72	0.19	0.04	0.28	0.13	0.22
3991	Ar2	1.08	0.22	0.52	0.22	0.02	0.28	0.09	0.2
3992	Er	0.86	0.15	0.62	0.17	0.04	0.24	0.11	0.18
3993	Er	1.47	0.34	0.32	0.16	0.04	0.24	0.09	0.18
3994	Ar2	1.62	0.39	0.32	0.2	0.02	0.22	0.15	0.25
3995	Ar2	1.16	0.24	0.52	0.19	0.04	0.26	0.11	0.19

3996	Er	1.05	0.11	0.37	0.13	0.04	0.26	0.06	0.11
3997	Er	1.06	0.17	0.31	0.15	0.04	0.22	0.06	0.13
3998	Er	1.34	0.24	0.23	0.15	0.02	0.17	0.11	0.22
3999	Ar2	0.89	0.24	0.47	0.13	0.02	0.15	0.11	0.22
4000	Er	0.99	0.17	0.54	0.16	0.02	0.19	0.11	0.17
4001	Ar2	0.87	0.15	0.59	0.15	0.03	0.19	0.09	0.15
4002	Ar2	1.27	0.15	0.37	0.15	0.01	0.17	0.13	0.15
4003	Er	0.75	0.15	0.63	0.16	0.02	0.19	0.09	0.16
4004	Haer	0.77	0.19	0.59	0.16	0.03	0.22	0.09	0.13
4005	Er	1.32	0.15	0.24	0.16	0.04	0.24	0.06	0.14
4006	Ar2	1.15	0.13	0.39	0.15	0.03	0.22	0.11	0.18
4007	Er	1.15	0.19	0.41	0.17	0.02	0.19	0.13	0.18
4008	Er	1.53	0.17	0.24	0.16	0.02	0.22	0.13	0.16
4009	Er	1.56	0.15	0.17	0.15	0.04	0.22	0.09	0.18
4010	Ar2	1.77	0.34	0.07	0.2	0.03	0.24	0.11	0.2
4011	Haer	1.14	0.11	0.4	0.15	0.06	0.24	0.06	0.13
4012	Er	1.11	0.17	0.37	0.19	0.03	0.24	0.13	0.19
4013	Ar2	0.77	0.19	0.53	0.18	0.04	0.26	0.09	0.16
4014	Haer	0.99	0.22	0.36	0.17	0.05	0.26	0.06	0.17
4015	Er	0.91	0.19	0.49	0.16	0.03	0.22	0.11	0.19
4016	Er	1.32	0.17	0.25	0.16	0.03	0.22	0.11	0.16
4017	Ar2	1.21	0.17	0.32	0.17	0.02	0.24	0.13	0.17
4018	Ar2	1.6	0.39	0.22	0.1	0.02	0.13	0.06	0.25
4019	Ar2	1.03	0.13	0.51	0.12	0.02	0.15	0.04	0.17
4020	Er	0.95	0.11	0.59	0.11	0.02	0.13	0.06	0.16
4021	Ar2	1.19	0.11	0.44	0.12	0.02	0.15	0.06	0.19
4022	Er	1.15	0.41	0.32	0.1	0.01	0.11	0.06	0.22
4023	Ar2	1.01	0.37	0.46	0.12	0.01	0.15	0.09	0.23
4024	Ar2	0.92	0.43	0.5	0.11	0.02	0.19	0.04	0.24
4025	Ar2	0.11	0.13	1.13	0.13	0.02	0.17	0.06	0.27
4026	Er	0.73	0.43	0.67	0.12	0.03	0.15	0.06	0.22
4027	Ar2	0.91	0.43	0.59	0.12	0.02	0.15	0.09	0.37
4028	Er	0.82	0.34	0.74	0.12	0.03	0.15	0.04	0.27
4029	Ar2	1.05	0.39	0.59	0.11	0.03	0.15	0.06	0.2
4030	Ar2	2.03	0.15	0.1	0.15	0.02	0.17	0.11	0.22
4031	Ar2	1.76	0.22	0.2	0.13	0.02	0.15	0.09	0.24
4032	Ar2	0.93	0.45	0.66	0.16	0.03	0.19	0.11	0.3
4033	Ar2	0.87	0.43	0.66	0.16	0.02	0.22	0.11	0.26
4034	Ar2	0.9	0.34	0.64	0.15	0.02	0.19	0.11	0.32
4035	Ar2	1.13	0.52	0.47	0.15	0.02	0.17	0.13	0.4
4036	Ar2	1.5	0.15	0.41	0.16	0.03	0.22	0.09	0.24
4037	Er	1.73	0.17	0.18	0.17	0.03	0.26	0.11	0.18
4038	Ar2	1.68	0.17	0.21	0.18	0.02	0.22	0.13	0.31
4039	Ar2	1.68	0.15	0.09	0.17	0.03	0.19	0.09	0.24
4040	Er	1.71	0.26	0.06	0.17	0.03	0.22	0.09	0.19
4041	Haer	1.61	0.82	0.07	0.17	0.04	0.28	0.06	0.16
4042	Ar2	1.33	0.34	0.27	0.16	0.03	0.19	0.11	0.24
4043	Ar2	1.56	0.62	0.11	0.15	0.03	0.19	0.11	0.25
4044	Ar2	1.04	0.17	0.5	0.18	0.03	0.22	0.06	0.18
4045	Ar2	0.96	0.19	0.59	0.18	0.03	0.22	0.09	0.17
4046	Ar2	1.32	0.37	0.35	0.17	0.02	0.19	0.09	0.26
4047	Er	0.81	0.3	0.62	0.15	0.04	0.19	0.06	0.24
4048	Ar2	1.22	0.43	0.29	0.15	0.02	0.17	0.11	0.32
4049	Er	1.03	0.11	0.45	0.15	0.03	0.24	0.06	0.14

4050	Er	0.7	0.45	0.64	0.15	0.03	0.17	0.09	0.37
4051	Er	0.72	0.58	0.62	0.11	0.03	0.17	0.09	0.22
4052	Ar2	0.93	0.78	0.67	0.15	0.03	0.19	0.09	0.34
4053	Ar2	1.1	0.62	0.67	0.15	0.03	0.17	0.11	0.27
4054	Ar2	1.25	0.58	0.53	0.15	0.02	0.19	0.11	0.45
4055	Ar2	0.97	0.41	0.77	0.14	0.02	0.19	0.11	0.3
4272	Ar2	0.85	2.15	0.63	0.28	0.04	0.34	0.22	1.15
4057	Ar2	0.86	0.26	0.76	0.13	0.03	0.17	0.09	0.24
4058	Ar2	1.52	0.32	0.3	0.14	0.03	0.19	0.06	0.36
4059	Ar2	1.3	0.69	0.42	0.12	0.02	0.15	0.06	0.31
4060	Ar2	1.1	0.41	0.5	0.14	0.02	0.15	0.09	0.63
4273	Ar2	1.34	2.05	0.41	0.26	0.06	0.37	0.13	1.38
4062	Ar2	0.94	0.82	0.65	0.16	0.02	0.19	0.11	0.39
4063	Ar2	1.15	0.26	0.47	0.18	0.04	0.22	0.09	0.19
4064	Ar2	1.19	0.17	0.46	0.18	0.03	0.22	0.11	0.2
4065	Ar2	0.79	0.67	0.62	0.15	0.02	0.17	0.11	0.38
4066	Ar2	0.98	0.47	0.64	0.16	0.02	0.22	0.11	0.24
4067	Ar2	1.02	0.47	0.57	0.16	0.03	0.19	0.09	0.38
4068	Ar2	1.11	0.9	0.46	0.15	0.03	0.19	0.09	0.25
4069	Ar2	2.01	0.88	0.47	0.13	0.01	0.17	0.09	0.57
4070	Ar2	1.2	0.93	0.87	0.12	0.02	0.17	0.06	0.54
4071	Ar2	1.44	0.84	0.69	0.12	0.02	0.17	0.06	0.52
4072	Ar2	1.18	0.82	0.81	0.13	0.02	0.17	0.09	0.47
4073	Ar2	1.22	0.56	0.74	0.15	0.03	0.19	0.09	0.33
4074	Ar2	1.08	0.82	0.68	0.14	0.04	0.22	0.06	0.42
4075	Ar2	2.16	1.23	0.16	0.15	0.02	0.19	0.06	0.21
4076	Er	2.08	0.78	0.12	0.11	0.04	0.19	0.04	0.32
4077	Ar2	1.33	0.15	0.71	0.14	0.03	0.17	0.09	0.24
4078	Ar2	1.38	0.47	0.59	0.14	0.03	0.19	0.06	0.44
4079	Ar2	1.08	0.65	0.78	0.13	0.03	0.19	0.06	0.46
4080	Ar2	1.8	0.97	0.3	0.13	0.03	0.15	0.06	0.56
4081	Ar2	2.03	0.47	0.19	0.13	0.03	0.17	0.09	0.38
4082	Ar2	1.11	0.93	0.63	0.12	0.02	0.15	0.06	0.48
4083	Ar2	1.19	1.14	0.63	0.12	0.02	0.15	0.09	0.56
4084	Ar2	0.9	0.9	0.72	0.15	0.03	0.24	0.06	0.39
4085	Ar2	1.38	0.97	0.41	0.13	0.03	0.17	0.04	0.72
4086	Ar2	1.94	0.17	0.13	0.18	0.02	0.22	0.15	0.33
4087	Ar2	1.22	0.5	0.54	0.16	0.02	0.19	0.13	0.42
4088	Ar2	1.23	0.54	0.51	0.16	0.02	0.19	0.11	0.49
4089	Ar2	1.53	0.97	0.35	0.13	0.02	0.15	0.11	0.48
4275	Ar2	1.45	2.07	0.32	0.29	0.06	0.41	0.13	1.15
4091	Ar2	1.28	0.09	0.44	0.14	0.05	0.24	0.06	0.13
4092	Ar2	1.94	0.28	0.12	0.14	0.03	0.17	0.11	0.33
4093	Er	1.17	0.15	0.76	0.16	0.03	0.22	0.11	0.15
4094	Er	1.07	0.34	0.77	0.16	0.02	0.19	0.11	0.23
4095	Er	2.29	0.15	0.04	0.18	0.03	0.26	0.13	0.17
4096	Ar2	1.47	0.13	0.55	0.16	0.05	0.24	0.09	0.16
4097	Er	1.25	0.19	0.69	0.2	0.04	0.3	0.13	0.2
4098	Ar2	0.74	0.19	0.92	0.18	0.04	0.26	0.11	0.23
4099	Er	1.3	0.41	0.56	0.19	0.02	0.24	0.11	0.22
4100	Er	1.25	0.19	0.61	0.19	0.02	0.24	0.11	0.21
4101	Ar2	1.8	0.32	0.44	0.17	0.03	0.22	0.11	0.27
4102	Ar2	1.65	0.5	0.42	0.18	0.02	0.22	0.15	0.31
4103	Ar2	1.49	0.34	0.56	0.18	0.04	0.24	0.09	0.27

4104	Er	1.49	0.28	0.74	0.16	0.03	0.19	0.09	0.2
4105	Ar2	1.82	0.19	0.49	0.17	0.04	0.24	0.06	0.2
4106	Ar2	1.13	0.37	0.84	0.18	0.02	0.22	0.15	0.33
4107	Er	1.16	0.15	0.86	0.17	0.03	0.24	0.11	0.17
4108	Er	1.15	0.34	0.89	0.18	0.03	0.24	0.11	0.23
4109	Ar2	1.19	0.15	0.98	0.17	0.03	0.22	0.09	0.16
4110	Ar2	1.54	0.17	0.75	0.18	0.03	0.22	0.13	0.17
4111	Ar2	1.85	0.19	0.57	0.16	0.05	0.26	0.06	0.19
4112	Er	1.47	0.17	0.97	0.18	0.02	0.22	0.13	0.16
4113	Er	1.44	0.11	0.92	0.16	0.04	0.22	0.06	0.16
4114	Er	1.65	0.11	0.75	0.14	0.04	0.22	0.09	0.17
4115	Ar2	2.36	0.15	0.21	0.21	0.04	0.26	0.13	0.25
4116	Ar2	2.8	0.52	0.06	0.19	0.02	0.22	0.17	0.29
4117	Ar2	2.73	0.45	0.09	0.19	0.03	0.24	0.15	0.24
4118	Ar2	1.16	0.45	1.01	0.16	0.03	0.24	0.11	0.26
4119	Er	1.78	0.19	0.68	0.18	0.03	0.24	0.13	0.19
4120	Er	1.18	0.45	1.02	0.21	0.03	0.26	0.15	0.26
4121	Ar2	1.58	0.26	0.65	0.22	0.05	0.28	0.06	0.22
4122	Ar2	1.06	0.15	1.01	0.18	0.04	0.24	0.13	0.19
4123	Er	1.6	0.47	0.66	0.21	0.03	0.24	0.11	0.28
4124	Ar2	1.03	0.43	0.76	0.16	0.02	0.19	0.09	0.35
4125	Ar2	0.89	0.45	0.83	0.14	0.02	0.19	0.09	0.31
4126	Ar2	0.88	0.34	0.81	0.15	0.02	0.19	0.11	0.25
4127	Ar2	0.97	0.62	0.76	0.16	0.01	0.17	0.13	0.27
4128	Ar2	1.4	0.34	0.55	0.17	0.03	0.22	0.11	0.27
4129	Ar2	1.11	0.43	0.75	0.15	0.03	0.19	0.11	0.34
4130	Ar2	1.17	0.19	0.69	0.14	0.04	0.22	0.06	0.2
4131	Haer	1.1	0.13	0.82	0.15	0.04	0.26	0.06	0.16
4132	Er	1.21	0.11	0.79	0.13	0.03	0.17	0.06	0.19
4133	Ar2	1.33	0.34	0.67	0.13	0.02	0.15	0.09	0.23
4134	Er	1.07	0.39	0.83	0.11	0.03	0.17	0.06	0.15
4135	Er	1.35	0.5	0.76	0.15	0.02	0.19	0.06	0.25
4136	Er	1.72	0.45	0.52	0.15	0.02	0.17	0.09	0.3
4137	Ar2	1.48	0.47	0.55	0.13	0.01	0.15	0.09	0.33
4138	Er	1.29	0.41	0.76	0.12	0.02	0.15	0.06	0.23
4139	Ar2	1.2	0.26	0.74	0.12	0.02	0.15	0.06	0.2
4140	Ar2	1.88	0.43	0.55	0.14	0.02	0.17	0.09	0.3
4141	Ar2	1.09	0.13	1.06	0.12	0.03	0.22	0.06	0.13
4142	Ar2	1.4	0.3	0.85	0.15	0.02	0.17	0.09	0.32
4143	Ar2	1.84	0.26	0.28	0.12	0.01	0.13	0.06	0.24
4144	Er	2.54	0.37	0.09	0.13	0.02	0.15	0.06	0.32
4145	Ar2	2.57	0.45	0.07	0.08	0.02	0.11	0.04	0.32
4146	Er	2.33	0.97	0.36	0.17	0.03	0.22	0.11	0.29
4147	Ar2	1.58	0.06	0.75	0.16	0.03	0.24	0.13	0.43
4148	Er	1.27	0.6	0.94	0.17	0.03	0.22	0.11	0.2
4149	Ar2	1.34	0.6	0.91	0.16	0.04	0.22	0.09	0.37
4150	Ar2	1.12	0.8	1.01	0.17	0.03	0.22	0.09	0.28
4151	Ar2	1.86	0.37	0.62	0.19	0.02	0.24	0.11	0.31
4152	Ar2	2.16	0.43	0.36	0.18	0.03	0.24	0.11	0.25
4276	Ar2	1.13	2.02	0.47	0.27	0.07	0.39	0.13	1.1
4154	Ar2	1.39	0.84	0.85	0.17	0.03	0.22	0.13	0.34
4155	Ar2	1.14	0.26	0.94	0.23	0.04	0.28	0.15	0.22
4156	Er	1.75	0.6	0.48	0.24	0.04	0.28	0.06	0.31
4157	Ar2	1.66	0.6	0.55	0.18	0.02	0.24	0.11	0.44

4158	Ar2	1	0.24	0.79	0.23	0.04	0.28	0.15	0.2
4159	Ar2	2.25	0.19	0.1	0.2	0.04	0.32	0.13	0.2
4160	Ar2	1.55	0.24	0.58	0.22	0.03	0.26	0.15	0.26
4161	Ar2	1.48	0.13	0.55	0.22	0.04	0.3	0.13	0.23
4162	Ar2	2.56	0.28	0.12	0.28	0.03	0.32	0.19	0.4
4163	Ar2	1.44	0.5	0.53	0.23	0.03	0.3	0.17	0.33
4277	Ar2	1.12	1.81	0.54	0.25	0.05	0.37	0.15	1
4165	Ar2	0.97	0.69	0.79	0.18	0.03	0.24	0.15	0.72
4278	Ar2	1.35	2.24	0.26	0.26	0.06	0.37	0.09	1.47
4279	Ar2	0.95	2.2	0.59	0.23	0.06	0.34	0.13	1.15
4168	Ar2	1.45	1.77	0.38	0.21	0.05	0.3	0.13	1.37
4280	Ar2	1.01	2.26	0.62	0.27	0.03	0.37	0.15	1.36
4281	Ar2	1.2	2	0.53	0.24	0.07	0.37	0.11	1.57
4283	Ar2	1.08	1.96	0.46	0.29	0.05	0.37	0.13	1.6
4172	Ar2	1.01	2.26	0.7	0.19	0.05	0.34	0.11	1.42
4284	Ar2	0.76	1.77	0.7	0.28	0.04	0.34	0.19	1.51
4286	Ar2	0.94	1.92	0.54	0.32	0.07	0.41	0.09	1.86
4287	Ar2	0.93	1.98	0.61	0.27	0.05	0.37	0.17	1.76
4176	Ar2	1.56	1.87	0.76	0.19	0.02	0.24	0.13	0.9
4177	Ar2	1.39	1.96	0.78	0.16	0.03	0.22	0.09	1.63
4178	Ar2	1.44	2.09	0.75	0.19	0.04	0.26	0.11	1.2
4179	Ar2	1.14	2.28	0.71	0.21	0.04	0.3	0.09	1.72
4180	Ar2	1.13	2.22	0.89	0.19	0.04	0.26	0.11	1.52
4288	Ar2	0.99	1.68	0.44	0.33	0.05	0.39	0.22	1.31
4289	Ar2	0.75	0.28	0.59	0.3	0.08	0.47	0.17	1.04
4290	Ar2	0.84	1.72	0.56	0.3	0.07	0.41	0.15	1.07
4291	Ar2	0.89	1.79	0.56	0.29	0.08	0.37	0.11	1.05
4292	Ar2	0.91	1.7	0.5	0.3	0.07	0.41	0.13	1.29
4293	Ar2	0.85	1.96	0.53	0.35	0.07	0.41	0.15	0.74
4294	Ar2	0.88	1.66	0.4	0.29	0.07	0.41	0.15	1.26
4295	Ar2	1.14	2	0.49	0.33	0.07	0.41	0.13	1.33
4296	Ar2	0.91	1.83	0.63	0.3	0.08	0.41	0.15	1.1
4190	Ar2	1.24	1.16	0.74	0.33	0.09	0.45	0.13	1.02
4191	Ar2	1.32	1.31	0.59	0.27	0.08	0.45	0.15	0.69
4192	Ar2	1.32	0.58	0.56	0.27	0.09	0.39	0.13	0.7
4193	Ar2	1.17	1.23	0.77	0.3	0.03	0.34	0.24	0.85
4297	Ar2	0.87	2.13	0.5	0.32	0.1	0.45	0.09	1.26
4195	Ar2	1.42	1.23	0.66	0.32	0.03	0.39	0.24	0.92
4196	Ar2	1.32	1.27	1.02	0.43	0.04	0.47	0.3	0.81
4197	Ar2	1.39	0.84	1.12	0.28	0.14	0.5	0.13	0.58
4198	Ar2	2.03	1.49	1.1	0.27	0.11	0.43	0.11	1
4298	Ar2	0.97	1.77	0.46	0.34	0.09	0.43	0.13	1.05
4200	Ar2	1.38	1.49	0.91	0.29	0.08	0.39	0.11	1.07
4299	Ar2	1.22	1.83	0.3	0.35	0.06	0.47	0.17	0.98
4300	Ar2	0.92	0.71	0.6	0.27	0.08	0.37	0.13	0.93
4301	Ar2	1.15	1.98	0.45	0.25	0.06	0.37	0.11	0.95
4302	Ar2	0.85	1.87	0.59	0.25	0.1	0.37	0.04	1.18
4205	Ar2	1.67	1.77	0.41	0.25	0.06	0.37	0.15	1.16
4306	Ar2	0.68	1.44	0.56	0.26	0.03	0.32	0.19	1.5
4307	Ar2	0.76	1.79	0.46	0.26	0.07	0.37	0.11	1.15
4208	Ar2	1.38	1.38	0.67	0.34	0.05	0.41	0.24	1.07
4209	Ar2	0.97	1.34	0.92	0.38	0.05	0.45	0.28	1.24
4308	Ar2	0.81	1.62	0.42	0.26	0.04	0.32	0.17	1.11
4211	Ar2	1.23	1.62	0.77	0.36	0.07	0.43	0.15	1.21

4309	Ar2	0.89	1.77	0.4	0.24	0.05	0.37	0.17	1.06
4213	Ar2	1.28	1.96	1.06	0.34	0.07	0.5	0.17	1.03
4317	Ar2	1.09	2	0.81	0.19	0.06	0.28	0.09	0.87
4319	Ar2	0.97	1.46	0.79	0.24	0.06	0.32	0.13	0.69
4216	Ar2	1.6	1.85	0.78	0.29	0.03	0.34	0.17	1.57
4320	Ar2	0.84	1.31	0.69	0.27	0.07	0.34	0.11	0.87
4218	Ar2	1.46	1.72	0.77	0.3	0.05	0.37	0.13	1.56
4325	Ar2	0.84	0.22	0.83	0.29	0.06	0.41	0.19	0.47
4327	Ar2	0.97	1.55	0.59	0.22	0.04	0.3	0.15	0.72
4332	Ar2	1.03	1.7	0.59	0.23	0.04	0.3	0.15	0.79
4222	Ar2	0.97	2	0.53	0.24	0.04	0.3	0.13	1.21
4223	Ar2	1.18	1.66	0.38	0.22	0.04	0.32	0.13	1.4
4334	Ar2	0.8	1.51	0.62	0.28	0.04	0.32	0.15	0.91
4338	Ar2	1.1	0.9	0.92	0.18	0.02	0.24	0.15	0.82
4343	Ar2	0.96	1.53	0.85	0.22	0.03	0.26	0.15	0.63
4227	Ar2	1.07	1.79	0.39	0.22	0.04	0.26	0.11	1.3
4228	Ar2	0.81	1.62	0.62	0.23	0.03	0.3	0.13	1.24
4349	Ar2	1.15	0.71	0.761186	0.2	0.04	0.28	0.13	0.52
4361	Ar2	1.23	1.31	0.92	0.24	0.04	0.3	0.15	0.89
4231	Ar2	1.21	1.79	0.31	0.22	0.04	0.3	0.13	1.55
4366	Ar2	1.02	0.86	0.94	0.21	0.05	0.32	0.11	0.68
4233	Ar2	1.25	1.7	0.36	0.21	0.04	0.28	0.11	0.82
4378	Ar2	0.85	1.72	0.68	0.16	0.01	0.17	0.11	0.65
4389	Ar2	0.96	0.24	0.77	0.18	0.04	0.24	0.11	0.35
4236	Ar2	0.83	1.4	0.54	0.23	0.06	0.37	0.15	0.57
4237	Ar2	0.92	1.31	0.41	0.23	0.06	0.3	0.13	0.9
4238	Ar2	1.15	1.64	0.23	0.22	0.04	0.3	0.15	0.79
4422	Ar2	1.31	1.44	0.59	0.24	0.03	0.3	0.13	0.87
4424	Ar2	1.44	0.78	0.67	0.23	0.02	0.26	0.15	0.93
4241	Ar2	1.44	0.73	0.24	0.24	0.06	0.32	0.09	0.87
4242	Ar2	0.97	1.46	0.41	0.22	0.04	0.3	0.11	1.1
4426	Ar2	0.89	1.57	0.85	0.2	0.03	0.24	0.11	0.78
4244	Ar2	1.49	1.62	0.22	0.2	0.03	0.26	0.15	1.2
4245	Ar2	1.06	1.4	0.49	0.24	0.02	0.28	0.15	1.04
4246	Ar2	1.06	1.49	0.41	0.21	0.05	0.32	0.13	1.17
4431	Ar2	1.51	1.57	0.38	0.19	0.05	0.28	0.06	0.85
4248	Ar2	1.09	1.38	0.48	0.2	0.05	0.28	0.09	1.17
4249	Ar2	1.25	2	0.41	0.22	0.03	0.28	0.15	0.92
4250	Ar2	0.99	1.38	0.6	0.22	0.04	0.28	0.15	1.16
4251	Ar2	1.54	1.42	0.36	0.24	0.03	0.32	0.15	0.8
4252	Ar2	1.18	1.79	0.52	0.27	0.04	0.32	0.13	0.62
4436	Ar2	0.94	1.7	0.57	0.18	0.02	0.22	0.15	1.21
4254	Ar2	1.17	1.44	0.44	0.17	0.04	0.28	0.11	1.09
4437	Ar2	1	1.96	0.63	0.18	0.03	0.22	0.13	0.98
4438	Ar2	1.02	0.86	0.64	0.18	0.03	0.22	0.13	0.72
4257	Ar2	0.89	1.38	0.54	0.18	0.04	0.26	0.13	1.11
4439	Ar2	1.05	1.66	0.51	0.18	0.03	0.24	0.13	0.77
4450	Ar2	1.19	0.86	1.04	0.17	0.05	0.24	0.09	0.55
4260	Ar2	0.73	1.66	0.54	0.3	0.06	0.39	0.09	1.43
4463	Ar2	1.41	0.82	1.26	0.24	0.05	0.37	0.13	0.68
4504	Ar2	2.56	0.82	1.04	0.3	0.04	0.41	0.22	0.55
4513	Ar2	1.77	0.26	1.26	0.22	0.05	0.3	0.09	0.38
4516	Ar2	1.32	1.7	1.03	0.21	0.04	0.26	0.13	0.58
4518	Ar2	2.55	1.81	0.5	0.21	0.07	0.32	0.11	0.57

4521	Ar2	2.97	1.94	0.62	0.15	0.02	0.19	0.06	0.98
4522	Ar2	1.73	1.92	1.15	0.15	0.02	0.17	0.09	1.13
4528	Ar2	1.69	1.57	1.14	0.19	0.03	0.22	0.09	0.8
4543	Ar2	0.5	1.34	0.36	0.25	0.03	0.28	0.15	0.79
4545	Ar2	0.49	1.03	0.43	0.24	0.05	0.28	0.15	0.82
4554	Ar2	0.72	1.46	0.36	0.21	0.03	0.26	0.11	0.74
4580	Ar2	0.52	1.64	0.37	0.21	0.02	0.26	0.17	0.59
4582	Ar2	0.55	1.64	0.26	0.22	0.03	0.26	0.15	0.67
4590	Ar2	0.76	0.9	0.04	0.17	0.03	0.22	0.09	0.72
4595	Ar2	0.41	1.27	0.18	0.23	0.03	0.26	0.09	0.7
4597	Ar2	0.53	1.55	0.13	0.24	0.04	0.28	0.13	0.54
4618	Ar2	0.49	0.88	0.33	0.14	0.02	0.19	0.13	0.75
4622	Ar2	0.78	0.22	0.32	0.22	0.03	0.28	0.13	0.56
4623	Ar2	0.82	1.42	0.21	0.17	0.03	0.22	0.11	0.52
4631	Ar2	0.52	1.55	0.11	0.28	0.06	0.34	0.09	0.93
4636	Ar2	0.53	1.66	0.24	0.24	0.04	0.28	0.13	1.15
4282	Ar2	1.2	2	0.55	0.26	0.05	0.32	0.17	1.51
4639	Ar2	0.68	1.98	0.23	0.3	0.05	0.37	0.17	1.04
4640	Ar2	0.56	2.15	0.21	0.28	0.04	0.32	0.17	1.31
4285	Ar2	1.15	1.49	0.53	0.27	0.03	0.32	0.22	1.72
4641	Ar2	0.51	2.11	0.29	0.25	0.04	0.32	0.19	1.47
4648	Ar2	0.51	0.71	0.45	0.28	0.06	0.34	0.17	0.6
4651	Ar2	0.57	1.46	0.32	0.28	0.04	0.34	0.17	1.2
4656	Ar2	0.71	1.77	0.34	0.25	0.05	0.3	0.17	0.83
4661	Ar2	0.6	2.02	0.27	0.24	0.07	0.37	0.13	1
4662	Ar2	0.74	1.79	0.21	0.28	0.04	0.37	0.17	1.3
4668	Ar2	0.83	1.92	0.36	0.26	0.06	0.34	0.09	1.11
4673	Ar2	1.05	1.94	0.22	0.22	0.03	0.28	0.17	1.12
4674	Ar2	0.7	1.96	0.37	0.23	0.07	0.34	0.09	0.98
4676	Ar2	0.52	1.77	0.426099	0.27	0.04	0.3	0.19	1.16
4678	Ar2	0.59	1.92	0.33	0.22	0.03	0.26	0.17	1.71
4685	Ar2	0.31	1.81	0.19	0.18	0.03	0.24	0.13	1.1
4688	Ar2	0.65	1.57	0.09	0.23	0.06	0.28	0.09	0.72
4695	Ar2	0.31	0.97	0.19	0.22	0.06	0.37	0.09	0.77
4699	Ar2	0.35	1.66	0.18	0.21	0.02	0.24	0.17	0.97
4702	Ar2	0.34	1.98	0.25	0.16	0.02	0.19	0.13	0.67
4707	Ar2	0.86	1.89	0.58	0.16	0.02	0.19	0.11	0.89
4303	Ar2	0.82	1.59	0.4	0.25	0.05	0.34	0.13	1.19
4304	Ar2	1.24	1.98	0.21	0.26	0.04	0.3	0.15	1.34
4305	Ar2	0.84	1.74	0.49	0.29	0.05	0.37	0.17	1.08
4708	Ar2	0.89	1.89	0.42	0.17	0.02	0.19	0.13	1.15
4713	Ar2	0.84	1.57	0.41	0.2	0.04	0.28	0.11	1.01
4714	Ar2	0.7	0.75	0.52	0.22	0.04	0.28	0.13	0.99
4720	Ar2	0.78	0.65	0.21	0.21	0.04	0.28	0.11	0.96
4310	Ar2	0.79	1.49	0.38	0.3	0.05	0.37	0.19	0.94
4311	Ar2	0.75	1.77	0.51	0.31	0.06	0.41	0.17	0.79
4312	Ar2	1.09	1.34	0.64	0.25	0.05	0.34	0.13	0.79
4313	Ar2	1.38	1.21	0.43	0.26	0.07	0.37	0.09	0.72
4314	Ar2	1.53	1.31	0.43	0.23	0.05	0.3	0.11	1.14
4315	Ar2	1.19	1.34	0.59	0.24	0.05	0.3	0.11	1.18
4316	Ar2	2.22	1.31	0.23	0.25	0.04	0.3	0.13	1.08
4721	Ar2	0.68	0.86	0.42	0.24	0.04	0.32	0.13	0.67
4318	Ar2	1.11	1.25	0.82	0.22	0.04	0.3	0.11	1.22
4725	Ar2	0.99	1.59	0.13	0.22	0.01	0.26	0.19	1.07

4728	Ar2	0.92	1.81	0.24	0.18	0.03	0.22	0.11	0.85
4321	Ar2	1.17	1.18	0.63	0.29	0.05	0.37	0.15	0.96
4322	Ar2	1.04	1.36	0.58	0.25	0.06	0.37	0.15	0.44
4323	Ar2	1.01	1.01	0.55	0.26	0.06	0.34	0.15	0.81
4324	Ar2	1.34	1.12	0.36	0.26	0.07	0.39	0.13	0.74
4729	Ar2	0.75	0.73	0.37	0.19	0.03	0.24	0.13	0.78
4326	Ar2	1.26	0.71	0.52	0.31	0.07	0.47	0.13	0.46
4733	Ar2	0.53	1.74	0.32	0.19	0.04	0.28	0.09	1.01
4328	Ar2	0.81	1.01	0.67	0.21	0.06	0.32	0.11	0.8
4329	Ar2	0.95	1.36	0.63	0.2	0.05	0.28	0.11	0.95
4330	Ar2	1.14	0.9	0.54	0.21	0.05	0.3	0.11	0.92
4331	Ar2	1.09	1.31	0.58	0.27	0.04	0.32	0.11	1.05
4738	Ar2	0.85	1.7	0.24386	0.18	0.04	0.24	0.11	0.84
4333	Ar2	0.91	1.46	0.7	0.28	0.03	0.32	0.19	0.46
4740	Ar2	0.47	1.29	0.36	0.19	0.04	0.26	0.11	0.55
4335	Ar2	1.2	1.18	0.49	0.28	0.05	0.34	0.15	0.86
4336	Ar2	0.97	0.22	0.7	0.26	0.05	0.39	0.15	0.36
4337	Ar2	1.63	1.25	0.11	0.24	0.04	0.34	0.15	0.87
4756	Ar2	0.55	0.84	0.32	0.22	0.04	0.3	0.15	1.02
4339	Ar2	1.38	1.34	0.54	0.16	0.03	0.19	0.09	0.99
4340	Ar2	1.06	1.38	0.84	0.16	0.03	0.19	0.11	0.89
4341	Ar2	1.25	1.29	0.76	0.22	0.04	0.3	0.13	0.45
4342	Ar2	1.72	1.23	0.49	0.24	0.02	0.3	0.13	0.91
4758	Ar2	0.6	2.05	0.2	0.2	0.05	0.28	0.06	1.42
4344	Ar2	1.34	1.16	0.57	0.19	0.04	0.24	0.09	1.01
4345	Ar2	1.97	1.27	0.21	0.24	0.02	0.28	0.15	0.85
4346	Ar2	1.52	0.99	0.31	0.2	0.05	0.28	0.13	0.63
4347	Ar2	1.87	1.23	0.17	0.23	0.03	0.28	0.15	0.74
4348	Ar2	1.19	1.14	0.57	0.2	0.04	0.28	0.13	0.73
4760	Ar2	0.56	0.26	0.47	0.28	0.02	0.32	0.22	0.99
4350	Ar2	0.99	1.01	0.83	0.19	0.03	0.24	0.11	0.64
4351	Ar2	2.16	1.21	0.09	0.21	0.03	0.26	0.13	0.57
4352	Ar2	0.94	0.6	0.78	0.21	0.03	0.24	0.13	0.82
4353	Ar2	1.35	1.4	0.5	0.23	0.04	0.26	0.11	0.58
4354	Ar2	1.56	1.21	0.41	0.21	0.02	0.26	0.15	0.63
4355	Ar2	1.16	0.86	0.86	0.34	0.05	0.43	0.15	0.52
4356	Ar2	1.76	1.03	0.6	0.27	0.03	0.3	0.22	0.84
4357	Ar2	1.24	1.25	0.81	0.27	0.03	0.32	0.19	0.43
4358	Ar2	0.57	1.14	1.073132	0.22	0.03	0.28	0.13	0.8
4359	Ar2	1.17	1.03	0.82	0.25	0.02	0.3	0.19	0.59
4360	Ar2	1.11	0.97	0.79	0.22	0.04	0.28	0.15	0.66
4765	Ar2	0.8	2.05	0.34	0.23	0.05	0.3	0.09	0.73
4362	Ar2	1.68	1.31	0.6	0.24	0.04	0.32	0.17	0.82
4363	Ar2	1.86	0.58	0.36	0.22	0.06	0.28	0.11	0.67
4364	Ar2	2.09	0.99	0.32	0.23	0.04	0.3	0.15	0.49
4365	Ar2	1.85	1.03	0.57	0.21	0.05	0.3	0.11	0.79
4767	Ar2	0.89	1.85	0.42	0.22	0.02	0.26	0.17	0.74
4367	Ar2	1.32	0.9	0.88	0.23	0.04	0.28	0.13	0.62
4368	Ar2	1.65	2.05	0.11	0.11	0.02	0.13	0.04	0.45
4369	Ar2	1.2	0.37	0.38	0.12	0.02	0.15	0.09	0.46
4370	Ar2	1.02	0.69	0.51	0.14	0.04	0.19	0.02	0.57
4371	Ar2	1.29	1.25	0.36	0.16	0.02	0.19	0.11	0.52
4372	Ar2	1.33	1.1	0.29	0.16	0.02	0.19	0.06	0.82
4373	Ar2	1.39	0.19	0.32	0.19	0.03	0.26	0.11	0.45

4374	Ar2	0.86	0.13	0.79	0.16	0.02	0.19	0.11	0.51
4375	Ar2	1.61	1.29	0.22	0.15	0.02	0.19	0.09	0.71
4376	Ar2	1.45	1.36	0.36	0.15	0.02	0.19	0.11	1.05
4377	Ar2	1.34	1.25	0.48	0.15	0.03	0.19	0.09	0.84
4768	Ar2	0.71	2.17	0.39	0.16	0.03	0.22	0.11	1.23
4379	Ar2	1.15	1.51	0.52	0.17	0.02	0.22	0.11	0.63
4380	Ar2	1.19	1.49	0.49	0.17	0.02	0.22	0.13	0.92
4381	Ar2	1.35	1.49	0.43	0.19	0.03	0.26	0.13	0.62
4382	Ar2	1.1	1.49	0.66	0.19	0.02	0.24	0.13	0.6
4383	Ar2	1.52	1.49	0.4	0.18	0.02	0.24	0.15	0.77
4384	Ar2	1.07	1.12	0.64	0.17	0.03	0.22	0.09	0.93
4385	Ar2	2.31	0.19	0.06	0.16	0.03	0.22	0.11	0.45
4386	Ar2	1.5	1.25	0.47	0.15	0.02	0.19	0.09	0.68
4387	Ar2	1.48	1.4	0.49	0.18	0.02	0.22	0.13	0.37
4388	Ar2	1.96	1.25	0.11	0.16	0.04	0.19	0.09	0.52
4770	Ar2	0.53	1.96	0.36	0.17	0.04	0.22	0.11	1.16
4390	Ar2	0.82	0.26	0.79	0.18	0.02	0.24	0.13	0.46
4391	Ar2	0.93	1.23	0.77	0.15	0.03	0.19	0.09	0.33
4392	Ar2	1.41	1.12	0.39	0.13	0.02	0.17	0.09	0.87
4393	Ar2	1.06	0.95	0.68	0.15	0.03	0.19	0.06	0.85
4394	Ar2	1.43	1.12	0.46	0.19	0.03	0.24	0.06	0.47
4395	Ar2	1.08	1.03	0.68	0.17	0.03	0.24	0.09	0.49
4396	Ar2	1.46	1.21	0.64	0.14	0.03	0.19	0.09	0.6
4397	Ar2	1.63	1.49	0.53	0.16	0.03	0.22	0.06	0.66
4398	Ar2	1.82	1.49	0.34	0.14	0.03	0.22	0.09	0.69
4399	Ar2	2.15	1.4	0.23	0.15	0.03	0.19	0.11	0.67
4400	Ar2	1.35	1.62	0.56	0.13	0.02	0.15	0.06	0.79
4401	Ar2	1.94	1.44	0.46	0.13	0.02	0.17	0.09	0.61
4402	Ar2	1.54	1.42	0.67	0.16	0.02	0.19	0.09	1.01
4403	Ar2	1.37	1.51	0.79	0.15	0.03	0.19	0.06	1
4404	Ar2	1.22	1.18	0.85	0.16	0.02	0.22	0.09	0.84
4405	Ar2	1.94	1.81	0.56	0.16	0.04	0.24	0.09	0.58
4406	Ar2	1.89	1.38	0.58	0.16	0.03	0.24	0.11	1.08
4407	Ar2	1.7	1.36	0.68	0.18	0.04	0.26	0.13	0.65
4408	Ar2	1.06	1.1	0.99	0.19	0.03	0.24	0.11	0.97
4409	Ar2	1.25	1.36	0.66	0.16	0.03	0.19	0.09	1.07
4410	Ar2	1.19	1.46	0.72	0.18	0.03	0.22	0.11	0.88
4411	Ar2	1.86	1.74	0.35	0.16	0.04	0.26	0.06	0.67
4412	Ar2	1.48	1.23	0.68	0.15	0.03	0.22	0.09	0.95
4413	Ar2	1.35	1.44	0.68	0.17	0.04	0.24	0.09	1.35
4414	Ar2	1.62	1.59	0.52	0.16	0.03	0.24	0.11	1.33
4415	Ar2	1.23	1.53	0.88	0.14	0.02	0.19	0.09	1.16
4416	Ar2	1.84	1.64	0.5	0.18	0.04	0.26	0.09	0.8
4417	Ar2	2.31	1.59	0.13	0.15	0.03	0.17	0.04	0.53
4418	Ar2	1.48	1.51	0.47	0.15	0.03	0.22	0.06	0.86
4419	Ar2	1.35	1.64	0.48	0.15	0.02	0.19	0.11	1.17
4420	Ar2	1.49	1.66	0.54	0.14	0.03	0.17	0.04	1.09
4421	Ar2	1.09	1.34	0.78	0.14	0.02	0.19	0.09	1.35
4775	Ar2	0.61	1.89	0.37	0.21	0.04	0.28	0.13	0.95
4423	Ar2	1.13	1.44	0.68	0.19	0.04	0.28	0.11	0.96
4776	Ar2	0.48	1.87	0.381247	0.2	0.05	0.24	0.11	1.3
4425	Ar2	1.84	1.21	0.32	0.21	0.04	0.26	0.15	0.5
4785	Ar2	1.35	1.64	0.26	0.18	0.03	0.22	0.09	0.59
4427	Ar2	1.78	1.44	0.36	0.17	0.03	0.24	0.11	1.03

4428	Ar2	0.88	1.36	0.75	0.14	0.03	0.22	0.11	0.97
4429	Ar2	0.99	1.1	0.72	0.17	0.03	0.22	0.11	1.1
4430	Ar2	1.94	1.31	0.08	0.14	0.04	0.32	0.09	0.88
4805	Ar2	0.89	0.15	0.48	0.2	0.03	0.28	0.13	0.29
4432	Ar2	1.41	1.51	0.42	0.18	0.04	0.26	0.11	0.89
4433	Ar2	0.89	1.49	0.44	0.16	0.03	0.19	0.09	1.06
4434	Ar2	0.98	2	0.52	0.15	0.03	0.19	0.06	0.74
4435	Ar2	0.98	1.27	0.53	0.14	0.02	0.17	0.11	1.12
4815	Ar2	0.79	1.12	0.42	0.21	0.05	0.28	0.11	0.74
4823	Ar2	0.83	0.82	0.58	0.21	0.04	0.28	0.13	0.73
4828	Ar2	0.86	0.82	0.54	0.19	0.04	0.26	0.11	0.76
4839	Ar2	0.75	0.97	0.51	0.2	0.04	0.26	0.11	0.5
4440	Ar2	1.45	1.57	0.28	0.2	0.05	0.26	0.06	0.77
4441	Ar2	1.04	1.14	0.84	0.11	0.02	0.13	0.06	0.87
4442	Ar2	2.27	1.31	0.17	0.15	0.02	0.19	0.09	0.61
4443	Ar2	1.88	1.64	0.38	0.16	0.03	0.19	0.11	0.41
4444	Ar2	1.61	1.14	0.53	0.16	0.03	0.19	0.11	0.61
4445	Ar2	1.99	1.23	0.37	0.18	0.05	0.24	0.09	0.52
4446	Ar2	2.37	0.88	0.12	0.21	0.05	0.26	0.09	0.57
4447	Ar2	1.84	0.88	0.36	0.18	0.02	0.24	0.13	0.54
4448	Ar2	2.12	0.86	0.18	0.17	0.02	0.22	0.11	0.55
4449	Ar2	2.19	1.1	0.14	0.2	0.02	0.24	0.11	0.43
4859	Ar2	0.76	0.95	0.34	0.13	0.02	0.15	0.09	0.33
4451	Ar2	1.87	0.84	0.62	0.18	0.03	0.24	0.11	0.69
4452	Ar2	1.88	1.23	0.78	0.21	0.04	0.26	0.13	0.45
4453	Ar2	1.65	1.25	0.81	0.21	0.04	0.26	0.11	0.48
4454	Ar2	1.96	1.12	0.47	0.15	0.03	0.19	0.09	0.7
4455	Ar2	1.42	0.11	0.95	0.2	0.03	0.26	0.13	0.42
4456	Ar2	1.76	1.12	0.63	0.17	0.02	0.22	0.13	0.57
4457	Ar2	1.2	0.75	0.88	0.15	0.03	0.22	0.11	0.62
4458	Ar2	1.41	0.84	0.9	0.16	0.02	0.19	0.11	0.69
4459	Ar2	1.44	1.1	0.89	0.17	0.03	0.22	0.11	0.29
4460	Ar2	1.74	0.82	0.65	0.16	0.02	0.19	0.11	0.79
4461	Ar2	2.86	0.99	0.39	0.25	0.03	0.28	0.19	0.51
4462	Ar2	1.52	1.08	1.11	0.22	0.05	0.28	0.13	0.52
4868	Ar2	0.68	0.3	0.55	0.16	0.02	0.17	0.13	0.51
4464	Ar2	1.62	1.01	1.26	0.25	0.04	0.3	0.15	0.34
4465	Ar2	1.98	1.16	0.76	0.2	0.04	0.26	0.13	0.62
4466	Ar2	1.48	1.18	1.06	0.21	0.04	0.28	0.13	0.56
4467	Ar2	1.55	1.42	1	0.21	0.1	0.37	0.04	0.44
4468	Ar2	2.13	1.1	0.78	0.23	0.05	0.3	0.11	0.58
4469	Ar2	1.94	0.22	0.92	0.2	0.03	0.26	0.15	0.33
4470	Ar2	2.57	1.38	0.36	0.16	0.03	0.19	0.06	0.6
4471	Ar2	1.73	0.52	0.83	0.16	0.02	0.19	0.11	0.74
4472	Ar2	1.24	1.03	1.02	0.16	0.03	0.19	0.09	0.73
4473	Ar2	1.68	1.03	0.99	0.15	0.03	0.19	0.09	0.75
4474	Ar2	1.59	1.23	0.93	0.16	0.03	0.19	0.09	0.73
4475	Ar2	1.52	1.18	0.99	0.16	0.02	0.19	0.11	0.75
4476	Ar2	3.01	0.17	0.23	0.13	0.02	0.15	0.09	0.43
4477	Ar2	2.25	1.25	0.81	0.16	0.03	0.19	0.09	0.38
4478	Ar2	1.86	0.97	1	0.14	0.04	0.19	0.06	0.51
4479	Ar2	2.09	2.15	0.81	0.17	0.02	0.22	0.13	0.28
4480	Ar2	1.83	1.34	0.71	0.16	0.03	0.22	0.09	0.67
4481	Ar2	1.5	1.31	1.04	0.16	0.03	0.19	0.11	0.5

4482	Ar2	1.58	0.6	0.82	0.18	0.03	0.24	0.13	0.67
4483	Ar2	1.4	1.21	0.83	0.18	0.04	0.22	0.11	0.44
4484	Ar2	1.51	1.27	0.68	0.22	0.03	0.28	0.11	0.55
4485	Ar2	1.21	1.14	0.84	0.18	0.03	0.24	0.11	0.65
4486	Ar2	1.41	1.23	0.87	0.19	0.04	0.24	0.11	1.11
4487	Ar2	1.77	1.14	0.78	0.18	0.03	0.26	0.13	0.77
4488	Ar2	1.4	1.27	0.88	0.21	0.03	0.24	0.11	0.74
4489	Ar2	1.41	1.49	0.94	0.19	0.04	0.26	0.11	0.74
4490	Ar2	1.45	1.29	1.01	0.18	0.03	0.24	0.11	0.6
4491	Ar2	1.44	1.23	0.93	0.16	0.04	0.22	0.09	0.65
4492	Ar2	1.8	0.24	1.43	0.12	0.02	0.13	0.06	0.36
4493	Ar2	1.94	0.6	1.31	0.14	0.02	0.17	0.09	0.65
4494	Ar2	2.92	1.25	0.56	0.15	0.02	0.17	0.09	0.57
4495	Ar2	1.71	1.55	1.21	0.14	0.02	0.17	0.11	0.53
4496	Ar2	2.29	1.03	1.02	0.16	0.02	0.17	0.11	0.53
4497	Ar2	1.5	0.45	1.47	0.16	0.02	0.19	0.11	0.52
4498	Ar2	1.66	1.18	1.43	0.15	0.03	0.22	0.09	0.38
4499	Ar2	1.62	0.15	1.49	0.18	0.04	0.26	0.09	0.23
4500	Ar2	2.09	0.93	1.24	0.15	0.04	0.22	0.06	0.4
4501	Ar2	2.24	0.88	1.08	0.14	0.04	0.19	0.06	0.37
4502	Ar2	2.78	1.25	0.61	0.15	0.03	0.19	0.11	0.3
4503	Ar2	2.26	0.37	1.07	0.12	0.02	0.15	0.09	0.4
4879	Ar2	0.6	0.97	0.45	0.14	0.02	0.17	0.09	0.54
4505	Ar2	2.56	1.08	1.04	0.27	0.03	0.32	0.19	0.8
4506	Ar2	2.99	1.46	0.73	0.18	0.03	0.24	0.11	0.44
4507	Ar2	2.02	0.93	1.21	0.18	0.03	0.26	0.11	0.76
4508	Ar2	1.7	0.84	1.46	0.16	0.05	0.26	0.06	0.53
4509	Ar2	2.19	1.01	1.25	0.19	0.04	0.28	0.11	0.73
4510	Ar2	2.31	1.59	0.93	0.21	0.03	0.26	0.15	0.38
4511	Ar2	1.99	0.37	1.15	0.2	0.02	0.28	0.13	0.43
4512	Ar2	2.49	0.9	0.79	0.26	0.06	0.32	0.11	0.49
4885	Ar2	0.65	0.19	0.39	0.18	0.04	0.26	0.09	0.29
4514	Er	1.61	1.21	1.22	0.19	0.05	0.3	0.11	0.24
4515	Ar2	1.4	1.29	1.02	0.19	0.05	0.28	0.11	1.04
4889	Ar2	0.8	0.95	0.2	0.19	0.03	0.22	0.13	0.62
4517	Ar2	1.55	1.74	0.87	0.18	0.02	0.24	0.13	0.88
4917	Ar2	0.5	0.22	0.48	0.25	0.04	0.3	0.17	0.88
4519	Ar2	1.61	1.59	0.92	0.25	0.04	0.3	0.15	1.06
4520	Ar2	1.32	1.21	1.17	0.23	0.05	0.3	0.15	1.03
4928	Ar2	0.6	0.8	0.52	0.25	0.06	0.34	0.17	0.64
4930	Ar2	0.8	0.3	0.47	0.29	0.04	0.37	0.19	0.37
4523	Ar2	1.72	1.53	1.06	0.16	0.03	0.19	0.09	0.82
4524	Ar2	1.85	1.38	1.37	0.22	0.04	0.26	0.11	0.78
4525	Ar2	2.52	1.87	0.92	0.18	0.03	0.22	0.09	0.6
4526	Ar2	1.75	1.89	1.24	0.24	0.04	0.3	0.15	0.94
4527	Ar2	2.14	1.4	1.18	0.23	0.05	0.34	0.13	0.85
4940	Ar2	1.02	1.08	0.46	0.27	0.06	0.39	0.17	0.81
4529	Ar2	1.47	2.05	1.3	0.2	0.02	0.24	0.13	0.42
4530	Ar2	1.79	1.38	1.14	0.21	0.02	0.28	0.11	0.72
4531	Ar2	2.01	1.42	1.03	0.16	0.03	0.22	0.09	0.97
4532	Ar2	1.6	1.57	1.01	0.2	0.04	0.26	0.06	1.14
4533	Ar2	2.12	1.51	0.82	0.2	0.03	0.26	0.17	0.97
4534	Ar2	1.95	1.31	1.23	0.2	0.03	0.28	0.11	0.69
4535	Ar2	2.11	1.38	1.1	0.15	0.03	0.19	0.11	0.66

4536	Ar2	2.15	1.21	0.9	0.2	0.04	0.28	0.09	0.65
4537	Ar2	1.8	1.16	1.02	0.2	0.03	0.24	0.11	0.83
4538	Ar2	2.19	1.27	1.01	0.2	0.03	0.26	0.13	0.6
4539	Ar2	1.78	1.36	1.33	0.21	0.04	0.28	0.13	0.56
4540	Ar2	0.87	1.4	0.34	0.21	0.04	0.3	0.13	0.89
4541	Ar2	0.67	1.25	0.35	0.24	0.04	0.3	0.13	1.07
4542	Ar2	0.71	1.29	0.41	0.23	0.03	0.3	0.17	0.73
4957	Ar2	0.89	0.41	0.72	0.34	0.08	0.41	0.19	0.46
4544	Ar2	0.75	1.14	0.23	0.2	0.04	0.26	0.11	1.03
4962	Ar2	1.08	1.36	0.37	0.29	0.05	0.37	0.19	0.85
4546	Ar2	0.55	0.86	0.49	0.24	0.04	0.28	0.17	0.76
4547	Ar2	0.79	1.46	0.25	0.26	0.03	0.3	0.15	0.89
4548	Ar2	0.58	1.14	0.35	0.21	0.04	0.28	0.15	1
4549	Ar2	0.68	1.4	0.35	0.21	0.03	0.26	0.15	1
4550	Ar2	0.63	1.46	0.3	0.2	0.03	0.26	0.15	1.12
4551	Ar2	0.52	1.23	0.42	0.21	0.03	0.26	0.17	0.9
4552	Ar2	0.7	1.49	0.27	0.15	0.03	0.22	0.09	0.98
4553	Ar2	0.71	1.29	0.4	0.17	0.02	0.26	0.11	0.93
4968	Ar2	0.78	1.51	0.25	0.35	0.06	0.41	0.19	0.92
4555	Ar2	0.78	1.51	0.32	0.18	0.03	0.24	0.11	0.84
4556	Ar2	1	0.3	0.26	0.27	0.05	0.34	0.13	0.35
4557	Ar2	0.6	0.84	0.53	0.19	0.06	0.3	0.06	0.68
4558	Ar2	1.01	1.57	0.18	0.2	0.04	0.26	0.13	0.72
4559	Ar2	1.05	1.31	0.23	0.21	0.03	0.26	0.15	0.71
4560	Ar2	0.71	1.66	0.2	0.2	0.04	0.28	0.13	0.94
4561	Ar2	0.49	1.55	0.25	0.21	0.05	0.28	0.11	0.97
4562	Ar2	0.66	1.49	0.21	0.22	0.03	0.26	0.15	1.14
4563	Ar2	0.6	1.89	0.24	0.21	0.04	0.26	0.13	1.08
4564	Ar2	0.59	1.7	0.29	0.22	0.03	0.28	0.15	1.16
4565	Ar2	0.68	1.42	0.34	0.22	0.04	0.3	0.15	0.81
4566	Ar2	0.59	1.36	0.34	0.22	0.03	0.28	0.15	0.99
4567	Ar2	0.96	1.59	0.1	0.22	0.03	0.3	0.13	0.57
4568	Ar2	0.71	1.29	0.28	0.17	0.04	0.26	0.13	0.93
4569	Ar2	0.58	1.57	0.31	0.2	0.03	0.24	0.15	1.03
4570	Ar2	0.51	1.14	0.36	0.2	0.03	0.24	0.15	1
4571	Ar2	0.52	1.46	0.33	0.19	0.03	0.24	0.13	1.01
4572	Ar2	0.6	1.77	0.35	0.21	0.03	0.26	0.15	0.58
4573	Ar2	0.49	1.21	0.4	0.2	0.03	0.24	0.13	1.18
4574	Ar2	0.5	1.81	0.35	0.17	0.02	0.19	0.11	1.02
4575	Ar2	0.48	1.64	0.34	0.17	0.02	0.22	0.13	0.99
4576	Ar2	0.44	1.55	0.25	0.14	0.02	0.22	0.11	1.23
4577	Ar2	0.44	1.21	0.3	0.18	0.03	0.22	0.11	1.06
4578	Ar2	0.53	1.38	0.25	0.16	0.03	0.22	0.11	0.93
4579	Ar2	0.86	1.79	0.05	0.17	0.06	0.3	0.09	0.46
4969	Ar2	0.71	1.38	0.39	0.3	0.06	0.45	0.19	0.97
4581	Ar2	0.46	1.23	0.38	0.18	0.02	0.24	0.15	0.8
4972	Ar2	0.58	1.38	0.33	0.22	0.09	0.34	0.09	0.94
4583	Ar2	0.42	1.31	0.28	0.17	0.03	0.24	0.13	0.89
4584	Ar2	0.48	1.57	0.2	0.18	0.03	0.24	0.13	0.97
4585	Haer	0.64	0.24	0.19	0.17	0.04	0.24	0.06	0.24
4586	Er	0.14	0.22	0.29	0.11	0.04	0.15	0.06	0.73
4587	Haer	0.5	1.74	0.1	0.14	0.04	0.19	0.06	0.46
4588	Ar2	0.7	1.38	0.09	0.14	0.01	0.17	0.09	0.78
4589	Ar2	0.45	0.52	0.27	0.17	0.03	0.22	0.11	0.5

4977	Ar2	0.79	1.42	0.44	0.27	0.04	0.34	0.17	1.01
4591	Ar2	0.72	1.18	0.1	0.15	0.03	0.19	0.11	0.61
4592	Ar2	0.46	0.69	0.15	0.17	0.04	0.24	0.09	0.54
4593	Ar2	0.33	1.38	0.1	0.15	0.03	0.22	0.06	0.65
4594	Ar2	0.35	1.14	0.23	0.18	0.03	0.24	0.13	1.09
5001	Ar2	0.92	0.67	0.64	0.21	0.03	0.26	0.15	0.58
4596	Ar2	0.42	1.27	0.19	0.16	0.03	0.22	0.13	0.91
5003	Er	0.94	0.73	0.58	0.15	0.04	0.24	0.09	0.38
4598	Ar2	0.38	0.84	0.26	0.18	0.04	0.26	0.11	0.87
4599	Ar2	0.47	1.25	0.1	0.18	0.04	0.24	0.11	0.8
4600	Haer	0.56	1.85	0.11	0.18	0.04	0.24	0.09	0.48
4601	Ar2	0.59	1.55	0.12	0.17	0.03	0.24	0.11	0.66
4602	Ar2	0.45	1.51	0.21	0.16	0.02	0.19	0.11	0.85
4603	Ar2	0.6	1.44	0.19	0.18	0.04	0.26	0.09	0.37
4604	Ar2	0.64	1.38	0.18	0.17	0.02	0.19	0.13	0.69
4605	Ar2	0.49	1.31	0.32	0.16	0.03	0.22	0.13	0.51
4606	Ar2	0.93	1.16	0.19	0.13	0.02	0.15	0.06	0.88
4607	Ar2	1.38	0.19	0.04	0.17	0.03	0.22	0.09	0.45
4608	Ar2	0.65	0.17	0.54	0.17	0.03	0.24	0.11	0.33
4609	Ar2	0.83	0.15	0.48	0.16	0.02	0.19	0.13	0.38
4610	Ar2	1.04	0.15	0.17	0.16	0.02	0.22	0.11	0.29
4611	Haer	0.8	0.39	0.17	0.18	0.04	0.24	0.11	0.26
4612	Ar2	0.74	0.13	0.25	0.16	0.05	0.26	0.06	0.34
4613	Ar2	0.67	1.34	0.36	0.14	0.03	0.17	0.09	0.8
4614	Ar2	0.95	1.34	0.18	0.14	0.02	0.17	0.04	1.03
4615	Ar2	0.76	1.38	0.37	0.13	0.02	0.19	0.11	1.14
4616	Ar2	1.03	1.57	0.25	0.15	0.03	0.22	0.09	0.61
4617	Ar2	0.53	1.42	0.26	0.15	0.03	0.22	0.11	0.7
5014	Ar2	0.76	0.71	0.73	0.21	0.02	0.26	0.15	0.52
4619	Ar2	0.59	1.36	0.28	0.17	0.02	0.22	0.11	0.53
4620	Ar2	0.51	1.51	0.25	0.13	0.04	0.19	0.06	1.06
4621	Ar2	0.52	1.55	0.3	0.17	0.03	0.22	0.09	0.82
5024	Ar2	0.61	0.88	0.36	0.27	0.04	0.37	0.17	0.57
5026	Ar2	0.67	0.34	0.64	0.27	0.02	0.32	0.24	0.45
4624	Ar2	0.76	1.49	0.23	0.16	0.03	0.19	0.09	0.74
4625	Ar2	0.5	1.68	0.29	0.13	0.02	0.15	0.11	1.01
4626	Ar2	0.55	1.44	0.24	0.16	0.03	0.19	0.09	0.72
4627	Ar2	0.58	1.4	0.29	0.15	0.01	0.17	0.11	0.84
4628	Ar2	0.48	1.42	0.24	0.14	0.02	0.17	0.09	0.59
4629	Ar2	0.69	1.38	0.21	0.13	0.03	0.17	0.06	0.45
4630	Ar2	0.31	1.29	0.24	0.23	0.04	0.3	0.15	1.23
5034	Ar2	0.87	1.08	0.36	0.24	0.04	0.3	0.13	0.67
4632	Ar2	0.51	1.55	0.15	0.23	0.04	0.37	0.15	1
4633	Ar2	0.47	1.53	0.21	0.26	0.05	0.34	0.15	1.19
4634	Ar2	0.44	1.59	0.32	0.26	0.05	0.32	0.17	1.11
4635	Ar2	0.59	1.77	0.26	0.27	0.03	0.3	0.22	0.55
5039	Ar2	0.7	0.75	0.66	0.22	0.04	0.32	0.15	0.51
4637	Ar2	0.48	1.72	0.23	0.24	0.02	0.28	0.17	1.5
4638	Ar2	0.48	1.77	0.24	0.29	0.06	0.37	0.17	1.24
5040	Ar2	1.3	0.78	0.35	0.21	0.06	0.3	0.09	0.62
5048	Er	0.72	0.73	0.57	0.23	0.03	0.28	0.17	0.37
5049	Ar2	0.9	0.67	0.42552	0.2	0.04	0.3	0.13	0.43
4642	Ar2	0.47	1.51	0.22	0.24	0.04	0.3	0.17	1.43
4643	Er	0.51	1.96	0.25	0.26	0.03	0.3	0.19	1.29

4644	Ar2	0.47	1.64	0.25	0.24	0.05	0.32	0.15	1.21
4645	Ar2	0.45	1.94	0.21	0.23	0.03	0.26	0.17	1.09
4646	Ar2	0.42	1.49	0.22	0.24	0.03	0.28	0.15	1.47
4647	Ar2	0.56	1.79	0.17	0.21	0.04	0.28	0.15	1.24
5067	Ar2	0.55	1.34	0.26	0.33	0.04	0.43	0.19	0.52
4649	Ar2	0.79	1.42	0.2	0.25	0.04	0.3	0.17	1.27
4650	Ar2	0.53	1.27	0.41	0.26	0.03	0.3	0.19	1.3
5070	Ar2	0.49	1.14	0.23	0.33	0.05	0.41	0.22	0.84
4652	Ar2	0.66	1.51	0.23	0.26	0.04	0.32	0.17	1.39
4653	Ar2	0.69	1.57	0.37	0.21	0.04	0.26	0.15	1.2
4654	Ar2	0.71	1.64	0.29	0.22	0.03	0.28	0.15	1.19
4655	Ar2	0.61	1.06	0.4	0.21	0.04	0.28	0.15	1.15
5078	Ar2	0.4	0.78	0.33	0.37	0.07	0.45	0.24	0.75
4657	Ar2	0.82	1.62	0.23	0.24	0.04	0.32	0.13	1.26
4658	Ar2	1.11	1.53	0.12	0.23	0.04	0.3	0.15	1.19
4659	Ar2	0.84	1.83	0.25	0.24	0.04	0.3	0.17	1.14
4660	Ar2	0.58	0.99	0.45	0.27	0.04	0.32	0.15	1.14
5082	Ar2	0.46	0.45	0.33	0.36	0.11	0.47	0.15	0.56
5083	Ar2	0.54	0.71	0.15	0.33	0.09	0.47	0.17	0.75
4663	Ar2	0.5	1.42	0.34	0.23	0.07	0.37	0.09	1.15
4664	Ar2	0.69	1.53	0.26	0.25	0.02	0.32	0.19	1.4
4665	Ar2	0.57	1.53	0.43	0.25	0.03	0.3	0.19	1.35
4666	Ar2	0.71	1.59	0.29	0.26	0.03	0.34	0.17	1.42
4667	Ar2	0.59	1.62	0.35	0.24	0.03	0.37	0.15	1.4
5086	Ar2	0.54	0.43	0.26	0.41	0.05	0.5	0.28	0.54
4669	Ar2	0.61	1.25	0.52	0.23	0.03	0.3	0.17	1.35
4670	Ar2	0.66	1.01	0.49	0.27	0.05	0.34	0.15	1.42
4671	Ar2	0.73	1.57	0.44	0.25	0.02	0.34	0.19	1.4
4672	Ar2	0.68	1.98	0.4	0.25	0.03	0.32	0.17	1.39
5095	Ar2	0.45	0.39	0.34	0.37	0.02	0.41	0.34	0.38
5097	Ar2	0.56	0.73	0.45	0.36	0.05	0.43	0.24	0.5
4675	Ar2	0.56	1.66	0.42	0.23	0.04	0.28	0.15	1.28
5098	Ar2	0.54	0.78	0.41	0.37	0.06	0.43	0.22	0.54
4677	Ar2	0.46	1.38	0.38	0.23	0.04	0.32	0.17	1.41
5107	Er	0.11	0.88	0.04	0.26	0.03	0.3	0.22	0.45
4679	Ar2	0.6	1.59	0.36	0.24	0.03	0.34	0.15	1.47
4680	Ar2	0.7	1.59	0.28	0.22	0.04	0.34	0.13	1.44
4681	Ar2	0.71	1.59	0.42	0.23	0.03	0.28	0.13	1.32
4682	Ar2	0.78	1.49	0.26	0.26	0.07	0.34	0.11	0.81
4683	Ar2	0.76	1.59	0.31	0.23	0.02	0.26	0.17	1.46
4684	Ar2	0.71	1.53	0.32	0.25	0.03	0.32	0.15	1.34
5109	Er	0.15	0.97	0.07	0.29	0.03	0.32	0.24	0.52
4686	Ar2	0.43	1.85	0.19	0.19	0.03	0.24	0.13	1.16
4687	Ar2	0.36	1.87	0.19	0.18	0.03	0.22	0.13	1.31
5111	Ar2	0.26	0.73	0.08	0.27	0.05	0.34	0.17	0.58
4689	Ar2	0.39	1.53	0.26	0.19	0.02	0.22	0.15	1.05
4690	Ar2	0.32	1.57	0.22	0.18	0.03	0.24	0.15	0.82
4691	Haer	0.2	0.11	0.08	0.2	0.03	0.28	0.15	0.68
4692	Ar2	0.36	1.81	0.17	0.18	0.02	0.24	0.13	1.19
4693	Haer	0.33	2.22	0.19	0.18	0.03	0.24	0.11	0.81
4694	Ar2	0.38	1.72	0.18	0.17	0.02	0.19	0.13	1.19
5113	Er	0.26	0.73	0.05	0.35	0.03	0.39	0.3	0.4
4696	Ar2	0.5	1.64	0.16	0.2	0.04	0.3	0.13	0.8
4697	Ar2	0.47	1.31	0.15	0.2	0.03	0.24	0.15	1.04

4698	Ar2	0.24	1.59	0.17	0.2	0.03	0.26	0.15	1.41
5115	Er	0.24	0.65	0.07	0.31	0.04	0.34	0.22	0.53
4700	Ar2	0.41	1.64	0.14	0.22	0.04	0.3	0.13	0.67
4701	Ar2	0.43	1.62	0.2	0.16	0.02	0.19	0.13	1.05
5116	Er	0.24	0.86	0.14	0.29	0.04	0.34	0.24	0.71
4703	Haer	0.37	2.37	0.15	0.17	0.04	0.26	0.09	0.36
4704	Er	0.23	0.41	0.2	0.18	0.05	0.22	0.09	0.96
4705	Ar2	0.47	1.62	0.23	0.18	0.02	0.19	0.13	0.99
4706	Ar2	0.42	2.37	0.21	0.16	0.02	0.22	0.13	0.71
5118	Ar2	0.34	0.88	0.09	0.28	0.09	0.43	0.17	0.49
5128	Ar2	0.38	0.39	0.18	0.4	0.03	0.5	0.37	0.39
4709	Ar2	1.05	1.46	0.35	0.18	0.04	0.24	0.09	1.1
4710	Ar2	0.66	2.3	0.4	0.19	0.03	0.26	0.15	0.61
4711	Ar2	0.66	1.44	0.44	0.17	0.03	0.22	0.11	1.2
4712	Ar2	0.62	1.83	0.5	0.18	0.03	0.24	0.13	1.03
5130	Er	0.08	0.78	0.07	0.33	0.09	0.45	0.22	0.63
5132	Ar2	0.27	0.41	0.21	0.43	0.03	0.47	0.39	0.98
4715	Ar2	1.15	1.68	0.25	0.19	0.04	0.26	0.11	0.81
4716	Ar2	0.5	1.42	0.35	0.2	0.03	0.26	0.15	0.97
4717	Ar2	0.41	1.46	0.33	0.19	0.02	0.22	0.15	1.13
4718	Ar2	0.44	1.77	0.29	0.2	0.02	0.24	0.15	1.27
4719	Ar2	0.47	1.36	0.25	0.19	0.03	0.26	0.11	1.16
5136	Ar2	0.4	0.39	0.2	0.39	0.03	0.5	0.34	0.46
5140	Ar2	0.2	1.1	0.17	0.24	0.03	0.28	0.19	0.6
4722	Ar2	0.63	1.66	0.31	0.18	0.03	0.26	0.13	1.16
4723	Ar2	0.61	1.74	0.32	0.22	0.03	0.26	0.11	1.3
4724	Ar2	0.59	1.62	0.27	0.18	0.03	0.22	0.13	1.33
5141	Ar2	0.25	0.3	0.18	0.27	0.03	0.3	0.22	0.54
4726	Ar2	0.49	1.49	0.36	0.2	0.03	0.26	0.17	1.11
4727	Ar2	0.91	0.13	0.12	0.17	0.04	0.24	0.09	0.97
5142	Ar2	0.16	1.08	0.13	0.25	0.02	0.28	0.22	0.56
5143	Ar2	0.28	0.22	0.07	0.26	0.05	0.32	0.15	0.45
4730	Ar2	0.68	1.64	0.28	0.15	0.03	0.24	0.09	0.83
4731	Ar2	0.7	1.55	0.19	0.18	0.03	0.24	0.11	1.26
4732	Ar2	0.58	1.51	0.27	0.19	0.04	0.26	0.09	0.97
5146	Ar2	0.24	0.97	0.06	0.27	0.04	0.34	0.22	0.79
4734	Ar2	0.93	1.51	0.15	0.2	0.04	0.26	0.11	1.06
4735	Ar2	0.5	1.42	0.31	0.16	0.02	0.22	0.11	1.3
4736	Ar2	0.56	1.85	0.35	0.16	0.03	0.19	0.11	1.07
4737	Ar2	0.81	1.55	0.21	0.17	0.03	0.22	0.11	0.78
5149	Ar2	0.34	1.14	0.17	0.26	0.03	0.3	0.19	0.77
4739	Ar2	0.69	1.44	0.28	0.17	0.03	0.24	0.11	1.04
5159	Ar2	0.43	0.22	0.08	0.25	0.06	0.39	0.17	0.32
4741	Ar2	0.44	2.2	0.26	0.18	0.03	0.24	0.13	0.9
4742	Ar2	0.87	1.49	0.04	0.21	0.03	0.24	0.11	0.96
4743	Ar2	0.45	1.44	0.27	0.19	0.03	0.22	0.11	1.03
4744	Ar2	0.42	1.79	0.23	0.15	0.01	0.17	0.13	1.56
4745	Ar2	0.45	1.72	0.29	0.18	0.03	0.24	0.15	1.07
4746	Ar2	0.58	2.05	0.28	0.13	0.03	0.19	0.04	1.29
4747	Ar2	0.49	1.83	0.3	0.16	0.02	0.19	0.11	1.32
4748	Ar2	0.69	1.49	0.21	0.19	0.03	0.22	0.09	1.17
4749	Ar2	0.93	2.07	0.08	0.19	0.02	0.22	0.15	1.02
4750	Ar2	0.66	2.56	0.15	0.15	0.02	0.17	0.04	0.43
4751	Ar2	0.78	1.96	0.36	0.13	0.03	0.17	0.06	1.05

4752	Ar2	0.87	1.87	0.27	0.14	0.03	0.17	0.04	0.79
4753	Ar2	0.59	1.55	0.27	0.19	0.02	0.26	0.15	1.21
4754	Ar2	0.43	2	0.27	0.19	0.03	0.24	0.13	1.34
4755	Ar2	0.51	1.74	0.29	0.21	0.03	0.26	0.15	1.24
5163	Ar2	0.52	0.71	0.06	0.24	0.06	0.37	0.15	0.52
4757	Ar2	0.6	1.59	0.21	0.22	0.03	0.3	0.09	1.46
5164	Er	0.22	0.97	0.17	0.22	0.06	0.32	0.13	0.74
4759	Ar2	0.56	2.63	0.25	0.21	0.04	0.26	0.13	0.96
5169	Ar2	0.3	0.8	0.24	0.24	0.04	0.32	0.17	0.71
4761	Ar2	0.47	1.64	0.25	0.21	0.04	0.26	0.13	1.46
4762	Ar2	0.42	1.74	0.34	0.21	0.03	0.28	0.17	1.25
4763	Ar2	0.55	1.74	0.29	0.21	0.04	0.26	0.15	1.39
4764	Ar2	0.49	1.12	0.4	0.22	0.03	0.28	0.15	1.36
5170	Ar2	0.38	0.6	0.26	0.29	0.04	0.32	0.19	0.41
4766	Ar2	1.02	1.89	0.21	0.19	0.04	0.26	0.11	1.08
5178	Ar2	0.65	1.57	0.12	0.21	0.04	0.28	0.15	0.74
5180	Ar2	0.53	1.38	0.26	0.24	0.04	0.28	0.13	0.77
4769	Ar2	0.63	1.57	0.3	0.18	0.03	0.22	0.11	1.17
5182	Ar2	0.4	0.24	0.38	0.24	0.07	0.34	0.06	0.52
4771	Ar2	0.57	1.81	0.36	0.18	0.02	0.22	0.13	1.13
4772	Ar2	0.67	1.7	0.28	0.18	0.03	0.28	0.11	1.29
4773	Ar2	0.6	1.72	0.28	0.19	0.03	0.24	0.13	1.35
4774	Ar2	0.58	1.21	0.46	0.21	0.03	0.26	0.13	1.35
5183	Ar2	0.64	1.03	0.14	0.2	0.06	0.28	0.06	0.71
5185	Ar2	0.5	0.9	0.23	0.26	0.06	0.32	0.17	0.56
4777	Ar2	0.93	0.39	0.44	0.15	0.03	0.19	0.11	0.56
4778	Ar2	1.11	0.88	0.3	0.17	0.02	0.19	0.13	0.65
4779	Ar2	0.84	0.37	0.5	0.18	0.03	0.22	0.11	0.52
4780	Ar2	1.43	1.34	0.17	0.17	0.02	0.19	0.11	0.52
4781	Ar2	0.88	0.95	0.51	0.14	0.02	0.17	0.11	0.82
4782	Ar2	0.8	1.18	0.55	0.15	0.02	0.19	0.11	0.51
4783	Ar2	1.01	1.16	0.49	0.14	0.03	0.19	0.09	0.72
4784	Ar2	0.82	0.82	0.58	0.15	0.03	0.22	0.06	0.81
5190	Ar2	0.44	1.4	0.17	0.26	0.03	0.3	0.17	0.88
4786	Ar2	0.82	0.99	0.6	0.16	0.04	0.22	0.06	0.75
4787	Ar2	1.03	1.12	0.39	0.14	0.02	0.17	0.11	1
4788	Ar2	0.76	0.88	0.52	0.15	0.04	0.22	0.06	0.79
4789	Ar2	0.72	1.12	0.62	0.14	0.02	0.19	0.11	0.53
4790	Ar2	0.74	0.84	0.55	0.16	0.02	0.19	0.09	0.67
4791	Ar2	0.7	0.73	0.66	0.15	0.03	0.19	0.11	0.65
4792	Ar2	1.04	1.23	0.47	0.13	0.02	0.17	0.11	0.75
4793	Ar2	0.91	1.14	0.56	0.21	0.04	0.26	0.13	0.74
4794	Ar2	1	1.1	0.59	0.2	0.06	0.26	0.11	0.91
4795	Ar2	0.97	0.86	0.54	0.28	0.07	0.32	0.06	0.75
4796	Ar2	1.67	0.95	0.22	0.19	0.03	0.24	0.13	0.53
4797	Ar2	1.67	1.1	0.09	0.2	0.04	0.24	0.09	0.8
4798	Ar2	1.03	1.18	0.55	0.22	0.02	0.26	0.13	0.8
4799	Ar2	1.17	1.18	0.42	0.18	0.02	0.22	0.13	0.97
4800	Ar2	1.04	1.16	0.35	0.17	0.04	0.26	0.11	0.9
4801	Ar2	0.83	1.29	0.45	0.18	0.03	0.24	0.11	0.93
4802	Ar2	0.75	1.29	0.5	0.18	0.03	0.22	0.13	0.65
4803	Ar2	0.95	1.12	0.42	0.18	0.02	0.22	0.11	0.92
4804	Ar2	0.75	1.21	0.53	0.16	0.03	0.26	0.09	0.78
5194	Ar2	0.18	0.97	0.03	0.3	0.03	0.34	0.24	0.92

4806	Ar2	1	1.12	0.38	0.18	0.05	0.24	0.11	0.58
4807	Ar2	0.98	0.62	0.41	0.2	0.03	0.24	0.15	0.5
4808	Ar2	0.81	0.47	0.49	0.21	0.05	0.28	0.06	0.68
4809	Ar2	0.78	0.82	0.52	0.19	0.04	0.26	0.09	0.64
4810	Roar	0.93	1.01	0.36	0.18	0.03	0.22	0.11	0.77
4811	Ar2	1.06	1.12	0.34	0.21	0.04	0.3	0.11	0.68
4812	Ar2	1.45	1.12	0.17	0.2	0.03	0.28	0.13	0.55
4813	Ar2	0.85	0.99	0.47	0.2	0.05	0.26	0.09	0.64
4814	Ar2	1.04	1.06	0.35	0.2	0.04	0.26	0.11	0.62
5195	Ar2	0.17	0.28	0.15	0.26	0.04	0.3	0.17	1.1
4816	Ar2	1.14	1.44	0.22	0.23	0.04	0.28	0.11	0.69
4817	Ar2	0.98	1.4	0.42	0.21	0.03	0.32	0.13	0.71
4818	Ar2	0.66	1.06	0.57	0.19	0.05	0.26	0.09	0.92
4819	Ar2	0.75	1.29	0.54	0.19	0.03	0.26	0.13	0.77
4820	Ar2	0.91	1.31	0.43	0.2	0.03	0.28	0.13	0.88
4821	Ar2	0.82	1.25	0.45	0.2	0.04	0.26	0.11	0.9
4822	Ar2	0.97	1.29	0.55	0.19	0.04	0.3	0.13	0.89
5196	Ar2	0.19	1.83	0.12	0.28	0.02	0.3	0.24	0.95
4824	Ar2	1.25	1.21	0.3	0.18	0.04	0.26	0.13	1.02
4825	Ar2	1.24	1.21	0.34	0.2	0.03	0.26	0.13	1.04
4826	Ar2	1.38	1.29	0.23	0.2	0.04	0.28	0.11	0.65
4827	Ar2	1.05	1.18	0.49	0.18	0.04	0.26	0.11	0.84
5205	Ar2	0.38	1.72	0.24	0.26	0.07	0.34	0.15	0.95
4829	Ar2	0.79	1.23	0.57	0.16	0.02	0.22	0.11	1.06
4830	Ar2	1.3	1.14	0.15	0.16	0.03	0.22	0.09	0.73
4831	Ar2	0.92	0.5	0.42	0.17	0.04	0.26	0.09	0.54
4832	Ar2	1.5	1.31	0.07	0.18	0.03	0.24	0.09	0.66
4833	Ar2	1.1	1.36	0.35	0.18	0.02	0.22	0.13	0.59
4834	Ar2	0.98	1.12	0.42	0.16	0.03	0.24	0.11	0.57
4835	Ar2	0.92	1.31	0.29	0.17	0.03	0.24	0.09	0.48
4836	Ar2	0.98	1.1	0.29	0.19	0.02	0.24	0.13	0.57
4837	Ar2	1.33	1.36	0.06	0.17	0.04	0.22	0.09	0.63
4838	Ar2	1.32	1.08	0.1	0.17	0.03	0.19	0.11	0.79
5207	Ar2	0.73	1.44	0.11	0.27	0.05	0.39	0.15	0.97
4840	Ar2	1.09	1.06	0.26	0.17	0.04	0.22	0.06	0.91
4841	Ar2	1.19	1.14	0.12	0.16	0.04	0.22	0.06	0.71
4842	Ar2	0.94	1.1	0.3	0.18	0.02	0.22	0.13	0.53
4843	Ar2	0.79	1.03	0.35	0.17	0.04	0.22	0.09	0.56
4844	Ar2	0.83	1.12	0.27	0.19	0.03	0.24	0.06	0.63
4845	Ar2	0.57	1.42	0.49	0.16	0.03	0.22	0.11	0.86
4846	Ar2	0.72	1.03	0.45	0.18	0.03	0.22	0.11	0.73
4847	Ar2	0.71	1.25	0.37	0.17	0.02	0.24	0.13	0.66
4848	Ar2	1.03	1.4	0.18	0.19	0.04	0.24	0.09	0.57
4849	Ar2	0.92	1.34	0.35	0.21	0.02	0.24	0.15	0.4
4850	Ar2	1.23	1.29	0.21	0.22	0.04	0.28	0.13	0.41
4851	Ar2	0.57	0.88	0.57	0.19	0.03	0.26	0.13	0.76
4852	Ar2	0.8	0.95	0.37	0.21	0.04	0.28	0.11	0.72
4853	Ar2	0.62	0.88	0.44	0.19	0.04	0.24	0.11	0.75
4854	Ar2	1.08	1.16	0.13	0.2	0.03	0.24	0.15	0.67
4855	Ar2	0.73	1.25	0.32	0.19	0.06	0.32	0.09	0.25
4856	Ar2	0.57	1.21	0.47	0.18	0.05	0.26	0.11	0.4
4857	Ar2	0.6	1.06	0.38	0.16	0.05	0.26	0.09	0.72
4858	Ar2	0.97	0.17	0.17	0.15	0.03	0.17	0.11	0.36
5208	Ar2	0.51	1.49	0.17	0.28	0.04	0.34	0.19	1.01

4860	Ar2	0.81	0.13	0.31	0.12	0.02	0.17	0.11	0.32
4861	Ar2	0.73	0.75	0.34	0.12	0.02	0.13	0.06	0.46
4862	Ar2	0.57	1.21	0.33	0.11	0.01	0.13	0.09	0.73
4863	Ar2	1.33	0.37	0.15	0.16	0.02	0.19	0.11	0.42
4864	Ar2	1.19	1.01	0.08	0.13	0.02	0.17	0.09	0.49
4865	Ar2	0.92	0.45	0.28	0.15	0.03	0.22	0.11	0.41
4866	Ar2	0.62	1.08	0.46	0.16	0.02	0.22	0.13	0.38
4867	Ar2	0.65	0.88	0.47	0.15	0.01	0.17	0.11	0.73
5220	Ar2	0.4	1.38	0.19	0.19	0.05	0.24	0.06	0.7
4869	Ar2	1.07	1.06	0.21	0.15	0.02	0.19	0.09	0.6
4870	Ar2	1.75	0.95	0.06	0.14	0.02	0.17	0.06	0.52
4871	Ar2	1.07	1.08	0.3	0.14	0.02	0.19	0.09	0.38
4872	Ar2	1.06	1.01	0.26	0.12	0.03	0.17	0.04	0.6
4873	Ar2	1.07	1.06	0.23	0.1	0.02	0.13	0.06	0.91
4874	Ar2	1.18	1.29	0.18	0.14	0.03	0.19	0.09	0.65
4875	Ar2	0.85	1.14	0.42	0.15	0.02	0.19	0.11	0.63
4876	Ar2	0.93	1.18	0.33	0.15	0.02	0.17	0.11	0.72
4877	Ar2	1.45	0.28	0.08	0.15	0.02	0.22	0.13	0.37
4878	Ar2	0.67	1.16	0.41	0.15	0.02	0.17	0.11	0.59
5224	Ar2	0.4	0.88	0.23	0.22	0.02	0.24	0.17	0.67
4880	Ar2	1	1.16	0.14	0.16	0.02	0.19	0.09	0.53
4881	Ar2	0.78	1.12	0.32	0.16	0.03	0.19	0.09	0.39
4882	Ar2	1.1	1.25	0.12	0.17	0.04	0.24	0.11	0.3
4883	Ar2	0.77	0.19	0.38	0.19	0.05	0.28	0.09	0.41
4884	Ar2	1.17	0.22	0.14	0.19	0.03	0.26	0.11	0.51
5255	Ar2	0.63	0.75	0.26	0.22	0.04	0.28	0.15	0.65
4886	Ar2	0.95	0.97	0.17	0.18	0.05	0.26	0.09	0.28
4887	Ar2	0.53	0.99	0.35	0.17	0.01	0.22	0.13	0.89
4888	Ar2	0.59	1.1	0.29	0.17	0.02	0.19	0.11	0.85
5266	Er	0.44	0.09	0.34	0.27	0.04	0.34	0.17	0.34
4890	Ar2	0.79	1.4	0.17	0.18	0.04	0.24	0.11	0.35
4891	Ar2	0.63	1.01	0.32	0.17	0.04	0.22	0.11	0.44
4892	Ar2	0.59	0.9	0.33	0.19	0.03	0.24	0.13	0.45
4893	Ar2	0.65	0.86	0.28	0.16	0.03	0.24	0.11	0.65
4894	Ar2	0.54	1.06	0.43	0.2	0.03	0.24	0.11	0.43
4895	Ar2	0.66	0.84	0.31	0.18	0.05	0.26	0.06	0.72
4896	Ar2	1.09	1.23	0.13	0.19	0.04	0.26	0.13	0.45
4897	Ar2	0.85	0.19	0.27	0.2	0.04	0.26	0.11	0.46
4898	Ar2	1.04	1.14	0.17	0.21	0.03	0.26	0.15	0.64
4899	Ar2	0.89	0.97	0.28	0.22	0.03	0.28	0.15	0.68
4900	Ar2	0.75	0.67	0.38	0.22	0.04	0.3	0.13	0.79
4901	Ar2	0.69	1.14	0.34	0.21	0.06	0.3	0.11	0.46
4902	Ar2	0.67	1.31	0.34	0.2	0.04	0.26	0.13	0.59
4903	Ar2	0.7	1.01	0.28	0.23	0.04	0.3	0.11	0.41
4904	Ar2	1.24	0.9	0.17	0.19	0.03	0.22	0.11	0.4
4905	Ar2	0.81	0.93	0.35	0.16	0.04	0.22	0.09	0.67
4906	Ar2	1.14	0.88	0.17	0.21	0.03	0.26	0.15	0.54
4907	Ar2	1.14	1.06	0.16	0.18	0.03	0.26	0.13	0.63
4908	Ar2	0.72	0.95	0.4	0.18	0.05	0.3	0.09	0.63
4909	Ar2	0.83	0.95	0.35	0.18	0.05	0.26	0.09	0.57
4910	Ar2	0.89	0.37	0.38	0.23	0.05	0.3	0.11	0.35
4911	Ar2	0.98	0.84	0.29	0.19	0.04	0.26	0.11	0.58
4912	Ar2	0.66	1.08	0.4	0.28	0.05	0.34	0.17	0.77
4913	Ar2	0.53	1.18	0.37	0.26	0.02	0.32	0.24	0.84

4914	Ar2	0.71	1.16	0.29	0.27	0.04	0.3	0.19	0.91
4915	Ar2	0.76	1.16	0.21	0.2	0.05	0.3	0.09	0.85
4916	Ar2	0.74	1.08	0.2	0.23	0.04	0.3	0.13	0.78
5267	Ar2	0.52	0.86	0.22	0.22	0.04	0.3	0.13	0.65
4918	Ar2	0.67	0.75	0.41	0.22	0.05	0.32	0.15	0.82
4919	Ar2	0.53	0.73	0.35	0.23	0.05	0.34	0.11	1.01
4920	Ar2	0.57	1.1	0.36	0.22	0.04	0.3	0.15	0.96
4921	Ar2	0.91	0.54	0.26	0.23	0.05	0.32	0.15	0.64
4922	Ar2	0.85	1.27	0.23	0.2	0.07	0.3	0.13	0.74
4923	Ar2	1.15	1.21	0.27	0.23	0.05	0.3	0.15	0.45
4924	Ar2	0.74	0.69	0.49	0.24	0.06	0.3	0.13	0.75
4925	Ar2	1.03	0.34	0.34	0.31	0.04	0.39	0.17	0.48
4926	Ar2	0.75	0.32	0.65	0.33	0.01	0.34	0.3	0.33
4927	Ar2	0.64	0.5	0.58	0.28	0.04	0.32	0.19	0.87
5276	Ar2	0.42	0.75	0.36	0.24	0.02	0.26	0.19	0.72
4929	Ar2	0.92	1.31	0.25	0.24	0.06	0.32	0.15	0.78
5278	Ar2	0.45	0.26	0.33	0.23	0.04	0.28	0.13	0.32
4931	Ar2	0.95	0.99	0.24	0.23	0.08	0.34	0.09	0.72
4932	Ar2	0.73	0.69	0.55	0.25	0.03	0.37	0.19	0.67
4933	Ar2	0.78	1.21	0.43	0.23	0.05	0.28	0.11	1.03
4934	Ar2	0.86	0.73	0.52	0.22	0.03	0.3	0.15	0.78
4935	Ar2	0.9	0.95	0.48	0.23	0.03	0.28	0.15	0.77
4936	Ar2	0.94	1.03	0.33	0.22	0.04	0.28	0.15	0.81
4937	Ar2	0.83	1.18	0.34	0.2	0.03	0.26	0.17	1.03
4938	Ar2	0.64	0.69	0.42	0.2	0.04	0.28	0.11	1.04
4939	Ar2	0.76	1.21	0.43	0.24	0.04	0.32	0.13	0.65
5279	Ar2	0.77	0.69	0.149955	0.18	0.06	0.28	0.09	0.44
4941	Ar2	0.65	0.8	0.63	0.29	0.04	0.34	0.22	0.8
4942	Ar2	0.7	0.88	0.53	0.35	0.05	0.39	0.13	0.69
4943	Ar2	1.25	1.27	0.3	0.34	0.05	0.41	0.19	0.51
4944	Ar2	1.17	1.23	0.39	0.31	0.05	0.41	0.17	0.39
4945	Ar2	0.8	0.71	0.48	0.27	0.08	0.37	0.13	0.86
4946	Ar2	1.44	0.22	0.08	0.31	0.07	0.41	0.17	0.46
4947	Roar	1.15	1.03	0.22	0.28	0.08	0.41	0.13	0.49
4948	Ar2	0.64	0.24	0.61	0.36	0.04	0.41	0.28	0.41
4949	Ar2	0.59	0.97	0.42	0.33	0.07	0.39	0.15	0.77
4950	Ar2	0.77	0.45	0.39	0.35	0.13	0.5	0.11	0.36
4951	Ar2	1.16	0.17	0.16	0.36	0.14	0.54	0.15	0.34
4952	Ar2	1.03	0.82	0.52	0.39	0.03	0.43	0.26	0.56
4953	Ar2	0.77	0.5	0.66	0.47	0.04	0.5	0.15	0.59
4954	Ar2	1.07	1.1	0.54	0.4	0.04	0.45	0.26	0.88
4955	Ar2	1.11	1.21	0.32	0.35	0.09	0.5	0.19	0.5
4956	Ar2	0.69	0.86	0.6	0.37	0.05	0.43	0.26	0.79
5285	Er	0.43	0.97	0.28	0.21	0.02	0.24	0.17	0.5
4958	Ar2	0.64	0.22	0.63	0.33	0.06	0.43	0.22	1.14
4959	Ar2	1.12	1.36	0.39	0.35	0.06	0.45	0.17	1.04
4960	Ar2	1.06	1.16	0.52	0.32	0.07	0.45	0.15	0.53
4961	Ar2	1.36	1.16	0.31	0.33	0.05	0.39	0.22	0.75
5286	Ar2	0.54	0.88	0.24	0.21	0.03	0.26	0.13	0.57
4963	Ar2	0.97	1.1	0.46	0.33	0.06	0.39	0.11	0.76
4964	Ar2	1.36	1.21	0.25	0.27	0.1	0.43	0.13	0.69
4965	Ar2	1.01	0.99	0.41	0.29	0.09	0.39	0.13	0.86
4966	Ar2	0.67	1.4	0.34	0.3	0.08	0.41	0.17	0.67
4967	Ar2	0.66	0.97	0.4	0.31	0.04	0.39	0.22	1.09

5293	Ar2	0.34	0.71	0.16	0.22	0.02	0.24	0.17	0.57
5296	Ar2	0.14	1.1	0.08	0.21	0.01	0.22	0.19	0.67
4970	Ar2	0.78	1.68	0.17	0.28	0.04	0.32	0.19	1.09
4971	Ar2	0.7	1.27	0.38	0.29	0.04	0.34	0.19	1.06
5298	Er	0.2	0.97	0.11	0.22	0.02	0.26	0.19	0.55
4973	Ar2	0.98	1.29	0.14	0.28	0.06	0.37	0.09	0.87
4974	Ar2	1.2	0.34	0.27	0.32	0.07	0.45	0.15	0.48
4975	Ar2	1.05	1.29	0.32	0.29	0.04	0.34	0.19	0.61
4976	Ar2	1.42	1.36	0.15	0.29	0.04	0.34	0.17	0.65
5299	Er	0.21	0.99	0.07	0.19	0.03	0.28	0.15	0.81
4978	Ar2	1.25	1.36	0.16	0.26	0.04	0.32	0.17	1.18
4979	Ar2	0.78	0.95	0.48	0.26	0.06	0.34	0.15	0.98
4980	Ar2	0.99	0.82	0.64	0.33	0.03	0.39	0.26	0.63
4981	Ar2	0.98	0.9	0.71	0.32	0.03	0.37	0.26	0.76
4982	Ar2	0.99	0.5	0.76	0.33	0.04	0.41	0.24	0.4
4983	Ar2	1.01	0.43	0.78	0.39	0.05	0.45	0.19	0.44
4984	Ar2	1.11	1.23	0.47	0.24	0.07	0.39	0.13	0.27
4985	Ar2	1.74	0.97	0.2	0.28	0.03	0.32	0.22	0.73
4986	Ar2	0.92	0.67	0.51	0.29	0.07	0.43	0.17	0.31
4987	Ar2	0.67	0.67	0.56	0.3	0.09	0.45	0.15	0.38
4988	Er	0.86	0.3	0.31	0.33	0.09	0.5	0.15	0.27
4989	Er	1.47	0.24	0.06	0.33	0.11	0.45	0.11	0.27
4990	Ar2	0.96	0.45	0.31	0.41	0.06	0.52	0.22	0.39
4991	Ar2	1.07	0.45	0.38	0.37	0.07	0.47	0.22	0.34
4992	Ar2	0.95	0.58	0.52	0.37	0.05	0.43	0.28	0.5
4993	Ar2	0.9	0.41	0.69	0.41	0.09	0.52	0.24	0.43
4994	Ar2	0.87	0.45	0.75	0.42	0.11	0.54	0.13	0.38
4995	Er	1.18	0.45	0.57	0.44	0.1	0.6	0.17	0.37
4996	Ar2	1.24	1.18	0.59	0.2	0.02	0.24	0.13	0.56
4997	Ar2	1.45	1.29	0.48	0.2	0.03	0.28	0.15	0.28
4998	Ar2	1.97	0.84	0.14	0.18	0.04	0.24	0.11	0.6
4999	Ar2	1.5	1.06	0.34	0.18	0.04	0.26	0.11	0.27
5000	Ar2	1.29	0.88	0.42	0.19	0.03	0.24	0.11	0.6
5310	Ar2	0.28	0.97	0.19	0.23	0.02	0.26	0.17	0.7
5002	Ar2	0.89	0.73	0.74	0.21	0.04	0.26	0.13	0.56
5317	Ar2	0.24	1.01	0.12	0.23	0.03	0.26	0.17	0.79
5004	Ar2	1.07	0.86	0.5	0.15	0.03	0.22	0.11	0.41
5005	Ar2	1.24	1.08	0.29	0.2	0.02	0.24	0.11	0.34
5006	Ar2	1.02	0.9	0.39	0.16	0.02	0.19	0.11	0.62
5007	Ar2	0.97	0.82	0.55	0.18	0.03	0.24	0.13	0.38
5008	Ar2	1.38	0.95	0.25	0.19	0.03	0.24	0.13	0.41
5009	Ar2	1.72	0.73	0.09	0.22	0.04	0.26	0.11	0.4
5010	Ar2	1.68	1.31	0.09	0.22	0.04	0.3	0.11	0.31
5011	Ar2	1.59	1.03	0.15	0.22	0.04	0.26	0.13	0.33
5012	Ar2	1.07	0.17	0.54	0.2	0.04	0.3	0.13	0.32
5013	Ar2	1.06	0.41	0.61	0.2	0.05	0.26	0.09	0.33
5323	Ar2	0.42	1.16	0.29	0.22	0.02	0.26	0.15	0.76
5015	Ar2	1.15	0.82	0.4	0.2	0.03	0.24	0.11	0.55
5016	Ar2	0.86	0.71	0.59	0.19	0.03	0.22	0.13	0.49
5017	Ar2	0.63	0.75	0.41	0.3	0.03	0.32	0.22	0.49
5018	Ar2	1.16	0.69	0.22	0.3	0.05	0.37	0.19	0.37
5019	Ar2	0.9	0.28	0.29	0.26	0.06	0.41	0.17	0.25
5020	Ar2	1.31	0.88	0.11	0.26	0.04	0.32	0.15	0.43
5021	Ar2	1.08	1.01	0.19	0.25	0.06	0.32	0.13	0.57

5022	Ar2	0.98	0.99	0.25	0.26	0.04	0.3	0.17	0.61
5023	Ar2	0.9	0.34	0.25	0.34	0.06	0.43	0.13	0.44
5326	Ar2	0.36	1.34	0.25	0.24	0.03	0.28	0.17	0.68
5025	Ar2	0.67	0.82	0.34	0.23	0.06	0.37	0.13	0.59
5328	Ar2	0.25	1.03	0.2	0.22	0.03	0.26	0.17	0.71
5027	Ar2	0.89	0.8	0.46	0.24	0.03	0.28	0.19	0.65
5028	Ar2	0.83	0.65	0.45	0.21	0.06	0.3	0.13	0.55
5029	Ar2	0.83	0.67	0.42	0.22	0.04	0.28	0.15	0.63
5030	Ar2	1.36	0.26	0.38	0.26	0.05	0.34	0.13	0.3
5031	Ar2	1.76	0.32	0.07	0.24	0.05	0.3	0.11	0.52
5032	Ar2	0.77	0.24	0.6	0.25	0.05	0.3	0.13	0.43
5033	Ar2	0.98	0.88	0.4	0.24	0.05	0.3	0.13	0.44
5329	Ar2	0.25	1.12	0.21	0.23	0.03	0.28	0.19	0.63
5035	Ar2	0.88	1.06	0.33	0.21	0.07	0.3	0.11	0.43
5036	Ar2	0.85	0.22	0.52	0.23	0.03	0.3	0.17	0.33
5037	Ar2	0.88	0.24	0.55	0.23	0.06	0.3	0.13	0.24
5038	Ar2	0.98	0.9	0.4	0.23	0.04	0.28	0.11	0.63
5330	Ar2	0.25	1.27	0.2	0.23	0.02	0.26	0.19	0.67
48	Ar1	1.02	2.17	0.38	0.41	0.05	0.47	0.24	2.09
5041	Er	0.65	0.28	0.57	0.31	0.05	0.41	0.24	0.26
5042	Ar2	0.59	0.34	0.44	0.29	0.07	0.39	0.15	0.31
5043	Ar2	0.75	0.47	0.45	0.31	0.04	0.37	0.17	0.43
5044	Ar2	0.99	0.28	0.67	0.25	0.05	0.32	0.15	0.24
5045	Ar2	1.02	0.22	0.54	0.23	0.06	0.32	0.13	0.28
5046	Ar2	0.75	0.45	0.64	0.28	0.09	0.41	0.09	0.25
5047	Ar2	1.45	0.22	0.2	0.27	0.05	0.37	0.19	0.25
179	Ar1	0.77	2.37	0.38	0.32	0.03	0.41	0.26	1.53
202	Ar1	0.57	2.3	0.18	0.33	0.04	0.41	0.26	1.77
5050	Ar2	1.43	1.21	0.11	0.21	0.04	0.3	0.11	0.25
5051	Ar2	1.28	0.22	0.28	0.22	0.03	0.28	0.15	0.27
5052	Ar2	1.61	0.15	0.09	0.21	0.03	0.3	0.15	0.23
5053	Ar2	1.15	0.32	0.28	0.23	0.04	0.28	0.15	0.3
5054	Ar2	1.22	0.47	0.2	0.18	0.05	0.28	0.11	0.35
5055	Ar2	1.11	0.22	0.48	0.21	0.05	0.32	0.11	0.2
5056	Ar2	0.82	0.26	0.52	0.24	0.04	0.3	0.13	0.24
5057	Ar2	1.05	0.45	0.45	0.23	0.03	0.28	0.15	0.33
5058	Ar2	1.12	0.26	0.45	0.24	0.02	0.3	0.17	0.22
5059	Ar2	0.83	0.22	0.68	0.23	0.04	0.28	0.15	0.24
5060	Ar2	1.2	0.43	0.45	0.21	0.04	0.28	0.15	0.32
5061	Ar2	0.94	0.19	0.56	0.24	0.06	0.32	0.13	0.25
5062	Ar2	1.61	0.82	0.2	0.22	0.06	0.28	0.11	0.42
5063	Ar2	1.63	0.67	0.14	0.28	0.04	0.32	0.11	0.58
5064	Ar2	0.57	0.9	0.25	0.23	0.09	0.41	0.13	0.62
5065	Ar2	0.8	0.95	0.15	0.29	0.05	0.41	0.19	0.59
5066	Ar2	0.5	1.16	0.19	0.33	0.02	0.34	0.26	0.89
476	Ar1	0.42	2.09	0.26	0.25	0.05	0.34	0.13	1.32
5068	Roar	0.54	0.82	0.22	0.33	0.07	0.43	0.17	0.54
5069	Ar2	0.67	1.51	0.09	0.34	0.08	0.5	0.19	0.41
497	Ar1	0.48	2.22	0.34	0.29	0.03	0.34	0.24	1.8
5071	Ar2	0.71	1.27	0.07	0.29	0.09	0.5	0.15	0.45
5072	Ar2	0.5	1.55	0.24	0.33	0.06	0.41	0.19	0.75
5073	Ar2	0.45	1.27	0.19	0.31	0.06	0.43	0.13	0.84
5074	Ar2	0.48	1.23	0.26	0.29	0.07	0.39	0.17	0.61
5075	Ar2	0.76	0.88	0.16	0.44	0.04	0.5	0.32	0.79

5076	Ar2	0.67	0.93	0.26	0.44	0.09	0.52	0.19	0.69
5077	Ar2	0.59	0.9	0.22	0.31	0.07	0.45	0.24	0.73
498	Ar1	0.54	2.43	0.17	0.29	0.02	0.32	0.26	1.71
5079	Ar2	0.55	0.88	0.27	0.4	0.07	0.47	0.24	0.71
5080	Ar2	0.41	0.45	0.22	0.39	0.07	0.47	0.26	0.47
5081	Ar2	0.4	0.69	0.3	0.38	0.06	0.5	0.3	0.63
500	Ar1	0.6	2.22	0.42	0.29	0.03	0.37	0.24	1.79
532	Ar1	0.32	1.77	0.11	0.29	0.02	0.32	0.26	1.45
5084	Ar2	0.48	0.5	0.28	0.41	0.09	0.5	0.26	0.54
5085	Ar2	0.74	1.31	0.07	0.36	0.08	0.5	0.24	0.62
560	Ar1	0.46	1.72	0.36	0.27	0.03	0.37	0.22	1.41
5087	Ar2	0.31	1.25	0.17	0.36	0.12	0.5	0.09	0.64
5088	Ar2	0.66	0.97	0.42	0.39	0.03	0.43	0.3	0.76
5089	Ar2	0.59	0.84	0.43	0.42	0.03	0.45	0.3	1.01
5090	Ar2	0.55	1.1	0.4	0.36	0.04	0.45	0.28	0.79
5091	Ar2	0.63	1.25	0.3	0.4	0.04	0.52	0.32	0.8
5092	Ar2	0.87	1.06	0.13	0.34	0.11	0.5	0.19	0.57
5093	Ar2	0.46	1.27	0.27	0.33	0.08	0.47	0.22	0.88
5094	Ar2	0.54	0.45	0.35	0.38	0.08	0.5	0.22	0.44
580	Ar1	0.82	2.26	0.61	0.3	0.03	0.37	0.22	1.6
5096	Ar2	0.66	0.41	0.16	0.39	0.06	0.47	0.24	0.34
694	Ar1	0.73	2.37	0.18	0.31	0.02	0.37	0.24	2.03
699	Ar1	0.69	2.22	0.4	0.32	0.02	0.37	0.28	2.13
5099	Ar2	0.48	0.28	0.37	0.31	0.05	0.39	0.24	0.35
5100	Ar2	0.69	0.37	0.43	0.35	0.05	0.41	0.22	0.43
5101	Ar2	0.32	0.97	0.22	0.34	0.01	0.37	0.32	0.33
5102	Ar2	0.35	0.34	0.21	0.3	0.07	0.41	0.17	0.34
5103	Ar2	0.44	0.28	0.11	0.3	0.05	0.37	0.22	0.28
5104	Ar2	0.74	0.34	0.22	0.36	0.04	0.41	0.28	0.35
5105	Ar2	0.43	0.39	0.31	0.34	0.02	0.39	0.32	0.35
5106	Ar2	0.22	0.28	0.08	0.26	0.03	0.34	0.22	0.28
708	Ar1	0.53	2.07	0.2	0.28	0.04	0.34	0.19	1.37
5108	Ar2	0.1	0.82	0.05	0.27	0.02	0.3	0.26	0.6
998	Ar1	0.92	2.41	0.42	0.56	0.1	0.65	0.24	2.54
5110	Ar2	0.14	0.52	0.09	0.29	0.03	0.37	0.26	0.36
1001	Ar1	0.82	3.47	0.57	0.56	0.1	0.69	0.37	2.27
5112	Ar2	0.28	0.28	0.07	0.29	0.04	0.32	0.19	0.66
1046	Ar1	0.97	3.36	0.81	0.56	0.1	0.65	0.26	2.21
5114	Er	0.07	0.9	0.05	0.31	0.02	0.34	0.28	0.83
1059	Ar1	1.02	1.83	0.54	0.55	0.11	0.62	0.17	2.14
1060	Ar1	0.88	1.96	0.6	0.53	0.1	0.62	0.19	2.09
5117	Er	0.26	0.8	0.13	0.3	0.04	0.39	0.26	0.53
1061	Ar1	0.75	2.11	0.57	0.51	0.11	0.6	0.22	2.11
5119	Ar2	0.28	0.97	0.12	0.26	0.07	0.39	0.17	0.75
5120	Ar2	0.27	0.88	0.16	0.35	0.05	0.43	0.19	0.76
5121	Ar2	0.24	0.99	0.19	0.34	0.01	0.37	0.32	0.78
5122	Ar2	0.63	1.08	0.06	0.35	0.05	0.43	0.26	0.37
5123	Ar2	0.56	1.08	0.03	0.32	0.06	0.43	0.17	0.38
5124	Ar2	0.29	0.45	0.27	0.51	0.04	0.56	0.43	0.49
5125	Ar2	0.19	0.34	0.17	0.32	0.07	0.45	0.24	0.41
5126	Ar2	0.22	0.39	0.18	0.39	0.06	0.45	0.26	0.54
5127	Ar2	0.13	0.69	0.07	0.39	0.09	0.47	0.15	0.66
1062	Ar1	1.04	1.83	0.54	0.55	0.11	0.62	0.22	2.13
5129	Ar2	0.14	0.45	0.06	0.39	0.1	0.5	0.17	0.47

1063	Ar1	0.83	2	0.6	0.56	0.1	0.65	0.22	2.04
5131	Er	0.18	0.45	0.07	0.43	0.03	0.45	0.37	0.59
1064	Ar1	0.74	2.24	0.58	0.53	0.12	0.65	0.11	2.32
5133	Ar2	0.27	0.84	0.16	0.42	0.02	0.5	0.41	0.85
5134	Chuff	0.06	1.16	0.03	0.39	0	0.39	0.39	1.13
5135	Ar2	0.2	0.41	0.07	0.37	0.07	0.47	0.24	0.6
1073	Ar1	0.87	3.57	0.57	0.57	0.09	0.69	0.24	2.19
5137	Ar2	0.22	1.16	0.17	0.37	0.04	0.45	0.3	0.68
5138	Ar2	0.38	0.45	0.2	0.39	0.07	0.47	0.26	0.46
5139	Ar2	0.33	1.16	0.07	0.36	0.03	0.43	0.28	0.81
1074	Ar1	0.85	3.49	0.52	0.62	0.12	0.71	0.19	2.27
1075	Ar1	0.72	1.96	0.63	0.56	0.09	0.65	0.37	2.24
1078	Ar1	0.79	1.85	0.55	0.55	0.11	0.65	0.26	2.22
1082	Ar1	0.69	1.96	0.56	0.57	0.07	0.71	0.39	2.1
5144	Ar2	0.22	0.26	0.15	0.24	0.03	0.28	0.19	0.27
5145	Er	0.13	1.23	0.03	0.25	0.02	0.28	0.24	1.04
1083	Ar1	0.64	2.26	0.43	0.53	0.12	0.67	0.24	2.39
5147	Ar2	0.28	1.4	0.03	0.25	0.07	0.37	0.13	0.89
5148	Ar2	0.35	1.23	0.17	0.23	0.06	0.3	0.15	0.43
1087	Ar1	0.45	2.17	0.29	0.5	0.11	0.58	0.24	2.21
5150	Ar2	0.07	0.37	0.26	0.25	0.05	0.3	0.15	1.04
5151	Ar2	0.25	0.28	0.21	0.29	0.06	0.37	0.15	0.38
5152	Ar2	0.44	1.42	0.08	0.28	0.05	0.34	0.15	0.48
5153	Ar2	0.37	1.46	0.13	0.26	0.03	0.34	0.22	0.43
5154	Ar2	0.31	1.01	0.14	0.24	0.07	0.43	0.13	0.71
5155	Ar2	0.25	1.06	0.11	0.27	0.07	0.32	0.15	0.88
5156	Ar2	0.4	1.14	0.19	0.27	0.05	0.34	0.19	0.52
5157	Ar2	0.42	1.03	0.15	0.25	0.04	0.34	0.17	0.8
5158	Ar2	0.22	1.01	0.16	0.22	0.07	0.32	0.13	0.9
1094	Ar1	0.63	1.77	0.45	0.53	0.13	0.67	0.19	2.04
5160	Ar2	0.22	0.26	0.19	0.3	0.05	0.37	0.19	0.4
5161	Ar2	0.47	0.24	0.08	0.29	0.05	0.39	0.19	0.5
5162	Ar2	0.34	0.28	0.28	0.3	0.05	0.39	0.19	0.31
1095	Ar1	0.86	1.85	0.42	0.54	0.1	0.65	0.24	2.03
1099	Ar1	0.82	2.11	0.4	0.52	0.11	0.65	0.17	2.02
5165	Ar2	0.34	1.01	0.12	0.24	0.05	0.34	0.13	0.58
5166	Ar2	0.38	0.95	0.26	0.26	0.07	0.34	0.11	0.57
5167	Ar2	0.38	0.93	0.16	0.22	0.07	0.37	0.13	0.68
5168	Ar2	0.39	0.97	0.27	0.28	0.05	0.37	0.17	0.59
1100	Ar1	0.78	2.13	0.24	0.61	0.14	0.8	0.28	2.27
1105	Ar1	0.71	3.75	0.37	0.55	0.1	0.62	0.26	2.09
5171	Roar	0.44	0.73	0.09	0.25	0.1	0.37	0.09	0.34
5172	Roar	0.43	0.56	0.2	0.25	0.06	0.32	0.17	0.62
5173	Roar	0.47	0.86	0.14	0.22	0.08	0.39	0.13	0.39
5174	Ar2	0.61	1.36	0.07	0.21	0.03	0.26	0.13	0.72
5175	Ar2	0.53	0.67	0.18	0.21	0.04	0.28	0.13	0.79
5176	Roar	0.48	1.36	0.19	0.21	0.04	0.32	0.15	0.46
5177	Ar2	0.61	1.51	0.15	0.23	0.06	0.34	0.15	0.36
1160	Ar1	0.54	2.2	0.17	0.42	0.09	0.54	0.24	2.06
5179	Ar2	0.6	1.25	0.13	0.24	0.03	0.28	0.15	1.01
1191	Ar1	0.58	2.35	0.19	0.31	0.03	0.41	0.26	1.81
5181	Ar2	0.5	1.21	0.21	0.19	0.03	0.24	0.13	1.17
1209	Ar1	0.67	2.43	0.26	0.35	0.04	0.41	0.26	2
1218	Ar1	0.67	3.19	0.41	0.46	0.07	0.52	0.24	2.02

5184	Ar2	0.75	1.25	0.13	0.26	0.08	0.43	0.13	0.37
1219	Ar1	0.48	3.23	0.44	0.38	0.13	0.5	0.09	1.91
5186	Ar2	0.68	1.49	0.18	0.27	0.06	0.41	0.15	0.4
5187	Ar2	0.57	1.08	0.22	0.25	0.07	0.37	0.13	0.55
5188	Ar2	0.54	1.44	0.24	0.25	0.05	0.3	0.17	0.86
5189	Ar2	0.39	1.29	0.28	0.22	0.03	0.28	0.17	1.18
1225	Ar1	0.61	2.24	0.35	0.43	0.12	0.58	0.06	1.93
5191	Ar2	0.58	1.25	0.12	0.25	0.04	0.32	0.15	0.99
5192	Ar2	0.41	1.44	0.17	0.26	0.04	0.3	0.17	0.85
5193	Ar2	0.62	1.36	0.1	0.27	0.03	0.3	0.19	1.01
1235	Ar1	0.48	2.22	0.09	0.48	0.06	0.56	0.32	2.15
1237	Ar1	0.57	2.07	0.33	0.45	0.09	0.54	0.24	1.86
1255	Ar1	0.49	2.35	0.19	0.39	0.09	0.52	0.22	2.05
5197	Ar2	0.34	1.87	0.16	0.3	0.04	0.34	0.22	0.66
5198	Ar2	0.43	1.25	0.13	0.3	0.02	0.34	0.26	0.68
5199	Ar2	0.47	1.42	0.12	0.27	0.06	0.34	0.15	0.72
5200	Ar2	0.68	1.42	0.12	0.29	0.06	0.41	0.13	0.61
5201	Ar2	0.5	1.4	0.28	0.29	0.06	0.37	0.17	0.99
5202	Ar2	0.58	1.79	0.24	0.3	0.03	0.37	0.22	1.25
5203	Ar2	0.41	1.55	0.22	0.35	0.05	0.41	0.24	0.84
5204	Ar2	0.39	1.44	0.17	0.29	0.04	0.37	0.22	1.18
1256	Ar1	0.46	3.08	0.03	0.32	0.02	0.37	0.3	2.19
5206	Ar2	0.67	1.36	0.13	0.3	0.04	0.37	0.24	0.82
1287	Ar1	0.8	1.83	0.56	0.52	0.08	0.62	0.32	2.02
1293	Ar1	0.98	2.22	0.71	0.5	0.08	0.6	0.22	2.03
5209	Ar2	0.39	1.01	0.31	0.15	0.03	0.22	0.13	0.84
5210	Ar2	0.55	1.03	0.19	0.16	0.02	0.19	0.13	0.82
5211	Ar2	0.51	1.08	0.22	0.19	0.03	0.24	0.11	0.68
5212	Ar2	0.58	1.08	0.18	0.18	0.03	0.22	0.09	0.82
5213	Ar2	0.41	0.45	0.36	0.16	0.02	0.22	0.13	0.58
5214	Ar2	0.53	0.99	0.28	0.15	0.02	0.19	0.09	0.84
5215	Ar2	0.93	0.32	0.11	0.22	0.03	0.26	0.13	0.3
5216	Ar2	0.58	1.08	0.17	0.17	0.04	0.24	0.11	0.34
5217	Ar2	0.55	1.03	0.2	0.16	0.04	0.22	0.11	0.48
5218	Ar2	0.41	1.18	0.22	0.15	0.03	0.24	0.09	0.84
5219	Ar2	0.47	1.18	0.18	0.17	0.02	0.22	0.13	0.95
1307	Ar1	1.19	2.43	0.48	0.44	0.07	0.54	0.22	2.13
5221	Ar2	0.47	1.14	0.19	0.19	0.04	0.22	0.09	0.87
5222	Ar2	0.5	0.86	0.23	0.2	0.03	0.26	0.17	0.44
5223	Ar2	0.35	0.99	0.19	0.2	0.04	0.26	0.11	0.61
1310	Ar1	0.81	2.2	0.55	0.46	0.09	0.58	0.22	2.13
5225	Ar2	0.46	1.12	0.12	0.19	0.03	0.22	0.13	0.86
5226	Ar2	0.49	1.12	0.21	0.18	0.02	0.22	0.15	0.77
5227	Ar2	0.39	1.1	0.21	0.18	0.02	0.22	0.13	0.97
5228	Ar2	0.52	1.14	0.13	0.18	0.02	0.22	0.13	0.83
5229	Ar2	0.45	1.1	0.2	0.17	0.04	0.24	0.11	0.69
5230	Ar2	0.31	0.99	0.23	0.16	0.02	0.19	0.13	0.9
5231	Ar2	0.37	1.12	0.2	0.16	0.04	0.26	0.11	0.51
5232	Ar2	0.54	1.14	0.16	0.18	0.04	0.24	0.11	0.47
5233	Ar2	0.49	1.01	0.16	0.17	0.02	0.22	0.13	0.84
5234	Ar2	0.88	1.57	0.19	0.2	0.04	0.26	0.11	0.36
5235	Ar2	0.68	1.51	0.22	0.2	0.02	0.24	0.13	0.59
5236	Ar2	0.76	1.34	0.15	0.18	0.04	0.24	0.09	0.82
5237	Ar2	0.73	1.23	0.26	0.17	0.02	0.22	0.11	0.83

5238	Ar2	0.7	1.25	0.28	0.15	0.03	0.22	0.11	0.92
5239	Ar2	0.56	1.12	0.34	0.18	0.03	0.24	0.09	0.87
5240	Ar2	0.52	1.34	0.38	0.17	0.02	0.22	0.13	0.65
5241	Ar2	0.71	0.84	0.22	0.18	0.04	0.24	0.11	0.59
5242	Ar2	0.45	1.01	0.33	0.17	0.02	0.19	0.11	0.93
5243	Ar2	0.63	1.14	0.22	0.18	0.03	0.24	0.13	0.7
5244	Ar2	0.58	0.95	0.26	0.2	0.04	0.28	0.11	0.51
5245	Ar2	0.9	1.25	0.08	0.2	0.04	0.26	0.09	0.67
5246	Ar2	0.5	0.9	0.39	0.19	0.03	0.24	0.13	0.7
5247	Ar2	0.62	1.14	0.29	0.2	0.03	0.24	0.13	0.67
5248	Ar2	0.65	1.21	0.23	0.17	0.03	0.24	0.13	0.74
5249	Ar2	0.63	1.1	0.35	0.2	0.03	0.28	0.11	0.75
5250	Ar2	0.7	1.23	0.22	0.18	0.03	0.26	0.13	0.85
5251	Ar2	0.76	1.18	0.22	0.2	0.03	0.26	0.13	0.86
5252	Ar2	0.61	1.18	0.21	0.17	0.02	0.22	0.13	1.11
5253	Ar2	0.56	1.14	0.28	0.2	0.04	0.26	0.11	0.72
5254	Ar2	0.76	1.34	0.16	0.19	0.03	0.28	0.13	0.69
1318	Ar1	0.9	1.96	0.73	0.5	0.05	0.56	0.37	2.03
5256	Ar2	0.74	1.14	0.17	0.19	0.04	0.26	0.13	0.91
5257	Ar2	0.52	1.14	0.24	0.22	0.04	0.26	0.13	0.92
5258	Ar2	0.59	1.16	0.18	0.2	0.04	0.26	0.11	0.78
5259	Ar2	0.37	0.95	0.31	0.2	0.03	0.24	0.13	1.08
5260	Ar2	1.06	0.15	0.11	0.26	0.06	0.34	0.11	0.28
5261	Ar2	0.93	0.3	0.17	0.25	0.04	0.37	0.17	0.27
5262	Ar2	0.61	0.3	0.25	0.23	0.06	0.34	0.13	0.27
5263	Ar2	0.5	0.9	0.3	0.25	0.04	0.28	0.15	0.59
5264	Ar2	0.64	0.9	0.23	0.2	0.06	0.3	0.13	0.46
5265	Ar2	0.68	0.84	0.19	0.23	0.07	0.32	0.09	0.5
1320	Ar1	0.96	2.09	0.64	0.57	0.1	0.69	0.24	2
1321	Ar1	0.92	1.87	0.51	0.58	0.09	0.67	0.3	1.94
5268	Ar2	0.69	1.1	0.16	0.23	0.03	0.28	0.15	0.39
5269	Ar2	0.57	0.95	0.11	0.2	0.04	0.26	0.15	0.8
5270	Ar2	0.4	0.73	0.28	0.25	0.02	0.26	0.22	0.42
5271	Roar	0.35	0.86	0.2	0.2	0.06	0.34	0.09	0.35
5272	Roar	0.55	0.99	0.12	0.19	0.05	0.28	0.06	0.79
5273	Ar2	0.52	0.95	0.19	0.18	0.04	0.24	0.13	0.79
5274	Ar2	0.45	0.88	0.23	0.19	0.04	0.26	0.13	0.75
5275	Roar	0.56	0.86	0.16	0.2	0.04	0.28	0.13	0.65
1325	Ar1	0.89	2.15	0.39	0.51	0.12	0.65	0.17	2
5277	Ar2	0.58	1.06	0.19	0.21	0.05	0.3	0.13	0.45
1326	Ar1	1.11	1.85	0.35	0.56	0.08	0.62	0.24	2.1
1336	Ar1	0.86	1.94	0.3	0.6	0.09	0.69	0.39	2.06
5280	Ar2	0.77	0.86	0.18	0.21	0.04	0.28	0.15	0.42
5281	Ar2	0.56	1.12	0.2	0.18	0.02	0.22	0.13	0.8
5282	Ar2	0.61	0.95	0.25	0.17	0.03	0.24	0.13	0.54
5283	Ar2	0.48	1.01	0.31	0.18	0.02	0.24	0.15	0.62
5284	Roar	0.65	0.99	0.12	0.19	0.05	0.3	0.09	0.37
1349	Ar1	0.74	1.85	0.51	0.61	0.07	0.69	0.39	1.9
1351	Ar1	0.95	1.98	0.18	0.58	0.11	0.67	0.22	2.03
5287	Ar2	0.48	0.19	0.06	0.21	0.04	0.28	0.13	0.29
5288	Ar2	0.35	0.15	0.11	0.2	0.06	0.28	0.11	0.25
5289	Haer	0.45	0.26	0.1	0.23	0.05	0.32	0.09	0.27
5290	Haer	0.23	1.06	0.11	0.17	0.04	0.22	0.11	0.52
5291	Roar	0.29	0.86	0.1	0.18	0.03	0.24	0.13	0.52

5292	Roar	0.26	1.03	0.07	0.21	0.03	0.24	0.13	0.79
1356	Ar1	0.53	1.87	0.32	0.49	0.14	0.67	0.19	2
5294	Ar2	0.29	0.99	0.13	0.19	0.04	0.28	0.11	0.54
5295	Ar2	0.11	1.08	0.05	0.18	0.06	0.28	0.13	0.31
1359	Ar1	0.78	2.2	0.39	0.56	0.11	0.67	0.24	2.19
5297	Ar2	0.28	1.1	0.04	0.21	0.03	0.28	0.15	0.33
1369	Ar1	0.8	2.13	0.55	0.52	0.12	0.65	0.24	1.96
1371	Ar1	0.65	2.24	0.52	0.5	0.12	0.65	0.22	2.11
5300	Roar	0.23	0.8	0.07	0.18	0.06	0.3	0.09	0.45
5301	Ar2	0.21	0.95	0.08	0.18	0.04	0.26	0.11	0.7
5302	Ar2	0.37	1.12	0.05	0.2	0.03	0.26	0.15	0.51
5303	Ar2	0.58	0.26	0.07	0.26	0.03	0.3	0.15	0.36
5304	Ar2	0.49	0.19	0.2	0.19	0.04	0.32	0.15	0.28
5305	Ar2	0.42	0.97	0.18	0.22	0.05	0.3	0.11	0.28
5306	Ar2	0.29	1.25	0.16	0.2	0.04	0.26	0.15	0.37
5307	Ar2	0.36	0.84	0.15	0.2	0.04	0.26	0.11	0.56
5308	Ar2	0.33	0.9	0.07	0.19	0.05	0.26	0.13	0.62
5309	Roar	0.3	0.86	0.08	0.16	0.05	0.34	0.11	0.64
1473	Ar1	0.91	1.89	0.68	0.34	0.07	0.5	0.19	2.01
5311	Roar	0.39	0.78	0.12	0.2	0.04	0.3	0.13	0.58
5312	Ar2	0.48	0.26	0.15	0.23	0.05	0.37	0.15	0.29
5313	Ar2	0.43	0.8	0.21	0.21	0.05	0.28	0.13	0.62
5314	Ar2	0.57	0.8	0.11	0.22	0.05	0.32	0.13	0.5
5315	Ar2	0.34	0.75	0.19	0.22	0.07	0.3	0.09	0.46
5316	Roar	0.28	0.78	0.22	0.22	0.05	0.3	0.15	0.62
1482	Ar1	1.11	1.96	0.55	0.32	0.04	0.43	0.17	1.93
5318	Ar2	0.35	0.9	0.1	0.21	0.05	0.3	0.13	0.52
5319	Roar	0.38	0.88	0.1	0.22	0.07	0.34	0.11	0.5
5320	Ar2	0.36	0.84	0.19	0.24	0.05	0.28	0.15	0.62
5321	Ar2	0.42	0.99	0.14	0.22	0.03	0.3	0.17	0.76
5322	Ar2	0.5	1.01	0.23	0.18	0.03	0.26	0.15	0.72
1494	Ar1	1.49	2.35	0.92	0.4	0.05	0.5	0.24	1.97
5324	Ar2	0.55	1.08	0.17	0.2	0.03	0.24	0.15	0.92
5325	Ar2	0.61	1.27	0.1	0.24	0.03	0.26	0.15	0.81
1686	Ar1	1.18	2.43	0.87	0.45	0.06	0.54	0.24	2.27
5327	Ar2	0.1	0.13	0.39	0.2	0.06	0.28	0.11	0.93
1716	Ar1	1.16	2.05	0.16	0.43	0.06	0.52	0.22	2.04
3736	Er	0.14	0.22	0.12	0.19	0.03	0.22	0.13	0.52
4274	Ar2	1.05	1.66	0.76	0.31	0.02	0.34	0.22	1.36
5331	Ar2	0.31	1.21	0.15	0.23	0.02	0.28	0.19	0.64
5332	Ar2	0.34	0.97	0.14	0.19	0.05	0.26	0.13	0.74
5333	Ar2	0.11	0.15	0.36	0.22	0.04	0.28	0.13	0.75
5334	Ar2	0.36	0.9	0.12	0.23	0.03	0.28	0.15	0.58
5335	Ar2	0.33	0.84	0.17	0.22	0.05	0.3	0.13	0.44

Freq50	Freq75	IQRBW	PulseNum	PulseRate	context
2.04	2.25	0.41	0	0	IS
2.07	2.27	0.44	0	0	IS
2.03	2.21	0.6	0	0	IS
1.83	2.27	0.65	0	0	IS
2.12	2.47	0.54	0	0	IS
2.03	2.26	0.41	0	0	IS
2	2.22	0.51	0	0	IS
1.82	2.21	0.71	0	0	IS
1.99	2.39	0.74	0	0	IS
2.08	2.37	0.48	0	0	IS
2.01	2.27	0.46	0	0	IS
1.96	2.31	0.84	0	0	IS
1.91	2.56	0.95	0	0	IS
1.65	2.4	1.19	0	0	IS
2.03	2.41	0.61	0	0	IS
2.13	2.52	0.57	0	0	IS
2.15	2.53	0.57	0	0	IS
2.17	2.45	0.46	0	0	IS
1.88	2.24	0.86	0	0	IS
2.12	2.33	0.5	0	0	IS
1.9	2.23	0.48	0	0	IS
2.11	2.35	0.5	0	0	IS
2.01	2.34	0.6	0	0	IS
1.97	2.2	0.58	0	0	IS
2.13	2.52	0.6	0	0	IS
2.13	2.44	0.68	0	0	IS
1.94	2.5	0.83	0	0	IS
2	2.48	0.71	0	0	IS
1.94	2.45	0.76	0	0	IS
1.93	2.22	0.57	0	0	IS
1.78	2.29	0.85	0	0	IS
1.82	2.14	0.73	0	0	IS
1.94	2.24	0.49	0	0	IS
1.79	2.26	0.78	0	0	IS
1.67	2.15	0.85	0	0	IS
1.82	2.2	0.61	0	0	IS
1.96	2.19	0.48	0	0	IS
1.93	2.59	1.09	0	0	IS
2.31	2.65	0.54	0	0	IS
2.13	2.64	0.85	0	0	IS
2.17	2.55	0.66	0	0	IS
2.1	2.41	0.57	0	0	IS
2.11	2.59	0.68	0	0	IS
2.11	2.77	1.03	0	0	IS
1.91	2.52	0.83	0	0	IS
2.06	2.84	1.09	0	0	IS
2.16	2.83	1.05	0	0	IS
2.09	2.58	0.8	0	0	IS
1.94	2.17	0.41	0	0	IS
1.99	2.14	0.34	0	0	IS
2.06	2.32	0.59	0	0	IS
2.03	2.57	0.89	0	0	IS
1.95	2.23	0.8	0	0	IS

2.28	2.59	0.48	0	0	IS
2	2.62	0.93	0	0	IS
1.96	2.41	0.82	0	0	IS
2.1	2.52	0.8	0	0	IS
1.96	2.31	0.64	0	0	IS
2.06	2.45	0.5	0	0	IS
2	2.28	0.53	0	0	IS
1.61	2.44	1.04	0	0	IS
1.84	2.36	0.76	0	0	IS
1.88	2.24	0.72	0	0	IS
1.84	2.07	0.34	0	0	IS
2.05	2.29	0.39	0	0	IS
2.1	2.26	0.31	0	0	IS
1.77	2.08	0.79	0	0	IS
1.75	2.23	0.82	0	0	IS
1.57	2.17	0.84	0	0	IS
2.02	2.34	0.64	0	0	IS
1.84	2.11	0.57	0	0	IS
1.88	2.06	0.48	0	0	IS
1.46	1.72	0.43	0	0	IS
1.65	1.85	0.37	0	0	IS
1.7	1.88	0.45	0	0	IS
1.64	2.1	0.86	0	0	IS
1.55	1.91	0.57	0	0	IS
1.75	1.91	0.39	0	0	IS
1.55	1.75	0.42	0	0	IS
1.71	1.91	0.38	0	0	IS
1.59	1.77	0.43	0	0	IS
1.69	1.93	0.35	0	0	IS
1.44	1.76	0.53	0	0	IS
1.54	1.84	0.47	0	0	IS
1.72	1.92	0.38	0	0	IS
1.62	1.98	0.54	0	0	IS
1.58	2.03	0.56	0	0	IS
1.69	2.21	0.93	0	0	IS
1.77	1.95	0.36	0	0	IS
1.59	1.92	0.53	0	0	IS
1.89	2.45	1.27	0	0	IS
1.5	1.78	0.64	0	0	IS
1.64	1.84	0.43	0	0	IS
1.9	2.05	0.38	0	0	IS
2.03	2.24	0.4	0	0	IS
1.91	2.28	0.59	0	0	IS
1.89	2.14	0.45	0	0	IS
1.96	2.19	0.62	0	0	IS
1.76	1.99	0.44	0	0	IS
1.82	2.06	0.42	0	0	IS
1.45	1.6	0.29	0	0	IS
1.85	2.03	0.31	0	0	IS
1.78	2	0.41	0	0	IS
1.74	1.98	0.49	0	0	IS
1.79	2.02	0.48	0	0	IS
1.88	2.16	0.57	0	0	IS
2.14	2.4	0.42	0	0	IS

1.97	2.16	0.47	0	0	IS
1.59	1.93	0.63	0	0	IS
1.59	2.21	1.08	0	0	IS
1.63	1.97	0.99	0	0	IS
1.77	2.03	0.69	0	0	IS
1.66	2.18	0.84	0	0	IS
1.62	1.97	0.82	0	0	IS
1.08	1.49	0.84	0	0	IS
1.49	1.79	0.62	0	0	IS
1.4	1.65	0.68	0	0	IS
1.46	1.91	0.96	0	0	IS
1.58	1.85	0.71	0	0	IS
1.82	2.05	0.5	0	0	IS
1.67	1.98	0.6	0	0	IS
1.76	2.03	0.46	0	0	IS
1.19	1.68	0.73	0	0	IS
1.58	1.95	0.55	0	0	IS
1.29	1.59	0.47	0	0	IS
1.33	1.54	0.4	0	0	IS
1.52	1.73	0.43	0	0	IS
1.49	1.7	0.46	0	0	IS
1.21	1.69	0.67	0	0	IS
1.47	1.65	0.38	0	0	IS
0.93	1.51	1.01	0	0	PL
1.46	1.81	0.55	0	0	IS
1.59	1.76	0.45	0	0	IS
1.49	1.69	0.43	0	0	IS
1.32	1.6	0.58	0	0	IS
1.57	1.83	0.47	0	0	IS
1.42	1.7	0.5	0	0	IS
1.34	1.61	0.51	0	0	IS
1.23	1.68	0.76	0	0	IS
1.52	1.81	0.49	0	0	IS
1.2	1.79	0.92	0	0	IS
1.25	1.73	0.8	0	0	IS
1.39	1.71	0.92	0	0	IS
0.98	1.8	1.2	0	0	IS
0.99	1.48	0.74	0	0	IS
1.19	1.81	1.13	0	0	IS
1.07	1.73	0.9	0	0	IS
1.4	1.74	0.45	0	0	IS
1.37	1.68	0.65	0	0	IS
1.37	1.56	0.47	0	0	IS
1.15	1.72	1.01	0	0	IS
1.14	1.77	0.98	0	0	IS
1.29	1.69	0.87	0	0	IS
1.47	1.82	0.77	0	0	IS
1.48	1.67	0.32	0	0	IS
1.43	1.94	0.75	0	0	IS
1.33	1.81	0.77	0	0	IS
1.64	1.97	0.96	0	0	IS
1.63	1.99	1.01	0	0	IS
1.83	2.23	0.53	0	0	IS
1.73	2	0.62	0	0	IS

1.53	2.16	1.08	0	0	IS
1.54	2.15	1	0	0	IS
1.76	2.1	0.76	0	0	IS
1.84	2.04	0.57	0	0	IS
1.45	1.87	0.96	0	0	IS
1.38	1.94	0.84	0	0	IS
1.79	2	0.47	0	0	IS
1.69	2.09	0.52	0	0	IS
1.47	1.97	0.76	0	0	IS
1.74	2.03	0.65	0	0	IS
2.01	2.17	0.29	0	0	IS
1.9	2.02	0.37	0	0	IS
1.55	1.99	0.81	0	0	IS
1.59	1.85	0.67	0	0	IS
1.64	1.93	0.48	0	0	IS
1.81	2.17	0.59	0	0	IS
1.56	1.95	0.72	0	0	IS
1.46	2.18	0.89	0	0	IS
1.5	1.94	1.03	0	0	IS
1.49	2.18	1.08	0	0	IS
1.99	2.16	0.54	0	0	IS
1.74	2.07	0.59	0	0	IS
1.7	1.95	0.51	0	0	IS
1.8	2.09	0.53	0	0	IS
1.72	2.05	0.55	0	0	IS
1.79	2	0.57	0	0	IS
1.91	2.33	0.75	0	0	IS
1.55	1.88	0.83	0	0	IS
1.83	2.02	0.34	0	0	IS
1.71	1.98	0.47	0	0	IS
1.65	1.9	0.77	0	0	IS
1.59	1.98	0.83	0	0	IS
1.78	2.05	0.47	0	0	IS
1.66	2.21	0.93	0	0	IS
1.58	2.03	0.83	0	0	IS
1.86	2.04	0.39	0	0	IS
1.9	2.1	0.37	0	0	IS
2	2.16	0.57	0	0	IS
1.79	2.11	0.85	0	0	PL
1.62	1.85	0.38	0	0	IS
1.65	1.9	0.43	0	0	IS
1.78	2.02	0.56	0	0	IS
1.58	1.81	0.51	0	0	IS
1.67	2	0.57	0	0	IS
1.73	1.91	0.45	0	0	IS
1.89	2.01	0.3	0	0	IS
1.68	2.01	0.49	0	0	IS
1.69	2.06	0.68	0	0	IS
1.86	2.18	0.6	0	0	IS
1.7	2.17	0.72	0	0	IS
1.8	2.02	0.53	0	0	IS
1.71	1.97	0.64	0	0	IS
1.82	2	0.33	0	0	IS
1.23	1.45	0.42	0	0	IS

1.55	2.14	1.11	0	0	PL
1.36	1.65	0.66	0	0	IS
1.47	1.66	0.41	0	0	IS
1.65	2.04	0.54	0	0	IS
1.67	2.09	0.64	0	0	IS
1.43	1.63	0.58	0	0	IS
1.4	1.72	0.46	0	0	IS
1.29	1.69	0.55	0	0	IS
1.6	1.93	0.64	0	0	IS
1.19	1.67	0.65	0	0	IS
1.22	1.58	0.53	0	0	IS
1.26	1.49	0.56	0	0	IS
1.45	1.57	0.33	0	0	IS
1.47	1.66	0.4	0	0	IS
1.69	1.94	0.5	0	0	IS
1.49	1.66	0.38	0	0	IS
1.29	1.5	0.36	0	0	IS
1.61	1.85	0.58	0	0	IS
1.72	1.88	0.33	0	0	IS
1.52	1.78	0.6	0	0	IS
1.56	1.9	0.46	0	0	IS
1.54	1.78	0.5	0	0	IS
1.67	1.83	0.43	0	0	IS
1.66	1.82	0.45	0	0	IS
1.66	2.05	0.74	0	0	IS
1.67	1.97	0.59	0	0	IS
1.53	1.82	0.46	0	0	IS
1.84	2.09	0.44	0	0	IS
1.81	2.09	0.46	0	0	IS
1.67	1.82	0.37	0	0	IS
1.75	1.99	0.39	0	0	IS
1.67	1.87	0.56	0	0	IS
1.28	1.4	0.4	0	0	IS
1.34	1.47	0.28	0	0	IS
1.14	1.47	0.71	0	0	IS
0.87	0.99	0.27	0	0	PL
0.56	0.69	0.18	0	0	IS
0.97	1.06	0.24	0	0	PL
1.27	1.41	0.24	0	0	PL
0.93	1.09	0.3	0	0	PL
1.09	1.23	0.26	0	0	PL
0.96	1.14	0.45	0	0	IS
1.22	1.43	0.42	0	0	PL
0.74	0.97	0.39	0	0	IS
0.97	1.17	0.4	0	0	IS
1.38	1.65	0.32	0	0	IS
1.4	1.71	0.48	0	0	PL
1.26	1.59	0.52	0	0	IS
1.15	1.29	0.46	0	0	IS
1.03	1.29	0.68	0	0	PL
0.9	1.59	1.09	0	0	PL
0.98	1.35	0.71	0	0	IS
1.53	1.86	0.17	0	0	IS
1.51	1.83	0.54	0	0	IS

1.47	1.95	0.59	0	0	IS
0.83	1.53	1	0	0	IS
1.02	1.39	0.72	0	0	IS
1.04	1.33	0.63	0	0	PL
1.16	1.36	0.52	0	0	PL
0.96	1.15	0.43	0	0	PL
0.92	1.29	0.63	0	0	PL
1.68	1.92	0.44	0	0	IS
0.94	1.3	0.68	0	0	PL
1.1	1.44	0.63	0	0	IS
1.03	1.33	0.64	0	0	PL
1.02	1.21	0.4	0	0	IS
0.87	1.03	0.32	0	0	PL
1.09	1.25	0.42	0	0	IS
1.51	1.89	0.75	0	0	IS
1.68	1.91	0.6	0	0	IS
1.78	2.01	0.42	0	0	IS
1.78	1.96	0.42	0	0	IS
1.99	2.18	0.33	0	0	IS
2.07	2.21	0.27	0	0	IS
1.73	2.13	0.64	0	0	IS
1.85	2.17	0.51	0	0	IS
1.77	2.08	0.57	0	0	IS
1.91	2.16	0.38	0	0	IS
1.89	2.06	0.3	0	0	IS
1.64	1.89	0.43	0	0	IS
1.76	1.97	0.4	0	0	IS
1.8	2.14	0.62	0	0	IS
1.87	2.11	0.42	0	0	IS
1.85	2.01	0.28	0	0	IS
1.54	1.91	0.58	0	0	IS
1.72	1.98	0.41	0	0	IS
1.64	1.89	0.46	0	0	IS
1.67	1.86	0.17	0	0	IS
1.49	1.92	0.68	0	0	IS
1.66	1.86	0.38	0	0	IS
1.43	1.9	0.83	0	0	IS
1.53	1.85	0.57	0	0	IS
1.34	1.69	0.72	0	0	PL
1.71	2	0.45	0	0	IS
1.47	1.92	0.78	0	0	IS
1.36	1.74	0.33	0	0	IS
1.39	1.89	0.76	0	0	IS
1.46	1.79	0.69	0	0	IS
1.48	1.83	0.31	0	0	IS
1.45	1.77	0.6	0	0	IS
1.67	1.87	0.55	0	0	IS
1.37	1.6	0.49	0	0	PL
1.91	2.07	0.62	0	0	IS
1.79	1.95	0.39	0	0	IS
1.56	2.01	0.74	0	0	IS
1.89	2.07	0.48	0	0	IS
1.87	1.99	0.22	0	0	IS
1.51	1.92	0.65	0	0	IS

1.73	1.82	0.19	0	0	IS
1.61	2.08	0.88	0	0	IS
1.69	1.99	0.54	0	0	IS
1.81	2.1	0.56	0	0	IS
1.86	2.16	0.66	0	0	IS
1.7	2.01	0.79	0	0	IS
1.67	2.07	0.73	0	0	IS
1.83	2.02	0.33	0	0	IS
1.44	1.92	1.25	0	0	IS
1.63	2.15	0.78	0	0	IS
1.68	2.18	0.86	0	0	IS
1.21	1.68	1.11	0	0	IS
1.5	2.54	1.83	0	0	IS
1.53	1.76	0.85	0	0	IS
1.47	2.48	1.58	0	0	IS
1.71	2.06	0.76	0	0	IS
1.37	1.95	0.88	0	0	IS
1.47	1.83	0.64	0	0	IS
1.62	1.91	0.53	0	0	IS
1.54	1.71	0.36	0	0	IS
1.35	1.59	0.57	0	0	IS
1.26	1.72	1.04	0	0	IS
1.22	1.97	1.3	0	0	IS
1.54	1.85	0.48	0	0	IS
1.64	2.07	0.44	0	0	IS
1.6	2.14	0.99	0	0	IS
1.48	1.81	0.8	0	0	IS
1.58	1.83	0.44	0	0	IS
1.54	2.03	0.71	0	0	IS
1.33	1.7	0.7	0	0	IS
1.53	1.73	0.57	0	0	IS
1.64	1.94	0.37	0	0	IS
1.86	2.1	0.64	0	0	IS
1.77	2.04	0.51	0	0	IS
1.4	2.1	0.99	0	0	IS
1.26	2.19	1.15	0	0	IS
1.49	1.99	1.35	0	0	IS
1.99	2.54	1.14	0	0	IS
1.46	2.18	1.07	0	0	IS
1.53	2.17	0.89	0	0	IS
1.58	1.95	0.64	0	0	IS
1.52	2.63	1.84	0	0	IS
1.59	2.41	1.68	0	0	IS
1.6	2.52	1.63	0	0	IS
1.57	2.22	1.06	0	0	IS
1.97	2.31	0.83	0	0	IS
1.22	2.17	1.16	0	0	IS
1.85	2.28	0.67	0	0	IS
1.77	2.26	1.33	0	0	IS
1.78	2.4	1.16	0	0	PL
1.53	2.17	1.34	0	0	PL
1.56	2.11	1.06	0	0	IS
1.75	2.59	1.5	0	0	IS
1.91	2.28	0.69	0	0	IS

2.1	2.6	0.7	0	0	IS
1.62	2.5	1.15	0	0	IS
1.73	2.28	1.25	0	0	IS
1.65	2.41	1.24	0	0	IS
1.69	2.32	0.93	0	0	IS
1.69	2.32	0.93	0	0	IS
1.77	2.28	0.73	0	0	IS
1.73	2.35	0.87	0	0	IS
1.81	2.4	1.08	0	0	IS
1.67	2.4	1.16	0	0	IS
1.89	2.28	0.95	0	0	IS
2.08	2.4	0.62	0	0	IS
2.08	2.4	0.62	0	0	IS
2.03	2.34	0.61	0	0	IS
1.87	2.35	0.9	0	0	IS
1.93	2.43	0.94	0	0	IS
1.79	2.35	1.08	0	0	IS
2.19	2.65	0.76	0	0	IS
1.61	2.5	1.24	0	0	IS
1.67	2.4	1.16	0	0	IS
2.04	2.36	0.74	0	0	IS
1.94	2.34	0.85	0	0	IS
1.72	2.72	1.39	0	0	IS
1.76	2.24	0.86	0	0	IS
1.72	2.24	0.97	0	0	IS
2.04	2.43	0.79	0	0	IS
1.89	2.48	1.28	0	0	IS
1.62	2.56	1.24	0	0	IS
2.07	2.34	0.6	0	0	IS
1.83	2.34	1.51	0	0	IS
2.21	2.81	1.24	0	0	IS
1.94	3	1.43	0	0	IS
1.9	2.82	1.68	0	0	IS
2.16	2.5	0.89	0	0	IS
1.9	2.91	1.4	0	0	IS
1.69	2.49	1.23	0	0	IS
1.95	2.82	1.3	0	0	IS
2.02	2.68	0.95	0	0	IS
2.02	2.87	1.29	0	0	IS
1.94	2.42	1.18	0	0	IS
1.72	1.99	1.03	0	0	IS
1.24	2.31	1.79	0	0	IS
1.86	2.78	1.38	0	0	IS
2.14	2.63	1	0	0	IS
1.64	2.13	0.88	0	0	IS
1.67	1.99	0.67	0	0	IS
1.65	2.18	0.97	0	0	IS
1.62	2.07	0.79	0	0	IS
1.66	2.05	0.85	0	0	IS
1.83	2.22	0.61	0	0	IS
1.73	2.25	0.91	0	0	IS
1.92	2.15	0.42	0	0	IS
1.88	2.28	0.71	0	0	IS
1.68	2.09	0.85	0	0	IS

1.54	1.99	0.81	0	0	IS
1.67	2.01	0.58	0	0	IS
1.84	2.28	0.73	0	0	IS
1.49	2.07	1.21	0	0	IS
1.72	2.19	0.81	0	0	IS
1.77	2.17	0.84	0	0	IS
1.47	2.1	1.31	0	0	IS
1.86	2.12	0.48	0	0	IS
1.95	2.23	0.52	0	0	IS
1.78	2.13	0.61	0	0	IS
1.75	2.15	0.74	0	0	IS
1.55	1.98	0.97	0	0	IS
1.78	2.14	0.78	0	0	IS
1.8	2.12	0.62	0	0	IS
1.77	2.68	1.39	0	0	IS
1.72	2.14	0.76	0	0	IS
1.93	2.35	0.92	0	0	IS
1.84	2.35	0.87	0	0	IS
1.69	2.06	1.07	0	0	IS
1.46	2.15	1.53	0	0	IS
1.64	2.02	0.76	0	0	IS
1.68	2.15	0.69	0	0	IS
1.67	2.16	0.86	0	0	IS
1.8	2.15	0.58	0	0	IS
1.68	2.06	0.79	0	0	IS
1.87	2.24	0.69	0	0	IS
1.57	1.98	0.87	0	0	IS
1.54	1.95	0.73	0	0	IS
1.61	2.03	0.86	0	0	IS
1.66	2.03	0.77	0	0	IS
1.76	2.01	0.5	0	0	IS
1.73	2.77	2.19	0	0	OC
1.74	2.14	0.87	0	0	HC
2	2.17	0.38	0	0	IS
1.72	2.24	1.19	0	0	OC
1.81	2.24	0.78	0	0	IS
1.79	2.19	0.87	0	0	IS
1.84	2.23	0.75	0	0	IS
1.78	2.18	0.71	0	0	IS
1.97	2.36	0.66	0	0	IS
1.75	2.78	1.65	0	0	IS
1.91	2.4	0.9	0	0	IS
2.06	2.47	0.75	0	0	IS
1.78	2.68	1.76	0	0	IS
1.84	2.49	0.9	0	0	IS
1.77	2.18	0.71	0	0	IS
2.03	2.4	0.64	0	0	IS
1.82	2.34	0.79	0	0	IS
1.96	2.34	0.68	0	0	IS
1.93	2.34	0.92	0	0	IS
1.89	2.34	0.89	0	0	IS
1.68	2.1	0.95	0	0	IS
1.9	2.29	0.64	0	0	IS
1.88	2.27	0.73	0	0	IS

1.81	2.21	0.81	0	0	IS
1.94	2.24	0.77	0	0	IS
1.94	2.3	0.73	0	0	IS
1.86	2.31	0.83	0	0	IS
1.69	2.28	1.2	0	0	IS
2.21	2.62	0.78	0	0	IS
1.82	2.33	0.71	0	0	IS
1.9	2.44	0.88	0	0	IS
1.68	2.51	1.14	0	0	IS
2.06	2.53	0.82	0	0	IS
1.84	2.56	0.9	0	0	IS
1.93	2.15	0.69	0	0	IS
1.78	2.01	0.95	0	0	IS
2.09	2.52	0.93	0	0	IS
1.98	2.11	0.57	0	0	IS
1.97	2.49	0.81	0	0	IS
1.97	2.15	0.45	0	0	IS
2.11	2.34	0.38	0	0	IS
2.02	2.43	0.8	0	0	IS
1.98	2.22	0.48	0	0	IS
2.1	2.47	0.8	0	0	IS
1.89	2.46	0.81	0	0	IS
1.85	2.15	0.76	0	0	IS
1.91	2.31	0.73	0	0	IS
2.05	2.35	0.55	0	0	IS
1.99	2.44	0.82	0	0	IS
1.98	2.39	0.77	0	0	IS
1.82	2.28	0.76	0	0	IS
1.93	2.2	0.53	0	0	IS
1.37	1.94	1.06	0	0	IS
1.79	2.07	0.53	0	0	IS
1.73	2.12	0.59	0	0	IS
1.95	2.36	0.75	0	0	IS
2.03	2.38	0.57	0	0	IS
1.71	2.47	1.02	0	0	IS
2.01	2.34	0.69	0	0	IS
1.8	2.32	0.82	0	0	IS
1.56	2.08	0.85	0	0	BS
1.57	1.99	0.68	0	0	IS
1.92	2.27	0.69	0	0	IS
1.95	2.32	0.68	0	0	IS
1.49	2.07	1.06	0	0	IS
1.82	2.28	0.76	0	0	IS
1.83	2.25	1.09	0	0	IS
2	2.36	0.68	0	0	IS
1.91	2.32	0.73	0	0	IS
1.68	2.05	0.81	0	0	IS
1.77	2.49	1.11	0	0	IS
2.07	2.48	0.75	0	0	IS
1.89	2.26	0.61	0	0	IS
1.76	2.02	0.68	0	0	IS
1.98	2.34	0.63	0	0	IS
1.58	2.02	0.92	0	0	IS
1.52	2.02	0.97	0	0	IS

1.89	2.17	0.44	0	0	IS
1.96	2.2	0.45	0	0	IS
1.87	2.11	0.46	0	0	IS
1.84	2.12	0.5	0	0	IS
1.95	2.31	0.64	0	0	IS
1.79	2.18	0.66	0	0	IS
1.92	2.33	0.6	0	0	IS
1.75	2.2	0.87	0	0	IS
1.58	2.02	0.97	0	0	IS
1.78	2.3	0.7	0	0	IS
2	2.14	0.4	0	0	IS
1.48	2.23	1.09	0	0	IS
1.77	2.12	0.91	0	0	IS
1.71	2.08	0.63	0	0	IS
1.61	1.99	0.95	0	0	IS
1.84	2.24	0.69	0	0	IS
1.9	2.29	0.72	0	0	IS
1.71	2.36	0.91	0	0	IS
1.68	2.47	0.96	0	0	IS
1.94	2.42	0.88	0	0	IS
1.83	2.32	0.94	0	0	IS
1.82	2.27	0.79	0	0	IS
1.83	2.24	0.75	0	0	IS
1.63	2.14	0.71	0	0	IS
1.9	2.23	0.54	0	0	IS
1.84	2.27	0.7	0	0	IS
1.76	2.48	0.92	0	0	IS
1.8	2.25	0.79	0	0	IS
2.13	2.25	0.28	0	0	IS
2.25	2.49	0.56	0	0	IS
1.81	2.36	1.26	0	0	IS
1.69	2.13	0.68	0	0	IS
1.91	2.48	1.1	0	0	IS
1.96	2.7	0.94	0	0	IS
2.18	2.5	0.7	0	0	IS
1.94	2.76	1.12	0	0	IS
1.68	2.79	1.84	0	0	IS
2.07	2.34	0.54	0	0	IS
2.04	2.4	0.65	0	0	IS
1.56	2.43	1.57	0	0	IS
2.02	2.42	0.68	0	0	IS
2.17	2.57	0.62	0	0	IS
2.19	2.49	0.54	0	0	IS
2.08	2.47	0.83	0	0	IS
1.95	2.34	1.01	0	0	IS
2.09	2.49	0.74	0	0	IS
2.16	2.51	0.66	0	0	IS
1.99	2.48	0.77	0	0	IS
1.94	2.3	0.96	0	0	IS
1.74	2.64	1.13	0	0	IS
1.8	2.67	1.15	0	0	IS
1.86	2.46	0.87	0	0	IS
1.97	2.46	0.81	0	0	BS
2.12	2.31	0.37	0	0	IS

1.84	2.25	0.75	0	0	IS
1.97	2.61	1.02	0	0	IS
1.67	2.33	1.03	0	0	IS
2.01	2.32	0.52	0	0	IS
1.99	2.18	0.48	0	0	IS
1.36	1.85	0.96	0	0	IS
1.82	2.28	0.95	0	0	IS
1.91	2.5	0.87	0	0	IS
1.91	2.5	0.87	0	0	IS
1.95	2.25	0.86	0	0	IS
1.76	2.39	0.88	0	0	IS
1.7	2.04	0.92	0	0	IS
1.89	2.32	0.66	0	0	IS
1.83	2.1	0.77	0	0	IS
1.92	2.29	0.57	0	0	IS
1.91	2.2	0.63	0	0	IS
1.76	2.3	0.85	0	0	IS
2.06	2.48	0.75	0	0	IS
2.16	2.54	0.65	0	0	IS
2.07	2.56	0.82	0	0	IS
2.21	2.54	0.55	0	0	IS
1.95	2.45	0.9	0	0	IS
1.98	2.43	0.72	0	0	IS
1.98	2.43	0.72	0	0	IS
1.86	2.3	0.98	0	0	IS
2.15	2.52	0.57	0	0	IS
2.11	2.39	0.63	0	0	IS
1.99	2.72	1	0	0	IS
1.92	2.65	1.05	0	0	IS
2.14	2.76	0.83	0	0	IS
1.92	2.57	1.08	0	0	IS
1.76	2.35	1.34	0	0	IS
1.8	2.56	1.17	0	0	IS
1.92	2.99	1.34	0	0	IS
2.1	2.81	1.22	0	0	IS
2.12	3.02	1.27	0	0	IS
1.9	2.95	1.41	0	0	IS
2.08	2.96	1.25	0	0	IS
2.26	2.78	0.82	0	0	BS
2.2	2.67	0.79	0	0	IS
2.04	2.77	1.15	0	0	IS
2.07	2.89	1.16	0	0	IS
2.2	2.58	0.74	0	0	IS
1.9	2.26	0.99	0	0	IS
1.97	2.46	0.84	0	0	IS
1.83	2.44	1.13	0	0	IS
1.74	2.6	1.33	0	0	IS
2.24	2.79	0.81	0	0	IS
2.19	2.45	0.62	0	0	IS
2.15	2.92	1.1	0	0	IS
2.25	2.79	0.84	0	0	IS
2.17	2.67	0.78	0	0	IS
1.94	2.87	1.32	0	0	IS
1.97	2.94	1.24	0	0	IS

2.07	2.89	1.21	0	0	IS
1.73	2.7	1.79	0	0	IS
2.11	3.02	1.42	0	0	IS
1.88	2.9	1.59	0	0	IS
1.91	3.09	1.5	0	0	IS
1.99	2.75	1.07	0	0	IS
2.06	3.07	1.67	0	0	IS
2.36	2.65	0.59	0	0	IS
2.1	3.06	1.32	0	0	IS
2.08	3.05	1.26	0	0	IS
1.98	3.14	1.66	0	0	IS
2.19	2.79	0.91	0	0	IS
2.04	2.79	1.34	0	0	IS
1.95	2.99	1.65	0	0	IS
2.28	2.77	0.85	0	0	IS
2.28	2.92	0.93	0	0	IS
2.28	2.92	0.93	0	0	IS
2.28	2.84	0.88	0	0	IS
2.3	2.77	0.74	0	0	IS
2.03	2.65	1.33	0	0	IS
2.19	2.81	0.82	0	0	IS
2.06	2.54	1.39	0	0	IS
2.34	3.18	1.27	0	0	IS
2.13	2.93	1.06	0	0	IS
2.13	2.93	1.06	0	0	IS
2.09	2.85	1.02	0	0	IS
2.2	2.84	1.06	0	0	IS
1.95	2.79	1.29	0	0	IS
2.04	2.71	0.94	0	0	IS
1.92	2.32	1.33	0	0	IS
1.88	2.5	1.23	0	0	IS
2.17	2.74	0.82	0	0	IS
1.87	2.71	1.33	0	0	IS
1.67	2.93	1.8	0	0	IS
2.27	2.78	0.77	0	0	IS
2.36	2.78	0.64	0	0	IS
2.24	2.63	0.8	0	0	IS
2.43	3.19	0.99	0	0	IS
2.23	2.95	1.17	0	0	IS
2.36	3.17	1.12	0	0	IS
2.07	2.86	1.18	0	0	IS
2.33	2.97	0.97	0	0	IS
2.1	2.92	1.24	0	0	IS
2.09	2.89	1.37	0	0	IS
1.93	3.04	1.61	0	0	IS
1.91	2.97	1.68	0	0	IS
1.87	3.2	1.77	0	0	IS
2.02	2.45	0.85	0	0	IS
2.09	3.17	1.6	0	0	IS
2.17	3.18	1.54	0	0	IS
2.2	3.19	1.45	0	0	IS
2.02	3.14	1.55	0	0	IS
2.31	3.14	1.2	0	0	IS
2.2	2.85	1.13	0	0	IS

1.84	3.37	2.17	0	0	IS
2.29	3.09	1.51	0	0	IS
1.91	2.9	1.5	0	0	IS
2.19	3.27	1.6	0	0	IS
2.27	3.32	1.43	0	0	IS
2.15	3	1.63	0	0	IS
2.28	3.38	1.34	0	0	IS
2.26	2.95	1.32	0	0	IS
4.96	5.94	1.79	0	0	IS
4.53	5.09	1.31	0	0	IS
4.13	4.68	0.72	0	0	IS
2.4	2.72	0.49	0	0	IS
2.21	3.23	1.5	0	0	IS
2.73	3.74	1.46	0	0	IS
1.68	2.38	0.91	0	0	IS
2.25	3	1.31	0	0	IS
1.6	2.22	0.81	0	0	IS
2.07	3.45	2.04	0	0	IS
1.57	1.72	0.33	0	0	IS
1.5	1.68	0.39	0	0	IS
1.71	1.96	0.46	0	0	IS
2.42	3.44	1.72	0	0	IS
2.37	3.26	1.59	0	0	IS
1.64	1.9	0.46	0	0	IS
2.26	2.83	1.14	0	0	IS
1.55	1.95	0.64	0	0	IS
1.66	2	0.85	0	0	IS
1.62	1.8	0.37	0	0	IS
1.4	1.93	0.85	0	0	IS
1.77	2.46	1.07	0	0	IS
2.38	3.16	1.09	0	0	IS
3.26	3.8	0.92	0	0	IS
3.87	4.36	1.45	0	0	IS
2.13	2.4	0.48	0	0	IS
2.24	2.66	0.45	0	0	IS
2.6	3.92	1.59	0	0	IS
1.89	2.14	0.58	0	0	IS
2.48	3.27	0.87	0	0	IS
4.11	5.11	2.04	0	0	IS
3.74	5.25	2.7	0	0	IS
2.74	3.02	0.69	0	0	IS
2.76	2.94	0.38	0	0	IS
1.87	2	0.17	0	0	IS
1.58	1.89	0.78	0	0	IS
1.03	1.06	0.15	0	0	IS
1.37	1.48	0.4	0	0	IS
0.99	1.16	0.19	0	0	IS
0.93	1.11	0.21	0	0	IS
1.32	1.47	0.48	0	0	IS
1.1	1.68	0.66	0	0	IS
1.03	1.36	0.35	0	0	IS
1.27	1.54	0.52	0	0	IS
1.16	1.32	0.34	0	0	IS
1.59	1.68	0.78	0	0	IS

1.57	1.67	0.51	0	0	IS
1.43	1.67	0.57	0	0	IS
1.22	1.55	0.59	0	0	IS
1.25	1.6	0.67	0	0	IS
1.28	1.56	0.59	0	0	IS
1.95	2.04	0.38	0	0	IS
1.72	1.86	0.34	0	0	IS
1.45	1.84	0.75	0	0	PL
1.43	1.95	0.89	0	0	IS
1.67	2.34	1.35	0	0	IS
0.98	1.11	0.35	0	0	PL
1.05	1.28	0.33	0	0	IS
1.24	1.51	0.59	0	0	IS
0.7	1.01	0.36	0	0	IS
0.9	1.27	0.51	0	0	IS
0.79	0.93	0.25	0	0	IS
0.96	1.14	0.34	0	0	IS
0.84	1.06	0.29	0	0	IS
0.85	0.87	0.04	0	0	IS
0.67	0.69	0.04	0	0	OC
0.48	0.74	0.5	5	16.95	HC
0.44	0.66	0.42	5	16.84	HC
1.49	1.66	0.34	0	0	PL
1.57	1.75	0.53	0	0	PL
1.63	1.77	0.37	0	0	PL
0.99	1.16	0.28	0	0	IS
0.97	1.27	0.36	0	0	IS
1.2	1.51	0.51	0	0	IS
1.08	1.4	0.48	0	0	IS
0.94	1.19	0.35	0	0	IS
1.21	1.34	0.19	0	0	IS
1.58	1.82	0.44	0	0	IS
1.69	1.82	0.27	0	0	IS
1.63	1.86	0.52	0	0	IS
1.53	1.55	0.04	0	0	IS
1.6	1.73	0.2	0	0	IS
1.48	1.59	0.21	0	0	IS
1.63	1.87	0.39	0	0	IS
1.73	1.86	0.42	0	0	IS
1.86	1.98	0.32	0	0	IS
1.79	1.93	0.31	0	0	IS
1.83	1.97	0.34	0	0	IS
1.73	1.9	0.31	0	0	IS
1.67	1.88	0.52	0	0	IS
1.58	1.86	0.55	0	0	IS
2.07	2.1	0.05	0	0	IS
1.67	1.94	0.75	0	0	IS
1.82	2.09	0.57	0	0	IS
1.71	1.98	0.3	0	0	IS
1.66	2.02	0.82	0	0	IS
1.79	2.04	0.58	0	0	IS
1.95	2.11	0.56	0	0	IS
2.1	2.14	1.05	0	0	IS
2.2	2.24	1.1	0	0	IS

2.07	2.31	0.7	0	0	IS
1.91	2.26	1.13	0	0	IS
2.14	2.18	0.34	0	0	IS
1.99	2.19	0.76	0	0	IS
2.25	2.53	0.69	0	0	IS
2.25	2.52	0.54	0	0	IS
1.85	2.1	0.55	0	0	IS
1.9	2.12	0.45	0	0	IS
1.14	1.41	0.46	0	0	IS
1.41	1.56	0.39	0	0	IS
1.58	1.81	0.5	0	0	PL
1.64	1.99	0.63	0	0	IS
1.66	2.07	0.69	0	0	IS
1.62	1.78	0.36	0	0	PL
1.52	1.85	0.67	0	0	PL
1.49	1.84	0.69	0	0	PL
2.07	2.34	0.73	0	0	IS
1.85	1.99	0.28	0	0	IS
2.61	3.24	1.23	0	0	IS
1.89	2.24	0.64	0	0	IS
1.39	1.89	0.89	0	0	IS
1.75	2.11	0.86	0	0	IS
1.22	1.48	0.41	0	0	IS
1.8	2.16	0.66	0	0	IS
1.19	1.56	0.63	0	0	IS
1.47	1.67	0.54	0	0	PL
1.08	1.34	0.6	0	0	IS
1.26	1.58	0.51	0	0	IS
1.55	1.7	0.38	0	0	IS
1.39	1.55	0.51	0	0	IS
1.09	1.4	0.69	0	0	IS
1.16	1.76	1.08	0	0	IS
1.51	1.73	0.39	0	0	IS
1.36	1.55	0.46	0	0	PL
1.53	1.65	0.58	0	0	IS
1.65	1.75	0.23	0	0	IS
1.89	2.06	0.41	0	0	IS
1.74	1.89	0.37	0	0	PL
1.62	1.81	0.33	0	0	IS
1.41	1.61	0.58	0	0	IS
1.69	1.83	0.27	0	0	IS
1.36	1.55	0.36	0	0	PL
1.62	1.79	0.34	0	0	IS
1.43	1.59	0.41	0	0	PL
1.57	1.78	0.6	0	0	IS
1.72	1.84	0.44	0	0	IS
1.07	1.48	0.62	0	0	IS
1.62	1.74	0.25	0	0	IS
1.86	1.96	0.35	0	0	IS
1.8	1.98	0.37	0	0	IS
1.81	1.94	0.35	0	0	IS
1.77	2.35	0.86	0	0	IS
1.75	1.9	0.34	0	0	IS
1.55	1.79	0.62	0	0	PL

1.63	1.78	0.44	0	0	PL
1.55	1.81	0.63	0	0	PL
1.57	1.81	0.47	0	0	PL
1.71	1.88	0.37	0	0	IS
1.8	1.92	0.36	0	0	IS
1.67	1.83	0.39	0	0	PL
1.63	1.81	0.39	0	0	IS
1.47	1.64	0.29	0	0	IS
1.42	1.6	0.3	0	0	IS
1.67	1.83	0.39	0	0	IS
1.69	2.06	0.67	0	0	IS
2.13	2.45	0.51	0	0	IS
1.79	2.13	0.74	0	0	IS
1.54	1.74	0.31	0	0	IS
2.09	2.33	0.44	0	0	IS
1.88	2.01	0.28	0	0	IS
1.97	2.25	0.36	0	0	IS
2.1	2.23	0.32	0	0	IS
2.1	2.36	0.4	0	0	IS
1.97	2.42	0.67	0	0	IS
2.11	2.37	0.5	0	0	IS
2.16	2.44	0.49	0	0	IS
2.16	2.46	0.58	0	0	IS
1.44	2.11	0.89	0	0	IS
1.87	1.99	0.23	0	0	IS
1.37	1.6	0.49	0	0	IS
2.15	2.32	0.31	0	0	IS
2.02	2.56	0.82	0	0	IS
2.31	2.49	0.31	0	0	IS
1.61	1.94	0.62	0	0	HC
1.95	2.33	0.66	0	0	IS
2.2	2.5	0.57	0	0	IS
1.78	2.1	0.42	0	0	IS
2.3	2.64	0.56	0	0	IS
2.32	2.44	0.32	0	0	IS
2.19	2.6	0.63	0	0	IS
3.64	4.18	1.46	0	0	IS
3.24	3.96	1.59	0	0	IS
3.02	4.11	1.63	0	0	IS
3.09	3.95	1.6	0	0	IS
3.2	4.21	1.66	0	0	IS
3.01	4.28	2.04	0	0	IS
3.18	3.91	1.52	0	0	IS
2.7	3.23	0.69	0	0	IS
2.75	3.27	0.88	0	0	IS
2.39	3.16	1.08	0	0	IS
2.94	3.62	1.13	0	0	IS
2.07	2.83	1.13	0	0	IS
2.51	3.2	0.92	0	0	IS
2.76	3.37	1.11	0	0	IS
2.21	3.69	1.93	0	0	IS
2.88	3.8	1.18	0	0	IS
2.87	4.9	2.77	0	0	IS
3.62	5.15	2.82	0	0	IS

4.73	6.28	2.9	0	0	IS
4.32	4.84	1.5	0	0	IS
3.16	3.63	1.3	0	0	IS
2.98	3.43	1.04	0	0	IS
2.77	3.29	0.93	0	0	IS
2.91	3.94	2	0	0	IS
2.53	3.86	1.98	0	0	IS
2.44	2.83	0.78	0	0	IS
2.17	2.76	1.01	0	0	IS
3.02	3.59	1.24	0	0	IS
2.29	3.24	1.44	0	0	IS
2.46	3.56	1.76	0	0	IS
3.31	4.06	1.35	0	0	IS
2.64	3.95	1.74	0	0	IS
2.86	3.95	1.39	0	0	IS
2.62	3.85	1.83	0	0	IS
2.84	4	1.55	0	0	IS
2.52	4.05	2.04	0	0	IS
3.03	3.82	1.12	0	0	IS
2.97	4.14	1.57	0	0	IS
3.42	4.66	2.21	0	0	IS
3.14	4.41	1.94	0	0	IS
3.03	4.04	1.95	0	0	IS
3.29	4.33	1.72	0	0	IS
3.94	5.01	1.81	0	0	IS
2.54	3.54	1.71	0	0	IS
3.81	4.94	1.99	0	0	IS
3.34	4.45	2	0	0	IS
2.32	3.49	1.68	0	0	IS
3.28	4.58	2.16	0	0	IS
3.82	4.38	1.28	0	0	IS
4.51	5.57	2.12	0	0	IS
4.69	5.37	1.48	0	0	IS
3.96	4.95	1.9	0	0	IS
3.65	5.2	2.49	0	0	IS
4.14	5.07	1.79	0	0	IS
4.18	4.76	1.51	0	0	IS
3.72	4.79	1.72	0	0	IS
3.8	4.92	2.13	0	0	IS
3.36	3.99	1.81	0	0	IS
3.08	4.32	2.06	0	0	IS
3.64	4.42	2.2	0	0	IS
4.16	5.95	2.91	0	0	IS
5.12	6.57	3.23	0	0	IS
3.57	4.42	1.9	0	0	IS
4.02	5.1	2.22	0	0	IS
3.44	5.59	3.39	0	0	IS
4.12	5.16	2.8	0	0	IS
4.2	5.25	2.07	0	0	IS
4.01	5.07	2.25	0	0	IS
5.24	6.98	3.45	0	0	IS
5.33	6.44	3.06	0	0	IS
5.18	6.79	3.16	0	0	IS
4.68	6.36	3.3	0	0	IS

3.67	5.25	2.41	0	0	IS
4.5	6.63	3.51	0	0	IS
5.09	7.42	4.97	0	0	IS
5.41	6.71	2.41	0	0	IS
3.67	4.94	2.39	0	0	IS
3.43	5.26	2.69	0	0	IS
3.1	4.74	2.26	0	0	IS
3.08	4.16	1.97	0	0	IS
3.49	4.23	1.46	0	0	IS
3.03	4.06	1.81	0	0	IS
3.01	4.17	1.76	0	0	IS
2.96	4.54	2.29	0	0	IS
3.04	4.27	1.86	0	0	IS
3.3	4.15	1.56	0	0	IS
2.74	4.53	2.48	0	0	IS
4.3	6.1	3.33	0	0	IS
2.72	4.54	2.18	0	0	IS
2.77	4.18	2.4	0	0	IS
3.33	4.92	2.57	0	0	BS
3.47	4.8	2.51	0	0	IS
3.54	4.75	2.19	0	0	IS
3.37	4.66	2.38	0	0	IS
3.79	4.69	1.99	0	0	IS
3.76	4.73	1.68	0	0	IS
3.64	5.27	2.6	0	0	IS
3.14	4.9	2.63	0	0	BS
3.17	5.06	2.66	0	0	IS
3.5	5.03	2.69	0	0	BS
3.38	4.61	2.34	0	0	IS
2.72	4.52	2.47	0	0	IS
3.51	4.4	2.25	0	0	IS
2.81	4.36	2.22	0	0	IS
3.12	4.59	2.33	0	0	IS
3.03	4.5	2.4	0	0	IS
3.21	4.37	2.13	0	0	IS
2.96	4.64	2.83	0	0	IS
3.25	5.02	2.84	0	0	IS
2.75	5.03	2.9	0	0	IS
3.09	4.34	2.31	0	0	IS
3.25	4.73	2.29	0	0	IS
4.47	6.05	3.24	0	0	IS
4.05	5.46	2.96	0	0	IS
4.34	5.72	3.28	0	0	IS
3.72	5.79	3.38	0	0	IS
3.37	5.26	3.11	0	0	IS
3.26	5.16	3.22	0	0	IS
3.7	5.52	2.77	0	0	IS
2.77	3.5	1.09	0	0	IS
2.85	3.61	1.2	0	0	IS
2.85	3.41	0.86	0	0	IS
2.75	3.41	0.95	0	0	IS
2.78	3.29	0.74	0	0	IS
2.83	3.58	1.2	0	0	IS
3.41	4.13	1.57	0	0	IS

2.64	3.76	1.78	0	0	IS
2.67	3.81	1.91	0	0	IS
2.67	4.04	1.78	0	0	IS
2.54	3.33	0.95	0	0	IS
3.6	5.13	2.57	0	0	BS
2.56	3.37	1.59	0	0	IS
2.81	3.46	1.25	0	0	IS
2.89	3.75	1.19	0	0	IS
2.65	3.68	1.55	0	0	IS
3.6	5.11	2.81	0	0	IS
2.46	3.22	1.16	0	0	IS
2.51	3.5	1.3	0	0	IS
2.83	4.17	2.02	0	0	IS
2.72	4	1.76	0	0	IS
2.72	3.56	1.11	0	0	IS
2.46	3.51	1.43	0	0	IS
2.85	3.89	1.74	0	0	IS
2.7	3.71	1.38	0	0	IS
2.42	3.76	1.7	0	0	IS
2.63	3.73	1.38	0	0	IS
2.69	3.75	1.55	0	0	IS
2.71	3.72	1.62	0	0	IS
2.61	3.68	1.38	0	0	IS
2.51	3.58	1.33	0	0	IS
3.34	4.37	1.91	0	0	IS
2.36	3.33	1.45	0	0	IS
2.6	3.8	1.4	0	0	IS
2.54	3.66	1.66	0	0	IS
2.68	3.62	1.19	0	0	IS
2.51	3.83	1.67	0	0	IS
2.89	3.84	1.26	0	0	IS
2.72	3.8	1.55	0	0	IS
2.29	4.04	2.49	0	0	IS
2.27	3.17	1.65	0	0	IS
2.53	3.61	1.54	0	0	IS
2.49	3.7	2.22	0	0	IS
2.65	3.76	1.8	0	0	IS
2.31	3.79	1.9	0	0	IS
2.91	4.33	2.21	0	0	IS
2.88	3.98	1.61	0	0	IS
2.93	3.98	1.71	0	0	IS
2.7	3.75	1.51	0	0	IS
2.87	3.64	1.4	0	0	IS
2.63	3.67	1.45	0	0	IS
2.54	3.65	1.56	0	0	IS
2.93	3.7	1.34	0	0	IS
1.95	2.7	0.99	0	0	IS
2.1	2.53	0.61	0	0	IS
2.11	2.39	0.63	0	0	IS
2.06	2.35	0.55	0	0	IS
2.02	2.33	0.58	0	0	IS
1.79	2.06	0.46	0	0	IS
1.79	2.06	0.54	0	0	IS
2.03	2.23	0.44	0	0	IS

1.99	2.21	0.56	0	0	IS
2.18	2.57	0.53	0	0	IS
2.1	2.38	0.58	0	0	IS
2.06	2.26	0.49	0	0	IS
2.08	2.45	0.69	0	0	IS
1.75	2.3	0.92	0	0	IS
2.65	4.02	1.92	0	0	IS
1.9	2.24	0.6	0	0	IS
1.8	2.24	1.06	0	0	IS
2.64	4.08	2.19	0	0	IS
2.72	3.49	1.34	0	0	IS
2.54	3.84	1.85	0	0	IS
2.04	2.58	0.8	0	0	IS
2.25	2.57	0.53	0	0	IS
2.06	2.44	0.65	0	0	IS
1.79	2.34	0.79	0	0	IS
2.1	2.77	1.01	0	0	IS
2.22	2.5	0.58	0	0	IS
1.95	2.27	0.61	0	0	IS
1.64	2.39	1.06	0	0	IS
1.94	2.93	1.54	0	0	IS
1.82	2.54	1.02	0	0	IS
1.77	2.48	1.01	0	0	IS
2.16	2.37	0.38	0	0	IS
3.23	4.37	1.99	0	0	IS
1.78	2.14	0.86	0	0	IS
2.06	2.2	0.3	0	0	IS
2.67	3.37	1.37	0	0	IS
2.69	3.68	1.55	0	0	IS
2.14	2.44	0.56	0	0	IS
1.95	2.25	0.53	0	0	IS
2.98	3.44	0.82	0	0	IS
1.99	2.32	0.56	0	0	IS
2.83	3.75	1.73	0	0	IS
2.01	2.38	0.66	0	0	IS
1.66	2.05	0.94	0	0	IS
2.26	2.72	0.66	0	0	IS
2.16	2.57	0.63	0	0	IS
2.3	2.79	0.67	0	0	IS
2.51	2.63	0.5	0	0	IS
1.69	2.72	1.26	0	0	IS
2.03	2.41	0.78	0	0	IS
2.17	2.58	0.54	0	0	IS
2.11	2.41	0.64	0	0	IS
2.19	2.64	0.63	0	0	IS
2.27	2.55	0.47	0	0	IS
2.21	2.37	0.42	0	0	IS
1.84	2.45	0.84	0	0	IS
2.06	2.63	0.88	0	0	IS
2.03	2.3	0.61	0	0	IS
1.87	2.95	1.78	0	0	BS
2.38	2.82	0.69	0	0	IS
1.83	2.44	0.94	0	0	IS
1.73	2.36	0.98	0	0	IS

1.93	2.62	0.88	0	0	IS
1.72	2.23	0.91	0	0	IS
2.07	2.22	0.53	0	0	IS
2.02	2.57	1.01	0	0	IS
2.2	2.61	0.64	0	0	IS
2.01	2.84	1.09	0	0	IS
2.04	2.62	0.79	0	0	IS
1.9	2.69	1.03	0	0	IS
1.86	2.27	0.79	0	0	IS
1.96	2.24	0.75	0	0	IS
2.03	2.41	0.63	0	0	IS
1.85	2.28	0.85	0	0	IS
1.81	2.21	0.68	0	0	IS
2.03	2.19	0.39	0	0	IS
2.08	2.66	1.04	0	0	IS
2.13	2.78	1.01	0	0	IS
1.95	2.49	0.86	0	0	IS
2.02	2.57	1.02	0	0	IS
2.12	2.6	0.8	0	0	IS
2	2.33	0.89	0	0	IS
2.15	2.47	0.6	0	0	IS
2.03	2.47	0.85	0	0	IS
2.11	2.56	0.7	0	0	IS
2.16	2.83	0.85	0	0	IS
2.04	2.54	1.08	0	0	IS
2.09	2.63	0.95	0	0	IS
2.2	2.68	0.84	0	0	IS
2.38	2.88	0.75	0	0	IS
2.36	2.77	0.96	0	0	IS
2.18	2.8	0.85	0	0	IS
2.02	2.87	1.14	0	0	IS
2.24	2.71	0.64	0	0	IS
1.99	2.73	1.37	0	0	IS
2.27	2.88	0.87	0	0	IS
2.37	3.04	1.02	0	0	IS
2.22	2.6	0.7	0	0	IS
2.28	2.8	0.83	0	0	IS
2.19	2.76	0.88	0	0	IS
2.27	2.5	0.44	0	0	IS
2.25	2.8	0.71	0	0	IS
2.2	2.66	0.62	0	0	IS
2.26	2.5	0.4	0	0	IS
2.24	2.58	0.74	0	0	IS
2.02	2.82	1.14	0	0	IS
2.29	3.02	1.03	0	0	IS
2.18	2.63	0.57	0	0	IS
2.37	3.11	1	0	0	IS
2.19	2.97	1.06	0	0	IS
2.3	2.98	0.93	0	0	IS
2.48	3.31	1.31	0	0	IS
1.66	2.65	1.46	0	0	IS
2.31	3.07	1.47	0	0	IS
1.76	2.2	1.22	0	0	IS
2.23	2.85	1.44	0	0	IS

2.11	2.83	1.12	0	0	IS
1.93	2.65	1.22	0	0	IS
2.01	3.06	1.48	0	0	IS
2.35	3.07	0.95	0	0	IS
2.42	3.39	1.21	0	0	IS
2.12	2.64	0.76	0	0	IS
2.31	3.25	1.25	0	0	IS
2.78	3.47	1.2	0	0	IS
2.46	3.44	1.57	0	0	IS
1.77	2.05	0.53	0	0	IS
1.63	2.03	0.59	0	0	IS
1.99	2.25	0.55	0	0	IS
1.73	2.08	0.56	0	0	IS
1.83	1.98	0.24	0	0	IS
1.87	2.15	0.48	0	0	IS
1.83	2.08	0.5	0	0	IS
1.75	1.98	0.64	0	0	IS
2.03	2.21	0.34	0	0	IS
1.95	2.18	0.43	0	0	IS
2.29	2.7	0.59	0	0	IS
2.06	2.52	0.76	0	0	IS
2.29	2.51	0.36	0	0	IS
2.12	2.35	0.47	0	0	IS
1.99	2.24	0.57	0	0	IS
2.19	2.34	0.47	0	0	IS
1.85	2.1	0.44	0	0	IS
1.91	2.12	0.47	0	0	IS
2.04	2.63	0.8	0	0	IS
1.9	2.34	0.72	0	0	IS
1.89	2.14	0.42	0	0	IS
2.25	2.53	0.44	0	0	IS
2.2	2.78	0.77	0	0	IS
2.02	2.4	0.64	0	0	IS
2.13	2.38	0.42	0	0	IS
2.15	2.46	0.5	0	0	IS
2.15	2.43	0.45	0	0	IS
2.08	2.65	0.84	0	0	IS
2.05	2.25	0.32	0	0	IS
2.16	2.26	0.28	0	0	IS
1.91	2.3	0.57	0	0	IS
2.16	3.03	1.18	0	0	IS
2.12	2.59	0.67	0	0	IS
2.19	2.56	0.72	0	0	IS
2.39	3.26	1.03	0	0	IS
2.16	2.67	0.76	0	0	IS
2.28	2.41	0.38	0	0	IS
1.98	2.36	0.61	0	0	IS
2.22	2.44	0.34	0	0	IS
2.24	2.97	0.91	0	0	IS
2.1	2.6	0.74	0	0	IS
1.95	3.13	1.51	0	0	IS
1.93	3.03	1.51	0	0	IS
2.08	2.47	0.64	0	0	IS
2.17	2.95	1.1	0	0	IS

2.11	2.84	1.24	0	0	IS
2.15	3.09	1.24	0	0	IS
2.4	2.89	1.13	0	0	IS
2.15	3.21	1.24	0	0	IS
2.39	3.2	1.11	0	0	IS
2.25	2.92	0.85	0	0	IS
2.43	3.08	1.11	0	0	IS
2.17	2.77	0.86	0	0	IS
2.32	3.64	1.68	0	0	IS
2.25	2.72	0.8	0	0	IS
2.39	2.71	0.72	0	0	IS
2.24	3.27	1.47	0	0	IS
2.42	3.16	1.18	0	0	IS
1.88	2.68	1.25	0	0	IS
2.23	2.68	0.74	0	0	IS
2.15	2.66	0.78	0	0	IS
2.19	3.6	1.6	0	0	IS
2.07	2.88	1.22	0	0	IS
2.2	3.09	1.32	0	0	IS
2.3	2.95	0.99	0	0	IS
2.28	2.83	0.95	0	0	IS
2.2	2.88	0.94	0	0	IS
2.08	2.8	1.37	0	0	IS
2.08	3.01	1.18	0	0	IS
1.47	1.89	0.74	0	0	IS
1.62	2.14	0.96	0	0	IS
2.4	3.15	1.23	0	0	IS
2.27	3.05	0.99	0	0	IS
2.58	2.95	0.88	0	0	IS
2.35	3.36	1.26	0	0	IS
2.47	3.23	1.13	0	0	IS
2.16	3.32	1.55	0	0	IS
2.25	3.06	1.06	0	0	IS
2.25	3.02	1.01	0	0	IS
2.49	3.25	1.3	0	0	IS
2.59	3.43	1.41	0	0	IS
2.43	3.35	1.29	0	0	IS
2.31	3.32	1.39	0	0	IS
2.33	3.05	1.51	0	0	IS
2.41	3.19	1.12	0	0	IS
2.41	3.19	1.39	0	0	IS
2.47	3.05	0.78	0	0	IS
2.19	2.91	1.4	0	0	IS
2.36	3.25	1.21	0	0	IS
2.49	3.18	0.84	0	0	IS
2.39	3.15	1.38	0	0	IS
2.58	3.3	0.99	0	0	IS
2.59	3.43	1.26	0	0	IS
2.46	3.43	1.16	0	0	IS
2.42	2.65	0.42	0	0	IS
2.4	3.31	1.15	0	0	IS
2.3	3.21	1.12	0	0	IS
2.62	3.4	1.24	0	0	IS
2.32	3.1	1.25	0	0	IS

2.17	2.92	1.1	0	0	IS
2.12	2.73	1.39	0	0	IS
2.28	3.11	1.5	0	0	IS
2.19	2.95	1.15	0	0	IS
2.13	3.02	1.47	0	0	IS
2.2	2.89	0.92	0	0	IS
2.28	3.1	1.02	0	0	IS
2.38	2.88	0.62	0	0	IS
2.61	2.82	0.49	0	0	IS
2.32	2.63	0.48	0	0	IS
2.07	2.84	1.18	0	0	IS
2.43	2.77	0.62	0	0	IS
2.34	2.66	0.55	0	0	IS
2.09	2.5	0.72	0	0	IS
2.08	2.64	0.84	0	0	IS
2.4	2.91	0.75	0	0	IS
2.39	2.71	0.66	0	0	IS
2.39	2.87	0.9	0	0	IS
2.37	2.76	0.77	0	0	IS
2.28	3.09	1.16	0	0	IS
2.29	2.98	0.92	0	0	IS
2.28	2.88	0.93	0	0	IS
2.3	2.55	0.55	0	0	IS
2.32	2.77	0.77	0	0	IS
2.22	2.77	1.01	0	0	IS
2.36	2.79	0.61	0	0	IS
1.97	2.81	1.22	0	0	IS
2.21	2.7	0.75	0	0	IS
2.28	2.83	0.93	0	0	IS
2.13	2.79	0.95	0	0	IS
2.09	3.02	1.27	0	0	IS
2.38	2.96	0.97	0	0	IS
2.1	2.81	1.13	0	0	IS
2.34	2.96	0.78	0	0	IS
1.95	2.63	0.93	0	0	IS
2.27	3.17	1.24	0	0	IS
2.19	2.57	0.55	0	0	IS
1.92	2.83	1.07	0	0	IS
2.16	3.03	1.12	0	0	IS
2.22	2.69	0.83	0	0	IS
2.23	2.72	0.96	0	0	IS
2.36	2.7	0.55	0	0	IS
2.43	3.05	1.03	0	0	IS
2.47	2.75	0.64	0	0	IS
2.37	3.04	1.21	0	0	IS
2.49	2.99	0.78	0	0	IS
2.52	2.83	0.49	0	0	IS
2.36	2.63	0.58	0	0	IS
2.04	2.71	0.89	0	0	IS
2.24	3.19	1.26	0	0	IS
2.25	2.88	0.95	0	0	IS
2.47	3.09	0.88	0	0	IS
2.3	2.84	0.85	0	0	IS
2.14	2.83	0.91	0	0	IS

2.39	2.86	0.6	0	0	IS
2.23	2.9	1.1	0	0	IS
2.47	2.76	0.41	0	0	IS
2.51	2.87	0.58	0	0	IS
1.5	1.92	0.71	0	0	IS
2.09	2.48	0.6	0	0	IS
2.04	2.35	0.52	0	0	IS
1.97	2.56	0.83	0	0	IS
2.17	2.62	0.61	0	0	IS
2.32	2.78	0.72	0	0	IS
2.27	3.12	1.06	0	0	IS
2.02	2.75	0.97	0	0	IS
2.02	2.45	0.66	0	0	IS
2.01	2.23	0.32	0	0	IS
2.34	2.49	0.27	0	0	IS
2.37	2.57	0.35	0	0	IS
2.45	2.79	0.49	0	0	IS
2.36	2.81	0.8	0	0	IS
2.06	2.61	0.8	0	0	IS
2.24	2.55	0.5	0	0	IS
2.27	2.99	1.06	0	0	IS
2.32	2.45	0.24	0	0	IS
1.62	2.31	1	0	0	IS
2.31	2.45	0.27	0	0	IS
2.27	2.58	0.46	0	0	IS
2.62	3.17	0.73	0	0	IS
2.18	2.46	0.51	0	0	IS
2.38	2.95	0.81	0	0	IS
2.44	3.61	1.4	0	0	IS
2.65	3.18	0.86	0	0	IS
2.38	3.66	1.58	0	0	IS
2.5	3.26	1.03	0	0	IS
2.32	2.87	0.82	0	0	IS
2.41	2.96	0.85	0	0	IS
2.41	2.83	0.76	0	0	IS
2.35	2.76	1.01	0	0	IS
2.27	3.04	1.04	0	0	IS
2.59	3.06	0.76	0	0	IS
2.25	3.1	1.4	0	0	IS
2.06	3.36	1.76	0	0	IS
2.33	3.24	1.22	0	0	IS
2.35	2.87	0.73	0	0	IS
2.08	2.95	1.03	0	0	IS
1.93	3.4	1.85	0	0	IS
2.21	3.18	1.67	0	0	IS
2.22	2.74	0.82	0	0	IS
2.36	3.26	1.19	0	0	IS
2.09	3.57	2.79	0	0	IS
2.14	3.06	1.6	0	0	IS
2.28	3.26	1.41	0	0	IS
2.33	2.74	0.62	0	0	IS
2.11	3.14	1.64	0	0	IS
2.47	3.17	1.07	0	0	IS
2.27	3.11	1.26	0	0	IS

2.47	3.05	0.83	0	0	IS
2.31	3.05	1.1	0	0	IS
2.32	3	1.13	0	0	IS
2.06	2.95	1.23	0	0	IS
2.21	2.99	1.04	0	0	IS
2.27	2.96	1.02	0	0	IS
2.25	2.95	1.03	0	0	IS
1.98	2.77	1.26	0	0	BS
2.13	2.85	1.05	0	0	IS
2.58	2.94	0.61	0	0	IS
2	3.02	1.52	0	0	IS
2.04	2.91	1.4	0	0	IS
2.11	2.86	1.27	0	0	IS
2.08	2.83	1.14	0	0	IS
2.69	4.07	2.01	0	0	IS
2.5	3.79	1.83	0	0	IS
2.29	3.64	1.95	0	0	IS
2.41	3.33	1.21	0	0	IS
2.18	3.21	1.54	0	0	IS
2.31	3.4	1.63	0	0	IS
2.25	3.1	1.22	0	0	IS
2.5	3.2	1.13	0	0	IS
2.07	2.8	1.26	0	0	IS
2.44	3.21	1.39	0	0	IS
2.19	2.86	0.88	0	0	IS
2.17	3.03	1.13	0	0	BS
2.33	2.9	0.89	0	0	IS
1.93	2.91	1.5	0	0	IS
2.08	3.17	1.82	0	0	IS
1.85	2.57	1.56	0	0	IS
2.19	2.85	0.89	0	0	BS
2.08	2.54	0.81	0	0	IS
2.3	2.74	0.58	0	0	IS
2.23	2.82	0.83	0	0	IS
2.1	2.75	0.98	0	0	IS
2.36	3.02	1.01	0	0	IS
2.08	2.74	0.93	0	0	IS
2.2	3	1.08	0	0	IS
2.22	2.64	0.58	0	0	IS
2.11	2.83	1.17	0	0	IS
1.89	2.91	1.74	0	0	IS
1.89	2.93	1.35	0	0	IS
1.85	2.76	1.26	0	0	IS
2.62	3.3	1.21	0	0	IS
1.96	2.69	1.23	0	0	BS
2.26	3.15	1.28	0	0	IS
2.11	2.66	1.14	0	0	IS
2.02	2.98	1.32	0	0	IS
1.61	1.98	0.54	0	0	IS
2.03	2.52	0.73	0	0	IS
2.22	2.83	0.99	0	0	IS
2.06	2.33	0.51	0	0	IS
1.78	2.24	0.68	0	0	IS
2.26	2.8	0.76	0	0	IS

1.66	2.63	1.82	0	0	IS
1.77	2.29	0.88	0	0	IS
2.11	3.2	1.38	0	0	IS
1.61	2.46	1.16	0	0	IS
1.56	1.96	1.07	0	0	IS
2.47	3.34	1.31	0	0	IS
2.32	2.67	0.58	0	0	IS
2.41	3.08	1.26	0	0	IS
2.23	2.75	0.74	0	0	IS
2.13	2.73	0.81	0	0	IS
1.88	2.92	1.51	0	0	IS
2.04	3.19	1.54	0	0	IS
2.1	2.7	0.98	0	0	IS
1.96	2.32	0.57	0	0	IS
2.26	2.6	0.49	0	0	IS
2.05	2.5	0.73	0	0	IS
2.13	2.29	0.31	0	0	IS
2.54	3.17	1.1	0	0	IS
1.89	2.22	0.54	0	0	IS
1.94	2.43	0.81	0	0	IS
2.19	2.43	0.55	0	0	IS
2.23	2.43	0.52	0	0	IS
1.88	2.1	0.36	0	0	IS
2.28	2.76	0.8	0	0	IS
1.88	2.09	0.53	0	0	IS
1.58	1.95	0.72	0	0	IS
1.95	2.35	0.57	0	0	IS
1.73	2.18	0.79	0	0	IS
1.83	2.21	0.65	0	0	IS
1.65	1.94	0.54	0	0	IS
1.82	2.19	0.54	0	0	IS
1.98	2.15	0.39	0	0	IS
2.09	2.24	0.46	0	0	IS
2.22	2.5	0.51	0	0	IS
2.06	2.5	0.76	0	0	IS
1.68	2.42	1.04	0	0	IS
1.94	2.4	0.81	0	0	IS
2.29	2.78	0.67	0	0	IS
2.12	2.64	0.76	0	0	IS
2.13	2.41	0.56	0	0	IS
2.52	3.33	1.57	0	0	IS
2.67	3.54	1.66	0	0	IS
2.38	3.33	1.57	0	0	IS
2.1	2.64	0.79	0	0	IS
2.17	2.69	0.81	0	0	IS
2.25	2.85	0.85	0	0	IS
2.42	3.01	1.2	0	0	IS
2.1	2.77	0.88	0	0	IS
2.02	2.69	1.39	0	0	IS
2	2.94	1.12	0	0	BS
2.45	2.99	0.87	0	0	BS
2.21	3.07	1.15	0	0	IS
3.16	4.86	3.07	0	0	IS
3.12	4.49	2.38	0	0	IS

2.66	4.54	2.27	0	0	IS
2.13	3.02	1.22	0	0	IS
2.9	4.59	2.85	0	0	IS
3.01	4.1	2.02	0	0	IS
2.74	5.09	2.94	0	0	IS
3.17	4.14	1.95	0	0	IS
3.42	4.24	1.84	0	0	IS
3.29	4.92	2.82	0	0	IS
2.13	3.05	1.33	0	0	IS
3.5	4.87	2.68	0	0	IS
3.57	5.02	2.52	0	0	IS
3.33	4.95	3.05	0	0	IS
3.65	4.93	2.84	0	0	IS
1.94	2.7	1.05	0	0	IS
2.51	3.77	1.85	0	0	IS
2.32	3.99	2.01	0	0	IS
2.95	3.97	2.15	0	0	IS
1.92	2.67	1.03	0	0	IS
2.84	3.8	1.78	0	0	IS
2.5	3.77	1.9	0	0	IS
2.71	3.85	1.91	0	0	IS
2.72	3.99	1.73	0	0	IS
2.13	2.88	1.28	0	0	IS
1.67	2.23	1.2	0	0	PL
2.8	4.07	1.81	0	0	IS
2.45	4.01	2.02	0	0	IS
2.94	3.82	1.32	0	0	IS
2.28	4.68	3.2	0	0	IS
3.14	4.29	2.55	0	0	IS
2.81	4.45	2.63	0	0	IS
2.53	4.29	2.23	0	0	IS
2.61	4.29	2.43	0	0	IS
2.64	3.69	2.21	0	0	IS
3.57	4.57	2.12	0	0	IS
3.09	4.35	2.3	0	0	IS
3.06	4.49	2.63	0	0	IS
3.09	3.94	2.2	0	0	IS
2.45	3.32	1.46	0	0	IS
3.06	4.15	1.83	0	0	IS
2.24	2.86	0.87	0	0	IS
2.78	4.12	2.17	0	0	IS
2.86	4.39	2.62	0	0	IS
2.66	4.41	2.56	0	0	IS
2.48	4.29	2.4	0	0	BS
2.75	4.34	2.08	0	0	IS
2.58	3.48	1.81	0	0	IS
3.13	4.11	1.66	0	0	IS
3.36	4.55	2.69	0	0	BS
2.88	4.69	2.45	0	0	IS
3.04	4.29	2.41	0	0	IS
2.63	4.48	2.48	0	0	IS
3.08	4.31	2.17	0	0	IS
2.32	3.91	2.11	0	0	IS
2.49	3.86	1.85	0	0	IS

2.59	3.77	1.75	0	0	IS
2.77	3.5	1.45	0	0	IS
2.03	2.77	1.25	0	0	BS
2.37	3.53	1.67	0	0	IS
1.98	3.57	2.02	0	0	IS
2.05	3.27	1.89	0	0	IS
2.48	3.33	1.57	0	0	IS
2.22	3.24	1.54	0	0	IS
2.04	3.54	2.23	0	0	IS
2.08	3.17	1.92	0	0	IS
2.53	3.53	1.32	0	0	IS
2.65	3.99	2.03	0	0	IS
2.04	3.73	2.31	0	0	IS
2.55	3.25	1.03	0	0	IS
2.27	3.65	2.24	0	0	IS
1.95	3.63	2.41	0	0	IS
2.27	3.55	1.88	0	0	IS
2.98	4.01	2	0	0	IS
2.21	3.57	2.17	0	0	IS
2.35	3.74	1.84	0	0	BS
2.63	3.71	1.56	0	0	IS
2.56	3.53	1.46	0	0	IS
3.09	3.9	2.31	0	0	IS
3.16	4.04	2.07	0	0	IS
3.36	3.96	1.87	0	0	IS
2.39	3.54	1.69	0	0	IS
2.71	3.97	1.8	0	0	IS
2.86	4.03	1.98	0	0	IS
2.79	4.08	2.01	0	0	IS
2.44	3.25	1.35	0	0	IS
2.51	3.6	1.67	0	0	IS
2.87	3.72	1.5	0	0	IS
2.68	4.01	2.21	0	0	IS
3.21	4.08	2.09	0	0	IS
2.35	3.85	1.7	0	0	IS
2.89	3.63	1.33	0	0	IS
2.43	3.47	1.8	0	0	IS
2.66	3.63	1.38	0	0	IS
3.36	4.03	1.51	0	0	IS
2.63	3.45	1.28	0	0	BS
2.65	4.04	2.26	0	0	IS
2.61	3.78	1.95	0	0	IS
2.69	4.12	2.1	0	0	IS
2.56	3.37	1.7	0	0	IS
2.56	4.04	2.18	0	0	IS
2.91	4.36	2.26	0	0	IS
2.53	3.78	1.87	0	0	IS
2.56	4.04	2.3	0	0	IS
2.67	3.95	1.99	0	0	IS
2.59	3.79	1.8	0	0	IS
2.57	3.96	1.82	0	0	IS
2.32	3.85	2	0	0	IS
2.17	3.29	1.53	0	0	IS
2.3	3.45	1.55	0	0	IS

2.14	3.41	1.72	0	0	IS
3.57	4.57	2.12	0	0	IS
2.24	3.39	1.47	0	0	IS
2.22	3.37	1.56	0	0	IS
2.41	3.23	1.17	0	0	IS
2.62	4.16	2.33	0	0	IS
2.19	3.39	1.54	0	0	IS
2.19	2.83	1.59	0	0	IS
2.29	3.21	1.29	0	0	IS
2.46	3.16	1.44	0	0	IS
2.4	3.81	1.96	0	0	IS
2.76	3.77	1.76	0	0	IS
2.75	3.85	1.63	0	0	IS
3.28	3.8	1.53	0	0	IS
2.75	3.7	1.5	0	0	IS
2.53	3.91	1.83	0	0	BS
2.61	3.62	1.3	0	0	IS
3.38	4.38	2.51	0	0	IS
2.42	3.48	1.48	0	0	IS
2.64	3.59	1.39	0	0	IS
3.4	4.91	2.67	0	0	IS
3.41	4.66	2.71	0	0	IS
2.31	3.52	1.59	0	0	IS
2.72	3.65	1.64	0	0	IS
2.58	3.76	1.6	0	0	IS
2.55	3.54	1.36	0	0	IS
2.57	3.53	1.27	0	0	IS
2.35	3.49	1.52	0	0	IS
2.54	3.47	1.26	0	0	IS
2.45	3.43	1.27	0	0	IS
2.28	3.6	1.76	0	0	IS
2.52	3.1	1.48	0	0	IS
2.39	3.82	2.09	0	0	IS
0.47	0.62	0.32	195	34.82	IS
2.59	3.27	1.28	0	0	IS
2.59	3.27	1.28	0	0	IS
2.44	3.48	1.53	0	0	IS
2.35	3.43	1.75	0	0	IS
0.65	0.73	0.2	27	71.05	IS
0.64	0.72	0.27	30	51.02	IS
0.47	0.61	0.31	56	33.61	IS
0.5	0.61	0.23	0	0	IS
0.46	0.61	0.3	0	0	IS
2.34	3.34	1.93	0	0	IS
0.61	0.79	0.52	0	0	IS
2.65	4	2.18	0	0	IS
0.52	1.03	0.79	0	0	IS
0.52	0.68	0.4	0	0	IS
0.55	0.66	0.29	0	0	IS
0.57	0.73	0.35	0	0	IS
0.56	0.8	0.38	0	0	IS
0.76	0.88	0.28	0	0	IS
2.66	3.88	2.37	0	0	IS
0.69	0.94	0.47	0	0	IS

0.78	0.87	0.25	0	0	IS
0.69	0.86	0.34	0	0	IS
0.71	0.98	0.41	0	0	IS
0.59	0.83	0.41	0	0	IS
0.66	0.8	0.47	0	0	IS
2.48	4.16	2.12	0	0	BS
2.62	3.4	1.61	0	0	IS
2.33	3.16	1.68	0	0	IS
0.64	0.97	0.59	0	0	IS
0.64	0.94	0.5	0	0	IS
0.56	0.65	0.26	0	0	IS
0.61	0.72	0.25	0	0	IS
0.54	0.7	0.34	0	0	IS
0.6	0.8	0.43	0	0	IS
0.59	0.7	0.26	0	0	IS
0.38	0.69	0.45	0	0	IS
0.57	0.85	0.5	0	0	IS
0.53	0.65	0.3	0	0	IS
0.51	0.66	0.32	0	0	IS
0.53	0.68	0.32	0	0	IS
0.75	0.86	0.32	0	0	IS
0.62	0.72	0.24	0	0	IS
0.39	0.53	0.21	0	0	IS
0.29	0.42	0.18	0	0	IS
0.35	0.53	0.35	0	0	IS
0.38	0.55	0.3	0	0	IS
0.34	0.63	0.41	0	0	IS
0.28	0.67	0.5	0	0	IS
0.34	0.58	0.37	0	0	IS
0.38	0.68	0.42	0	0	IS
0.42	0.57	0.33	0	0	IS
0.43	0.55	0.26	0	0	IS
0.47	0.59	0.26	0	0	IS
0.28	0.47	0.26	0	0	IS
2.39	3.17	1.1	0	0	IS
0.29	0.51	0.31	0	0	IS
0.36	0.57	0.38	0	0	IS
0.49	0.67	0.34	0	0	IS
0.5	0.66	0.33	0	0	IS
0.38	0.62	0.39	0	0	IS
0.52	0.67	0.36	0	0	IS
0.51	0.67	0.31	0	0	IS
0.53	0.62	0.34	0	0	IS
0.53	0.66	0.43	0	0	IS
0.5	0.75	0.43	0	0	IS
0.57	0.69	0.31	0	0	IS
0.5	0.66	0.43	0	0	IS
2.46	3.45	1.48	0	0	IS
2.51	3.45	1.43	0	0	IS
0.58	0.76	0.51	0	0	IS
0.48	0.62	0.3	0	0	IS
0.44	0.59	0.29	0	0	IS
0.53	0.58	0.22	0	0	IS
0.47	0.71	0.47	0	0	IS

0.47	0.72	0.5	0	0	IS
0.43	0.62	0.32	0	0	IS
0.47	0.68	0.4	0	0	IS
0.25	0.33	0.14	246	57.41	PL
0.55	0.67	0.36	0	0	IS
0.31	0.47	0.26	253	71.21	PL
0.34	0.45	0.17	216	66.16	PL
0.2	0.29	0.16	164	58.66	PL
0.39	0.52	0.25	189	61.97	PL
0.21	0.3	0.14	178	58.61	PL
0.16	0.21	0.1	134	43.18	OH
0.26	0.38	0.21	165	46.73	PL
0.25	0.38	0.22	162	51.48	PL
0.39	0.47	0.16	207	38.72	OH
0.31	0.4	0.2	185	53.97	PL
0.32	0.37	0.11	173	56.39	HC
0.33	0.44	0.19	186	40.23	OH
0.29	0.43	0.24	150	57.25	PL
0.12	0.17	0.09	107	25.75	OH
0.16	0.25	0.16	95	44	PL
0.23	0.3	0.13	113	42.24	PL
0.2	0.31	0.18	99	48.86	PL
0.12	0.18	0.1	76	39.71	PL
0.21	0.27	0.11	89	36.81	OH
0.22	0.34	0.19	76	45.13	PL
0.29	0.38	0.18	79	50.16	PL
0.13	0.19	0.11	65	26.06	OH
0.14	0.22	0.12	50	28.51	OH
0.09	0.13	0.07	50	24.21	OH
0.11	0.16	0.09	106	22.92	OH
0.12	0.16	0.07	90	24.87	OH
0.14	0.19	0.11	71	27.12	OH
0.13	0.19	0.09	77	25.81	OH
0.36	0.45	0.19	130	38.21	OH
0.36	0.42	0.16	117	52.14	PL
0.24	0.41	0.25	127	51.69	PL
0.41	0.6	0.4	105	64.66	PL
0.31	0.56	0.37	113	74.1	PL
0.42	0.48	0.2	143	61.06	PL
0.31	0.39	0.17	145	56.25	PL
0.24	0.46	0.3	133	55.79	PL
0.3	0.67	0.48	144	42.13	OH
0.29	0.4	0.2	148	41.74	OH
0.31	0.43	0.21	128	64.1	PL
0.3	0.38	0.2	104	59.87	PL
0.2	0.3	0.15	97	58.43	PL
0.2	0.29	0.14	96	58.01	PL
0.32	0.46	0.23	103	60.16	PL
0.38	0.48	0.2	93	74.22	PL
0.14	0.18	0.06	101	66.89	PL
0.22	0.32	0.16	108	61.22	PL
0.23	0.32	0.18	120	43.94	PL
0.18	0.25	0.13	101	61.14	PL
0.27	0.34	0.15	112	41.95	OH

0.3	0.42	0.23	102	55.8	PL
0.38	0.45	0.17	112	37.5	OH
0.24	0.32	0.16	93	53.54	PL
0.37	0.46	0.2	104	39.02	OH
0.16	0.23	0.12	99	43.19	PL
0.22	0.3	0.15	86	48.92	PL
0.27	0.34	0.14	81	53.32	PL
0.19	0.32	0.18	85	62.82	PL
0.19	0.25	0.11	87	59.18	PL
0.32	0.39	0.15	90	64.7	PL
0.21	0.3	0.15	96	48.24	PL
0.2	0.26	0.09	88	61.11111	PL
0.18	0.26	0.13	18	33.58	OH
0.26	0.38	0.2	16	41.45	PL
0.21	0.29	0.15	29	35.37	OH
0.2	0.26	0.13	18	36.36	PL
0.27	0.38	0.2	31	42.18	PL
0.28	0.39	0.21	32	38.51	OH
0.21	0.34	0.19	29	38.67	PL
0.28	0.5	0.32	9	50.85	OC
0.13	0.21	0.12	18	42.76	OC
0.21	0.27	0.13	17	40.96	PL
0.2	0.59	0.47	12	31.91	OH
0.16	0.24	0.13	10	34.97	OH
0.22	0.29	0.14	7	37.04	OH
0.2	0.28	0.17	11	29.1	OH
0.09	0.16	0.1	12	29.2	OH
0.15	0.21	0.12	16	28.07	OH
0.13	0.37	0.3	14	25.09	OH
0.17	0.23	0.1	44	33.33	OH
0.12	0.2	0.12	42	23.49	OC
0.1	0.14	0.06	37	25.45	OH
0.26	0.31	0.12	29	34.48	OH
0.15	0.19	0.07	40	32.15	OH
0.13	0.3	0.21	28	25.69	OH
0.12	0.18	0.11	34	27.44	OH
0.12	0.18	0.1	32	26.73	OH
0.17	0.25	0.14	34	27.49	OH
0.15	0.28	0.19	31	25.7	OH
0.13	0.2	0.11	27	27.03	OH
0.11	0.2	0.13	35	25.64	OH
0.12	0.2	0.12	39	26.77	OH
0.17	0.27	0.15	43	41.19	OH
0.15	0.21	0.11	30	40.38	OH
0.11	0.19	0.12	46	27.33	OH
0.29	0.34	0.14	29	41.79	PL
0.19	0.26	0.14	35	41.32	PL
0.16	0.35	0.25	35	40.32	OC
0.19	0.34	0.22	41	56.87	PL
0.19	0.25	0.11	55	51.94	PL
0.19	0.32	0.17	59	55.66	PL
0.18	0.21	0.07	60	48.74	PL
0.22	0.42	0.27	62	48.32	OH
0.26	0.3	0.11	61	54.27	PL

0.14	0.29	0.18	40	61.35	PL
0.23	0.28	0.12	40	55.02	PL
0.34	0.49	0.26	41	53.04	PL
0.2	0.31	0.16	43	53.82	PL
0.22	0.32	0.16	50	48.64	PL
0.23	0.35	0.2	48	44.57	PL
0.21	0.43	0.27	39	50.85	PL
0.24	0.35	0.34	44	50.69	PL
0.27	0.5	0.32	45	49.61	OH
0.25	0.38	0.42	47	50	PL
0.46	0.54	0.17	32	47.2	OC
0.45	0.66	0.31	32	50.24	OC
0.23	0.35	0.2	36	47.12	PL
0.23	0.4	0.25	29	55.98	PL
0.17	0.21	0.06	28	50.27	PL
0.29	0.6	0.41	24	52.86	PL
0.25	0.32	0.14	17	55.02	PL
0.41	0.66	0.41	0	0	IS
0.41	0.48	0.16	24	69.36	OC
0.36	0.42	0.12	52	49.81	HC
1.29	2.02	1.29	53	44.17	OC
0.63	1	0.76	44	42.23	OC
0.52	1.11	0.83	42	47.89	OC
0.59	0.8	0.38	0	0	IS
0.57	0.78	0.45	0	0	IS
1.27	1.66	1.24	92	61.01	BS
1.79	2.39	1.4	75	46.32	OC
1.72	2.96	1.8	64	68.97	PL
0.22	0.27	0.1	49	48.56	PL
0.27	0.32	0.11	57	46.84	PL
0.23	0.31	0.15	56	37.74	OH
0.25	0.34	0.2	48	44	PL
0.2	0.34	0.2	58	33.6	OH
0.17	0.24	0.12	61	34.39	OH
0.14	0.19	0.09	85	34.04	OH
0.24	0.34	0.18	76	37.68	OH
0.3	0.43	0.23	72	43.61	PL
0.2	0.27	0.13	56	43.38	PL
0.13	0.21	0.12	72	34.77	OH
0.32	0.4	0.18	16	61.68	PL
0.24	0.49	0.34	65	34.87	OH
0.15	0.21	0.11	54	38.24	OH
0.24	0.36	0.19	49	65.95	PL
0.17	0.29	0.16	40	74.35	PL
0.2	0.38	0.23	68	53.88	PL
0.22	0.34	0.18	56	61.14	PL
0.3	0.38	0.16	75	61.98	PL
0.3	0.41	0.24	56	68.46	PL
0.25	0.35	0.16	65	62.62	PL
0.34	0.46	0.25	67	59.93	OC
0.32	0.44	0.21	75	60.58	PL
0.26	0.35	0.15	74	60.11	PL
0.2	0.27	0.13	81	47.99	PL
0.36	0.45	0.19	94	39.15	OH

0.25	0.35	0.16	70	57.61	PL
0.24	0.35	0.18	76	54.32	PL
0.33	0.5	0.29	93	45.97	OH
0.25	0.39	0.22	93	45.01	PL
0.29	1.19	1.02	74	43.56	OH
0.35	0.58	0.35	88	39.34	PL
0.38	0.44	0.12	68	48.12	HC
0.35	0.4	0.16	69	44.15	OH
0.33	0.42	0.2	56	54.42	PL
0.24	0.32	0.16	74	40.64	OH
0.18	0.27	0.13	84	41.36	PL
0.3	0.4	0.21	70	51.17	PL
1.51	3.45	2.96	4	18.52	HC
1.93	3.08	2.18	5	22.22	HC
1.4	2.86	2.2	4	18.1	HC
1.55	3.13	2.11	3	25.64	HC
0.96	2.98	2.67	4	22.86	HC
0.65	2.82	2.58	4	19.42	HC
1.21	2.78	2.45	4	18.35	HC
2.16	4.36	3.47	5	14.75	HC
2.6	4.66	3.93	3	20.27	HC
2.34	4.5	3.55	3	20.83	HC
2.39	4.35	3.38	3	21.58	PL
1.81	3.86	3.26	3	21.74	HC
2.58	3.91	2.62	3	24.39	HC
2.2	3.73	2.58	4	17.24	HC
2.07	3.41	2.46	4	17.47	HC
1.2	1.91	1.21	5	17.48	HC
1.15	2.49	2.08	4	17.32	HC
1.37	2.29	1.7	3	20	HC
1.19	2.41	1.92	3	21.74	HC
0.97	2.41	2	4	16.95	HC
0.89	2.18	1.87	4	18.35	HC
0.83	2.11	1.73	3	23.62	HC
0.7	2.29	2.1	3	21.28	HC
0.84	2.29	1.92	3	22.39	HC
0.61	2.19	1.9	5	17.42	HC
0.91	1.82	1.44	4	19.14	HC
0.77	1.84	1.55	5	18.12	HC
1.03	1.97	1.54	4	18.18	HC
0.87	2.08	1.63	5	16.72	HC
1.04	1.94	1.53	4	19.23	HC
0.77	1.92	1.47	3	21.43	HC
1.34	2.06	1.36	4	23.26	HC
0.99	1.97	1.52	4	19.23	HC
0.96	1.91	1.45	3	22.9	HC
0.74	1.72	1.37	6	15.11	HC
1.11	1.78	1.14	5	17.99	HC
0.73	1.72	1.3	4	18.78	HC
0.85	1.69	1.35	4	19.42	HC
0.92	1.78	1.37	4	20.62	HC
0.75	1.73	1.39	5	17.86	HC
0.85	1.65	1.22	5	17.86	HC
0.76	1.89	1.52	3	20.69	HC

1.16	1.82	1.18	3	21.13	HC
0.92	1.92	1.36	4	17.09	HC
1.22	1.9	1.25	4	19.42	HC
1.09	1.78	1.26	6	17.19	HC
0.93	1.82	1.3	4	18.96	HC
1.04	1.77	1.26	4	14.65	HC
1.18	1.93	1.28	4	16.74	HC
0.65	1.21	0.88	4	19.23	IS
0.82	1.4	0.96	4	19.14	HC
0.61	1.56	1.27	5	16.13	HC
0.71	1.36	0.98	4	18.78	HC
0.8	1.6	1.12	3	21.28	HC
0.76	1.51	1.13	4	18.43	HC
0.7	1.54	1.13	4	19.61	HC
0.74	1.36	1.01	3	22.56	HC
0.59	1.47	1.19	4	17.86	HC
0.75	1.48	1.09	4	19.51	HC
0.79	1.37	0.93	4	20	HC
0.74	1.17	0.73	6	18.35	CC
0.76	1.26	0.81	4	17.94	HC
0.7	1.32	0.98	5	16.61	HC
0.79	1.31	0.89	5	18.52	HC
0.53	1.34	1	5	17.42	HC
0.81	1.37	0.95	4	19.32	HC
0.57	1.51	1.3	8	19.23	HC
0.74	1.57	1.2	6	16.95	HC
0.7	1.48	1.1	5	16.84	HC
0.87	1.58	1.02	4	21.86	HC
0.76	1.37	0.96	3	22.06	HC
0.66	1.61	1.32	4	17.62	HC
0.79	1.51	1.25	4	18.26	HC
0.9	1.36	0.92	5	18.18	IS
0.77	1.46	1.08	4	18.1	HC
0.82	1.55	1.15	3	20.98	HC
0.58	1.5	1.26	3	22.06	HC
0.41	1.56	1.36	3	19.61	HC
0.63	1.5	1.2	4	19.14	HC
0.58	1.46	1.25	4	22.6	HC
0.56	1.34	1.1	3	21.13	HC
0.63	1.36	1.09	4	18.96	HC
0.68	1.44	1.13	4	19.14	HC
0.53	1.44	1.2	4	18.69	PL
0.66	1.41	1.19	4	20.62	HC
0.76	1.6	1.32	4	17.24	HC
0.83	1.78	1.34	5	17.92	HC
0.89	1.7	1.25	5	16.13	HC
0.82	1.61	1.23	4	17.32	HC
0.45	1.93	1.71	3	21.74	HC
0.58	1.75	1.49	3	21.9	HC
0.88	1.64	1.32	3	20.98	HC
0.62	1.73	1.48	4	19.51	HC
0.65	1.79	1.46	4	18.26	HC
0.83	1.81	1.55	4	18.1	PL
0.76	1.57	1.21	3	20.13	HC

0.65	1.04	0.79	6	15.15	HC
0.5	1.05	0.82	6	17.19	HC
0.65	1.07	0.75	7	16.59	HC
0.46	1.16	0.93	5	17.42	HC
0.38	1.2	1.01	4	18.26	HC
0.56	1.21	0.93	5	14.2	HC
0.6	1.16	0.84	5	14.58	HC
0.47	1.04	0.77	4	18.02	HC
0.47	0.93	0.72	4	18.69	HC
0.65	1	0.73	4	16.95	IS
0.45	1.18	0.98	5	17.54	IS
0.45	1.12	0.92	5	16.45	HC
0.47	0.93	0.66	6	16.95	HC
0.57	1.02	0.73	4	18.6	HC
0.57	1.02	0.73	5	17.06	HC
0.53	1.01	0.8	3	20.13	HC
0.62	0.97	0.7	3	20.83	HC
0.43	1.1	0.85	3	18.4	HC
0.5	1.08	0.86	5	18.05	HC
0.6	0.99	0.73	4	18.69	HC
0.47	1.12	0.87	4	17.94	CC
0.53	1.1	0.87	4	19.32	HC
0.45	1.44	1.17	3	20.69	HC
0.43	1.31	1.12	3	21.43	HC
0.35	1.33	1.12	3	21.74	HC
0.48	1.18	0.92	3	24.59	HC
0.44	1.08	0.82	3	21.74	HC
0.49	1.03	0.75	3	22.06	HC
0.74	1.13	0.82	5	18.59	HC
0.41	1.24	1.04	4	19.7	HC
0.61	1.21	0.95	5	20.58	HC
0.65	1.38	1.11	5	19.31	IS
0.82	1.33	0.97	5	17.86	HC
0.63	1.22	0.89	4	18.87	HC
0.64	1.18	0.73	3	22.39	HC
0.65	1.18	0.89	4	20.73	HC
0.41	1.28	1.04	5	15.92	HC
0.69	1.23	0.94	4	18.35	HC
0.65	1.13	0.84	4	18.78	HC
0.54	1.28	0.9	4	18.87	HC
0.66	1.15	0.85	6	16.17	HC
0.57	1.25	0.95	5	18.87	HC
0.61	1.14	0.88	6	18.35	CC
0.54	1.32	1.03	4	15.21	HC
0.34	0.46	1.16	3	12.3	CC
0.64	1.28	0.99	4	20	HC
0.65	1.3	0.95	4	19.32	HC
0.59	1.26	0.97	5	17.61	HC
0.53	1.35	1.09	4	18.69	HC
0.49	1.21	0.96	4	18.78	HC
0.37	1.1	0.9	3	18.87	HC
0.35	1.31	1.09	4	18.96	HC
0.35	0.75	0.52	7	16.17	HC
0.29	0.63	0.45	6	18.81	HC

0.35	0.62	0.38	4	19.05	HC
0.4	0.68	0.43	5	18.32	HC
0.45	0.72	0.51	4	18.43	HC
0.3	0.92	0.74	5	18.18	IS
0.53	0.79	0.57	6	19.54	HC
0.37	0.85	0.68	6	19.05	PL
0.34	0.73	0.53	6	20.83	HC
0.29	0.73	0.56	7	20.35	HC
0.46	0.76	0.55	4	18.43	HC
0.4	0.75	0.54	3	21.9	HC
0.32	0.75	0.54	3	22.22	HC
0.48	0.73	0.47	4	21.74	HC
0.33	0.91	0.74	5	21.65	HC
0.43	0.8	0.58	4	21.51	HC
0.45	0.8	0.56	5	21.93	HC
0.31	0.99	0.8	3	20.55	HC
0.26	0.97	0.8	4	20.2	HC
0.51	0.92	0.67	3	21.74	HC
0.37	0.89	0.68	4	16.53	HC
0.31	1	0.79	4	17.86	HC
0.34	0.87	0.69	4	18.6	IS
0.35	0.77	0.54	4	18.78	HC
0.36	0.8	0.61	5	17.73	HC
0.45	0.83	0.52	4	19.14	HC
0.41	0.78	0.52	5	17.79	HC
0.34	0.81	0.58	4	20.41	HC
0.38	0.8	0.57	4	19.14	HC
0.45	0.94	0.68	4	22.22	HC
0.41	0.86	0.63	6	21.35	HC
0.43	0.91	0.66	7	19.66	HC
0.41	0.89	0.65	6	18.35	CC
0.41	0.83	0.59	5	19.92	HC
0.33	0.84	0.66	6	19.8	HC
0.69	1.12	0.72	4	17.78	HC
0.55	1.09	0.73	4	18.26	HC
0.63	1.11	0.78	5	18.66	HC
0.67	1.19	0.88	6	19.74	HC
0.38	1.16	0.87	4	19.23	PL
0.82	1.19	0.66	3	24	PL
0.55	1.01	0.76	3	20.55	HC
0.44	0.86	0.63	4	19.7	HC
0.31	0.94	0.71	4	21.05	HC
0.53	0.97	0.6	3	21.28	HC
0.61	0.88	0.59	3	21.58	HC
0.42	0.76	0.48	4	20	HC
0.44	0.88	0.61	5	17.54	HC
0.41	0.84	0.52	3	19.74	HC
0.55	1	0.7	3	21.43	HC
0.49	0.91	0.52	4	18.18	OH
0.51	0.86	0.47	4	18.52	HC
0.5	0.82	0.49	7	15.22	HC
0.33	0.9	0.71	5	17.92	HC
0.48	1.14	0.88	6	16.39	HC
0.58	1.07	0.7	6	15.63	HC

0.47	0.96	0.73	4	19.14	HC
0.38	0.85	0.64	4	19.42	HC
0.5	0.86	0.54	4	19.7	HC
0.42	0.95	0.72	5	16.72	HC
0.47	0.82	0.57	4	18.02	HC
0.46	0.89	0.64	6	17.91	HC
0.41	0.99	0.77	6	17.96	CC
0.56	0.98	0.66	5	17.73	HC
0.52	1.01	0.75	5	17.67	HC
0.45	1	0.72	4	15.44	HC
0.55	0.99	0.69	5	17.12	HC
0.52	0.98	0.65	4	17.17	HC
0.34	0.99	0.78	4	17.24	HC
0.42	1.02	0.81	4	17.54	HC
0.54	0.97	0.76	4	18.84	HC
0.4	1.06	0.8	4	18.52	HC
0.43	1.05	0.86	4	18.35	PL
0.52	1.05	0.79	5	18.38	HC
0.43	1.02	0.79	5	17.86	HC
0.48	1.02	0.81	6	19.74	HC
0.36	1.19	0.98	4	19.23	HC
0.49	0.94	0.7	5	17.67	HC
0.27	0.36	0.16	5	17.01	HC
0.2	0.27	0.11	4	17.32	HC
0.23	0.32	0.14	5	17.3	HC
0.31	0.49	0.32	5	18.87	HC
0.26	0.51	0.32	6	16.35	HC
0.3	0.43	0.22	5	15.38	HC
0.23	0.36	0.18	5	17.79	CC
0.28	0.41	0.21	6	17.6	CC
0.27	0.56	0.37	6	16.22	HC
0.33	0.68	0.48	6	17.49	HC
0.3	0.62	0.41	7	14.77	HC
0.39	0.62	0.35	6	15.92	HC
0.34	0.5	0.25	6	17	HC
0.33	0.59	0.37	6	19.8	HC
0.36	0.65	0.45	4	19.42	HC
0.32	0.49	0.28	5	19.69	HC
0.3	0.48	0.27	7	18.67	HC
0.33	0.48	0.25	6	19.74	CC
0.28	0.53	0.34	7	19.23	HC
0.37	0.58	0.3	4	14.87	HC
0.36	0.51	0.25	5	14.04	PL
0.27	0.57	0.4	5	17.36	IS
0.28	0.54	0.35	6	16.76	CC
0.28	0.48	0.25	4	16.33	HC
0.29	0.56	0.35	4	17.32	HC
0.28	0.39	0.18	4	17.32	HC
0.26	0.45	0.27	5	17.42	HC
0.23	0.36	0.21	4	17.94	CC
0.2	0.36	0.23	4	18.6	HC
0.27	0.54	0.36	4	19.42	HC
0.29	0.48	0.28	5	17.73	PL
0.25	0.43	0.27	5	20.49	HC

0.22	0.31	0.16	4	21.86	HC
0.2	0.31	0.18	6	18.24	HC
0.31	0.41	0.18	5	17.48	HC
0.25	0.5	0.33	4	16.88	HC
0.15	0.36	0.27	4	18.18	HC
0.24	0.37	0.2	4	17.24	HC
0.33	0.6	0.37	6	21.13	HC
0.29	0.55	0.36	7	19.77	HC
0.27	0.56	0.36	8	20.3	HC
0.28	0.59	0.41	4	19.8	HC
0.22	0.57	0.41	4	17.32	HC
0.3	0.46	0.25	4	19.32	HC
0.28	0.49	0.34	5	18.18	HC
0.32	0.58	0.38	6	17.75	CC
0.29	0.46	0.24	6	18.35	HC
0.17	0.28	0.15	3	22.22	HC
0.25	0.38	0.2	4	23.26	HC
0.28	0.37	0.17	4	24.1	HC
0.21	0.34	0.17	3	23.26	PL
0.26	0.41	0.22	3	22.39	HC
0.28	0.38	0.16	5	18.87	CC
0.25	0.4	0.23	4	20.41	HC
0.3	0.4	0.17	5	19.69	HC
0.3	0.43	0.21	5	20.66	HC
0.26	0.41	0.24	5	21.46	HC
0.28	0.33	0.1	5	21.74	HC
0.24	0.4	0.24	4	20.1	HC
0.26	0.46	0.27	3	22.22	HC
0.23	0.34	0.18	4	17.62	HC
0.25	0.33	0.14	4	18.18	HC
0.21	0.34	0.18	4	18.35	HC
0.22	0.4	0.24	3	20.27	HC
0.25	0.38	0.22	4	18.87	HC
0.25	0.49	0.29	4	18.35	HC
0.2	0.32	0.17	4	18.26	HC
0.32	0.64	0.42	4	24.1	HC
0.28	0.61	0.41	4	22.86	HC
0.29	0.65	0.49	3	21.28	HC
0.27	0.43	0.23	4	22.6	HC
0.33	0.44	0.18	3	22.73	HC
0.25	0.56	0.38	5	20.08	HC
0.37	0.68	0.43	4	19.05	HC
0.38	0.67	0.43	5	17.86	CC
0.34	0.58	0.4	3	20	HC
0.32	0.69	0.5	3	20.27	HC
0.23	0.6	0.43	4	19.7	HC
0.37	0.58	0.37	4	20.51	HC
0.23	0.68	0.52	3	16.48	HC
0.27	0.59	0.4	3	16.3	HC
0.33	0.52	0.31	3	17.54	HC
0.26	0.47	0.31	4	17.39	HC
0.23	0.4	0.24	4	16.13	IS
0.32	0.42	0.18	4	22.22	HC
0.32	0.45	0.21	4	20.94	HC

0.3	0.49	0.28	3	21.28	HC
0.27	0.47	0.28	4	20.83	HC
0.29	0.47	0.26	5	19.92	HC
0.3	0.43	0.21	4	21.16	HC
0.28	0.41	0.2	4	20.73	HC
0.3	0.39	0.17	4	21.05	HC
0.33	0.43	0.21	3	23.62	HC
0.36	0.53	0.3	3	22.9	HC
0.24	0.54	0.36	3	23.26	HC
0.25	0.6	0.41	3	24	HC
0.39	0.56	0.27	2	23.26	HC
0.4	0.69	0.44	3	20.55	HC
0.24	0.54	0.34	3	18.99	HC
0.32	0.57	0.35	4	18.02	HC
0.31	0.51	0.27	3	20.27	HC
0.28	0.49	0.29	3	21.74	CC
0.37	0.47	0.22	4	21.51	HC
0.34	0.53	0.31	3	20.98	HC
0.34	0.43	0.19	3	20.69	HC
0.38	0.47	0.16	3	20.41	HC
0.22	0.29	0.12	3	16.39	CC
0.19	0.22	0.05	4	16.39	PL
0.21	0.31	0.16	3	18.29	HC
0.18	0.27	0.11	4	16.88	CC
0.33	0.44	0.2	3	17.86	HC
0.31	0.45	0.25	3	17.54	HC
0.25	0.4	0.21	3	17.05	HC
0.31	0.47	0.25	3	17.34	CC
0.27	0.37	0.16	3	14.29	CC
0.25	0.42	0.25	3	15.31	HC
0.23	0.33	0.15	3	18.99	PL
0.22	0.29	0.12	4	18.96	HC
0.26	0.35	0.13	3	19.61	HC
0.21	0.27	0.09	3	23.44	PL
0.24	0.32	0.13	4	21.51	PL
0.25	0.32	0.14	4	20.62	HC
0.2	0.28	0.12	3	20.41	HC
0.24	0.31	0.1	4	18.69	HC
0.2	0.24	0.07	4	22.1	CC
0.22	0.32	0.16	3	21.9	HC
0.22	0.29	0.12	5	20	CC
0.3	0.4	0.18	3	22.56	HC
0.32	0.45	0.23	3	23.62	HC
0.26	0.37	0.17	4	20.1	HC
0.28	0.35	0.12	5	20.49	HC
0.25	0.36	0.16	4	21.74	HC
0.26	0.39	0.19	3	21.9	HC
0.42	0.75	0.47	5	18.12	HC
0.44	0.73	0.48	4	17.62	HC
0.44	0.7	0.4	4	18.52	HC
0.38	0.67	0.44	4	18.02	HC
0.39	0.72	0.45	5	18.12	HC
0.4	0.74	0.47	4	17.47	PL
0.4	0.8	0.54	7	15.95	HC

0.36	0.65	0.41	4	17.32	HC
0.41	0.8	0.57	4	17.62	HC
0.36	0.7	0.46	6	16.95	HC
0.31	0.76	0.54	5	17.73	HC
0.38	0.79	0.55	5	18.66	HC
0.32	0.72	0.52	4	19.05	HC
0.41	0.83	0.63	4	18.96	HC
0.44	0.75	0.5	4	18.69	HC
0.33	0.48	0.25	6	15.63	HC
0.26	0.4	0.19	4	17.86	HC
0.28	0.43	0.21	5	16.67	HC
0.24	0.39	0.27	4	18.78	PL
0.31	0.45	0.22	6	16.95	HC
0.28	0.44	0.23	5	15.77	CC
0.26	0.36	0.16	6	16	HC
0.33	0.59	0.38	6	18.99	HC
0.29	0.53	0.33	4	20.1	HC
0.34	0.5	0.26	5	18.45	HC
0.33	0.47	0.23	4	19.7	HC
0.34	0.45	0.2	4	19.23	HC
0.34	0.59	0.34	4	18.69	HC
0.33	0.48	0.24	5	17.36	HC
0.32	0.43	0.17	4	17.86	HC
0.23	0.51	0.33	5	16.18	HC
0.26	0.46	0.25	6	17.34	CC
0.24	0.46	0.27	6	15	HC
0.37	0.46	0.17	5	18.73	HC
0.26	0.39	0.19	4	18.78	HC
0.33	0.46	0.23	5	18.38	HC
0.32	0.39	0.14	5	19.01	HC
0.33	0.42	0.15	4	18.35	HC
0.29	0.49	0.31	6	16.71	HC
0.34	0.65	0.39	6	17.54	CC
0.36	0.63	0.37	5	18.05	HC
0.4	0.53	0.2	5	18.05	HC
0.41	0.62	0.33	3	21.43	HC
0.38	0.58	0.31	4	21.51	HC
0.34	0.61	0.38	4	20.83	HC
0.39	0.7	0.42	4	20.83	HC
0.37	0.53	0.27	4	17.94	HC
0.27	0.56	0.37	5	16.45	HC
0.39	0.58	0.28	4	17.86	HC
0.28	0.63	0.44	4	16.67	HC
0.28	0.64	0.45	4	18.78	HC
0.38	0.67	0.44	4	19.23	HC
0.29	0.68	0.48	5	18.25	HC
0.37	0.87	0	3	18.99	CC
0.38	0.44	0.16	3	19.35	HC
0.33	0.5	0.29	4	18.35	CC
0.39	0.49	0.2	5	19.92	HC
0.45	0.6	0.36	4	19.7	HC
0.32	0.43	0.18	5	18.59	HC
0.29	0.5	0.28	5	18.12	HC
0.31	0.45	0.22	5	19.76	HC

0.27	0.41	0.2	4	20.2	HC
0.34	0.46	0.21	4	17.02	HC
0.32	0.46	0.25	5	17.24	HC
0.34	0.43	0.17	4	19.23	HC
0.32	0.48	0.23	4	18.6	HC
1.67	2.16	0.98	0	0	OC
0.97	2.4	1.8	0	0	OH
1.15	2.22	1.64	0	0	PL
1.23	2	1.28	0	0	OH
1	2.12	1.52	0	0	OH
1.29	2.02	1.29	0	0	OH
1.09	2.17	1.55	0	0	PL
1.79	2.39	1.4	0	0	OH
0.9	2.31	1.82	0	0	OH
1.59	2.44	1.76	0	0	OH
1.25	2.22	1.37	0	0	OC
0.63	1.44	1.15	0	0	OH
0.89	1.56	1.04	0	0	PL
0.81	1.43	0.88	0	0	OH
0.72	1.72	1.27	0	0	OH
0.77	1.72	1.23	0	0	OH
1.18	1.72	1	0	0	OH
1.16	1.6	0.86	0	0	OC
0.71	1.6	1.12	0	0	PL
0.77	1.6	1.11	0	0	OH
0.82	1.46	1.02	0	0	OH
0.67	1.51	1.09	0	0	OH
0.64	1.46	1.07	0	0	PL
0.69	1.34	1.02	0	0	OH
0.91	1.33	0.82	0	0	OH
0.89	1.31	0.63	0	0	PL
0.87	1.35	0.76	0	0	OH
1.02	1.66	1.1	0	0	OH
0.73	1.51	1.1	0	0	OC
0.77	1.38	0.93	0	0	OC
0.72	1.54	1.18	0	0	OH
0.8	1.14	0.63	0	0	OH
0.76	1.23	0.75	0	0	OH
0.58	1.29	0.94	0	0	OH
0.52	1.28	1.06	0	0	OH
1.24	2.19	1.45	0	0	OH
1.34	1.91	1.03	0	0	PL
1.46	2.08	1.18	0	0	OH
1.05	1.84	1.3	0	0	OH
1.03	1.33	0.54	0	0	OH
0.85	1.33	0.86	0	0	OH
1	1.58	0.91	0	0	OH
0.98	1.71	1.19	0	0	OH
0.96	1.68	1.2	0	0	OH
1.13	1.62	1.01	0	0	OH
1.3	1.77	1	0	0	OC
1.5	1.91	1	0	0	OC
1.14	1.63	0.9	0	0	OC
1.06	1.73	1.15	0	0	PL

1.45	1.84	0.85	0	0	OC
0.34	0.48	0.22	0	0	OH
0.35	0.53	0.25	0	0	OH
0.33	0.52	0.31	0	0	OH
0.45	0.61	0.31	0	0	OH
0.35	0.43	0.19	0	0	OH
0.41	0.56	0.27	0	0	OH
0.32	0.45	0.2	0	0	OH
0.58	0.85	0.46	0	0	OH
0.45	0.93	0.61	0	0	OH
0.56	0.99	0.65	0	0	OH
0.47	1.01	0.71	0	0	OH
0.58	0.92	0.56	0	0	OH
0.49	0.75	0.44	0	0	OH
0.63	1.17	0.83	0	0	OH
0.46	0.98	0.76	0	0	OH
0.59	0.84	0.43	0	0	OH
0.44	0.79	0.5	0	0	OH
0.54	1.06	0.73	0	0	OH
0.48	0.92	0.61	0	0	OH
0.54	0.92	0.54	0	0	OH
0.52	0.68	0.33	0	0	OH
0.51	0.76	0.38	0	0	OH
0.46	0.77	0.45	0	0	OH
0.42	0.84	0.58	0	0	OH
0.32	0.74	0.57	0	0	OH
0.39	0.74	0.52	0	0	OH
0.36	0.7	0.47	0	0	OH
0.53	0.77	0.42	0	0	OH
0.38	0.76	0.51	0	0	OH
0.43	0.9	0.67	0	0	OH
0.4	0.8	0.54	0	0	OH
0.38	0.83	0.62	0	0	OH
0.5	0.93	0.6	0	0	OH
0.86	1.46	1.04	0	0	OC
0.64	1.19	0.85	0	0	OH
0.27	0.47	0.31	0	0	OH
0.32	0.45	0.23	0	0	OH
0.42	0.71	0.5	0	0	OH
0.45	0.67	0.36	0	0	OH
0.79	1.15	0.66	0	0	OH
0.95	1.33	0.77	0	0	OH
1.08	1.52	0.82	0	0	OH
1.38	2.09	1.12	0	0	OH
0.62	1.01	0.62	0	0	OH
0.57	1.03	0.71	0	0	OH
0.52	1.05	0.78	0	0	OH
0.65	1.13	0.78	0	0	PL
0.45	1.21	0.96	0	0	OH
0.56	1.17	0.77	0	0	OH
0.48	1.11	0.82	0	0	OC
0.53	1.14	0.86	0	0	OH
0.67	0.93	0.49	0	0	OH
0.58	0.98	0.55	0	0	OH

0.56	1.03	0.61	0	0	OC
0.66	0.93	0.51	0	0	OH
0.57	0.98	0.64	0	0	OH
0.61	1.01	0.6	0	0	OH
0.52	0.95	0.58	0	0	OH
0.63	1.24	0.81	0	0	OH
0.66	1.14	0.67	0	0	OH
0.63	1.19	0.82	0	0	OH
0.77	1.15	0.72	0	0	OH
0.81	1.2	0.64	0	0	OH
0.61	1.19	0.79	0	0	OH
0.57	1.18	0.79	0	0	OH
0.42	1.02	0.82	0	0	OH
0.42	0.97	0.71	0	0	OH
0.61	0.93	0.44	0	0	IS
0.55	1.05	0.7	0	0	OH
0.48	0.86	0.52	0	0	OH
0.6	0.92	0.47	0	0	OH
0.62	0.91	0.48	0	0	PL
0.69	1.17	0.76	0	0	OC
0.66	1.09	0.67	0	0	PL
0.6	1.09	0.77	0	0	PL
0.22	0.64	0.48	0	0	OH
0.24	0.62	0.45	0	0	OC
0.36	0.5	0.21	0	0	OH
0.38	0.47	0.15	0	0	OH
0.23	0.53	0.36	0	0	OH
0.42	0.48	0.1	0	0	OH
0.38	0.63	0.36	0	0	OH
0.37	0.67	0.42	0	0	OH
0.37	0.66	0.42	0	0	OH
0.41	0.54	0.21	0	0	OH
0.35	0.55	0.28	0	0	OH
0.39	0.57	0.28	0	0	OH
0.41	0.59	0.28	0	0	OH
0.39	0.67	0.41	0	0	OH
0.46	0.69	0.33	0	0	OH
0.43	0.78	0.46	0	0	OH
0.39	0.82	0.56	0	0	OH
0.5	0.83	0.49	0	0	OH
0.48	0.79	0.46	0	0	OH
0.56	0.7	0.26	0	0	OH
0.47	0.58	0.24	0	0	PL
0.54	0.75	0.4	0	0	OH
0.41	0.76	0.49	0	0	OH
0.44	0.94	0.58	0	0	OH
0.49	0.78	0.42	0	0	OH
0.22	0.33	0.19	0	0	OH
0.23	0.32	0.16	0	0	OH
0.24	0.34	0.17	0	0	OH
0.32	0.4	0.15	0	0	OH
0.24	0.39	0.24	0	0	OH
0.4	0.53	0.22	0	0	OH
0.4	0.54	0.23	0	0	OH

0.38	0.51	0.22	0	0	OH
0.28	0.55	0.37	0	0	OH
0.27	0.56	0.41	0	0	OH
0.26	0.46	0.3	0	0	OH
0.32	0.52	0.28	0	0	OH
0.29	0.52	0.31	0	0	OH
0.46	0.78	0.41	0	0	OC
0.43	0.74	0.39	0	0	OC
0.42	0.72	0.43	0	0	OH
0.38	0.72	0.47	0	0	OH
0.31	0.68	0.46	0	0	OC
0.35	0.58	0.35	0	0	OH
0.45	0.63	0.29	0	0	OH
0.44	0.61	0.29	0	0	OH
0.44	0.7	0.42	0	0	OH
0.38	0.66	0.4	0	0	OH
0.32	0.43	0.17	0	0	OH
0.34	0.43	0.15	0	0	OH
0.32	0.43	0.2	0	0	OH
0.26	0.43	0.25	0	0	OH
0.35	0.53	0.25	0	0	OH
0.32	0.59	0.38	0	0	OH
0.39	0.57	0.28	0	0	OH
0.42	0.6	0.28	0	0	OH
0.41	0.55	0.22	0	0	OH
0.38	0.59	0.29	0	0	OH
0.37	0.84	0.59	0	0	OH
0.61	0.88	0.46	0	0	OH
0.43	0.92	0.62	0	0	OH
0.53	0.85	0.57	0	0	OH
0.49	0.87	0.55	0	0	OH
0.53	0.87	0.52	0	0	OH
0.51	0.84	0.49	0	0	OH
0.41	0.82	0.53	0	0	OH
0.45	0.81	0.51	0	0	OH
0.57	0.78	0.32	0	0	OC
0.46	0.8	0.47	0	0	OH
0.45	0.8	0.49	0	0	OH
0.32	0.48	0.22	0	0	OH
0.3	0.41	0.22	0	0	OH
0.29	0.48	0.26	0	0	OH
0.3	0.51	0.32	0	0	OH
0.28	0.65	0.48	0	0	OH
0.39	0.65	0.37	0	0	OH
0.38	0.6	0.31	0	0	OH
0.41	0.56	0.24	0	0	OH
0.44	0.62	0.27	0	0	OH
0.43	0.59	0.23	0	0	OC
0.41	0.7	0.42	0	0	OH
0.43	0.74	0.49	0	0	OH
0.48	0.68	0.3	0	0	OH
0.49	0.71	0.34	0	0	OH
0.44	0.66	0.33	0	0	OH
0.37	0.7	0.43	0	0	OC

0.2	0.46	0.83	0	0	PL
0.28	0.75	0.61	0	0	OC
0.49	0.84	0.59	0	0	PL
0.51	0.67	0.3	0	0	PL
0.34	0.76	0.51	0	0	PL
0.52	0.75	0.34	0	0	IS
0.42	0.56	0.28	0	0	PL
0.33	0.54	0.3	0	0	IS
0.42	0.6	0.34	0	0	PL
0.52	0.77	0.35	0	0	PL
0.24	0.73	0.49	0	0	PL
0.4	0.6	0.36	0	0	PL
0.38	0.65	0.43	0	0	OC
0.44	0.72	0.48	0	0	PL
0.34	0.54	0.34	0	0	PL
0.43	0.56	0.26	0	0	PL
0.35	0.64	0.45	0	0	PL
0.34	0.47	0.26	0	0	IS
0.4	0.53	0.24	0	0	PL
0.47	0.75	0.43	0	0	PL
0.43	0.65	0.38	0	0	PL
0.57	0.74	0.35	0	0	IS
0.38	0.7	0.45	0	0	PL
0.61	0.73	0	0	0	PL
0.47	0.66	0.3	0	0	PL
0.41	0.78	0.55	0	0	PL
0.45	0.6	0.3	0	0	IS
0.45	0.6	0.3	0	0	IS
0.38	0.49	0.19	0	0	IS
0.61	0.69	0.18	0	0	PL
0.4	0.54	0.24	0	0	IS
0.34	0.54	0.3	0	0	IS
0.42	0.9	0.64	0	0	PL
0.32	0.84	0.61	0	0	PL
0.59	0.69	0.21	0	0	PL
0.5	0.63	0.28	0	0	PL
0.3	0.52	0.3	0	0	IS
0.45	0.73	0.47	0	0	PL
0.59	0.84	0.55	0	0	PL
0.45	0.59	0.32	0	0	IS
0.69	0.86	0.56	0	0	PL
0.3	0.58	0.41	0	0	PL
0.35	0.58	0.35	0	0	PL
0.33	0.43	0.41	0	0	OC
0.43	0.55	0.22	0	0	PL
0.24	0.65	0.48	0	0	PL
0.34	0.58	0.37	0	0	PL
0.51	0.66	0.32	0	0	PL
0.41	0.59	0.3	0	0	PL
0.86	0.93	0.16	0	0	PL
0.71	0.91	0.53	0	0	PL
0.44	0.75	0.48	0	0	PL
0.41	0.7	0.44	0	0	PL
0.68	0.97	0.48	0	0	PL

0.51	0.99	0.63	0	0	PL
0.52	0.95	0.74	0	0	PL
0.67	0.73	0.14	0	0	PL
0.47	0.81	0.54	0	0	OC
0.65	0.9	0.53	0	0	PL
0.58	0.8	0.52	0	0	PL
0.63	0.72	0.22	0	0	PL
0.48	0.67	0.29	0	0	PL
0.55	0.69	0.27	0	0	PL
0.64	0.93	0.54	0	0	PL
0.65	0.78	0.33	0	0	PL
0.73	0.94	0.54	0	0	PL
0.62	1.07	0.73	0	0	PL
0.38	0.81	0.62	0	0	PL
0.68	0.8	0.35	0	0	IS
0.54	0.98	0.71	0	0	PL
0.36	0.61	0.41	0	0	IS
0.48	0.7	0.42	0	0	PL
0.47	0.97	0.67	0	0	PL
0.63	0.96	0.65	0	0	PL
0.63	0.8	0.4	0	0	PL
0.35	0.67	0.43	0	0	IS
0.76	0.85	0.27	0	0	PL
0.66	0.87	0.48	0	0	PL
0.8	0.89	0.25	0	0	IS
0.87	1.03	0.35	0	0	IS
0.69	0.89	0.4	0	0	PL
0.61	0.89	0.49	0	0	PL
0.87	0.99	0.29	0	0	PL
0.78	1.01	0.42	0	0	IS
0.87	0.95	0.24	0	0	PL
0.42	0.55	0.26	0	0	IS
0.67	0.96	0.61	0	0	PL
0.38	0.55	0.35	0	0	IS
0.67	0.81	0.35	0	0	PL
0.45	0.56	0.19	0	0	BS
0.76	0.91	0.33	0	0	PL
0.42	0.54	0.24	0	0	IS
0.59	0.86	0.44	0	0	PL
0.74	1.03	0.56	0	0	OC
0.71	0.93	0.48	0	0	PL
0.65	1.06	0.74	0	0	PL
0.55	1.05	0.75	0	0	PL
0.42	0.66	0.46	0	0	PL
0.73	0.81	0.24	0	0	PL
0.56	0.79	0.45	0	0	OC
0.71	0.92	0.35	0	0	PL
0.65	0.75	0.26	0	0	PL
0.73	0.96	0.42	0	0	IS
0.47	0.68	0.38	0	0	IS
0.46	1	0.76	0	0	PL
0.65	0.91	0.48	0	0	PL
0.65	0.82	0.36	0	0	PL
0.76	0.81	0.15	0	0	PL

0.67	0.85	0.41	0	0	PL
0.78	0.89	0.31	0	0	PL
0.45	1.06	0.79	0	0	OC
0.66	1.06	0.74	0	0	PL
0.66	0.83	0.38	0	0	PL
0.39	0.8	0.59	0	0	PL
0.69	0.9	0.42	0	0	PL
0.81	1.07	0.61	0	0	PL
0.63	0.91	0.56	0	0	PL
0.87	1.01	0.26	0	0	IS
0.59	0.84	0.51	0	0	PL
0.69	0.81	0.32	0	0	PL
0.72	1.12	0.82	0	0	HC
0.82	0.95	0.39	0	0	PL
0.43	1.22	0.98	0	0	IS
0.77	1.14	0.77	0	0	PL
0.74	0.88	0.53	0	0	PL
0.66	0.88	0.42	0	0	PL
0.48	0.96	0.69	0	0	PL
0.46	0.63	0.3	0	0	IS
0.65	1.11	0.23	0	0	PL
0.74	1.24	0.88	0	0	PL
0.58	0.96	0.57	0	0	PL
0.52	1.25	0.94	0	0	PL
0.71	1.22	0.95	0	0	PL
0.49	0.62	0.33	0	0	IS
0.4	0.59	0.28	0	0	IS
0.57	1.15	0.83	0	0	PL
0.52	1.07	0.84	0	0	PL
0.44	0.9	0.71	0	0	OC
0.73	1.01	0.67	0	0	PL
0.79	1.3	0.96	0	0	PL
0.65	0.99	0.58	0	0	PL
0.83	1.09	0.58	0	0	PL
0.96	1.28	0.67	0	0	IS
0.97	1.13	0.5	0	0	PL
0.85	1.42	0.91	0	0	IS
0.96	1.12	0.27	0	0	IS
0.68	1.05	0.71	0	0	PL
0.99	1.14	0.32	0	0	PL
0.93	1.05	0.4	0	0	PL
0.54	0.67	0.3	0	0	IS
0.39	0.65	0.45	0	0	PL
0.6	0.71	0.34	0	0	PL
0.45	0.55	0.23	0	0	IS
0.63	0.68	0.15	0	0	PL
0.58	0.64	0.15	0	0	PL
0.43	0.55	0.25	0	0	IS
0.46	0.56	0.22	0	0	IS
0.37	0.66	0.43	0	0	IS
0.36	0.76	0.57	0	0	PL
0.49	0.64	0.33	0	0	PL
0.4	0.52	0.21	0	0	IS
0.52	0.67	0.3	0	0	IS

0.49	0.56	0.19	0	0	IS
0.55	0.6	0.12	0	0	PL
0.45	0.62	0.32	0	0	OC
0.3	0.62	0.48	0	0	PL
0.35	0.58	0.36	0	0	IS
0.46	0.83	0.54	0	0	PL
0.49	0.65	0.38	0	0	PL
0.47	0.68	0.38	0	0	PL
0.53	0.73	0.4	0	0	PL
0.56	0.75	0.41	0	0	PL
0.59	0.77	0.54	0	0	PL
0.58	0.68	0.29	0	0	PL
0.51	0.87	0.61	0	0	OC
0.54	0.72	0.35	0	0	PL
0.37	0.8	0.64	0	0	PL
0.37	0.63	0.4	0	0	PL
0.37	0.79	0.62	0	0	PL
0.49	0.63	0.28	0	0	PL
0.47	0.59	0.27	0	0	PL
0.38	0.55	0.34	0	0	IS
0.57	0.66	0.24	0	0	IS
0.28	0.57	0.41	0	0	IS
0.53	0.76	0.38	0	0	PL
0.26	0.61	0.43	0	0	PL
0.35	0.64	0.45	0	0	PL
0.41	0.66	0.4	0	0	PL
0.4	0.66	0.38	0	0	PL
0.34	0.63	0.43	0	0	PL
0.34	0.43	0.17	0	0	PL
0.23	0.57	0.41	0	0	PL
0.36	0.65	0.41	0	0	PL
0.3	0.55	0.36	0	0	PL
0.22	0.57	0.43	0	0	PL
0.41	0.58	0.27	0	0	PL
0.31	0.57	0.4	0	0	OC
0.36	0.51	0.28	0	0	IS
0.33	0.67	0.45	0	0	PL
0.39	0.69	0.46	0	0	PL
0.66	0.77	0.34	0	0	IS
0.37	0.83	0.63	0	0	OC
0.57	0.91	0.62	0	0	PL
0.66	0.94	0.59	0	0	PL
0.53	0.65	0.25	0	0	PL
0.58	0.69	0.24	0	0	PL
0.42	0.85	0.65	0	0	PL
0.49	0.87	0.67	0	0	PL
0.5	0.8	0.6	0	0	PL
0.32	0.81	0.63	0	0	OC
0.49	0.83	0.55	0	0	PL
0.68	0.83	0.3	0	0	PL
0.62	0.91	0.59	0	0	PL
0.58	0.79	0.43	0	0	PL
0.57	0.77	0.39	0	0	PL
0.57	0.81	0.45	0	0	IS

0.52	0.9	0.67	0	0	PL
0.56	0.82	0.58	0	0	PL
0.65	0.77	0.2	0	0	IS
0.5	0.81	0.54	0	0	PL
0.34	0.78	0.57	0	0	OC
0.53	0.7	0.31	0	0	PL
0.33	0.47	0.25	0	0	PL
0.29	0.57	0.34	0	0	IS
0.26	0.5	0.34	0	0	IS
0.21	0.3	0.15	0	0	OC
0.21	0.29	0.12	0	0	OC
0.2	0.32	0.18	0	0	OC
0.17	0.34	0.22	0	0	PL
0.3	0.41	0.22	0	0	PL
0.24	0.39	0.23	0	0	PL
0.22	0.29	0.13	0	0	PL
0.17	0.24	0.11	0	0	PL
0.27	0.37	0.17	0	0	PL
0.21	0.27	0.11	0	0	PL
0.24	0.42	0.26	0	0	PL
0.22	0.37	0.23	0	0	PL
0.24	0.4	0.22	0	0	PL
0.19	0.27	0.13	0	0	PL
0.23	0.3	0.13	0	0	PL
0.2	0.27	0.13	0	0	OC
0.19	0.25	0.09	0	0	OC
0.27	0.3	0.06	0	0	PL
0.19	0.24	0.09	0	0	OC
0.18	0.28	0.15	0	0	PL
0.18	0.26	0.12	0	0	OC
0.15	0.19	0.07	0	0	PL
0.16	0.22	0.1	0	0	PL
0.14	0.18	0.08	0	0	OC
0.14	0.18	0.07	0	0	PL
0.18	0.25	0.12	0	0	PL
0.31	0.51	0.19	0	0	OC
0.34	0.69	0.5	0	0	IS
0.47	0.5	0.22	0	0	PL
0.34	0.51	0.31	0	0	OC
0.29	0.66	0.49	0	0	PL
0.34	0.46	0.21	0	0	PL
0.36	0.44	0.24	0	0	PL
0.31	0.4	0.2	0	0	OC
0.28	0.39	0.18	0	0	PL
0.31	0.48	0.26	0	0	PL
0.26	0.49	0.31	0	0	OC
0.19	0.36	0.22	0	0	OC
0.28	0.49	0.31	0	0	PL
0.17	0.36	0.25	0	0	OC
0.4	0.51	0.27	0	0	PL
0.33	0.42	0.19	0	0	PL
0.31	0.41	0.21	0	0	PL
0.33	0.51	0.3	0	0	PL
0.25	0.39	0.24	0	0	OC

0.26	0.45	0.18	0	0	PL
0.22	0.33	0.18	0	0	PL
0.19	0.22	0.08	0	0	OC
0.24	0.37	0.18	0	0	PL
0.18	0.31	0.18	0	0	PL
0.18	0.24	0.09	0	0	OC
0.2	0.37	0.22	0	0	PL
0.27	0.37	0.19	0	0	PL
0.21	0.34	0.19	0	0	OC
0.21	0.27	0.12	0	0	OC
0.32	0.38	0.12	0	0	PL
0.35	0.4	0.1	0	0	PL
0.22	0.4	0.25	0	0	PL
0.21	0.3	0.15	0	0	OC
0.23	0.31	0.15	0	0	OC
0.25	0.39	0.21	0	0	PL
0.18	0.32	0.2	0	0	PL
0.22	0.33	0.17	0	0	OC
0.22	0.39	0.24	0	0	PL
0.17	0.33	0.21	0	0	PL
0.26	0.32	0.1	0	0	PL
0.21	0.42	0.27	0	0	PL
0.2	0.3	0.17	0	0	PL
0.21	0.26	0.09	0	0	PL
0.19	0.35	0.21	0	0	PL
0.22	0.29	0.12	0	0	OC
0.22	0.28	0.11	0	0	OC
0.17	0.21	0.08	0	0	OC
0.23	0.32	0.15	0	0	PL
0.26	0.34	0.15	0	0	PL
0.24	0.44	0.27	0	0	PL
0.21	0.46	0.31	0	0	PL
0.2	0.35	0.2	0	0	PL
0.33	0.4	0.13	0	0	PL
0.23	0.35	0.19	0	0	PL
0.23	0.4	0.22	0	0	PL
0.19	0.35	0.22	0	0	OC
0.23	0.3	0.16	0	0	PL
0.25	0.3	0.11	0	0	CC
0.19	0.22	0.07	0	0	CC
0.16	0.22	0.1	0	0	OC
0.16	0.25	0.13	0	0	OC
0.17	0.19	0.06	0	0	OC
0.15	0.19	0.08	0	0	OC
0.15	0.21	0.1	0	0	PL
0.15	0.21	0.09	0	0	PL
0.22	0.3	0.16	0	0	PL
0.17	0.37	0.26	0	0	PL
0.28	0.38	0.15	0	0	PL
0.37	0.53	0.33	0	0	IS
0.19	0.46	0.32	0	0	HC
0.24	0.31	0.14	0	0	OC
0.24	0.35	0.18	0	0	PL
0.21	0.33	0.22	0	0	PL

0.21	0.46	0.32	0	0	PL
0.2	0.41	0.27	0	0	IS
0.31	0.37	0.13	0	0	PL
0.26	0.37	0.17	0	0	PL
0.32	0.47	0.25	0	0	PL
0.28	0.44	0.24	0	0	PL
0.29	0.5	0.35	0	0	PL
0.23	0.38	0.23	0	0	OC
0.27	0.5	0.32	0	0	PL
0.29	0.41	0.2	0	0	PL
0.18	0.25	0.13	0	0	PL
0.14	0.19	0.09	0	0	PL
0.15	0.23	0.11	0	0	PL
0.15	0.2	0.09	0	0	PL
0.13	0.17	0.08	0	0	PL
0.12	0.21	0.12	0	0	PL
0.14	0.23	0.14	0	0	PL
0.26	0.29	0.07	0	0	PL
0.27	0.34	0.13	0	0	PL
0.31	0.37	0.11	0	0	PL
0.23	0.32	0.17	0	0	PL
0.24	0.35	0.17	0	0	CC
0.16	0.28	0.18	0	0	PL
0.22	0.34	0.2	0	0	PL
0.14	0.31	0.22	0	0	PL
0.32	0.46	0.28	0	0	IS
0.42	0.49	0.14	0	0	HC
0.33	0.54	0.36	0	0	PL
0.23	0.34	0.18	0	0	PL
0.24	0.46	0.33	0	0	PL
0.24	0.43	0.27	0	0	PL
0.2	0.4	0.26	0	0	OC
0.27	0.38	0.2	0	0	PL
0.4	0.46	0.14	0	0	PL
0.26	0.38	0.23	0	0	PL
0.31	0.37	0.11	0	0	PL
0.29	0.4	0.21	0	0	PL
0.3	0.38	0.13	0	0	HC
0.31	0.48	0.3	0	0	PL
0.27	0.43	0.28	0	0	PL
0.27	0.43	0.28	0	0	PL
0.36	0.48	0.24	0	0	PL
0.39	0.55	0.26	0	0	PL
0.24	0.46	0.3	0	0	OC
0.29	0.48	0.31	0	0	PL
0.35	0.59	0.37	0	0	PL
0.19	0.64	0.5	0	0	PL
0.2	0.6	0.46	0	0	PL
0.27	0.54	0.4	0	0	OC
0.32	0.59	0.38	0	0	OC
0.31	0.4	0.23	0	0	OC
0.24	0.53	0.4	0	0	PL
0.29	0.38	0.2	0	0	PL
0.24	0.42	0.4	0	0	PL

0.31	0.45	0.23	0	0	PL
0.45	0.61	0.26	0	0	PL
0.32	0.59	0.46	0	0	PL
0.43	0.53	0.22	0	0	PL
0.28	0.44	0.26	0	0	PL
0.3	0.38	0.16	0	0	PL
0.32	0.43	0.19	0	0	PL
0.39	0.56	0.33	0	0	IS
0.23	0.55	0.42	0	0	PL
0.34	0.43	0.19	0	0	PL
0.37	0.62	0.44	0	0	PL
0.3	0.47	0.25	0	0	HC
0.18	0.3	0.16	0	0	IS
0.19	0.27	0.14	0	0	PL
0.23	0.29	0.11	0	0	OC
0.26	0.31	0.11	0	0	HC
0.21	0.34	0.2	0	0	PL
0.24	0.38	0.25	0	0	PL
0.32	0.41	0.17	0	0	PL
0.29	0.36	0.12	0	0	PL
0.23	0.39	0.29	0	0	PL
0.33	0.41	0.17	0	0	PL
0.21	0.35	0.23	0	0	PL
0.2	0.24	0.07	0	0	CC
0.2	0.24	0.06	0	0	CC
0.19	0.31	0.18	0	0	PL
0.14	0.24	0.15	0	0	PL
0.21	0.39	0.27	0	0	PL
0.14	0.23	0.15	0	0	OC
0.21	0.33	0.17	0	0	PL
0.2	0.29	0.13	0	0	OC
0.11	0.21	0.12	0	0	PL
0.13	0.17	0.07	0	0	PL
0.14	0.19	0.1	0	0	PL
0.18	0.31	0.18	0	0	PL
0.21	0.26	0.09	0	0	PL
0.25	0.3	0.1	0	0	PL
0.23	0.31	0.12	0	0	HC
0.27	0.52	0.31	0	0	PL
0.34	0.61	0.36	0	0	PL
0.29	0.48	0.26	0	0	PL
0.26	0.41	0.22	0	0	PL
0.35	0.43	0.15	0	0	PL
0.27	0.42	0.23	0	0	OC
0.32	0.4	0.14	0	0	PL
0.3	0.41	0.17	0	0	PL
0.47	0.58	0.15	0	0	PL
0.29	0.48	0.28	0	0	PL
0.25	0.68	0.35	0	0	PL
0.32	0.57	0.33	0	0	PL
0.41	0.69	0.16	0	0	PL
0.35	0.57	0.31	0	0	PL
0.43	0.61	0.3	0	0	PL
0.38	0.42	0.09	0	0	PL

0.21	0.33	0.18	0	0	OC
0.32	0.39	0.15	0	0	PL
0.29	0.5	0.28	0	0	PL
0.31	0.5	0.27	0	0	PL
0.22	0.45	0.29	0	0	PL
0.28	0.41	0.2	0	0	PL
0.33	0.48	0.24	0	0	PL
0.23	0.45	0.28	0	0	PL
0.39	0.66	0.39	0	0	PL
0.45	0.74	0.3	0	0	PL
0.37	0.64	0.39	0	0	PL
0.24	0.48	0.3	0	0	PL
0.43	0.9	0.12	0	0	PL
0.27	0.61	0.45	0	0	PL
0.35	1.03	0.22	0	0	PL
0.45	0.74	0.26	0	0	PL
0.48	0.57	0.18	0	0	PL
0.25	0.34	0.15	0	0	PL
0.18	0.27	0.13	0	0	PL
0.18	0.29	0.17	0	0	PL
0.21	0.31	0.15	0	0	OC
0.21	0.37	0.22	0	0	PL
0.25	0.41	0.24	0	0	PL
0.17	0.31	0.19	0	0	OC
0.23	0.34	0.17	0	0	PL
0.28	0.34	0.11	0	0	PL
0.35	0.44	0.19	0	0	PL
0.36	0.46	0.2	0	0	OC
0.29	0.59	0.4	0	0	PL
0.3	0.43	0.23	0	0	OC
0.32	0.44	0.24	0	0	PL
0.33	0.44	0.22	0	0	PL
0.41	0.46	0.12	0	0	PL
0.25	0.41	0.22	0	0	PL
0.27	0.47	0.29	0	0	PL
0.23	0.4	0.24	0	0	PL
0.24	0.38	0.21	0	0	PL
0.27	0.58	0.42	0	0	PL
0.33	0.43	0.21	0	0	PL
0.4	0.49	0.17	0	0	PL
0.37	0.46	0.19	0	0	PL
0.31	0.41	0.18	0	0	PL
0.25	0.45	0.25	0	0	PL
0.22	0.3	0.14	0	0	PL
0.25	0.41	0.21	0	0	PL
0.23	0.34	0.19	0	0	OC
0.29	0.34	0.12	0	0	PL
0.23	0.37	0.18	0	0	PL
0.2	0.38	0.23	0	0	PL
0.22	0.38	0.21	0	0	OC
0.19	0.26	0.1	0	0	PL
0.17	0.23	0.09	0	0	PL
0.18	0.24	0.1	0	0	PL
0.17	0.23	0.1	0	0	PL

0.19	0.25	0.11	0	0	OC
0.18	0.22	0.08	0	0	PL
0.23	0.3	0.13	0	0	HC
0.3	0.36	0.13	0	0	PL
0.23	0.37	0.21	0	0	OC
0.24	0.35	0.19	0	0	PL
0.25	0.36	0.17	0	0	PL
0.28	0.38	0.17	0	0	PL
0.18	0.29	0.16	0	0	OC
0.24	0.31	0.14	0	0	PL
0.18	0.29	0.16	0	0	OC
0.34	0.63	0.45	0	0	OC
0.47	0.65	0.31	0	0	IS
0.35	0.53	0.37	0	0	PL
0.34	0.63	0.45	0	0	PL
0.41	0.61	0.42	0	0	IS
0.33	0.46	0.26	0	0	IS
0.24	0.53	0.39	0	0	PL
0.25	0.61	0.46	0	0	PL
0.21	0.43	0.3	0	0	PL
0.22	0.46	0.35	0	0	OC
0.38	0.47	0.18	0	0	HC
0.31	0.42	0.21	0	0	IS
0.3	0.41	0.24	0	0	PL
0.25	0.52	0.36	0	0	OC
0.35	0.41	0.16	0	0	PL
0.31	0.4	0.18	0	0	PL
0.35	0.42	0.13	0	0	PL
0.34	0.4	0.15	0	0	PL
0.24	0.47	0.33	0	0	PL
0.3	0.59	0.38	0	0	IS
0.35	0.56	0.33	0	0	IS
0.36	0.49	0.3	0	0	IS
0.31	0.46	0.25	0	0	PL
0.33	0.52	0.39	0	0	PL
0.38	0.68	0.45	0	0	PL
0.4	0.56	0.32	0	0	IS
0.35	0.63	0.42	0	0	IS
0.35	0.63	0.42	0	0	IS
0.33	0.52	0.33	0	0	IS
0.32	0.56	0.42	0	0	PL
0.46	0.55	0.19	0	0	OC
0.39	0.52	0.29	0	0	IS
0.41	0.58	0.31	0	0	IS
0.41	0.6	0.39	0	0	PL
0.39	0.59	0.37	0	0	PL
0.43	0.6	0.28	0	0	IS
0.37	0.52	0.3	0	0	IS
0.37	0.52	0.24	0	0	IS
0.26	0.53	0.37	0	0	OC
0.42	0.52	0.23	0	0	IS
0.41	0.47	0.22	0	0	IS
0.35	0.48	0.23	0	0	IS
0.34	0.54	0.34	0	0	IS

0.45	0.76	0.55	0	0	IS
0.51	0.63	0.29	0	0	PL
0.4	0.58	0.31	0	0	IS
0.49	0.75	0.42	0	0	IS
0.45	0.67	0.34	0	0	IS
0.41	0.49	0.27	0	0	IS
0.58	0.85	0.55	0	0	PL
0.36	0.47	0.26	0	0	IS
0.31	0.49	0.29	0	0	IS
0.2	0.75	0.63	0	0	PL
0.33	0.45	0.23	0	0	IS
0.29	0.51	0.32	0	0	IS
0.47	0.54	0.22	0	0	IS
0.43	0.48	0.2	0	0	IS
0.38	0.51	0.24	0	0	IS
0.3	0.73	0.54	0	0	PL
0.3	0.6	0.39	0	0	IS
0.31	0.72	0.52	0	0	PL
0.44	0.55	0.2	0	0	IS
0.29	0.47	0.28	0	0	IS
0.25	0.34	0.14	0	0	IS
0.17	0.8	0.72	0	0	PL
0.75	0.83	0.19	0	0	PL
0.37	0.52	0.29	0	0	IS
0.54	0.76	0.41	0	0	PL
0.38	0.98	0.77	0	0	PL
0.61	0.85	0.58	0	0	PL
0.31	0.49	0.29	0	0	IS
0.39	0.79	0.62	0	0	OH
0.35	0.55	0.38	0	0	IS
0.28	0.46	0.28	0	0	OC
0.34	0.52	0.28	0	0	IS
0.3	0.49	0.31	0	0	PL
0.26	0.36	0.18	0	0	IS
0.24	0.35	0.15	0	0	IS
0.23	0.42	0.28	0	0	IS
0.36	0.49	0.24	0	0	IS
0.28	0.41	0.21	0	0	IS
0.36	0.44	0.22	0	0	IS
0.29	0.43	0.29	0	0	IS
0.34	0.44	0.26	0	0	PL
0.46	0.75	0.5	0	0	PL
0.51	0.57	0.14	0	0	BS
0.3	0.39	0.18	0	0	IS
0.34	0.45	0.2	0	0	IS
0.36	0.43	0.19	0	0	IS
0.32	0.44	0.21	0	0	IS
0.31	0.44	0.21	0	0	IS
0.37	0.43	0.19	0	0	IS
0.29	0.42	0.24	0	0	IS
0.35	0.73	0.54	0	0	PL
0.3	0.39	0.18	0	0	IS
0.42	0.67	0.37	0	0	PL
0.31	0.44	0.27	0	0	IS

0.55	0.71	0.33	0	0	IS
0.47	0.59	0.26	0	0	IS
0.52	0.63	0.26	0	0	IS
0.66	0.75	0.2	0	0	IS
0.57	0.83	0.41	0	0	IS
0.64	0.9	0.54	0	0	IS
0.56	0.68	0.26	0	0	IS
0.52	0.66	0.28	0	0	IS
0.46	0.61	0.26	0	0	IS
0.48	0.6	0.29	0	0	IS
0.4	0.63	0.36	0	0	IS
0.48	0.58	0.21	0	0	IS
0.45	0.69	0.46	0	0	IS
0.61	0.84	0.4	0	0	IS
0.61	0.73	0.27	0	0	IS
0.56	0.68	0.27	0	0	IS
0.61	0.75	0.34	0	0	IS
0.5	0.59	0.25	0	0	IS
0.47	0.62	0.27	0	0	IS
0.57	0.67	0.28	0	0	IS
0.53	0.75	0.34	0	0	IS
0.59	0.8	0.4	0	0	IS
0.57	0.7	0.25	0	0	IS
0.52	0.66	0.26	0	0	BS
0.52	0.72	0.39	0	0	IS
0.73	0.84	0.23	0	0	IS
0.58	0.71	0.27	0	0	IS
0.59	0.74	0.25	0	0	IS
0.41	0.52	0.34	0	0	IS
0.25	0.62	0.46	0	0	PL
0.56	0.7	0.33	0	0	IS
0.61	0.74	0.31	0	0	IS
0.44	0.52	0.18	0	0	PL
0.54	0.67	0.24	0	0	IS
0.52	0.76	0.35	0	0	IS
0.58	0.69	0.22	0	0	IS
0.27	0.54	0.36	0	0	PL
0.5	0.65	0.33	0	0	IS
0.61	0.72	0.23	0	0	IS
0.46	0.61	0.27	0	0	IS
0.52	0.6	0.25	0	0	IS
0.56	0.68	0.28	0	0	IS
0.82	0.9	0.16	0	0	IS
0.49	0.6	0.3	0	0	IS
0.55	0.64	0.22	0	0	IS
0.74	0.87	0.32	0	0	IS
0.84	1.02	0.37	0	0	IS
0.71	0.9	0.38	0	0	IS
0.62	0.87	0.53	0	0	IS
0.55	0.69	0.27	0	0	IS
0.34	0.61	0.42	0	0	PL
0.4	0.81	0.54	0	0	IS
0.43	0.84	0.55	0	0	IS
0.36	0.51	0.32	0	0	IS

0.7	0.84	0.24	0	0	IS
0.29	0.46	0.54	0	0	PL
0.64	0.8	0.34	0	0	IS
0.71	0.83	0.22	0	0	IS
0.32	0.39	0.13	0	0	PL
0.24	0.34	0.17	0	0	PL
0.66	0.77	0.32	0	0	IS
0.25	0.39	0.2	0	0	PL
0.26	0.34	0.12	0	0	PL
0.66	0.8	0.25	0	0	IS
0.23	0.29	0.1	0	0	HC
0.39	0.47	0.17	0	0	PL
0.34	0.5	0.29	0	0	IS
0.33	0.41	0.17	0	0	PL
0.28	0.37	0.17	0	0	IS
0.36	0.46	0.15	0	0	IS
0.32	0.42	0.23	0	0	PL
0.68	0.79	0.15	0	0	IS
0.38	0.48	0.23	0	0	PL
0.42	0.47	0.12	0	0	PL
0.34	0.44	0.24	0	0	PL
0.47	0.66	0.36	0	0	IS
0.31	0.5	0.3	0	0	PL
0.28	0.36	0.13	0	0	PL
0.28	0.37	0.17	0	0	PL
0.26	0.35	0.13	0	0	PL
0.3	0.38	0.2	0	0	PL
0.32	0.46	0.26	0	0	HC
0.25	0.5	0.32	0	0	OC
0.24	0.49	0.36	0	0	PL
0.23	0.31	0.15	0	0	PL
0.27	0.4	0.24	0	0	PL
0.18	0.46	0.32	0	0	OC
0.28	0.35	0.12	0	0	PL
0.28	0.43	0.22	0	0	OC
0.26	0.45	0.26	0	0	PL
0.33	0.42	0.16	0	0	PL
0.37	0.41	0.07	0	0	HC
0.24	0.36	0.17	0	0	PL
0.25	0.36	0.17	0	0	PL
0.32	0.38	0.11	0	0	HC
0.3	0.37	0.16	0	0	PL
0.31	0.34	0.09	0	0	PL
0.22	0.41	0.24	0	0	PL
0.22	0.5	0.36	0	0	PL
0.31	0.43	0.23	0	0	PL
0.22	0.52	0.38	0	0	PL
0.36	0.45	0.19	0	0	PL
0.29	0.39	0.17	0	0	PL
0.35	0.43	0.16	0	0	PL
0.19	0.22	0.06	0	0	HC
0.18	0.24	0.09	0	0	IS
0.17	0.25	0.11	0	0	PL
0.18	0.26	0.12	0	0	PL

0.17	0.21	0.08	0	0	HC
0.24	0.31	0.12	0	0	PL
0.22	0.3	0.13	0	0	PL
0.23	0.29	0.12	0	0	PL
0.2	0.28	0.12	0	0	PL
0.2	0.34	0.18	0	0	PL
0.21	0.33	0.18	0	0	PL
0.19	0.24	0.07	0	0	IS
0.18	0.27	0.12	0	0	PL
0.2	0.26	0.1	0	0	PL
0.21	0.27	0.13	0	0	PL
0.27	0.3	0.08	0	0	PL
0.23	0.27	0.08	0	0	PL
0.21	0.29	0.14	0	0	PL
0.18	0.26	0.12	0	0	PL
0.19	0.22	0.07	0	0	HC
0.16	0.19	0.06	0	0	PL
0.19	0.26	0.11	0	0	PL
0.19	0.26	0.14	0	0	PL
0.22	0.29	0.12	0	0	PL
0.18	0.29	0.14	0	0	PL
0.17	0.22	0.1	0	0	PL
0.21	0.24	0.05	0	0	PL
0.2	0.25	0.08	0	0	PL
0.21	0.28	0.1	0	0	OC
0.19	0.26	0.1	0	0	PL
0.16	0.23	0.1	0	0	PL
0.21	0.27	0.09	0	0	HC
0.24	0.35	0.21	0	0	PL
0.2	0.32	0.19	0	0	PL
0.28	0.52	0.29	0	0	IS
0.19	0.35	0.23	0	0	IS
0.52	0.61	0.16	0	0	IS
0.2	0.41	0.27	0	0	PL
0.18	0.23	0.11	0	0	PL
0.17	0.27	0.15	0	0	HC
0.18	0.22	0.09	0	0	HC
0.14	0.27	0.16	0	0	IS
0.2	0.33	0.2	0	0	PL
0.21	0.26	0.09	0	0	PL
0.29	0.38	0.18	0	0	IS
0.23	0.31	0.16	0	0	PL
0.21	0.33	0.22	0	0	PL
0.27	0.43	0.22	0	0	PL
0.26	0.38	0.22	0	0	HC
0.18	0.33	0.24	0	0	PL
0.16	0.34	0.24	0	0	OC
0.15	0.21	0.11	0	0	PL
0.12	0.16	0.06	0	0	PL
0.16	0.22	0.1	0	0	PL
0.2	0.34	0.19	0	0	PL
0.28	0.33	0.11	0	0	PL
0.19	0.28	0.13	0	0	PL
0.2	0.28	0.12	0	0	HC

0.26	0.33	0.17	0	0	HC
0.22	0.34	0.2	0	0	PL
0.21	0.31	0.16	0	0	PL
0.22	0.27	0.09	0	0	PL
0.23	0.27	0.07	0	0	HC
0.22	0.29	0.14	0	0	PL
0.23	0.32	0.16	0	0	HC
0.2	0.29	0.15	0	0	HC
0.27	0.39	0.22	0	0	PL
0.28	0.38	0.17	0	0	HC
0.21	0.38	0.23	0	0	PL
0.24	0.32	0.15	0	0	PL
0.25	0.36	0.21	0	0	PL
0.2	0.37	0.22	0	0	PL
0.5	0.6	0.26	0	0	BS
0.26	0.42	0.25	0	0	PL
0.19	0.26	0.13	0	0	PL
0.22	0.33	0.19	0	0	PL
0.21	0.42	0.31	0	0	IS
0.43	0.54	0.24	0	0	IS
0.27	0.42	0.24	0	0	PL
0.26	0.37	0.2	0	0	PL
0.25	0.39	0.25	0	0	IS
0.53	0.72	0.37	0	0	IS
0.47	0.64	0.31	0	0	IS
0.29	0.37	0.16	0	0	PL
0.29	0.34	0.11	0	0	PL
0.3	0.41	0.17	0	0	IS
0.26	0.36	0.22	0	0	HC
0.3	0.4	0.18	0	0	PL
0.26	0.35	0.17	0	0	HC
0.5	0.76	0.48	0	0	IS
0.29	0.37	0.17	0	0	PL
0.38	0.46	0.12	0	0	PL
0.57	0.65	0.2	0	0	BS
0.47	0.58	0.2	0	0	IS
0.19	0.46	0.34	0	0	PL
0.25	0.44	0.26	0	0	PL
0.23	0.48	0.35	0	0	IS
0.25	0.46	0.3	0	0	PL
0.41	0.64	0.39	0	0	IS
0.49	0.52	0.1	0	0	IS
0.27	0.4	0.22	0	0	PL
0.32	0.41	0.16	0	0	PL
0.41	0.49	0.16	0	0	IS
0.43	0.55	0.25	0	0	IS
0.33	0.63	0.42	0	0	IS
0.5	0.65	0.41	0	0	IS
0.29	0.37	0.2	0	0	HC
0.67	0.8	0.24	0	0	PL
0.72	0.78	0.14	0	0	PL
0.68	0.79	0.22	0	0	PL
0.61	0.77	0.3	0	0	PL
0.61	0.74	0.35	0	0	PL

0.58	0.74	0.28	0	0	PL
0.61	0.9	0.55	0	0	PL
0.55	0.76	0.32	0	0	PL
0.43	0.73	0.48	0	0	PL
0.6	0.71	0.31	0	0	PL
0.46	0.78	0.46	0	0	PL
0.47	0.82	0.55	0	0	PL
0.46	0.6	0.28	0	0	PL
0.47	0.63	0.3	0	0	PL
0.47	0.76	0.47	0	0	PL
0.42	0.73	0.5	0	0	PL
0.49	0.64	0.29	0	0	IS
0.48	0.81	0.59	0	0	OC
0.54	0.67	0.21	0	0	PL
0.39	0.55	0.31	0	0	IS
0.32	0.55	0.34	0	0	PL
0.54	0.59	0.12	0	0	PL
0.3	0.72	0.53	0	0	PL
0.45	0.58	0.3	0	0	PL
0.53	0.8	0.53	0	0	OC
0.43	0.85	0.64	0	0	PL
0.3	0.64	0.45	0	0	IS
0.55	0.69	0.34	0	0	PL
0.5	0.83	0.61	0	0	PL
0.63	0.72	0.2	0	0	PL
0.7	0.76	0.16	0	0	PL
0.59	0.78	0.39	0	0	IS
0.65	0.75	0.26	0	0	PL
0.66	0.84	0.37	0	0	PL
0.59	0.74	0.3	0	0	PL
0.43	0.53	0.17	0	0	IS
0.46	0.67	0.33	0	0	OC
0.43	0.8	0.58	0	0	PL
0.45	0.64	0.38	0	0	PL
0.46	0.7	0.43	0	0	PL
0.46	0.58	0.26	0	0	IS
0.45	0.68	0.45	0	0	PL
0.41	0.9	0.68	0	0	PL
0.41	0.87	0.67	0	0	OC
0.42	0.79	0.57	0	0	PL
0.32	0.61	0.43	0	0	OH
0.6	0.83	0.59	0	0	PL
0.74	0.94	0.45	0	0	PL
0.51	0.68	0.34	0	0	PL
0.68	0.95	0.57	0	0	PL
0.6	0.71	0.23	0	0	PL
0.62	0.89	0.37	0	0	IS
0.73	0.86	0.29	0	0	PL
0.68	0.82	0.45	0	0	PL
0.29	0.56	0.37	0	0	PL
0.24	0.52	0.36	0	0	PL
0.27	0.55	0.35	0	0	PL
0.31	0.6	0.4	0	0	PL
0.23	0.36	0.17	0	0	PL

0.24	0.32	0.14	0	0	PL
0.27	0.46	0.28	0	0	OC
0.3	0.44	0.22	0	0	OC
0.24	0.43	0.24	0	0	PL
0.33	0.44	0.22	0	0	PL
0.31	0.42	0.23	0	0	PL
0.36	0.43	0.15	0	0	PL
0.31	0.47	0.26	0	0	PL
0.26	0.38	0.19	0	0	OC
0.38	0.45	0.17	0	0	PL
0.38	0.43	0.11	0	0	PL
0.23	0.42	0.25	0	0	PL
0.27	0.47	0.27	0	0	PL
0.3	0.42	0.24	0	0	PL
0.22	0.43	0.27	0	0	OC
0.23	0.47	0.33	0	0	PL
0.34	0.46	0.21	0	0	PL
0.4	0.51	0.2	0	0	PL
0.29	0.56	0.39	0	0	OC
0.35	0.76	0.53	0	0	PL
0.28	0.53	0.35	0	0	PL
0.41	0.69	0.5	0	0	PL
0.37	0.66	0.43	0	0	PL
0.38	0.69	0.5	0	0	PL
0.45	0.55	0.19	0	0	IS
0.62	0.69	0.15	0	0	PL
0.36	0.47	0.23	0	0	IS
0.62	0.71	0.13	0	0	PL
0.39	0.76	0.53	0	0	PL
0.35	0.69	0.5	0	0	PL
0.91	1.42	0.73	0	0	IS
0.51	0.58	0.21	0	0	PL
0.54	0.6	0.18	0	0	PL
1.08	1.49	0.86	0	0	IS
0.97	1.55	1.08	0	0	IS
0.33	0.59	0.53	0	0	PL
0.36	0.54	0.28	0	0	OC
0.33	0.65	0.46	0	0	PL
0.68	1.72	1.37	0	0	BS
0.43	0.59	0.28	0	0	PL
0.25	0.6	0.43	0	0	PL
0.4	0.63	0.36	0	0	PL
0.45	0.61	0.32	0	0	IS
0.32	0.71	0.51	0	0	PL
0.43	0.57	0.32	0	0	PL
0.81	1.54	0.97	0	0	IS
0.33	0.59	0.4	0	0	PL
0.23	0.62	0.45	0	0	PL
0.35	0.67	0.43	0	0	PL
0.39	0.58	0.31	0	0	OC
0.3	0.6	0.4	0	0	PL
0.79	1.18	0.84	0	0	IS
0.57	0.86	0.44	0	0	IS
0.43	0.65	0.37	0	0	PL

0.53	0.67	0.27	0	0	PL
0.6	0.67	0.15	0	0	PL
0.97	1.56	1.22	0	0	IS
1.14	1.46	0.79	0	0	IS
0.55	0.89	0.48	0	0	IS
1.07	1.43	1.03	0	0	IS
0.49	0.77	0.4	0	0	IS
0.63	1.27	0.94	0	0	IS
0.36	0.6	0.38	0	0	PL
0.53	0.76	0.48	0	0	IS
0.45	0.72	0.4	0	0	PL
0.35	0.74	0.54	0	0	PL
0.45	0.72	0.4	0	0	PL
0.5	0.67	0.36	0	0	PL
0.47	0.66	0.34	0	0	PL
0.42	0.6	0.32	0	0	PL
0.57	0.7	0.3	0	0	IS
0.48	0.63	0.31	0	0	PL
0.23	0.34	0.17	0	0	OC
0.41	0.49	0.16	0	0	PL
0.26	0.37	0.18	0	0	PL
0.31	0.44	0.21	0	0	PL
0.23	0.3	0.11	0	0	OC
0.21	0.36	0.2	0	0	PL
0.21	0.3	0.13	0	0	OC
0.21	0.29	0.14	0	0	OC
0.44	0.6	0.22	0	0	PL
0.28	0.36	0.12	0	0	PL
0.27	0.43	0.22	0	0	OC
0.48	0.6	0.23	0	0	IS
0.44	0.54	0.24	0	0	IS
0.78	1.08	0.53	0	0	IS
0.24	0.39	0.21	0	0	PL
0.27	0.54	0.35	0	0	OC
0.3	0.46	0.25	0	0	PL
0.6	0.66	0.15	0	0	PL
0.68	1.16	0.8	0	0	IS
0.5	0.6	0.23	0	0	PL
0.64	0.76	0.37	0	0	IS
0.53	0.75	0.34	0	0	IS
0.64	0.83	0.38	0	0	IS
0.62	0.74	0.27	0	0	PL
0.7	0.74	0.25	0	0	IS
0.5	0.79	0.46	0	0	IS
0.69	0.78	0.29	0	0	IS
0.46	0.74	0.45	0	0	IS
0.72	0.88	0.42	0	0	IS
0.41	0.76	0.51	0	0	PL
0.67	0.83	0.47	0	0	IS
0.63	0.78	0.56	0	0	IS
0.74	0.93	0.45	0	0	IS
0.55	0.73	0.39	0	0	IS
0.74	0.81	0.13	0	0	PL
0.49	0.59	0.16	0	0	IS

0.55	0.71	0.34	0	0	IS
0.68	0.77	0.23	0	0	PL
0.53	0.72	0.34	0	0	IS
0.52	0.65	0.28	0	0	IS
0.41	0.79	0.54	0	0	PL
0.48	0.74	0.4	0	0	IS
0.53	0.78	0.37	0	0	IS
0.52	0.63	0.23	0	0	IS
0.42	0.61	0.3	0	0	IS
0.37	0.68	0.47	0	0	IS
0.5	0.58	0.15	0	0	IS
0.52	0.76	0.35	0	0	IS
0.45	0.64	0.29	0	0	IS
0.56	0.69	0.26	0	0	IS
0.4	0.85	0.58	0	0	PL
0.56	0.68	0.22	0	0	IS
0.53	0.71	0.35	0	0	IS
0.58	0.67	0.29	0	0	IS
0.5	0.69	0.34	0	0	IS
0.52	0.68	0.32	0	0	BS
0.51	0.64	0.27	0	0	IS
0.52	0.73	0.4	0	0	IS
0.55	0.9	0.62	0	0	IS
0.48	0.89	0.67	0	0	PL
0.61	0.75	0.32	0	0	IS
0.59	0.71	0.27	0	0	IS
0.7	1.09	0.57	0	0	IS
0.64	1.07	0.62	0	0	IS
0.64	1.05	0.62	0	0	IS
0.59	1.11	0.77	0	0	IS
0.65	0.74	0.23	0	0	IS
0.64	0.78	0.4	0	0	IS
0.65	0.79	0.46	0	0	IS
0.54	0.78	0.48	0	0	IS
0.43	0.54	0.19	0	0	IS
0.45	0.49	0.14	0	0	IS
0.54	0.68	0.27	0	0	IS
0.69	0.94	0.5	0	0	BS
0.44	0.53	0.23	0	0	IS
0.42	0.5	0.19	0	0	IS
0.71	1.01	0.52	0	0	IS
0.37	0.4	0.06	0	0	HC
0.29	0.42	0.2	0	0	IS
0.27	0.37	0.14	0	0	IS
0.25	0.36	0.17	0	0	OC
0.31	0.53	0.3	0	0	PL
0.36	0.46	0.16	0	0	PL
0.4	0.48	0.15	0	0	PL
0.31	0.36	0.08	0	0	IS
0.33	0.41	0.13	0	0	IS
0.27	0.32	0.09	0	0	PL
0.43	0.64	0.39	0	0	IS
0.67	0.92	0.58	0	0	IS
0.79	0.89	0.38	0	0	IS

0.38	0.73	0.47	0	0	IS
0.69	0.9	0.38	0	0	IS
0.26	0.36	0.18	30	12.09	IS
1.23	1.56	0.59	0	0	IS
0.52	0.83	0.54	0	0	IS
0.63	0.77	0.33	0	0	IS
0.57	0.75	0.37	0	0	IS
0.43	0.55	0.2	0	0	IS
0.32	0.7	0.49	0	0	OC
0.32	0.69	0.46	0	0	PL
0.36	0.61	0.38	0	0	IS
0.42	0.56	0.29	0	0	IS
0.52	0.66	0.32	0	0	IS
0.42	0.62	0.38	0	0	IS
0.34	0.49	0.25	0	0	IS
0.43	0.61	0.28	0	0	IS
0.42	0.59	0.31	0	0	IS
0.34	0.64	0.4	0	0	PL
0.43	0.71	0.37	0	0	IS
0.45	0.65	0.3	0	0	IS
0.47	0.63	0.22	0	0	PL
0.67	0.81	0.38	0	0	IS
0.42	0.54	0.27	0	0	IS
1.55	1.93	0.85	0	0	BS
1.65	2.07	0.59	0	0	IS
0.39	0.52	0.26	0	0	IS
0.41	0.49	0.16	0	0	PL
1.65	1.88	0.47	0	0	IS
1.42	1.82	0.69	0	0	IS
1.64	1.93	0.75	0	0	IS
1.64	1.91	0.56	0	0	IS
1.92	2.09	0.3	0	0	IS
0.37	0.63	0.42	0	0	PL
0.42	0.62	0.33	0	0	PL
1.88	2.23	0.55	0	0	IS
1.92	2.24	0.85	0	0	IS
2.04	2.58	0.71	0	0	IS
0.4	0.45	0.2	0	0	IS
0.36	0.44	0.12	0	0	PL
0.39	0.44	0.12	0	0	PL
0.29	0.52	0.27	0	0	IS
0.36	0.43	0.19	0	0	IS
0.33	0.43	0.22	0	0	IS
0.28	0.42	0.21	0	0	PL
0.43	0.54	0.24	0	0	IS
0.39	0.5	0.22	0	0	PL
1.65	2.58	1.17	0	0	IS
0.37	0.43	0.09	0	0	HC
0.98	1.13	0.38	0	0	PL
1.04	1.2	0.29	0	0	IS
1.07	1.18	0.28	0	0	PL
1.04	1.14	0.42	0	0	PL
0.78	1.19	0.82	0	0	IS
0.98	1.16	0.6	0	0	PL

1.79	2.06	0.52	0	0	IS
1.72	2.08	0.68	0	0	IS
1.79	2.45	0.93	0	0	IS
1.07	1.46	0.87	0	0	PL
1.88	2.38	1.04	0	0	IS
0.96	1.21	0.37	0	0	IS
0.88	1.08	0.73	0	0	PL
0.89	1.24	0.79	0	0	PL
1.04	1.48	0.82	0	0	IS
0.79	1.18	0.84	0	0	IS
0.7	1.73	1.42	0	0	OC
0.92	1.19	0.58	0	0	IS
0.93	1.51	0.89	0	0	PL
1.17	1.32	0.36	0	0	IS
1.55	1.77	0.5	0	0	IS
1.26	1.35	0.14	0	0	PL
1.34	1.4	0.11	0	0	PL
0.94	1.38	0.74	0	0	IS
0.64	1.05	0.83	0	0	PL
0.71	1.23	0.82	0	0	PL
0.71	1.29	0.83	0	0	PL
0.44	1.3	0.94	0	0	PL
0.58	1.19	0.96	0	0	PL
0.47	1.19	0.98	0	0	PL
0.62	1	0.58	0	0	PL
0.49	0.96	0.81	0	0	PL
0.34	1.17	1.09	0	0	PL
1.3	1.67	1.02	0	0	IS
0.63	1.27	0.94	0	0	IS
0.79	1.05	0.65	0	0	PL
0.68	1.05	0.59	0	0	IS
0.76	1.04	0.55	0	0	PL
0.78	1.15	0.89	0	0	PL
0.79	1.08	0.72	0	0	PL
0.66	1.03	0.58	0	0	PL
1.31	1.67	0.69	0	0	IS
0.59	1.03	0.74	0	0	PL
0.94	1.1	0.41	0	0	PL
0.79	0.94	0.48	0	0	PL
0.82	1.46	1.06	0	0	IS
0.75	1.25	0.73	0	0	IS
0.63	1.16	0.83	0	0	IS
1.48	2.04	1.03	0	0	IS
0.82	1.34	0.79	0	0	IS
0.96	1.21	0.42	0	0	PL
0.8	1.07	0.59	0	0	PL
0.97	1.08	0.58	0	0	PL
0.88	1.25	0.89	0	0	PL
0.76	1.41	1.1	0	0	OH
0.59	1.47	1.24	0	0	PL
1.07	1.36	0.79	0	0	PL
1.09	1.31	0.58	0	0	PL
1.25	1.58	0.55	0	0	IS
1.26	1.39	0.3	0	0	IS

1.39	1.63	0.64	0	0	IS
0.27	0.85	0.67	0	0	PL
1.49	1.77	0.53	0	0	IS
1.86	2.12	0.67	0	0	IS
1.45	1.83	1.05	0	0	IS
1.65	1.89	0.59	0	0	IS
0.42	0.78	0.59	0	0	PL
0.44	0.8	0.64	0	0	PL
0.48	0.98	0.78	0	0	PL
0.39	0.9	0.71	0	0	PL
0.64	0.72	0.17	0	0	OC
0.56	0.68	0.29	0	0	PL
0.39	0.74	0.57	0	0	PL
0.35	0.51	0.72	0	0	PL
0.45	0.82	0.6	0	0	PL
0.5	0.7	0.31	0	0	PL
0.57	0.68	0.22	0	0	PL
0.48	0.59	0.19	0	0	OC
0.49	0.77	0.55	0	0	PL
0.42	0.61	0.34	0	0	PL
1.53	2.08	0.6	0	0	IS
0.42	0.62	0.42	0	0	PL
1.51	1.77	0.44	0	0	IS
0.48	0.63	0.32	0	0	PL
1.28	1.62	0.79	0	0	IS
1.18	1.62	1.1	0	0	IS
1.29	1.58	0.68	0	0	IS
1.38	1.76	0.84	0	0	IS
0.45	0.64	0.29	0	0	IS
1.37	1.76	0.9	0	0	IS
1.39	1.98	0.83	0	0	IS
1.37	1.76	1	0	0	IS
0.99	1.36	0.62	0	0	IS
0.53	0.67	0.36	0	0	IS
0.53	0.72	0.35	0	0	IS
0.49	0.74	0.46	0	0	OC
1.27	1.46	0.37	0	0	IS
1.13	1.4	0.48	0	0	IS
1.26	1.56	0.53	0	0	IS
0.43	0.7	0.4	0	0	IS
1.03	1.67	0.85	0	0	IS
0.5	0.79	0.47	0	0	OC
1.07	1.48	0.87	0	0	IS
0.42	0.87	0.62	0	0	PL
0.47	0.72	0.46	0	0	PL
0.62	0.9	0.63	0	0	OC
0.47	0.93	0.67	0	0	PL
0.5	0.96	0.7	0	0	PL
0.41	0.95	0.76	0	0	PL
0.62	1.07	0.78	0	0	PL
0.78	1.01	0.48	0	0	IS
0.54	0.78	0.55	0	0	PL
1.07	1.47	0.76	0	0	IS
0.79	0.89	0.28	0	0	PL

1.01	1.27	0.55	0	0	IS
0.72	1.09	0.58	0	0	IS
0.73	0.81	0.41	0	0	PL
0.67	0.8	0.23	0	0	PL
0.65	0.85	0.46	0	0	PL
0.73	0.85	0.27	0	0	PL
0.4	0.86	0.64	0	0	PL
0.78	0.88	0.51	0	0	PL
0.45	1	0.72	0	0	OC
1.13	1.59	0.65	0	0	IS
1.92	2.1	0.43	0	0	IS
1.68	2.18	0.67	0	0	IS
0.59	1.01	0.82	0	0	PL
0.45	0.93	0.75	0	0	PL
0.5	0.7	0.46	0	0	PL
1.67	2	0.53	0	0	IS
0.66	0.73	0.17	0	0	OC
0.51	0.66	0.36	0	0	PL
0.42	0.63	0.39	0	0	OC
0.43	0.63	0.4	0	0	PL
0.69	0.82	0.33	0	0	PL
1.71	1.94	0.71	0	0	IS
1.63	1.86	0.62	0	0	IS
1.53	1.91	0.78	0	0	IS
0.41	0.88	0.69	0	0	OC
0.65	0.8	0.39	0	0	PL
0.46	0.82	0.59	0	0	PL
0.66	0.83	0.38	0	0	PL
0.61	0.92	0.61	0	0	PL
0.53	1.14	0.84	0	0	PL
0.66	1.06	0.72	0	0	PL
1.43	1.91	0.82	0	0	IS
0.67	0.87	0.48	0	0	PL
1.72	2.07	0.52	0	0	IS
0.37	0.46	1.02	0	0	PL
0.6	1.06	0.79	0	0	PL
0.56	1.08	0.84	0	0	PL
0.58	1.07	0.85	0	0	OC
0.7	1.01	0.56	0	0	PL
0.66	0.88	0.43	0	0	PL
0.65	1.01	0.73	0	0	PL
0.42	1	0.75	0	0	OC
0.52	0.79	0.51	0	0	PL
1.67	1.91	0.54	0	0	IS
0.69	0.91	0.52	0	0	PL
0.68	0.93	0.55	0	0	HC
1.44	1.74	0.75	0	0	IS
0.67	0.82	0.47	0	0	PL
1.47	2.13	0.94	0	0	IS
0.4	0.73	0.52	0	0	PL
0.43	0.64	0.4	0	0	PL
0.5	0.73	0.45	0	0	PL
0.1	0.28	0.07	0	0	IS
0.3	0.34	0.08	0	0	PL

0.27	0.31	0.07	0	0	PL
0.3	0.35	0.09	0	0	PL
0.31	0.48	0.26	0	0	PL
0.31	0.46	0.25	0	0	PL
0.28	0.33	0.12	0	0	PL
0.32	0.37	0.1	0	0	PL
0.41	0.62	0.34	0	0	PL
0.32	0.43	0.2	0	0	PL
0.33	0.36	0.08	0	0	PL
0.56	0.85	0.5	0	0	PL
0.43	0.57	0.26	0	0	PL
0.49	0.6	0.22	0	0	PL
1.7	1.99	0.66	0	0	IS
0.62	0.89	0.47	0	0	PL
0.98	1.36	0.78	0	0	PL
1.66	1.96	0.68	0	0	IS
0.63	0.94	0.46	0	0	IS
0.55	0.71	0.29	0	0	PL
1.23	1.36	0.35	0	0	PL
1.2	1.35	0.47	0	0	PL
1.07	1.25	0.35	0	0	PL
0.92	1.23	0.57	0	0	PL
1.7	2.02	0.72	0	0	PL
1.54	1.93	0.8	0	0	PL
1.56	1.94	0.9	0	0	PL
0.64	0.81	0.38	0	0	PL
0.69	0.79	0.21	0	0	PL
0.76	0.98	0.43	0	0	PL
0.57	0.97	0.55	0	0	PL
0.68	0.88	0.5	0	0	PL
0.49	0.64	0.28	0	0	PL
0.87	1.19	0.62	0	0	PL
0.75	0.87	0.3	0	0	PL
0.82	1.07	0.41	0	0	PL
0.8	1.17	0.67	0	0	PL
0.92	1.31	0.29	0	0	PL
0.75	1.29	0.92	0	0	PL
1.02	1.61	1.03	0	0	HC
1.23	1.47	0.72	0	0	PL
0.8	1.25	0.83	0	0	PL
1.17	1.25	0.17	0	0	PL
0.94	1.21	0.66	0	0	PL
1.03	1.3	0.64	0	0	PL
1.18	1.43	0.68	0	0	PL
1.2	1.33	0.33	0	0	PL
0.87	1.14	0.56	0	0	PL
0.35	0.43	0.15	0	0	PL
0.27	0.35	0.12	0	0	PL
0.31	0.42	0.18	0	0	PL
0.36	0.51	0.28	0	0	PL
0.72	0.94	0	0	0	PL
0.72	0.82	0.21	0	0	PL
0.33	0.44	0.22	0	0	PL
0.37	0.56	0.26	0	0	PL

0.36	0.43	0.15	0	0	PL
0.31	0.36	0.09	0	0	PL
0.53	0.65	0.22	0	0	PL
0.28	0.37	0.15	0	0	PL
0.36	0.42	0.14	0	0	PL
0.3	0.36	0.14	0	0	PL
1.11	1.27	0.38	0	0	PL
0.57	1.02	0.66	0	0	PL
1.06	1.26	0.5	0	0	PL
1.64	1.92	0.69	0	0	PL
0.7	1.26	0.94	0	0	PL
0.94	1.26	0.54	0	0	PL
0.41	0.63	0.33	0	0	PL
0.46	0.65	0.31	0	0	PL
0.48	0.63	0.29	0	0	PL
0.5	0.68	0.34	0	0	PL
0.38	0.61	0.36	0	0	PL
0.37	0.55	0.29	0	0	PL
0.41	0.56	0.24	0	0	PL
0.35	0.45	0.27	0	0	PL
0.38	0.49	0.19	0	0	PL
0.28	0.37	0.17	0	0	PL
0.24	0.36	0.2	0	0	PL
0.24	0.36	0.21	0	0	PL
0.29	0.33	0.09	0	0	PL
0.31	0.37	0.12	0	0	PL
0.2	0.26	0.12	0	0	CC
0.33	0.4	0.15	0	0	PL
0.25	0.33	0.15	0	0	PL
0.39	0.47	0.18	0	0	PL
0.35	0.56	0.36	0	0	PL
0.4	0.6	0.31	0	0	PL
0.4	0.61	0.33	0	0	PL
0.35	0.53	0.3	0	0	PL
0.4	0.5	0.18	0	0	PL
0.34	0.43	0.16	0	0	PL
0.23	0.3	0.13	0	0	PL
0.29	0.38	0.16	0	0	PL
0.3	0.37	0.13	0	0	PL
0.25	0.36	0.17	0	0	PL
0.47	0.72	0.4	0	0	PL
0.66	0.73	0.35	0	0	PL
0.52	0.82	0.18	0	0	PL
0.41	0.54	0.22	0	0	PL
0.29	0.33	0.08	0	0	PL
0.31	0.42	0.17	0	0	PL
0.4	0.54	0.27	0	0	PL
0.35	0.43	0.17	0	0	PL
0.28	0.54	0.33	0	0	PL
0.34	0.39	0.12	0	0	PL
0.74	0.83	0.27	0	0	PL
0.76	0.85	0.17	0	0	PL
0.48	0.94	0.64	0	0	PL
0.44	0.78	0.54	0	0	PL

0.71	0.78	0.21	0	0	PL
0.5	0.67	0.32	0	0	PL
0.56	0.73	0.33	0	0	PL
0.62	0.7	0.2	0	0	PL
0.62	0.96	0.59	0	0	PL
0.56	0.79	0.41	0	0	PL
0.48	0.8	0.44	0	0	PL
0.5	0.72	0.37	0	0	PL
0.75	0.93	0.35	0	0	PL
0.61	0.84	0.43	0	0	PL
0.6	0.74	0.33	0	0	PL
0.65	0.72	0.17	0	0	PL
0.44	0.83	0.6	0	0	PL
0.49	0.66	0.32	0	0	PL
0.55	0.82	0.49	0	0	PL
0.58	0.91	0.52	0	0	PL
0.69	0.79	0.21	0	0	PL
0.38	0.48	0.17	0	0	PL
0.46	0.67	0.35	0	0	PL
0.46	0.56	0.2	0	0	IS
0.42	0.52	0.2	0	0	PL
0.39	0.55	0.26	0	0	PL
0.31	0.41	0.18	0	0	PL
0.23	0.34	0.16	0	0	PL
0.24	0.31	0.1	0	0	PL
0.29	0.36	0.15	0	0	PL
0.36	0.47	0.19	0	0	PL
0.3	0.41	0.18	0	0	PL
0.28	0.38	0.18	0	0	PL
0.36	0.42	0.13	0	0	PL
0.36	0.41	0.11	0	0	PL
0.27	0.34	0.13	0	0	PL
0.23	0.29	0.11	0	0	PL
0.28	0.48	0.29	0	0	PL
0.36	0.41	0.08	0	0	PL
0.17	0.22	0.08	0	0	PL
0.16	0.27	0.16	0	0	PL
0.19	0.23	0.06	0	0	PL
0.25	0.39	0.22	0	0	PL
0.21	0.27	0.11	0	0	PL
0.33	0.43	0.2	0	0	PL
0.26	0.37	0.15	0	0	PL
0.3	0.34	0.11	0	0	PL
0.22	0.27	0.09	0	0	PL
0.16	0.24	0.13	0	0	PL
0.16	0.22	0.1	0	0	PL
0.15	0.2	0.09	0	0	PL
0.18	0.36	0.25	0	0	PL
0.2	0.35	0.22	0	0	PL
0.21	0.46	0.33	0	0	PL
0.35	0.4	0.09	0	0	PL
0.29	0.42	0.58	0	0	PL
0.35	0.51	0.26	0	0	PL
0.38	0.51	0.24	0	0	PL

0.42	0.53	0.19	0	0	PL
0.41	0.67	0.36	0	0	PL
0.44	0.63	0.32	0	0	PL
0.48	0.63	0.35	0	0	PL
0.38	0.44	0.14	0	0	PL
0.33	0.54	0.33	0	0	PL
0.3	0.52	0.36	0	0	PL
0.34	0.43	0.16	0	0	PL
0.29	0.43	0.22	0	0	PL
0.36	0.6	0.44	0	0	PL
0.32	0.39	0.13	0	0	PL
0.33	0.38	0.1	0	0	PL
0.33	0.42	0.15	0	0	PL
0.32	0.41	0.16	0	0	PL
0.21	0.31	0.16	0	0	PL
0.28	0.34	0.08	0	0	PL
0.29	0.35	0.1	0	0	HC
0.19	0.29	0.16	0	0	PL
0.17	0.28	0.16	0	0	PL
0.16	0.25	0.13	0	0	OC
0.16	0.25	0.13	0	0	PL
0.15	0.19	0.08	0	0	PL
0.18	0.31	0.2	0	0	PL
0.31	0.38	0.15	0	0	PL
0.2	0.4	0.28	0	0	PL
0.25	0.4	0.22	0	0	PL
0.32	0.45	0.22	0	0	PL
0.17	0.33	0.21	0	0	OC
0.32	0.36	0.08	0	0	IS
0.29	0.43	0.23	0	0	PL
0.33	0.42	0.19	0	0	PL
0.31	0.39	0.17	0	0	PL
0.17	0.23	0.11	0	0	PL
0.15	0.29	0.2	0	0	PL
0.13	0.19	0.09	0	0	PL
0.19	0.32	0.19	0	0	PL
0.17	0.34	0.24	0	0	PL
0.16	0.22	0.09	0	0	PL
0.14	0.18	0.07	0	0	PL
0.24	0.28	0.07	0	0	HC
0.17	0.25	0.13	0	0	PL
0.21	0.32	0.18	0	0	PL
0.19	0.26	0.12	0	0	PL
0.23	0.33	0.17	0	0	PL
0.28	0.35	0.13	0	0	PL
0.19	0.29	0.16	0	0	PL
0.23	0.27	0.1	0	0	PL
0.38	0.52	0.25	0	0	PL
0.37	0.65	0.43	0	0	PL
0.27	0.49	0.29	0	0	PL
0.29	0.46	0.28	0	0	PL
0.26	0.33	0.15	0	0	PL
0.33	0.39	0.14	0	0	PL
0.26	0.39	0.2	0	0	PL

0.16	0.26	0.15	0	0	PL
0.17	0.24	0.12	0	0	PL
0.3	0.39	0.16	0	0	PL
0.26	0.32	0.1	0	0	HC
0.22	0.31	0.14	0	0	PL
0.2	0.29	0.15	0	0	PL
0.19	0.31	0.17	0	0	PL
0.19	0.28	0.13	0	0	PL
0.16	0.19	0.06	0	0	OC
0.17	0.22	0.08	0	0	PL
0.26	0.33	0.15	0	0	PL
0.25	0.33	0.14	0	0	PL
0.23	0.39	0.23	0	0	PL
0.24	0.3	0.12	0	0	PL
0.26	0.33	0.13	0	0	PL
0.19	0.29	0.16	0	0	OC
0.26	0.41	0.22	0	0	PL
0.25	0.45	0.29	0	0	PL
0.23	0.35	0.18	0	0	OC
0.28	0.45	0.26	0	0	PL
0.23	0.37	0.21	0	0	PL
0.24	0.42	0.25	0	0	PL
0.36	0.56	0.31	0	0	PL
0.27	0.41	0.25	0	0	PL
0.25	0.52	0.37	0	0	PL
0.3	0.56	0.37	0	0	PL
0.32	0.43	0.21	0	0	PL
0.36	0.58	0.35	0	0	PL
0.34	0.43	0.18	0	0	PL
0.41	0.58	0.32	0	0	PL
0.36	0.45	0.23	0	0	HC
0.45	0.51	0.14	0	0	PL
0.32	0.36	0.09	0	0	PL
0.29	0.4	0.19	0	0	PL
0.36	0.61	0.39	0	0	PL
0.36	0.54	0.29	0	0	PL
0.38	0.5	0.2	0	0	PL
0.42	0.49	0.24	0	0	PL
0.44	0.66	0.33	0	0	PL
0.49	0.57	0.16	0	0	PL
0.39	0.63	0.4	0	0	PL
0.31	0.7	0.41	0	0	PL
0.5	0.71	0.4	0	0	PL
0.36	0.52	0.28	0	0	PL
0.27	0.4	0.22	0	0	PL
0.24	0.41	0.25	0	0	PL
0.34	0.54	0.3	0	0	PL
0.35	0.45	0.2	0	0	PL
0.28	0.59	0.41	0	0	PL
0.24	0.37	0.45	0	0	PL
0.33	0.51	0.25	0	0	PL
0.33	0.46	0.23	0	0	PL
0.42	0.5	0.19	0	0	PL
0.2	0.53	0.39	0	0	PL

0.44	0.51	0.14	0	0	HC
0.36	0.46	0.24	0	0	PL
0.5	0.64	0.3	0	0	PL
0.42	0.64	0.37	0	0	PL
0.58	0.66	0.21	0	0	PL
0.41	0.84	0.54	0	0	PL
1.69	1.97	0.82	0	0	IS
0.4	0.78	0.54	0	0	PL
0.49	0.69	0.34	0	0	PL
0.47	0.67	0.36	0	0	PL
0.75	0.85	0.23	0	0	PL
1.69	1.9	0.52	0	0	IS
0.56	0.76	0.37	0	0	PL
0.26	0.5	0.67	0	0	PL
0.34	0.81	0.61	0	0	PL
0.52	0.64	0.26	0	0	PL
0.42	0.7	0.46	0	0	PL
0.49	0.76	0.38	0	0	PL
0.38	0.58	0.33	0	0	PL
0.73	0.85	0.28	0	0	PL
0.75	0.91	0.36	0	0	PL
0.69	0.81	0.28	0	0	PL
0.71	0.86	0.39	0	0	PL
0.55	0.97	0.64	0	0	IS
0.57	0.73	0.31	0	0	PL
0.37	0.61	0.4	0	0	PL
0.52	0.63	0.32	0	0	PL
0.43	0.81	0.57	0	0	PL
0.56	0.73	0.3	0	0	PL
0.6	0.68	0.22	0	0	PL
0.78	1.06	0.39	0	0	PL
0.64	1.12	0.74	0	0	PL
0.7	0.9	0.42	0	0	PL
0.76	0.95	0.39	0	0	PL
0.68	0.97	0.58	0	0	PL
0.89	0.97	0.25	0	0	PL
0.79	1.05	0.72	0	0	PL
0.61	0.94	0.53	0	0	PL
0.69	1.03	0.54	0	0	PL
0.71	0.87	0.39	0	0	PL
1.55	1.91	0.76	0	0	IS
0.2	0.55	1.03	0	0	PL
0.51	0.93	0.6	0	0	PL
0.2	0.27	0.12	0	0	PL
0.29	0.33	0.1	0	0	PL
0.21	0.29	0.12	0	0	PL
0.21	0.31	0.16	0	0	PL
0.26	0.33	0.13	0	0	PL
0.32	0.45	0.23	0	0	PL
0.29	0.36	0.14	0	0	PL
0.28	0.37	0.16	0	0	PL
0.34	0.44	0.17	0	0	PL
0.4	0.5	0.19	0	0	PL
0.36	0.46	0.19	0	0	PL

0.27	0.39	0.19	0	0	PL
0.28	0.4	0.2	0	0	PL
0.36	0.41	0.09	0	0	PL
0.25	0.4	0.23	0	0	PL
0.33	0.38	0.15	0	0	PL
0.2	0.31	0.15	0	0	PL
0.22	0.28	0.2	0	0	PL
0.23	0.31	0.12	0	0	PL
0.22	0.34	0.17	0	0	PL
0.23	0.31	0.14	0	0	PL
0.24	0.36	0.19	0	0	PL
0.32	0.44	0.2	0	0	PL
0.43	0.5	0.2	0	0	PL
0.33	0.42	0.18	0	0	PL
0.38	0.49	0.22	0	0	PL
0.34	0.63	0.44	0	0	PL
0.37	0.5	0.24	0	0	PL
0.33	0.52	0.3	0	0	PL
0.28	0.57	0.28	0	0	PL
0.37	0.43	0.15	0	0	PL
0.42	0.48	0.13	0	0	PL
0.46	0.6	0.28	0	0	PL
0.36	0.59	0.34	0	0	PL
0.39	0.54	0.27	0	0	PL
0.47	0.64	0.37	0	0	PL
0.45	0.59	0.25	0	0	PL
0.42	0.69	0.49	0	0	PL
0.29	0.72	0.57	0	0	PL
0.27	0.36	0.17	0	0	PL
0.3	0.39	0.16	0	0	PL
0.25	0.34	0.19	0	0	PL
0.38	0.51	0.25	0	0	HC
0.4	0.47	0.16	0	0	PL
0.47	0.59	0.25	0	0	PL
0.33	0.47	0.24	0	0	PL
0.3	0.43	0.24	0	0	PL
0.39	0.7	0.4	0	0	PL
0.29	1.15	0.51	0	0	PL
0.47	0.85	0.53	0	0	PL
0.41	0.71	0.47	0	0	PL
0.42	0.6	0.28	0	0	PL
0.42	0.5	0.18	0	0	PL
0.44	0.57	0.48	0	0	PL
0.73	1.01	0.58	0	0	PL
0.32	0.6	0.67	0	0	PL
0.52	0.81	0.44	0	0	PL
0.48	0.81	0.52	0	0	PL
0.46	0.76	0.45	0	0	PL
0.43	0.69	0.43	0	0	PL
1.59	1.89	0.79	0	0	IS
0.46	0.61	0.26	0	0	PL
0.34	0.78	0.56	0	0	PL
0.47	0.64	0.33	0	0	PL
0.57	0.69	0.25	0	0	PL

0.25	0.37	0.51	0	0	PL
0.3	0.58	0.38	0	0	PL
0.38	0.6	0.34	0	0	PL
0.39	0.71	0.47	0	0	PL
0.62	0.94	0.53	0	0	PL
0.5	0.8	0.46	0	0	PL
1.56	1.84	0.84	0	0	IS
0.84	0.92	0.2	0	0	PL
1.76	1.93	0.46	0	0	IS
1.59	1.86	0.71	0	0	IS
1.59	1.85	0.48	0	0	PL
1.85	2.05	0.69	0	0	IS
1.76	2.09	0.52	0	0	IS
1.83	2.25	0.66	0	0	IS
1.69	1.96	0.55	0	0	IS
1.81	2.25	0.74	0	0	IS
1.96	2.08	0.22	0	0	IS
1.9	2.22	0.46	0	0	IS
1.64	1.97	1.07	0	0	BS
1.92	2.19	0.56	0	0	IS
1.63	1.99	0.79	0	0	PL
1.95	2.2	0.49	0	0	IS
1.77	2.15	0.63	0	0	IS
1.52	1.66	0.35	0	0	IS
1.42	2	0.96	0	0	IS
1.31	1.64	0.57	0	0	IS
1.24	1.59	0.54	0	0	IS
1.51	1.78	0.48	0	0	IS
1.15	1.61	0.87	0	0	IS
1.5	1.74	0.48	0	0	IS
1.52	1.65	0.32	0	0	IS
1.46	1.74	0.64	0	0	IS
1.15	1.23	0.21	0	0	PL
1.03	1.35	0.66	0	0	PL
1.14	1.52	0.82	0	0	PL
1.12	1.23	0.38	0	0	PL
1.52	1.69	0.43	0	0	IS
1.18	1.37	0.44	0	0	PL
1.14	1.36	0.55	0	0	PL
0.73	0.92	0.34	0	0	PL
1.27	1.42	0.42	0	0	PL
1.27	1.89	0.84	0	0	IS
1.3	1.49	0.42	0	0	PL
1.54	1.72	0.75	0	0	IS
1.39	1.77	0.83	0	0	IS
1.29	1.55	0.6	0	0	IS
1.35	1.57	0.39	0	0	IS
1.43	1.66	0.5	0	0	PL
1.72	1.85	0.35	0	0	IS
1.48	1.67	0.51	0	0	IS
1.38	1.6	0.53	0	0	PL
1.44	1.65	0.56	0	0	PL
1.41	1.54	0.43	0	0	IS
1.5	1.78	0.57	0	0	PL

1.29	1.5	0.44	0	0	IS
1.57	1.91	0.88	0	0	PL
1.14	1.32	0.45	0	0	IS
0.87	1.07	0.39	0	0	IS
1.85	2.16	0.6	0	0	IS
1.04	1.15	0.28	0	0	IS
1.77	2.1	0.54	0	0	IS
0.83	1.24	0.77	0	0	IS
0.97	1.18	0.46	0	0	IS
0.99	1.27	0.48	0	0	IS
1.42	1.65	0.44	0	0	IS
1.6	1.77	0.37	0	0	PL
1.2	1.34	0.43	0	0	IS
1.21	1.47	0.66	0	0	IS
0.81	1.17	0.54	0	0	IS
1.51	1.64	0.35	0	0	PL
1.54	1.86	0.43	0	0	IS
0.8	1.2	0.68	0	0	IS
1.03	1.19	0.3	0	0	IS
1.7	1.83	0.28	0	0	PL
0.87	1.18	0.5	0	0	IS
1.4	1.79	0.96	0	0	PL
1.02	1.26	0.62	0	0	IS
0.78	1.38	1.03	0	0	IS
1.02	1.45	0.88	0	0	PL
1.16	1.45	0.55	0	0	PL
1.11	1.48	0.7	0	0	IS
1.14	1.5	0.63	0	0	IS
1.24	1.68	0.76	0	0	IS
1.38	1.82	0.95	0	0	PL
1.35	1.62	0.55	0	0	IS
1.24	1.57	0.79	0	0	IS
1.46	1.7	0.5	0	0	PL
1.36	1.78	0.74	0	0	PL
1.37	1.59	0.42	0	0	IS
1.13	1.43	0.58	0	0	IS
1.39	1.64	0.47	0	0	PL
1.29	1.55	0.63	0	0	PL
1.41	1.61	0.45	0	0	PL
1.19	1.58	0.77	0	0	PL
1.02	1.36	0.74	0	0	PL
1.42	1.64	0.42	0	0	IS
1.33	1.46	0.37	0	0	PL
1.27	1.58	0.59	0	0	IS
1.03	1.74	1.01	0	0	IS
1.36	1.53	0.42	0	0	PL
1.03	1.56	0.79	0	0	IS
0.72	0.83	0.28	0	0	IS
1.78	2.17	0.74	0	0	IS
0.89	1.13	0.45	0	0	IS
0.8	1.34	0.79	0	0	IS
0.58	0.97	0.59	0	0	IS
1.07	1.49	0.91	0	0	IS
1.01	1.74	1.18	0	0	IS

1.56	1.82	0.85	0	0	IS
1.51	1.75	0.62	0	0	BS
1.11	1.39	0.59	0	0	IS
1.1	1.28	0.5	0	0	IS
1.05	1.44	0.62	0	0	IS
1.03	1.35	0.61	0	0	IS
0.98	1.41	0.82	0	0	IS
1.03	1.53	0.85	0	0	IS
0.9	1.5	0.77	0	0	BS
1.15	1.3	0.59	0	0	IS
0.94	1.32	0.78	0	0	IS
1.07	1.65	0.9	0	0	IS
1.03	1.76	1.2	0	0	IS
1.03	1.41	0.89	0	0	BS
1.33	1.62	0.69	0	0	IS
1.48	1.63	0.48	0	0	IS
1.8	2.14	0.63	0	0	IS
1.52	1.78	0.74	0	0	IS
1.58	1.84	0.53	0	0	IS
2	2.39	0.67	0	0	IS
1.64	1.97	0.5	0	0	IS
1.27	1.7	1.09	0	0	IS
1.32	1.65	0.46	0	0	IS
1.23	1.62	0.78	0	0	IS
1.32	1.6	0.59	0	0	BS
1.51	1.85	0.55	0	0	IS
1.42	1.74	0.63	0	0	IS
1.54	1.98	0.86	0	0	IS
1.37	1.86	0.87	0	0	IS
1.41	1.94	0.77	0	0	IS
1.84	1.98	0.27	0	0	IS
1.52	1.81	0.71	0	0	IS
1.29	1.74	1.02	0	0	IS
1.02	1.69	0.92	0	0	IS
1.19	1.75	0.78	0	0	IS
0.98	1.42	0.75	0	0	BS
1.26	1.72	0.82	0	0	IS
1.48	1.73	0.5	0	0	IS
1.5	1.68	0.34	0	0	IS
1.4	1.68	0.6	0	0	IS
1.42	1.75	0.6	0	0	BS
1.3	1.86	0.85	0	0	IS
1.48	1.89	0.9	0	0	IS
1.46	1.97	1.01	0	0	IS
1.17	1.58	0.64	0	0	IS
1.21	1.49	0.7	0	0	IS
1.2	1.38	0.59	0	0	PL
1.01	1.31	0.59	0	0	PL
1.31	1.46	0.32	0	0	PL
1.38	1.55	0.37	0	0	PL
1.3	1.47	0.39	0	0	PL
1.29	2	1.33	0	0	IS
1.4	1.61	0.39	0	0	PL
1.53	1.81	0.74	0	0	IS

1.47	1.74	0.9	0	0	BS
1.14	1.25	0.29	0	0	PL
0.7	1.13	0.7	0	0	PL
1.02	1.25	0.38	0	0	PL
0.97	1.15	0.41	0	0	PL
1.26	2.06	1.29	0	0	IS
0.82	1.17	0.7	0	0	PL
1.31	1.56	0.55	0	0	IS
1.03	1.21	0.41	0	0	PL
1.12	1.29	0.33	0	0	PL
1.17	1.4	0.48	0	0	PL
1.2	1.33	0.28	0	0	PL
1.18	1.64	0.8	0	0	IS
0.87	1.37	0.91	0	0	PL
0.94	2.12	1.57	0	0	IS
1.17	1.41	0.56	0	0	PL
0.96	1.64	1.28	0	0	PL
1.1	1.28	0.41	0	0	PL
1.61	2.21	1.19	0	0	IS
1.15	1.25	0.26	0	0	PL
1.08	1.24	0.35	0	0	PL
0.88	1.24	0.79	0	0	PL
1.15	1.32	0.41	0	0	PL
1.6	1.86	0.44	0	0	IS
1.15	1.25	0.24	0	0	PL
1.12	1.35	0.5	0	0	PL
0.96	1.21	0.58	0	0	PL
1.06	1.29	0.55	0	0	PL
1.05	1.27	0.55	0	0	PL
1.64	2.38	1.4	0	0	IS
0.92	1.21	0.57	0	0	PL
0.9	1.16	0.6	0	0	PL
1.08	1.39	0.57	0	0	PL
0.77	1.15	0.57	0	0	PL
0.99	1.19	0.56	0	0	PL
0.69	0.87	0.35	0	0	PL
0.96	1.06	0.22	0	0	PL
0.69	1.02	0.59	0	0	PL
0.98	1.1	0.3	0	0	IS
0.9	1.22	0.63	0	0	PL
0.9	1.08	0.42	0	0	PL
1.31	1.73	1	0	0	IS
1.11	1.29	0.47	0	0	PL
1.01	1.26	0.59	0	0	PL
0.79	1.11	0.62	0	0	PL
0.93	1.06	0.27	0	0	PL
1.39	2.11	1.37	0	0	IS
0.96	1.24	0.62	0	0	PL
0.92	1.39	0.94	0	0	PL
1.01	1.74	1.28	0	0	PL
1.02	1.6	0.7	0	0	PL
0.85	1.24	0.72	0	0	PL
1.15	1.3	0.48	0	0	PL
1.08	1.75	1.3	0	0	PL

1.16	1.66	1.14	0	0	PL
1.06	1.48	0.77	0	0	PL
1.25	1.42	0.37	0	0	PL
1.13	1.37	0.54	0	0	PL
1.62	1.91	0.68	0	0	IS
0.94	1.3	0.67	0	0	PL
1.14	1.36	0.44	0	0	PL
1.03	1.36	0.74	0	0	PL
0.93	1.32	0.73	0	0	HC
1	1.25	0.48	0	0	IS
1.09	1.3	0.37	0	0	PL
0.87	1.51	1.06	0	0	PL
0.97	1.32	0.64	0	0	PL
0.72	1.25	0.88	0	0	PL
0.83	1.18	0.67	0	0	PL
1.57	1.81	0.65	0	0	IS
0.93	1.42	0.97	0	0	IS
0.71	1	0.68	0	0	PL
1.04	1.12	0.24	0	0	PL
0.97	1.06	0.21	0	0	PL
0.82	1.06	0.6	0	0	PL
0.82	1.07	0.58	0	0	PL
1.03	1.37	0.78	0	0	PL
1.02	1.37	0.71	0	0	PL
1	1.3	0.6	0	0	PL
1.05	1.28	0.61	0	0	PL
1.05	1.44	0.65	0	0	PL
0.98	1.47	0.86	0	0	PL
1.17	1.29	0.28	0	0	PL
1.18	1.41	0.41	0	0	IS
1.22	1.43	0.59	0	0	PL
1	1.29	0.71	0	0	BS
1.32	1.57	0.48	0	0	PL
1.15	1.4	0.75	0	0	PL
1.26	1.52	0.54	0	0	PL
1.28	1.49	0.43	0	0	PL
1.23	1.43	0.55	0	0	PL
0.98	1.35	0.68	0	0	IS
1.26	1.59	0.64	0	0	PL
1.45	1.58	0.23	0	0	PL
1.49	1.63	0.3	0	0	PL
1.46	1.62	0.46	0	0	PL
1.24	1.65	0.84	0	0	PL
1.3	2.02	1.49	0	0	PL
1.44	1.88	1.02	0	0	PL
1.54	1.93	0.76	0	0	PL
1.34	1.61	0.52	0	0	PL
1.58	1.92	0.57	0	0	PL
1.47	1.84	0.89	0	0	IS
1.31	1.51	0.55	0	0	PL
1.52	1.95	0.65	0	0	IS
0.92	1.49	0.99	0	0	PL
0.84	1.01	0.42	0	0	IS
1.33	1.58	0.55	0	0	PL

1.29	1.53	0.57	0	0	PL
1.35	1.59	0.49	0	0	PL
1.25	1.49	0.6	0	0	PL
0.69	1.34	1.04	0	0	IS
1.26	1.51	0.63	0	0	PL
1.31	1.55	0.49	0	0	PL
1.02	1.33	0.59	0	0	IS
1.3	1.53	0.41	0	0	PL
0.95	1.37	0.63	0	0	IS
1.07	1.55	0.82	0	0	IS
0.94	1.55	0.79	0	0	IS
0.87	1.49	0.99	0	0	IS
1.1	1.55	0.77	0	0	IS
1.01	1.11	0.23	0	0	PL
0.83	1.04	0.43	0	0	PL
0.72	0.97	0.55	0	0	PL
0.88	1.06	0.45	0	0	PL
0.69	0.87	0.35	0	0	PL
0.77	0.91	0.34	0	0	PL
0.87	0.98	0.44	0	0	PL
0.78	1.08	0.53	0	0	PL
0.59	0.98	0.55	0	0	PL
0.67	1.07	0.74	0	0	IS
0.78	0.94	0.25	0	0	PL
0.68	0.93	0.47	0	0	PL
0.7	0.85	0.36	0	0	PL
0.89	1.09	0.39	0	0	PL
0.87	1.29	0.87	0	0	PL
0.81	1.02	0.45	0	0	PL
0.81	0.99	0.37	0	0	PL
0.87	1.01	0.32	0	0	PL
0.58	0.91	0.62	0	0	PL
0.88	1.03	0.24	0	0	PL
0.78	1	0.48	0	0	PL
0.72	0.9	0.38	0	0	PL
0.82	1.25	0.74	0	0	IS
0.71	1.1	0.75	0	0	PL
0.84	1.11	0.5	0	0	PL
0.83	1.11	0.54	0	0	PL
0.74	0.99	0.55	0	0	HC
0.96	1.19	0.61	0	0	PL
0.8	1.48	1.14	0	0	PL
0.82	1.09	0.48	0	0	PL
0.98	1.25	0.51	0	0	PL
0.95	1.11	0.37	0	0	PL
0.95	1.13	0.38	0	0	PL
0.92	1.1	0.36	0	0	PL
0.91	1.05	0.3	0	0	PL
0.71	1.28	0.86	0	0	PL
0.71	1.12	0.74	0	0	PL
0.78	0.99	0.48	0	0	PL
0.8	1.19	0.91	0	0	PL
0.98	1.36	0.68	0	0	PL
0.93	1.38	0.87	0	0	PL

0.99	1.41	0.74	0	0	PL
0.82	1.12	0.68	0	0	PL
0.82	1.18	0.63	0	0	PL
0.9	1.13	0.49	0	0	PL
1.2	1.4	0.29	0	0	PL
1.02	1.31	0.68	0	0	PL
1.04	1.35	0.61	0	0	PL
0.92	1.13	0.38	0	0	PL
0.93	1.19	0.59	0	0	PL
0.92	1.13	0.48	0	0	PL
0.73	1.42	1.05	0	0	PL
1.04	1.28	0.63	0	0	PL
0.94	1.17	0.6	0	0	PL
0.88	1.16	0.23	0	0	PL
0.75	1	0.47	0	0	PL
0.88	1.13	0.61	0	0	PL
0.69	0.98	0.6	0	0	PL
0.59	1.32	1.09	0	0	PL
0.61	0.82	0.42	0	0	PL
0.56	0.71	0.34	0	0	PL
0.56	0.86	0.56	0	0	PL
0.61	1.03	0.63	0	0	PL
0.84	1.22	0.68	0	0	IS
0.98	1.09	0.29	0	0	PL
0.57	0.74	0.3	0	0	PL
0.89	0.96	0.2	0	0	PL
0.79	1.14	0.61	0	0	PL
0.9	1.06	0.32	0	0	PL
0.61	0.85	0.47	0	0	PL
0.74	1.12	0.68	0	0	PL
0.62	0.75	0.26	0	0	PL
0.7	1.22	0.94	0	0	IS
0.36	0.64	0.4	0	0	PL
1.29	1.7	0.66	0	0	PL
0.87	1.03	0.42	0	0	IS
1.23	1.63	0.75	0	0	PL
1.14	1.43	0.54	0	0	IS
1.39	1.82	0.76	0	0	PL
1.34	1.66	0.64	0	0	PL
0.95	1.47	0.83	0	0	IS
0.8	1.47	1.11	0	0	IS
1.27	1.58	0.76	0	0	PL
1.12	1.7	0.92	0	0	PL
1.09	1.47	0.88	0	0	PL
1.32	1.71	0.78	0	0	IS
1.14	1.45	0.6	0	0	PL
0.92	1.02	0.21	0	0	IS
0.66	1.3	0.88	0	0	PL
1.18	1.47	0.75	0	0	PL
1.31	1.46	0.49	0	0	PL
1.24	1.48	0.34	0	0	IS
1.19	1.49	0.52	0	0	PL
1.05	1.33	0.64	0	0	PL
1.03	1.3	0.64	0	0	PL

0.9	1.17	0.51	0	0	PL
1.06	1.21	0.39	0	0	PL
0.94	1.15	0.54	0	0	PL
0.78	1.11	0.55	0	0	PL
1.18	1.41	0.52	0	0	PL
1.25	1.48	0.41	0	0	PL
1.14	1.45	0.72	0	0	PL
1.15	1.65	1.19	0	0	IS
1.2	1.33	0.31	0	0	PL
1.06	1.31	0.47	0	0	IS
1.13	1.43	0.67	0	0	PL
1.14	1.43	0.53	0	0	IS
1.2	1.54	0.54	0	0	IS
1.22	1.38	0.38	0	0	PL
1.27	1.4	0.28	0	0	PL
1.08	1.47	0.57	0	0	IS
1.31	1.57	0.21	0	0	IS
1.16	1.38	0.44	0	0	PL
1.12	1.34	0.42	0	0	IS
1.05	1.33	0.49	0	0	PL
0.75	1.66	1.31	0	0	PL
1.14	1.6	0.92	0	0	IS
1.06	1.46	0.74	0	0	PL
1.15	1.44	0.73	0	0	PL
1.19	1.44	0.49	0	0	IS
1.36	1.68	0.32	0	0	IS
1.43	1.53	0.39	0	0	PL
1.29	1.47	0.39	0	0	IS
1.37	1.56	0.4	0	0	IS
1.22	1.55	0.74	0	0	PL
1.29	1.62	0.63	0	0	IS
1.09	1.5	0.93	0	0	PL
1.32	1.61	0.68	0	0	PL
1.25	1.55	0.52	0	0	IS
1.26	1.66	0.66	0	0	IS
1.31	1.49	0.49	0	0	IS
1.06	1.36	0.78	0	0	BS
1.36	1.6	0.42	0	0	IS
1.31	1.49	0.46	0	0	IS
1.29	1.56	0.57	0	0	IS
1.48	1.6	0.37	0	0	PL
1.31	1.68	0.61	0	0	BS
1.27	1.56	0.63	0	0	PL
0.74	1.27	0.81	0	0	PL
1.24	1.43	0.46	0	0	IS
1.17	1.52	0.72	0	0	IS
1.12	1.29	0.35	0	0	IS
1.17	1.51	0.62	0	0	IS
1.24	1.45	0.47	0	0	IS
0.61	1.65	1.42	0	0	OC
1.14	1.52	0.8	0	0	PL
0.72	1.22	0.76	0	0	OC
1.02	1.31	0.53	0	0	PL
0.81	1.51	1.01	0	0	IS

1.21	1.35	0.34	0	0	IS
0.92	1.29	0.79	0	0	PL
0.97	1.51	0.96	0	0	PL
0.91	1.25	0.59	0	0	IS
1.21	1.45	0.37	0	0	PL
0.75	1.08	0.5	0	0	IS
1.19	1.33	0.42	0	0	PL
0.62	0.82	0.44	0	0	IS
1.19	1.54	0.68	0	0	IS
1.16	1.39	0.59	0	0	PL
0.74	1.23	0.74	0	0	OC
1.02	1.39	0.73	0	0	PL
1.17	1.54	0.3	0	0	PL
0.8	1.26	0.89	0	0	PL
0.97	1.37	0.68	0	0	PL
0.94	1.35	0.85	0	0	PL
1.1	1.32	0.44	0	0	PL
0.89	1.54	1.09	0	0	PL
0.78	1.61	1.28	0	0	PL
1.01	1.64	1.26	0	0	PL
0.75	1.6	1.31	0	0	PL
0.68	1.61	1.34	0	0	OC
0.87	1.5	1.17	0	0	PL
1.28	1.42	0.62	0	0	PL
1.27	1.41	0.38	0	0	PL
1.33	1.55	0.41	0	0	PL
1.04	1.52	0.9	0	0	PL
1.23	1.4	0.7	0	0	PL
0.66	0.88	0.35	0	0	IS
1.09	1.41	0.88	0	0	PL
1.26	1.54	0.17	0	0	IS
1.1	1.36	0.54	0	0	IS
0.73	0.99	0.43	0	0	IS
0.75	0.96	0.52	0	0	IS
1.08	1.36	0.62	0	0	PL
1.37	1.58	0.57	0	0	IS
1.15	1.55	0.83	0	0	IS
1.26	1.58	0.74	0	0	PL
1.01	1.55	0.96	0	0	IS
0.97	1.4	0.96	0	0	PL
1.55	1.82	0.59	0	0	IS
0.76	0.92	0.25	0	0	IS
1.36	1.66	0.66	0	0	PL
1.41	1.7	0.52	0	0	PL
1.44	1.78	0.67	0	0	IS
1.1	1.54	0.99	0	0	IS
0.7	0.92	0.4	0	0	IS
1.64	1.82	0.3	0	0	IS
1.53	1.95	0.71	0	0	IS
0.72	0.85	0.23	0	0	IS
0.52	0.67	0.3	0	0	IS
0.58	0.72	0.28	0	0	IS
1.55	1.75	0.65	0	0	IS
1.6	1.9	0.61	0	0	IS

1.5	1.96	0.76	0	0	IS
1.52	1.86	0.77	0	0	IS
1.62	1.8	0.59	0	0	IS
1.47	1.82	0.58	0	0	IS
0.75	0.93	0.41	0	0	IS
1.41	1.64	0.37	0	0	PL
1.53	1.72	0.42	0	0	IS
1.06	1.15	0.31	0	0	IS
1.51	1.72	0.32	0	0	IS
1.41	1.67	0.47	0	0	IS
1.54	1.84	0.34	0	0	IS
1.45	1.81	0.67	0	0	IS
0.81	0.87	0.12	0	0	IS
1.43	1.64	0.37	0	0	IS
1.42	1.6	0.41	0	0	PL
1.31	1.55	0.41	0	0	IS
1.37	1.7	0.56	0	0	IS
0.92	1.42	0.86	0	0	IS
1.04	1.3	0.55	0	0	IS
1.46	1.74	0.51	0	0	IS
1.6	1.87	0.46	0	0	IS
1.57	1.86	0.52	0	0	IS
1.59	1.76	0.51	0	0	IS
1.62	1.85	0.37	0	0	IS
0.97	1.7	1.16	0	0	IS
1.69	2	0.65	0	0	IS
1.72	2.04	0.62	0	0	IS
1.62	1.85	0.62	0	0	IS
1.67	2	0.38	0	0	IS
0.63	1.04	0.65	0	0	IS
0.69	0.85	0.35	0	0	IS
1.62	2.01	0.73	0	0	PL
0.72	0.92	0.38	0	0	IS
1.65	1.95	0.54	0	0	IS
0.68	0.89	0.44	0	0	IS
1.66	1.84	0.61	0	0	IS
1.67	1.94	0.51	0	0	IS
1.59	1.84	0.46	0	0	IS
1.29	1.76	0.94	0	0	PL
1.65	1.97	0.45	0	0	IS
1.57	1.76	0.59	0	0	IS
0.8	0.97	0.45	0	0	IS
1.49	1.89	0.72	0	0	IS
1.58	1.79	0.49	0	0	IS
0.74	0.96	0.38	0	0	IS
1.45	1.83	0.78	0	0	PL
1.51	1.98	0.75	0	0	IS
1.67	2.16	1.49	0	0	OC
1.63	1.91	0.81	0	0	IS
1.28	1.86	1.06	0	0	OC
1.65	2.06	0.87	0	0	IS
0.66	0.83	0.43	0	0	IS
1.25	1.44	0.64	0	0	PL
1.33	1.67	0.62	0	0	IS

1.54	1.74	0.33	0	0	IS
0.65	0.8	0.28	0	0	IS
1.12	1.58	0.92	0	0	PL
1.31	1.63	0.57	0	0	IS
0.84	0.91	0.21	0	0	IS
0.7	1.22	0.86	0	0	OC
1.42	1.93	0.97	0	0	IS
1.39	1.82	0.83	0	0	IS
1.11	1.56	0.84	0	0	IS
0.72	1.08	0.59	0	0	IS
0.46	1.04	0.65	0	0	IS
1.33	1.66	0.77	0	0	PL
1.1	1.54	0.92	0	0	HC
1.5	1.73	0.53	0	0	PL
1.36	1.65	0.61	0	0	IS
0.79	0.96	0.33	0	0	IS
1.25	1.38	0.41	0	0	IS
1.24	1.62	0.81	0	0	PL
1.43	1.85	0.88	0	0	IS
1.5	1.87	0.74	0	0	IS
1.48	1.76	0.49	0	0	IS
1.43	1.77	0.61	0	0	IS
0.95	1.46	1	0	0	IS
0.87	1.39	0.79	0	0	IS
1.55	1.96	0.62	0	0	IS
1.53	1.84	0.54	0	0	IS
1.56	1.84	0.39	0	0	PL
0.94	1.45	0.91	0	0	IS
1.38	1.84	0.73	0	0	IS
1.43	2.04	1.07	0	0	IS
0.85	1.32	0.76	0	0	IS
1.02	1.56	1.11	0	0	IS
1.36	1.69	0.86	0	0	PL
1.51	1.72	0.45	0	0	PL
1.33	1.67	0.7	0	0	PL
1.01	1.33	0.54	0	0	IS
1.41	1.66	0.6	0	0	PL
1.5	1.76	0.48	0	0	IS
1.33	1.69	0.61	0	0	IS
1.25	1.57	0.8	0	0	PL
1.1	1.32	0.55	0	0	IS
1.4	1.64	0.6	0	0	PL
0.74	1.23	0.91	0	0	IS
1.28	1.82	0.92	0	0	IS
1.41	1.96	1	0	0	PL
1.46	1.9	0.87	0	0	PL
1.82	2.2	0.64	0	0	IS
1.58	2.05	0.98	0	0	PL
1.67	1.88	0.59	0	0	PL
1.62	2.05	0.72	0	0	IS
1.51	2.05	0.88	0	0	IS
1.43	1.94	0.93	0	0	PL
1.1	1.71	1.28	0	0	HC
1.43	1.73	0.68	0	0	IS

1.29	1.83	1.04	0	0	PL
1.64	1.96	0.73	0	0	IS
1.59	1.9	0.56	0	0	IS
1.55	1.95	0.71	0	0	IS
0.85	1.08	0.57	0	0	IS
1.67	1.91	0.71	0	0	IS
0.9	1.01	0.27	0	0	IS
1.38	1.75	0.79	0	0	IS
0.77	0.83	0.11	0	0	IS
1.67	1.83	0.46	0	0	IS
1.59	1.88	0.64	0	0	IS
1.61	1.89	0.5	0	0	IS
1.7	2.09	0.73	0	0	IS
0.6	0.88	0.47	0	0	IS
1.5	1.84	0.76	0	0	PL
0.93	1.25	0.51	0	0	IS
1.08	1.34	0.57	0	0	IS
1.45	1.89	0.71	0	0	IS
1.05	1.47	0.94	0	0	IS
1.51	1.93	0.8	0	0	IS
1.64	2.02	0.79	0	0	IS
1.65	1.96	0.77	0	0	IS
1.76	2.19	0.84	0	0	IS
1.02	1.27	0.56	0	0	IS
0.87	1.3	0.74	0	0	IS
0.9	1.11	0.55	0	0	PL
0.78	1.09	0.44	0	0	PL
0.88	1.15	0.63	0	0	PL
0.77	1	0.48	0	0	PL
0.98	1.11	0.29	0	0	PL
0.79	1.02	0.51	0	0	PL
0.94	1.1	0.38	0	0	PL
1.03	1.2	0.38	0	0	IS
1.13	1.31	0.43	0	0	IS
0.97	1.16	0.41	0	0	IS
1.12	1.19	0.2	0	0	PL
1.01	1.2	0.41	0	0	IS
0.93	1.17	0.64	0	0	PL
1.02	1.21	0.54	0	0	PL
0.94	1.31	0.67	0	0	PL
1.05	1.2	0.45	0	0	PL
0.91	1.03	0.29	0	0	PL
1.02	1.11	0.2	0	0	PL
0.91	1.05	0.29	0	0	IS
0.84	1.08	0.55	0	0	PL
1.01	1.17	0.37	0	0	PL
1.03	1.2	0.39	0	0	PL
1.09	1.16	0.19	0	0	PL
1.11	1.2	0.3	0	0	PL
1.05	1.21	0.28	0	0	PL
0.95	1.18	0.52	0	0	IS
1.13	1.23	0.31	0	0	PL
1.05	1.19	0.41	0	0	PL
1.45	1.81	0.9	0	0	IS

0.79	1.08	0.5	0	0	PL
0.78	1.27	0.77	0	0	PL
0.84	0.98	0.72	0	0	IS
0.83	1.07	0.42	0	0	PL
0.91	1.03	0.26	0	0	PL
0.89	1.07	0.39	0	0	PL
0.77	1.01	0.46	0	0	PL
0.86	1.01	0.37	0	0	PL
0.89	1.01	0.39	0	0	PL
1.5	2.1	1	0	0	IS
1.05	1.32	0.62	0	0	PL
1	1.26	0.56	0	0	IS
1.15	1.33	0.41	0	0	IS
1.1	1.36	0.59	0	0	PL
1.15	1.35	0.47	0	0	PL
1.16	1.35	0.45	0	0	PL
1.13	1.38	0.49	0	0	PL
1.36	1.79	0.85	0	0	IS
1.25	1.38	0.36	0	0	PL
1.25	1.43	0.39	0	0	PL
0.94	1.24	0.59	0	0	PL
1.1	1.32	0.48	0	0	PL
1.17	1.41	0.45	0	0	IS
1.27	1.42	0.35	0	0	PL
1	1.26	0.53	0	0	PL
0.92	1.43	0.9	0	0	PL
0.93	1.18	0.52	0	0	PL
0.89	1.23	0.65	0	0	PL
1.06	1.27	0.7	0	0	PL
0.83	1.22	0.74	0	0	PL
0.99	1.29	0.72	0	0	PL
0.89	1.24	0.61	0	0	PL
1.1	1.48	0.68	0	0	PL
1.21	1.38	0.41	0	0	IS
1.11	1.39	0.49	0	0	PL
0.92	1.13	0.41	0	0	PL
0.97	1.11	0.58	0	0	PL
0.8	1.03	0.48	0	0	PL
0.87	1.07	0.44	0	0	IS
1.05	1.18	0.32	0	0	IS
1	1.13	0.4	0	0	PL
0.91	1.15	0.49	0	0	IS
0.79	1.11	0.54	0	0	PL
0.78	1.12	0.72	0	0	PL
0.75	1.15	0.74	0	0	PL
1.01	1.22	0.46	0	0	IS
0.94	1.18	0.46	0	0	PL
1.02	1.16	0.41	0	0	IS
0.94	1.14	0.46	0	0	PL
0.55	1.06	0.81	0	0	PL
0.86	1.12	0.72	0	0	PL
0.96	1.19	0.48	0	0	IS
0.71	1.26	0.9	0	0	PL
1.22	1.43	0.42	0	0	IS

0.77	1.21	0.89	0	0	PL
0.67	1.22	0.76	0	0	IS
1.02	1.14	0.41	0	0	PL
0.81	1.15	0.73	0	0	PL
0.8	0.92	0.44	0	0	PL
0.74	1.13	0.72	0	0	PL
0.64	1	0.62	0	0	PL
0.9	1.08	0.35	0	0	IS
0.92	1.08	0.37	0	0	IS
0.79	1.06	0.46	0	0	PL
0.76	1.1	0.58	0	0	PL
0.76	1.12	0.73	0	0	PL
0.85	1.06	0.46	0	0	PL
1.02	1.08	0.17	0	0	PL
0.86	1.11	0.46	0	0	PL
0.89	1.15	0.53	0	0	PL
0.93	1.11	0.39	0	0	PL
0.77	1.4	1.02	0	0	PL
0.93	1.08	0.49	0	0	PL
0.96	1.28	0.6	0	0	IS
0.89	1.15	0.62	0	0	PL
0.75	1.14	0.75	0	0	PL
0.59	1.04	0.75	0	0	PL
0.89	1.21	0.8	0	0	PL
0.91	1.23	0.72	0	0	PL
1.06	1.33	0.68	0	0	IS
0.58	0.97	0.7	0	0	PL
0.98	1.12	0.23	0	0	IS
0.97	1.08	0.23	0	0	IS
0.62	1.03	0.69	0	0	IS
0.62	0.95	0.6	0	0	PL
0.64	0.9	0.45	0	0	PL
0.7	0.98	0.53	0	0	PL
0.87	1	0.35	0	0	PL
0.71	0.98	0.54	0	0	PL
0.86	0.96	0.24	0	0	PL
0.64	0.9	0.45	0	0	PL
0.85	1.07	0.61	0	0	PL
0.87	1.1	0.45	0	0	PL
0.89	1.14	0.46	0	0	PL
0.91	1.13	0.45	0	0	IS
0.74	1.01	0.55	0	0	PL
0.78	0.94	0.35	0	0	PL
0.69	0.93	0.52	0	0	PL
0.62	0.94	0.53	0	0	PL
0.78	0.86	0.19	0	0	PL
0.75	0.97	0.43	0	0	PL
0.77	0.9	0.27	0	0	PL
0.79	0.93	0.3	0	0	PL
0.74	0.93	0.36	0	0	PL
0.66	1.13	0.78	0	0	PL
0.81	1.07	0.49	0	0	PL
1.01	1.13	0.36	0	0	PL
1.14	1.23	0.39	0	0	PL

1.03	1.21	0.3	0	0	IS
0.98	1.13	0.28	0	0	PL
0.97	1.1	0.33	0	0	PL
0.79	0.88	0.23	0	0	IS
0.96	1.15	0.45	0	0	IS
1.11	1.32	0.36	0	0	IS
1.15	1.28	0.32	0	0	IS
1.04	1.35	0.71	0	0	PL
1.01	1.15	0.41	0	0	PL
0.78	1.12	0.58	0	0	PL
0.95	1.1	0.36	0	0	PL
0.8	1.28	0.8	0	0	PL
0.71	1.5	1.17	0	0	IS
1.09	1.28	0.41	0	0	IS
0.92	1.13	0.42	0	0	IS
1.1	1.27	0.49	0	0	PL
0.65	1.22	0.9	0	0	IS
0.96	1.12	0.4	0	0	PL
0.94	1.49	0.82	0	0	PL
1.17	1.31	0.28	0	0	PL
1.05	1.37	0.59	0	0	PL
1.04	1.3	0.53	0	0	PL
1.01	1.19	0.39	0	0	PL
1.12	1.18	0.15	0	0	PL
1.2	1.42	0.31	0	0	IS
0.97	1.22	0.57	0	0	PL
0.64	1.03	0.59	0	0	IS
0.99	1.22	0.42	0	0	IS
0.87	1.15	0.46	0	0	IS
0.72	1.1	0.59	0	0	PL
0.76	1.09	0.7	0	0	PL
1.01	1.22	0.37	0	0	IS
0.88	1.11	0.64	0	0	PL
0.76	0.98	0.49	0	0	PL
0.77	1.4	0.99	0	0	PL
0.89	0.99	0.21	0	0	PL
0.5	0.99	0.63	0	0	PL
0.57	0.96	0.62	0	0	PL
0.8	1.11	0.55	0	0	PL
0.92	1.29	0.69	0	0	IS
1.03	1.27	0.39	0	0	PL
0.81	1.13	0.63	0	0	PL
1.01	1.27	0.48	0	0	IS
0.75	0.97	0.48	0	0	IS
1.43	1.72	0.58	0	0	IS
1.29	1.46	0.42	0	0	PL
0.83	1.16	0.63	0	0	PL
1.09	1.26	0.51	0	0	PL
0.79	0.93	0.37	0	0	IS
1.09	1.36	0.6	0	0	PL
1.01	1.29	0.6	0	0	PL
1.06	1.29	0.43	0	0	PL
1.06	1.36	0.69	0	0	PL
1.28	1.61	0.52	0	0	IS

0.72	1.16	0.59	0	0	IS
0.9	1.11	0.45	0	0	IS
1.22	1.3	0.22	0	0	IS
1.24	1.5	0.44	0	0	IS
0.88	0.98	0.42	0	0	IS
1.2	1.38	0.51	0	0	PL
1.02	1.55	1.07	0	0	PL
1.04	1.46	0.85	0	0	PL
1.04	1.41	0.76	0	0	PL
0.93	1.04	0.23	0	0	IS
1.34	1.48	0.3	0	0	PL
1.2	1.44	0.52	0	0	IS
0.8	0.93	0.3	0	0	PL
0.89	1.07	0.31	0	0	IS
0.58	0.82	0.42	0	0	PL
0.66	0.95	0.51	0	0	PL
0.44	0.68	0.42	0	0	PL
0.82	0.91	0.19	0	0	PL
0.53	0.87	0.56	0	0	PL
0.49	0.67	0.29	0	0	IS
0.35	0.43	0.16	0	0	PL
0.34	0.49	0.23	0	0	PL
0.49	0.77	0.38	0	0	PL
0.44	0.61	0.27	0	0	PL
0.55	0.6	0.09	0	0	PL
0.61	0.87	0.44	0	0	PL
0.43	0.51	0.13	0	0	HC
0.45	0.52	0.15	0	0	PL
0.79	0.96	0.4	0	0	PL
0.54	0.8	0.52	0	0	PL
0.78	0.89	0.29	0	0	PL
0.51	0.89	0.62	0	0	PL
0.76	0.89	0.29	0	0	PL
0.85	0.93	0.23	0	0	IS
0.8	0.95	0.39	0	0	PL
0.92	1.01	0.22	0	0	IS
0.64	0.82	0.41	0	0	PL
0.56	0.86	0.51	0	0	PL
0.78	0.91	0.29	0	0	PL
0.63	0.94	0.56	0	0	PL
0.62	0.85	0.45	0	0	PL
0.67	0.9	0.5	0	0	PL
0.49	0.73	0.42	0	0	PL
0.53	0.73	0.4	0	0	PL
0.61	0.99	0.67	0	0	PL
0.53	0.79	0.45	0	0	PL
0.95	1.09	0.33	0	0	IS
0.68	0.8	0.25	0	0	PL
0.63	0.76	0.26	0	0	PL
0.62	0.72	0.23	0	0	PL
0.48	0.81	0.44	0	0	PL
0.42	0.86	0.61	0	0	PL
0.66	0.84	0.41	0	0	PL
0.74	0.95	0.18	0	0	PL

0.76	0.9	0.3	0	0	PL
0.67	0.95	0.51	0	0	PL
0.98	1.16	0.48	0	0	IS
0.78	0.88	0.28	0	0	PL
0.89	1.27	0.56	0	0	IS
0.76	0.82	0.32	0	0	PL
0.65	0.78	0.24	0	0	PL
0.74	0.86	0.23	0	0	PL
0.53	1.19	0.89	0	0	PL
0.78	1.18	0.66	0	0	PL
0.76	1.17	0.74	0	0	PL
0.67	0.92	0.48	0	0	PL
0.86	1.21	0.58	0	0	IS
0.69	0.9	0.47	0	0	PL
0.72	1.11	0.78	0	0	PL
0.44	1.02	0.78	0	0	PL
0.75	0.84	0.21	0	0	PL
0.94	1.14	0.47	0	0	IS
2.31	2.72	0.63	0	0	IS
0.31	0.36	0.1	0	0	PL
0.4	0.49	0.18	0	0	PL
0.49	0.56	0.13	0	0	PL
0.33	0.59	0.35	0	0	PL
0.41	0.52	0.23	0	0	PL
0.33	0.46	0.21	0	0	PL
0.32	0.43	0.18	0	0	PL
1.87	2.07	0.54	0	0	IS
1.98	2.25	0.47	0	0	IS
0.38	0.58	0.33	0	0	PL
0.47	0.9	0.64	0	0	PL
0.43	0.93	0.7	0	0	PL
0.48	1.03	0.73	0	0	PL
0.57	0.87	0.52	0	0	PL
0.33	0.65	0.45	0	0	PL
0.34	0.57	0.33	0	0	PL
0.45	0.63	0.3	0	0	PL
0.4	0.62	0.4	0	0	PL
0.53	0.86	0.62	0	0	PL
0.52	0.73	0.41	0	0	PL
0.45	0.73	0.48	0	0	PL
0.53	0.66	0.23	0	0	PL
0.66	0.77	0.19	0	0	PL
0.86	1.04	0.42	0	0	PL
0.86	1.08	0.49	0	0	PL
1.02	1.16	0.27	0	0	IS
1.69	2.32	1.01	0	0	IS
0.71	0.89	0.35	0	0	PL
0.66	0.96	0.55	0	0	PL
2.12	2.53	0.73	0	0	IS
0.67	0.94	0.49	0	0	PL
0.99	1.21	0.46	0	0	IS
1.08	1.24	0.4	0	0	PL
0.94	1.15	0.54	0	0	PL
0.89	1	0.21	0	0	PL

0.82	0.96	0.28	0	0	PL
0.85	0.93	0.2	0	0	PL
2.05	2.62	0.91	0	0	IS
0.83	0.96	0.25	0	0	PL
0.66	1.06	0.59	0	0	IS
0.77	1.14	0.51	0	0	IS
2.04	2.46	0.67	0	0	IS
1.86	2.42	0.97	0	0	IS
0.92	1.56	1.02	0	0	IS
0.94	1.29	0.66	0	0	PL
1.75	2.27	0.86	0	0	IS
0.97	1.12	0.48	0	0	PL
0.96	1.14	0.38	0	0	IS
1.15	1.32	0.33	0	0	IS
1.09	1.2	0.41	0	0	IS
1.11	1.26	0.45	0	0	IS
0.82	1.07	0.49	0	0	PL
1.04	1.18	0.3	0	0	IS
0.88	1.35	0.91	0	0	IS
2.03	2.42	0.82	0	0	IS
0.45	0.83	0.49	0	0	PL
2.32	2.73	0.71	0	0	IS
2.56	3.19	1.05	0	0	IS
0.55	0.86	0.51	0	0	IS
0.59	0.78	0.35	0	0	PL
0.51	0.65	0.31	0	0	IS
0.45	0.58	0.24	0	0	IS
0.37	0.65	0.36	0	0	PL
0.38	0.5	0.15	0	0	IS
0.43	0.62	0.27	0	0	PL
0.61	1.03	0.75	0	0	PL
1.97	2.99	1.62	0	0	IS
0.78	0.98	0.38	0	0	IS
3.4	4.34	1.8	0	0	IS
0.54	1.12	0.76	0	0	PL
3.17	3.9	1.63	0	0	IS
0.84	1.26	0.59	0	0	IS
3.06	3.66	1.44	0	0	IS
0.87	0.91	0.08	0	0	IS
2.78	3.66	1.52	0	0	IS
2.76	3.72	1.63	0	0	IS
0.73	0.84	0.31	0	0	IS
2.53	3.66	1.55	0	0	IS
0.89	0.98	0.23	0	0	PL
0.9	1.06	0.3	0	0	PL
0.96	1.21	0.43	0	0	IS
0.58	0.9	0.53	0	0	PL
0.57	0.84	0.46	0	0	PL
0.51	0.54	0.04	0	0	IS
0.57	0.73	0.31	0	0	IS
0.72	0.97	0.54	0	0	IS
0.71	0.77	0.11	0	0	OC
2.8	3.64	1.51	0	0	IS
0.6	0.71	0.24	0	0	OC

2.77	3.61	1.57	0	0	IS
0.88	1.18	0.59	0	0	IS
2.87	3.85	1.53	0	0	IS
0.98	1.13	0.28	0	0	IS
1.16	1.18	0.05	4	21.74	HC
1.19	1.97	0.48	0	0	PL
2.71	3.46	1.28	0	0	IS
1	1.28	0.6	0	0	IS
0.7	1.06	0.88	0	0	IS
1.04	1.14	0.33	0	0	PL
2.85	3.31	1.04	0	0	IS
2.91	3.83	1.59	0	0	IS
3.01	3.73	1.51	0	0	IS
2.81	3.74	1.64	0	0	IS
0.73	1.53	1.26	0	0	IS
1.15	1.26	0.22	0	0	PL
2.84	3.92	1.53	0	0	IS
1.07	1.19	0.29	0	0	PL
0.9	1.12	0.69	0	0	PL
2.68	4.11	1.9	0	0	IS
1.25	1.45	0.4	0	0	PL
0.78	1.26	1.18	0	0	IS
0.88	1.27	0.79	0	0	PL
0.8	1.34	0.91	0	0	PL
0.93	1.03	0.32	0	0	PL
1.01	1.1	0.23	0	0	PL
0.83	1.11	0.59	0	0	PL
1.01	1.2	0.47	0	0	OC
1.07	1.15	0.25	0	0	PL
2.8	3.91	1.87	0	0	IS
0.83	1.31	0.91	0	0	IS
0.97	1.32	0.83	0	0	IS
0.53	1.37	1.06	0	0	IS
2.81	4.11	2.08	0	0	IS
2.78	3.75	1.73	0	0	IS
0.86	1.06	0.48	0	0	PL
0.85	1.04	0.47	0	0	PL
0.83	0.96	0.28	0	0	PL
0.8	0.99	0.4	0	0	PL
3.04	4.3	2.03	0	0	IS
3	3.74	1.65	0	0	IS
0.55	0.74	0.4	0	0	PL
0.81	1.02	0.39	0	0	PL
0.61	0.82	0.42	0	0	PL
1.02	1.25	0.53	0	0	PL
1.09	1.38	0.59	0	0	PL
0.83	1.17	0.7	0	0	PL
0.72	1.2	0.85	0	0	PL
2.32	2.78	0.72	0	0	IS
1.19	1.4	0.39	0	0	PL
2.28	2.96	1.16	0	0	IS
1.31	1.44	0.27	0	0	PL
2.19	2.58	0.58	0	0	IS
2.43	2.84	0.82	0	0	IS

0.67	1.12	0.75	0	0	PL
2.22	2.54	0.63	0	0	IS
0.75	1.18	0.79	0	0	PL
0.91	1.21	0.66	0	0	PL
1.1	1.32	0.46	0	0	IS
1.3	1.47	0.29	0	0	IS
2.2	2.74	0.8	0	0	IS
1.2	1.3	0.31	0	0	PL
1.03	1.41	0.56	0	0	IS
1.28	1.45	0.44	0	0	PL
2.5	3.13	0.98	0	0	IS
2.21	2.99	1.13	0	0	IS
2.5	3.1	1.05	0	0	IS
1.07	1.33	0.67	0	0	IS
1.17	1.59	0.92	0	0	HC
1.14	1.49	0.77	0	0	IS
1.02	1.47	0.85	0	0	PL
1.19	1.65	0.67	0	0	IS
1.42	1.65	0.4	0	0	IS
1.07	1.35	0.84	0	0	IS
1.35	1.54	0.36	0	0	PL
2.87	3.16	0.96	0	0	IS
1.17	1.56	0.74	0	0	PL
2.25	2.69	0.67	0	0	IS
2.29	2.73	0.7	0	0	IS
1.03	1.19	0.35	0	0	PL
0.93	1.13	0.31	0	0	IS
0.87	1.06	0.39	0	0	PL
0.92	1.08	0.26	0	0	PL
0.9	1.28	0.69	0	0	IS
0.95	1.12	0.28	0	0	IS
0.53	1.31	1.01	0	0	PL
0.71	1.06	0.72	0	0	PL
0.83	1.1	0.62	0	0	PL
1.03	1.28	0.34	0	0	IS
1.09	1.22	0.27	0	0	PL
2.41	2.94	0.8	0	0	IS
1.07	1.17	0.3	0	0	PL
0.74	1.3	0.86	0	0	IS
0.89	1.03	0.42	0	0	PL
2.46	2.85	0.71	0	0	IS
1.03	1.12	0.26	0	0	PL
0.99	1.15	0.38	0	0	PL
1.05	1.18	0.21	0	0	IS
0.95	1.07	0.24	0	0	IS
0.9	1.04	0.35	0	0	IS
1	1.14	0.25	0	0	IS
0.83	1.02	0.52	0	0	PL
0.78	1.08	0.6	0	0	PL
0.98	1.19	0.35	0	0	IS
0.7	1.23	0.87	0	0	PL
0.92	1.28	0.69	0	0	PL
1.05	1.28	0.46	0	0	PL
1.06	1.18	0.35	0	0	PL

1.12	1.22	0.3	0	0	PL
1.06	1.24	0.36	0	0	IS
0.9	1.31	0.65	0	0	IS
0.92	1.28	0.69	0	0	PL
1.07	1.29	0.36	0	0	IS
0.93	1.09	0.4	0	0	PL
0.88	1.15	0.64	0	0	PL
0.87	1.07	0.4	0	0	PL
0.95	1.18	0.48	0	0	IS
0.94	1.17	0.5	0	0	PL
0.98	1.17	0.42	0	0	PL
1.03	1.26	0.51	0	0	PL
1.05	1.22	0.37	0	0	PL
1.09	1.27	0.41	0	0	IS
1.2	1.31	0.2	0	0	PL
1.04	1.23	0.51	0	0	PL
0.92	1.2	0.51	0	0	PL
2.43	3.19	1.16	0	0	IS
1.1	1.31	0.4	0	0	PL
1.05	1.24	0.32	0	0	IS
1.02	1.17	0.39	0	0	PL
1.2	1.34	0.26	0	0	IS
0.5	0.9	0.63	0	0	PL
0.57	1.1	0.83	0	0	PL
0.56	0.99	0.72	0	0	PL
0.82	0.96	0.36	0	0	PL
0.65	0.86	0.4	0	0	PL
0.67	0.82	0.32	0	0	PL
2.28	3.07	1.07	0	0	IS
2.43	3.46	1.51	0	0	IS
0.58	0.77	0.37	0	0	PL
0.89	0.99	0.19	0	0	PL
0.6	1.15	0.73	0	0	IS
0.69	0.92	0.57	0	0	PL
0.88	0.98	0.19	0	0	PL
0.94	1.02	0.23	0	0	PL
0.9	0.96	0.21	0	0	PL
0.84	0.96	0.31	0	0	PL
2.4	3.08	1.08	0	0	IS
0.72	0.94	0.49	0	0	PL
2.67	3.33	1.24	0	0	IS
2.68	3.55	1.49	0	0	IS
0.76	1.03	0.61	0	0	PL
0.91	1.02	0.23	0	0	IS
0.8	0.95	0.41	0	0	PL
0.82	0.99	0.37	0	0	IS
0.56	0.78	0.4	0	0	PL
2.46	3.58	1.68	0	0	IS
2.62	3.41	1.39	0	0	IS
0.65	1.31	1.02	0	0	PL
0.61	1.24	0.99	0	0	PL
0.48	1.12	0.85	0	0	OC
0.79	0.98	0.46	0	0	PL
0.82	1.01	0.49	0	0	PL

0.96	1.04	0.25	0	0	PL
2.45	3.15	1.15	0	0	IS
0.85	1.02	0.48	0	0	PL
0.66	0.95	0.64	0	0	PL
2.39	3.3	1.11	0	0	IS
0.77	0.95	0.62	0	0	PL
2.17	2.93	0.97	0	0	IS
2.47	2.81	0.7	0	0	IS
0.71	0.9	0.44	0	0	PL
0.81	0.94	0.24	0	0	PL
0.76	0.97	0.45	0	0	PL
0.59	1.05	0.69	0	0	PL
0.55	0.99	0.71	0	0	PL
0.52	0.82	0.54	0	0	PL
0.6	0.83	0.45	0	0	IS
0.8	0.91	0.35	0	0	PL
0.78	0.89	0.27	0	0	PL
0.78	0.85	0.22	0	0	PL
2.46	3.27	1.26	0	0	IS
0.73	0.84	0.26	0	0	PL
0.66	1.05	0.75	0	0	PL
0.78	0.91	0.29	0	0	PL
0.69	0.89	0.4	0	0	PL
0.59	0.79	0.33	0	0	IS
0.75	0.91	0.29	0	0	PL
2.14	2.93	1	0	0	IS
0.7	0.97	0.45	0	0	PL
0.78	0.9	0.4	0	0	PL
0.77	1.07	0.34	0	0	IS
0.91	1.02	0.26	0	0	PL
0.97	1.15	0.43	0	0	PL
2.35	3.2	1.22	0	0	IS
1.03	1.14	0.22	0	0	IS
0.93	1.07	0.26	0	0	IS
3.02	3.85	1.58	0	0	IS
1.21	1.36	0.43	0	0	PL
3.14	3.89	1.85	0	0	IS
0.72	1.18	0.67	0	0	IS
1.71	2.15	0.79	0	0	IS
0.93	1.11	0.48	0	0	PL
0.91	1.03	0.3	0	0	PL
1.03	1.29	0.53	0	0	PL
0.85	1.1	0.52	0	0	PL
0.72	1.08	0.63	0	0	PL
