# Supplementary material for: Behavioral-psychological motivations encoded in the vocal repertoire of captive Amur tiger (Panthera tigris altaica) cubs
Source: BMC Zool. 2022 Jan 4;7:2. doi: 10.1186/s40850-021-00102-9 (PMC10127000; doi:10.1186/s40850-021-00102-9)
Supplement: Supplementary file 2 — Additional file 2: Table S2. Discriminant function analysis with Principal Component scores for the classification of nine call types. [file 40850_2021_102_MOESM2_ESM.pdf]

S1 Table. Discriminant function analysis with Principal Component scores for classification of nine call types.

		Call type									Total
		Ar-1	***Chuff***	***eee***	***Er***	***Growl***	***Haer***	***Hiss***	***Roar***	***Ar-2***	
**Number of Correct Classification**	***Ar-1***	1631	0	0	0	0	0	0	5	89	1725
	Chuff	0	428	0	0	0	0	0	0	0	428
	eee	0	2	66	0	37	0	0	0	0	105
	Er	0	0	0	214	0	48	0	296	70	628
	Growl	0	12	15	0	40	0	0	0	0	67
	Haer	2	0	0	68	0	29	0	21	6	126
	Hiss	0	0	0	0	0	0	208	0	3	211
	Roar	0	0	0	7	0	11	0	69	4	91
	Ar-2	58	0	0	222	0	109	2	414	1149	1954
**Rate of Correct Classification**	***Ar-1***	94.6	0	0	0	0	0	0	0.3	5.2	100
	Chuff	0	100.0	0	0	0	0	0	0	0	100
	eee	0	1.9	62.9	0	35.2	0	0	0	0	100
	Er	0	0	0	34.1	0	7.6	0	47.1	11.1	100
	Growl	0	17.9	22.4	0	59.7	0	0	0	0	100
	Haer	1.6	0	0	54.0	0	23.0	0	16.7	4.8	100
	Hiss	0	0	0	0	0	0	98.6	0	1.4	100
	Roar	0	0	0	7.7	0	12.1	0	75.8	4.4	100
	Ar-2	3.0	0	0	11.4	0	5.6	0.1	21.2	58.8	100
